# Arctic cloud condensation nuclei enhanced by iodine, sulfur and organic precursors

**DOI:** 10.1038/s41561-026-02062-6

**Published:** 2026-08-05

**Authors:** Mao Du, James Brean, Douglas R. Worsnop, Congbo Song, Yangmei Zhang, Vipul Lal Chandani, Deepchandra Srivastava, David C. S. Beddows, W. Joe F. Acton, Darrel Baumgardner, Jo Browse, Anna B. Callaghan, Manjula Canagaratna, Yuqing Dai, Pete M. Edwards, Jingkun Jiang, Thomas M. Jordan, James D. Lee, Roberto Sommariva, Harald Stark, Mark D. Tarn, Loren G. Temple, Gavin H. Tilstone, Mingxi Yang, William J. Bloss, Roy M. Harrison, Manuel Dall’Osto, Zongbo Shi

**Affiliations:** 1 School of Geography Earth and Environment Sciences, University of Birmingham, Birmingham, UK; 2 Institute for Atmospheric and Earth System Research/Physics, Faculty of Science, University of Helsinki, Helsinki, Finland; 3 Aerodyne Research, Billerica, MA USA; 4 State Key Laboratory of Severe Weather, Key Laboratory of Atmospheric Chemistry of CMA, Chinese Academy of Meteorological Sciences, Beijing, China; 5 Droplet Measurement Technologies, LLC, Longmont, CO USA; 6 Centre for Geography and Environmental Science, University of Exeter, Penryn, UK; 7 Wolfson Atmospheric Chemistry Laboratories, Department of Chemistry, University of York, York, UK; 8 National Centre for Atmospheric Science, University of York, York, UK; 9 State Key Laboratory of Regional Environment and Sustainability, School of Environment, Tsinghua University, Beijing, China; 10 Plymouth Marine Laboratory, Plymouth, UK; 11 CIRES and CU Boulder, Boulder, CO USA; 12 School of Earth, Environment and Sustainability, University of Leeds, Leeds, UK; 13 Institute of Marine Science, Consejo Superior de Investigaciones Científicas, Barcelona, Spain; 14 Anhui Institute of Optics and Fine Mechanics, Chinese Academy of Sciences, Hefei, China; 15 Present Address: National Centre for Atmospheric Science, University of Manchester, Manchester, UK; 16 Present Address: Department of Environmental Sciences, Faculty of Meteorology, Environment and Arid Land Agriculture, King Abdulaziz University, Jeddah, Saudi Arabia

**Keywords:** Atmospheric chemistry, Atmospheric science

## Abstract

New particle formation is an important source of Arctic atmospheric particles and cloud condensation nuclei, yet their precursor sources and molecular-level mechanisms remain poorly understood. Here we report comprehensive ship-based observations from 19 May to 26 June 2022 from southeastern to western Greenland and into the Davis Strait’s marginal ice zone to investigate sources and processes controlling atmospheric particles and cloud condensation nuclei. Our observations provide field evidence of frequent nucleation events driven by the multicomponent iodine oxoacid and sulfuric acid mechanism recently identified in laboratory studies. Newly formed particles grew rapidly beyond 20 nm on 8 out of 13 nucleation days, mainly driven by oxygenated organic molecules from aldehyde and monoterpene oxidation. We also report a previously unobserved class of iodine-containing oxygenated organic molecules that contributed to particle growth and enhanced cloud condensation nuclei formation. We show that marginal sea ice zone produces precursors that drive rapid new particle formation and enhance cloud condensation nuclei concentrations by up to 50-fold. Our findings demonstrate that Arctic iodine, sulfur and organic precursors can enhance cloud condensation nuclei abundance through new particle formation, highlighting a potential but unquantified pathway for influencing cloud cover, radiative balance and the hydrological cycle.

## Abstract (web summary)

Frequent Arctic nucleation events arise from coupled iodine and sulfur chemistry, with organics driving rapid growth. Ship-based observations show that this process can increase cloud condensation nuclei by up to 50-fold near marginal sea ice.

## Main

Over the past 40 years, Arctic surface temperature has risen more than three times faster than the global average^1,2^. Aerosol particles play an important but uncertain role in this phenomenon: they both reflect and absorb solar radiation, and act as cloud condensation nuclei (CCN), thereby affecting cloud radiative properties^3–5^. New particle formation (NPF), involving nucleation of gases to form new particles and their subsequent growth, is considered an important source of Arctic CCN in summer^6,7^. However, climate models consistently struggle to accurately reproduce observations of Arctic aerosols and CCN, largely ascribed to a limited understanding of precursor sources and NPF mechanisms^5,8,9^, limiting their ability to model clouds.

Although nucleation and growth of new particles have been observed across the Arctic^7,10–15^, it is a diverse region with a complex biome consisting of various ecosystems that emit multiple gases, whose photochemical reaction products participate in NPF. Recent Arctic studies showed that dominant nucleation mechanisms differ at different locations and seasons^13,14^ (Supplementary Fig. 1a and Supplementary Text 1.1). For example, sulfuric acid (H_2_SO_4_) and ammonia (NH_3_) have been reported to be important for nucleation at Svalbard, while nucleation in the high Arctic has been attributed primarily to iodic acid (HIO_3_)^14^.

As part of extensive work unveiling the mechanisms of nucleation and growth in a variety of chemical systems, recent CERN Cosmics Leaving OUtdoor Droplets (CLOUD) chamber measurements discovered multicomponent nucleation (that is, involving more than two components simultaneously, excluding H_2_O)^16^ of iodine oxoacids (HIO_*x*_ = HIO_3_, and iodous acid, HIO_2_) and H_2_SO_4_. They can synergistically form new particles, even in the absence of alkaline gases^17^, with HIO_*x*_ enhancing H_2_SO_4_–NH_3_ nucleation rates by factors of 10–10,000 (ref. ^17^). This challenges the prevailing view on the dominant role of H_2_SO_4_–NH_3_ or HIO_3_ nucleation as independent pathways at high latitudes^6,8,13,14^ (Supplementary Fig. 1a and Supplementary Text 1.1). However, this laboratory-derived mechanism remains unvalidated by real-world observations, essential to confirm atmospheric relevance and/or importance of the laboratory findings^18–20^.

Particle growth in the Arctic has been attributed variously to H_2_SO_4_ and methanesulfonic acid (MSA) at Svalbard^13^, HIO_3_ in central Arctic^14^ and, hypothesized but not definitively confirmed, oxygenated organic molecules (OOMs: organic molecules with more than four carbon and more than three oxygen atoms) and marine-originated oxygenated volatile organic compounds (OVOCs: volatile organic compounds (VOCs) with one to three oxygen atoms)^11,15,21–23^ (Supplementary Fig. 1b and Supplementary Text 1.2). Existing studies suggest that NPF contributes to Arctic CCN^13,14,24^ (Supplementary Text 1.3), but none has followed the same particle population from molecular clusters through growth to directly measured CCN. Such closure is increasingly important given the rapidly changing Arctic environment, including rising emissions of iodine and dimethylsulfide (DMS)^12,25–28^.

Here, using comprehensive measurements aboard the RRS *Discovery* during the DY151 campaign (19 May to 26 June 2022), we provide real-world evidence that Arctic nucleation is driven by synergistic H_2_SO_4_ and HIO_*x*_ clustering. Furthermore, we show that subsequent growth to CCN is dominated by condensation of OOMs, including a previously unobserved class of iodine-containing OOMs. By directly linking emissions from the marginal ice zone to a substantial increase in CCN, our findings provide a complete observational closure from precursors to cloud- and climate-relevant particles.

## Characteristics of nucleation events

**Fig. 1:**
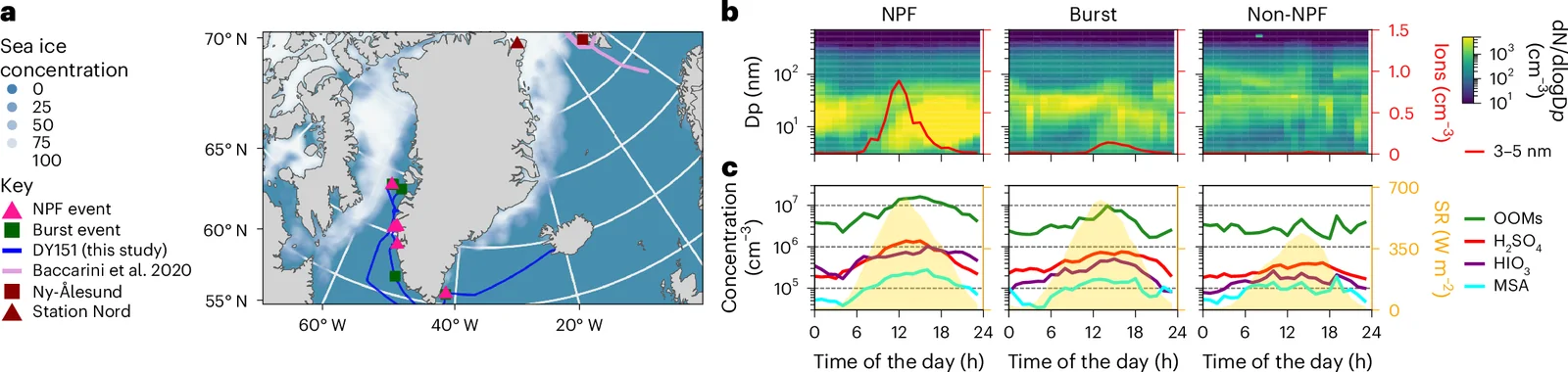
Arctic NPF phenomenology. **a**, Map of the Arctic coloured by sea ice concentration (June 2022). Blue line indicates the ship track during the campaign. Pink triangles and green squares represent locations of NPF and burst events, respectively. Dark-red triangle and square, and purple trace, represent locations of previous studies^13,14^. **b**, Diel cycles of PNSD and ion concentrations between 3 nm and 5 nm on all NPF event (8 days), burst event (5 days) and non-event (8 days) days. D_p_, particle diameter. Days with extensive pollution from our ship (Supplementary Text 2) are excluded from the data analysis. The PNSD is derived from the NAIS (3–25 nm), and two MPSSs (25–40 nm and 40–736 nm). The 3–5 nm ion number concentration is from the NAIS negative channel. **c**, Diel cycle of acid (H_2_SO_4_, HIO_3_ and MSA) and OOM concentrations, measured by nitrate-chemical ionization mass spectrometer (NO_3_^−^ CIMS), alongside SR (solar radiation) on NPF, burst and non-event event days. Source data

**Extended Data Fig. 1:**
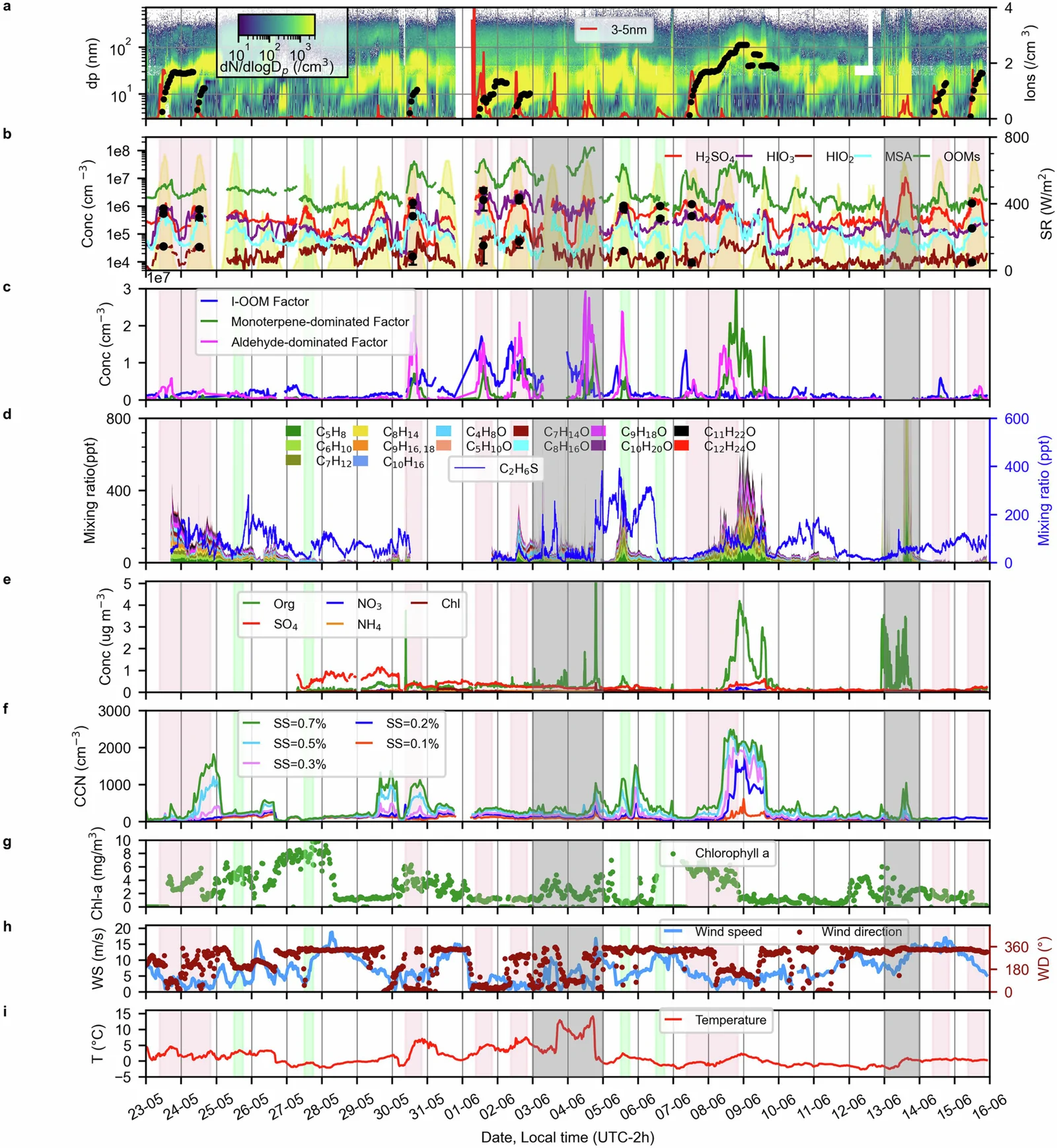
Measurement overview from 23 May to 16 June 2022. (**a**) Particle number size distribution (heatmap), ion concentrations (red trace), and modal diameters during NPF events (black points), (**b**) acid and oxygenated organic molecule concentrations, alongside solar radiation (yellow shaded region), with black dots denoting the data used for Fig. 3b, (**c**) PMF factors, (**d**) PTR-MS ions, (**e**) AMS species, (**f**) CCN number concentration, (**g**) chlorophyll-a, (**h**) wind speed and direction, and (**i**) temperature measured during the campaign. Pink shaded periods indicate NPF event days, green shaded periods indicate burst event days, and gray shaded periods indicate periods affected by ship-stack contamination. Blank periods indicate missing data, such as instrument downtime. Source data

Figure 1a shows sea ice concentrations across the Arctic during June 2022, overlaid with the cruise track; the expedition focused on the understudied region around the Davis Strait’s melting sea ice zone (Methods). A suite of instruments measured particle number size distributions (PNSD), gas-phase NPF precursors, including H_2_SO_4_, MSA and HIO_*x*_, VOCs and OOMs, as well as other gaseous pollutants and aerosol composition (Fig. 1, Extended Data Fig. 1, Supplementary Fig. 2 and Supplementary Methods 2–9). Background concentrations of NO_*x*_ (<50 pptv), SO_2_ (median 23 pptv) and black carbon (BC) (1.9 ng m^−3^) were very low, indicating pristine marine conditions. Days with identifiable pollution from the ship were excluded from data analysis (Supplementary Text 2 and Supplementary Fig. 2).

Based on the criteria detailed in Supplementary Methods 10, nucleation events were observed on 13 days, accounting for 81% of all days when peak hourly solar radiation exceeded 600 W m^−2^. Among the nucleation days, eight showed particle growth to sizes greater than 20 nm (Extended Data Fig. 1), of which six days had measurable growth rates (GR; Supplementary Methods 11). Nucleation days with observable growth are referred to as ‘NPF events’, while those without growth are termed ‘burst events’, characterized by a burst of sub-10-nm particle number; days with no evidence of nucleation are defined as ‘non-event days’.

**Extended Data Fig. 2:**
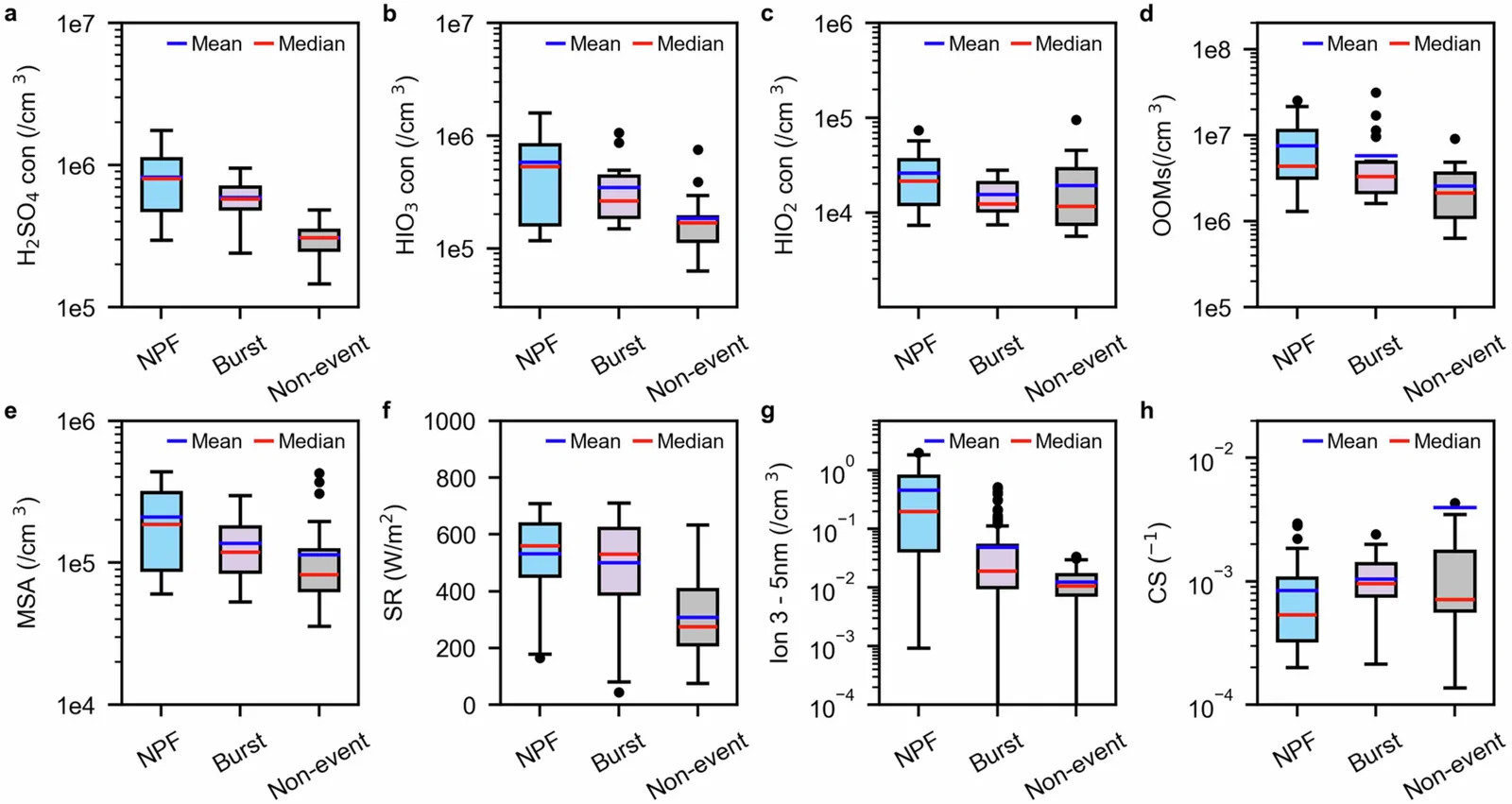
Box plots comparing NPF, burst, and non-event periods. Box plots show (**a**) H_2_SO_4_, (**b**) HIO_3_, (**c**) HIO_2_, (**d**) OOMs, (**e**) MSA, (**f**) solar radiation, (**g**) 3–5 nm ion number concentration, and (**h**) condensation sink for NPF, burst, and non-event days. Red lines indicate medians, blue lines indicate means, boxes represent the 25th to 75th percentiles, whiskers extend to 1.5 times the interquartile range, and black dots indicate outliers. Data are from hourly measurements between 10:00 and 15:00 for 8 NPF event days, 5 burst event days, and 8 non-event days, excluding periods dominated by ship emissions. Source data

NPF and burst events, defined by 3–5 nm ion concentrations as a metric for NPF event intensity^29^, occurred under elevated concentrations of OOMs, H_2_SO_4_, HIO_3_ and MSA relative to non-event days (Fig. 1b,c and Extended Data Fig. 2). Median condensable OOM concentrations were ~40% lower during burst than during NPF events. Condensation sinks were lower on NPF and burst days (7 × 10^−4^ s^−1^ and 5 × 10^−4^) versus non-event days (1 × 10^−3^ s^−1^; Extended Data Fig. 2 and Supplementary Table 1).

## Multicomponent nucleation

**Fig. 2:**
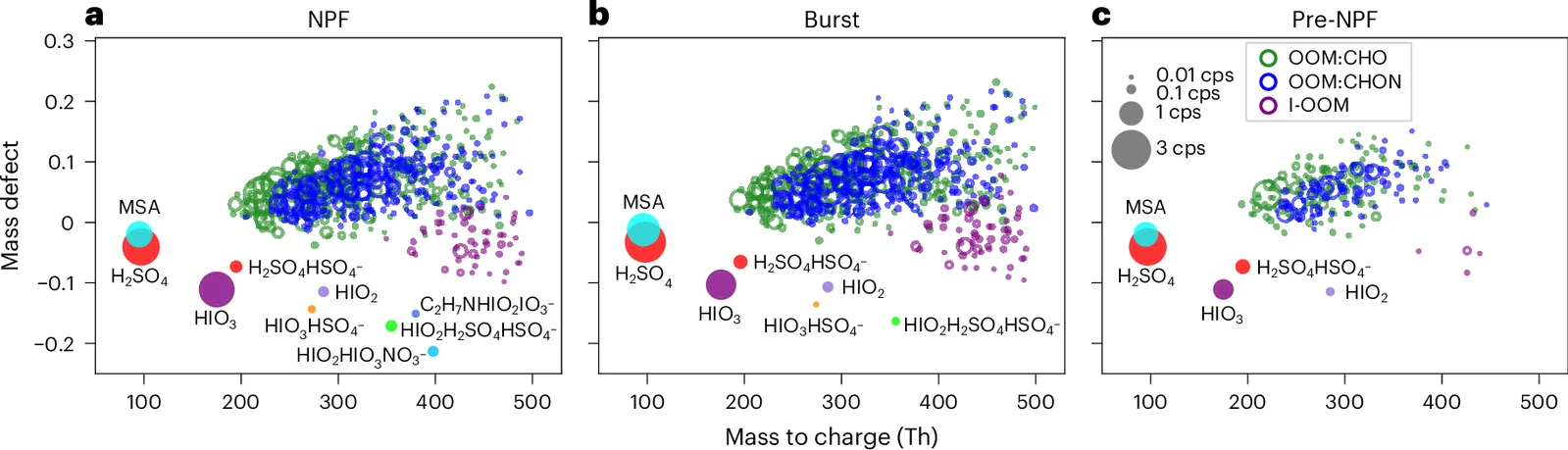
Molecular composition of detected clusters during nucleation and background periods. **a**–**c**, Mass defect plots showing neutral molecules and clusters detected by NO_3_^−^ CIMS, averaged across NPF events (**a**), burst events (**b**) and pre-NPF background periods (**c**; typically from 2:00 to 4:00, when photochemistry is minimal). Each circle represents a detected species; its position on the *y* axis reflects the difference between its exact and nominal mass, a fingerprint that helps identify molecular composition, and its size is proportional to signal intensity (cps, counts per second). Key species containing H_2_SO_4_, HIO_3_ and HIO_2_ are labelled. H_2_SO_4_, MSA, HIO_3_ and HIO_2_ are reported as the sum of their deprotonated and clustered forms; individual ions are listed in Supplementary Table 2. Source data

**Extended Data Fig. 3:**
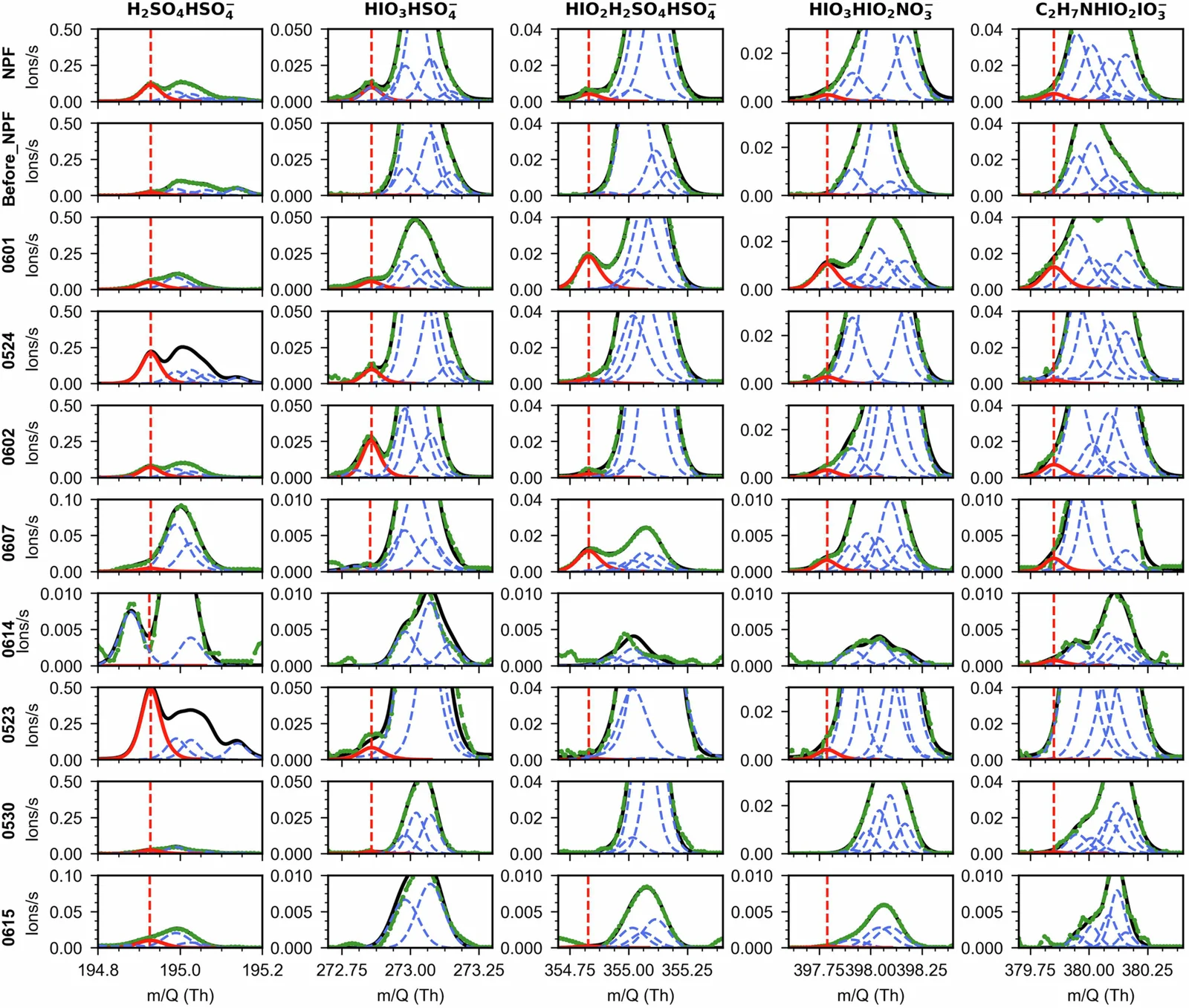
High-resolution peak fits for H2SO4 dimers and clusters. High-resolution peak fits are shown for H_2_SO_4_ dimers and clusters, including HIO_3_-HSO_4_^−^, H_2_SO_4_-HSO_4_^−^, HIO_3_^−^HIO_2_-NO_3_^−^, HIO_2_-H_2_SO_4_-HSO_4_^−^, and C_2_H_7_N-HIO_2_-IO_3_^−^. Spectra are presented for individual NPF events, averaged NPF periods, and pre-NPF periods measured by NO_3_^−^ CIMS. Red lines indicate target cluster peaks, dashed blue lines indicate other peaks, and missing red lines indicate undetected target peaks. Panels are sorted vertically by descending J_1.7_. Before NPF was typically the window from 02:00 to 04:00, as in Fig. 2. Source data

**Extended Data Fig. 4:**
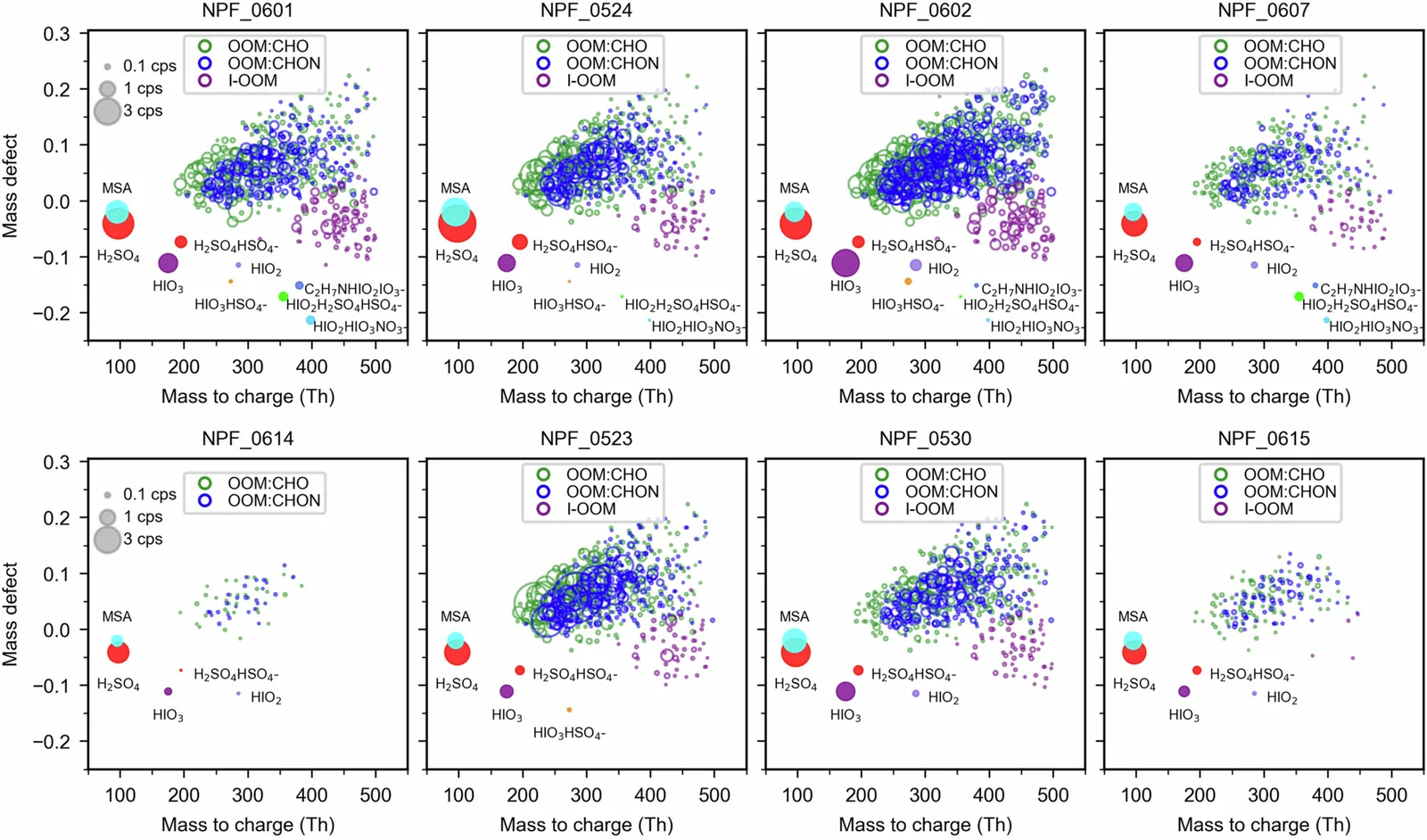
Mass defect plots of neutral molecules and clusters for individual NPF events. Mass defect plots show neutral molecules and clusters observed during individual NPF events. Mass defect is calculated as the difference between each species’ exact mass and the nearest integer mass. Circle size is proportional to observed signal intensity. H_2_SO_4_, MSA, HIO_3_, and HIO_2_ are reported as the sum of their deprotonated and clustered forms, with individual ions listed in Table S2. Source data

Clusters containing H_2_SO_4_, HIO_3_ and HIO_2_ were detected during nucleation events (Fig. 2a,b and Supplementary Table 2), including dimers HIO_3_–HSO_4_^−^ and HIO_2_–HIO_3_–NO_3_^−^, as well as the occasional detection of trimers HIO_2_–H_2_SO_4_–HSO_4_^−^ and C_2_H_7_N–HIO_2_–IO_3_^−^ (Fig. 2, Extended Data Figs. 3 and 4 and Supplementary Methods 3). As NO_3_^−^ in HIO_2_–HIO_3_–NO_3_^−^ is a charger ion introduced by the mass spectrometer^17^, this ion represents a cluster of HIO_*x*_. These clusters are similar to those identified in previous Arctic studies of mixtures of H_2_SO_4_ and HIO_*x*_ (refs. ^13,14^). Three of these clusters were detectable during burst events with signals lower than those on NPF days, while none of these clusters was detected before NPF began (Fig. 2c). A day-by-day analysis (Extended Data Figs. 3 and 4) shows at least one cluster containing both HIO_*x*_ and H_2_SO_4_ on nearly every NPF day, confirming consistent co-involvement of both precursor systems despite variable detectability of individual species.

**Fig. 3:**
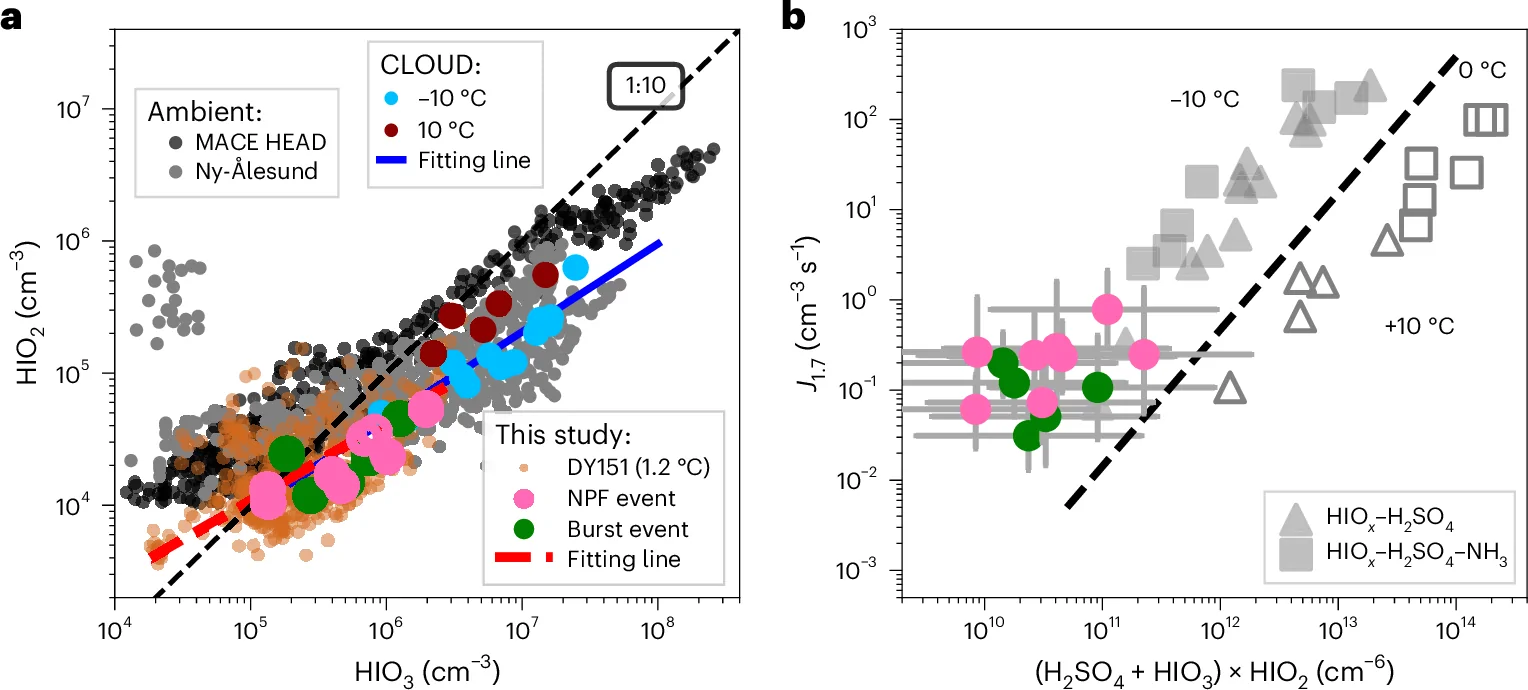
Multicomponent nucleation involving HIO_*x*_. **a**, Scatter plot of HIO_2_ versus HIO_3_ from our measurements compared with data from the CLOUD chamber, Mace Head and Ny-Ålesund^17^. The dashed red line represents a total least-squares regression fit for our data, yielding an equation of log_10_(HIO_2_) = 0.6 × log_10_(HIO_3_) + 1.05, consistent with the CLOUD fitting line (blue line). Pink and green filled circles represent the mean values during the nucleation period for NPF and burst events, respectively. Two hollow pink circles indicate two events, 23 and 24 May, where HIO_2_ concentrations were estimated by the aforementioned fitting equation. The average temperature throughout the entire campaign was 1.2 °C. **b**, *J*_1.7_ versus (HIO_3_ + H_2_SO_4_) × HIO_2_ for all measured nucleation events, together with CLOUD data. The dashed line in **b** is parallel to and has the same distance to the regression lines of the J_1.7_ and (HIO_3_ + H_2_SO_4_) × HIO_2_ at temperatures of 10 °C and −10 °C. Assuming a linear relationship, this line represents the expected value at 0 °C. Pink and green data points in **b** represent average values during each nucleation event. Error bars represent one standard deviation of the mean value. The hollow circle in the figure represents the HIO_2_ values estimated based on the fitting equation shown above. See Supplementary Methods 3 and Supplementary Text 3 for estimates of uncertainties in concentration measurements and nucleation rate, as well as limits of detection. Source data

HIO_3_ and HIO_2_ concentrations were well correlated, with median concentration 2 × 10^5^ cm^−3^ for HIO_3_ and 1.6 × 10^4^ cm^−3^ for HIO_2_. The power-law relationship between them (Fig. 3a) agrees with He et al.^17^, indicating that the HIO_2_:HIO_3_ ratio varies with concentration, here at concentrations over an order of magnitude lower. Using this relationship, we applied the more readily observed HIO_3_ to estimate HIO_2_ concentrations when they were not detectable on 23–24 May (Extended Data Fig. 1).

**Extended Data Fig. 5:**
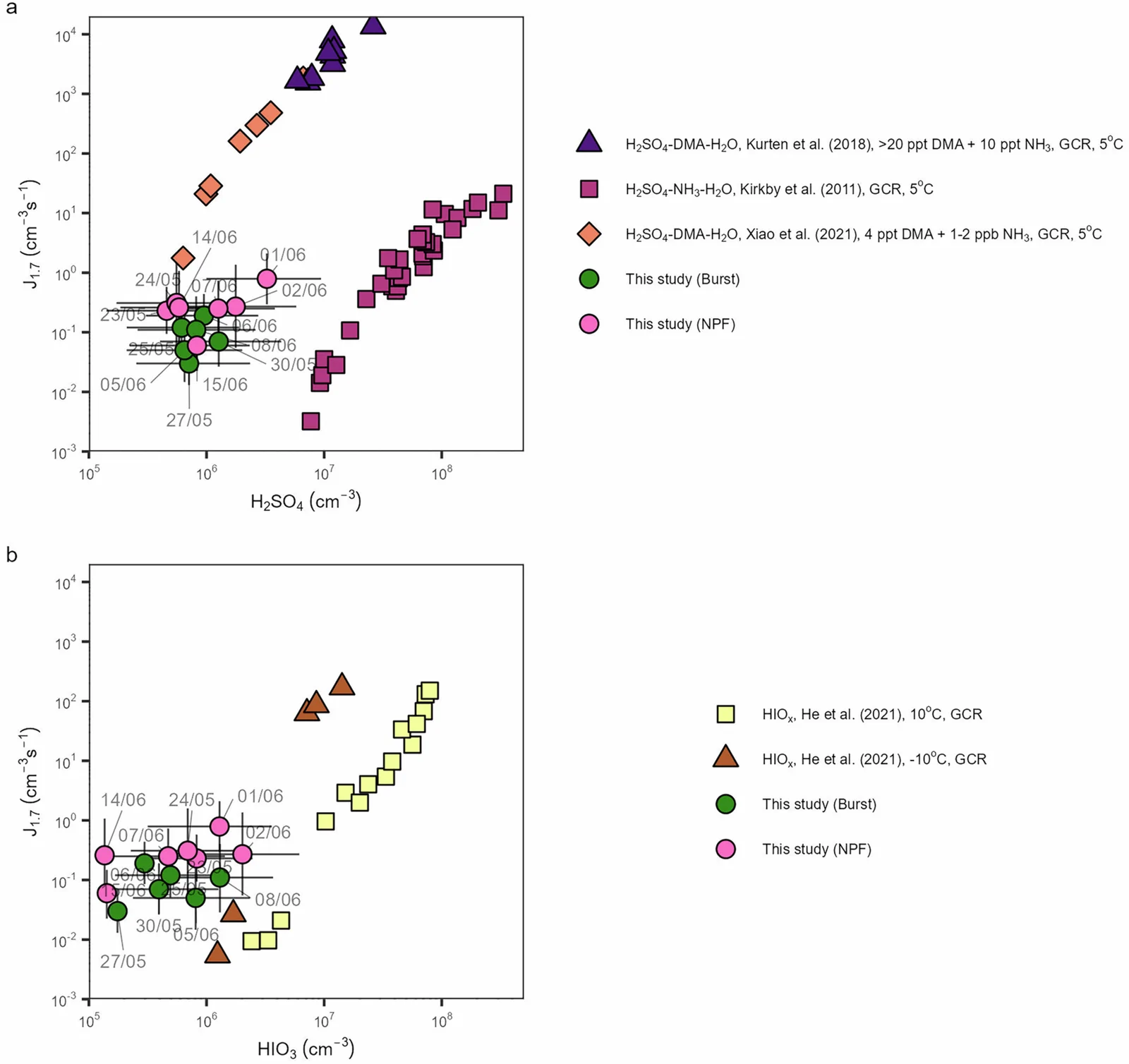
Comparison of nucleation rates for H_2_SO_4_ and HIO_3_ systems with previous studies. Particle formation rates J_1.7_ are compared with H_2_SO_4_ and HIO_3_ concentrations. Panel **a** shows J_1.7_ against H_2_SO_4_ concentrations and includes CLOUD chamber data for H_2_SO_4_-DMA-H_2_O and H_2_SO_4_-NH_3_^−^H_2_O systems. Panel **b** shows J_1.7_ against HIO_3_ concentrations and includes CLOUD chamber data at 10 °C and −10 °C. Pink circles represent NPF events from this study, green circles represent burst events, and each point is labelled by date in dd/mm format. Source data

The peak hourly formation rate at 1.7 nm (*J*_1.7_) for each day varied from 0.04 to 1.4 cm^−3^ s^−1^, in line with values at Svalbard of 0.33 (*J*_1.5_)^13^ and 0.04 (*J*_3_)^30^. Our observed formation rate at 10 nm (*J*_10_) is within the observed range in Arctic^10^, although the median is relatively high (Supplementary Fig. 3). The measured H_2_SO_4_ concentration is too low to explain *J*_1.7_ when compared with CLOUD experiments involving H_2_SO_4_–NH_3_–H_2_O or H_2_SO_4_–DMA–H_2_O (<5 pptv DMA, 1–2 pptv NH_3_)^31^. Our data similarly do not match experiments with 4 pptv DMA and 10 pptv NH_3_ (Extended Data Fig. 5a)^32^. NH_3_ was below the detection limit (65 pptv) throughout the campaign, lower than that measured in Baffin Bay and East Canadian Archipelago^33^ but consistent with model predictions of low concentrations in the region (<100 pptv)^33^ (Supplementary Text 4). Furthermore, NO_3_^−^ CIMS can measure amines at concentrations as low as a few parts per trillion by volume^34,35^, but none was detected here, indicating that the amine concentration is very low. Croft et al.^33^ reported that amine concentrations near Alert were below detection limits (~0.5 pptv). Neither H_2_SO_4_–DMA–H_2_O nor H_2_SO_4_–NH_3_–H_2_O alone is sufficient to explain the observed *J*_1.7_. Similarly, at our observed concentrations of HIO_3_, nucleation cannot be explained when considering HIO_3_ alone^36^ (Extended Data Fig. 5b).

Following He et al.^17^, we plotted *J*_1.7_ versus (HIO_3_ + H_2_SO_4_) × HIO_2_, representing the formation rate of H_2_SO_4_–HIO_2_ and HIO_3_–HIO_2_ (Fig. 3b). Among all systems that have been studied at the CLOUD chamber, the HIO_*x*_–H_2_SO_4_ system most consistently reproduces our *J*_1.7_ values, indicating the key role of both HIO_*x*_ and H_2_SO_4_ in our observed nucleation events. Considering the average ambient temperature of 1.4 °C during the nucleation periods, the central value of *J*_1.7_ on some days sits slightly above our interpolated fitting line based on the temperature dependence measured in CLOUD^17^ (Fig. 3b). This enhancement is probably driven by two factors: reduced cluster scavenging due to a lower condensation sink than in the CLOUD chamber (9 × 10^−4^ versus 2 × 10^−3^ s^−1^)^37^ and the potential participation of other stabilizing species. One plausible stabilizing species is DMA, as indicated by the occasional detection of DMA clustered with HIO_*x*_ (Supplementary Fig. 4 and Extended Data Figs. 3 and 4). The DMA–HIO_2_–HIO_3_ cluster signal correlates with HIO_3_ concentrations and is highest when HIO_3_ is at its maximum (Supplementary Figs. 4 and 5). Quantum chemical modelling studies showed that DMA can accept a proton from HIO_3_ as a base, forming a stable cluster (DMA–HIO_2_–HIO_3_)^38^. OOMs may also enhance nucleation rates^39^; however, we see no positive relationship between OOM concentrations (of any volatility class or source) and nucleation rates relative to He et al.^17^ (Supplementary Fig. 6). This indicates that the OOMs are unlikely to have a substantial effect on nucleation.

Our results demonstrate that H_2_SO_4_, HIO_3_ and HIO_2_ are the core components of a synergistic nucleation mechanism in our study area, which appears to be enhanced by bases such as DMA. This provides real atmospheric validation of the multicomponent nucleation mechanism by He et al.^17^ at lower, tropospheric vapour concentrations.

## Particle growth

**Extended Data Fig. 6:**
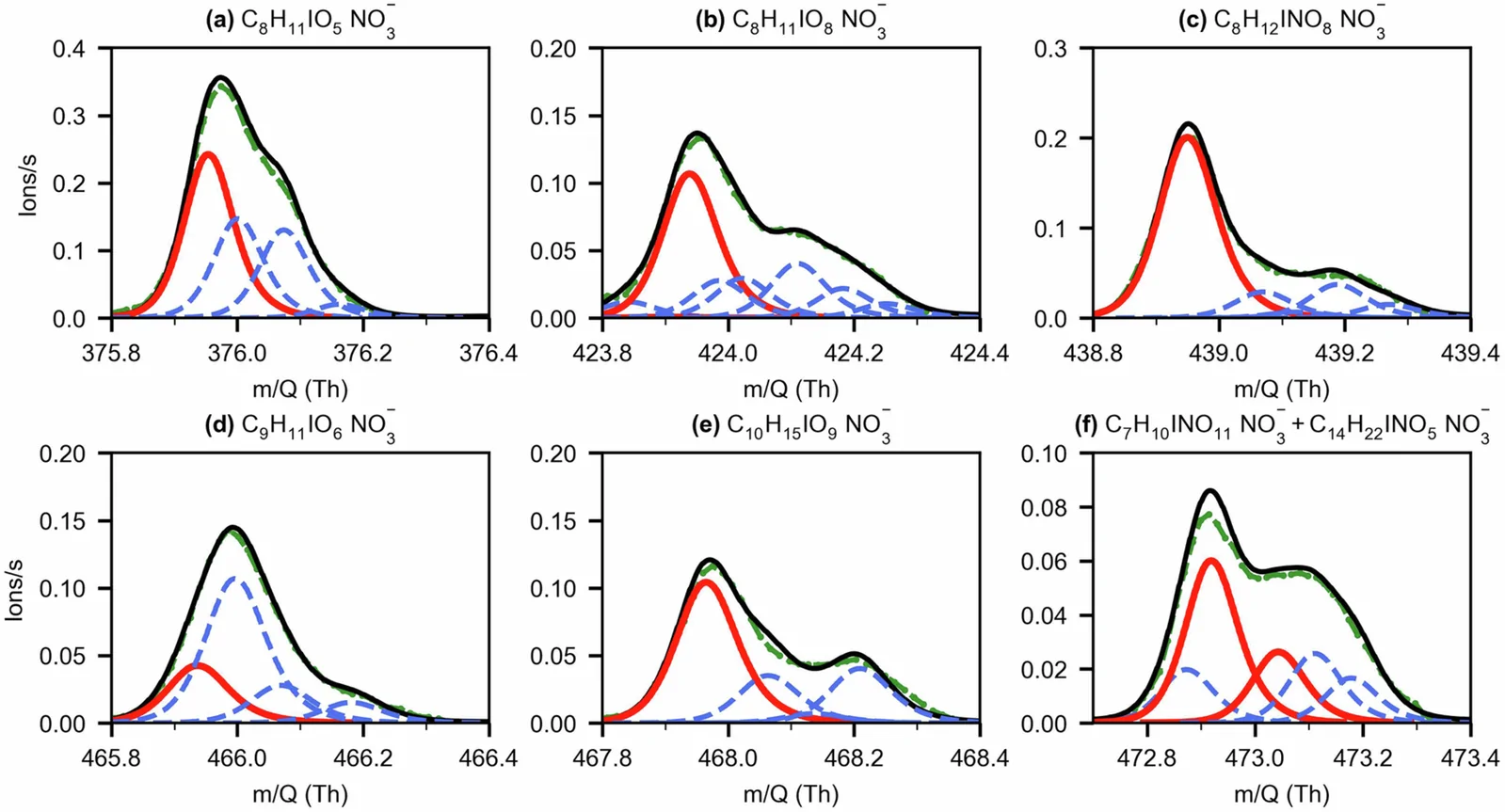
High-resolution peak fits for representative iodine-containing OOMs. High-resolution peak fits are shown for representative iodine-containing OOMs, including peaks at m/Q (**a**) 376, (**b**) 424, (**c**) 439, (**d**) 466, (**e**) 468, and (**f**) 473. Dashed blue lines represent fitted non-I-OOM peaks, red lines represent fitted I-OOMs, dashed green lines represent summed fits, and black lines represent raw data. I-OOMs were assigned when formulas containing only C, H, O, and N were insufficient, while chlorine and bromine were excluded based on isotope patterns. Source data

**Extended Data Fig. 7:**
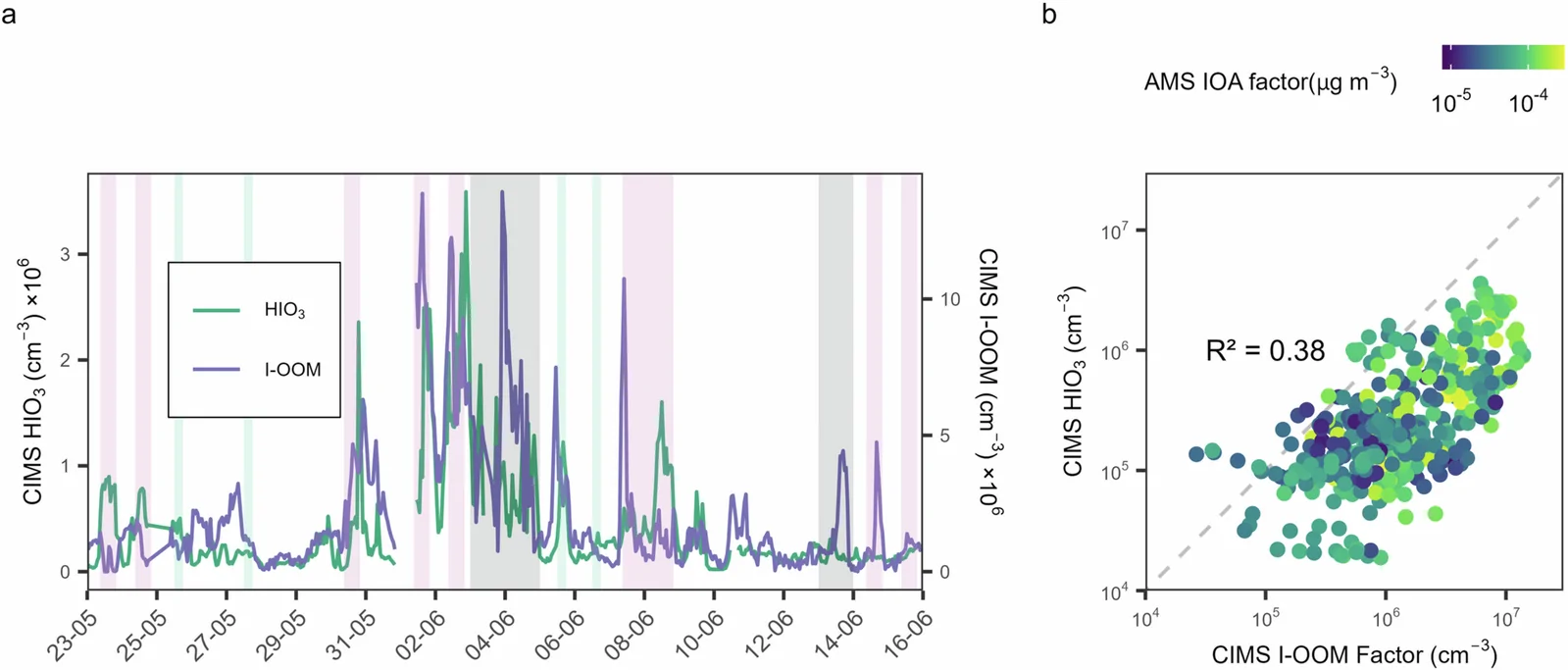
Iodine-containing species measured by NO_3_^−^ CIMS. Time series and correlation analyses are shown for iodine-containing species measured by NO_3_^−^ CIMS. Panel **a** shows HIO3 from NO_3_^−^ CIMS and the I-OOM PMF factor. Panel **b** shows the correlation between HIO3 and the I-OOM PMF factor, coloured by the IOA factor from AMS. The dashed line indicates a 1:1 ratio. Pink shaded periods indicate NPF event days, green shaded periods indicate burst event days, and gray shaded periods indicate periods affected by ship-stack contamination. Blank periods indicate missing data, such as instrument downtime. Source data

**Extended Data Fig. 8:**
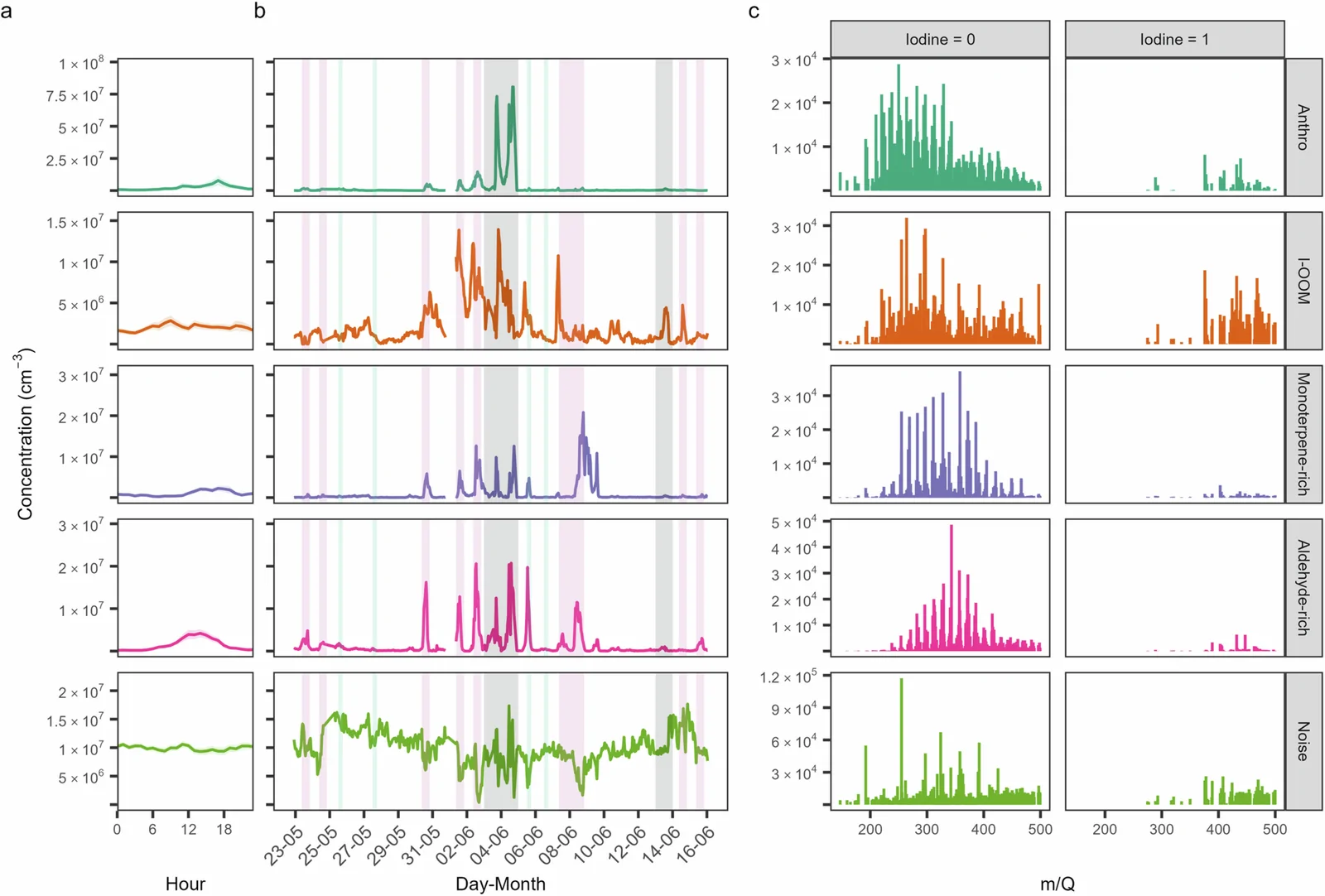
Sources of oxidized organic molecules from PMF analysis. PMF analysis of NO_3_^−^ CIMS data is used to identify sources of oxygenated organic molecules. Five factors are shown, ordered vertically, with (**a**) average diel profiles, (**b**) time series, and (**c**) mass spectra separated horizontally by the number of iodine molecules. Further methodological details are provided in Supplementary Methods 12. Pink shaded periods indicate NPF event days, green shaded periods indicate burst event days, and gray shaded periods indicate periods affected by ship-stack contamination. Blank periods indicate missing data, such as instrument downtime. Source data

We identified 591 gas-phase OOMs. In addition, 91 iodine-containing OOMs were detected, which we term as I-OOMs (see example peak fits in Extended Data Fig. 6; Supplementary Methods 3). These I-OOMs correlate well with HIO_3_ (Extended Data Fig. 7). Positive matrix factorisation (PMF) analysis of NO_3_^−^ CIMS data identified three distinct natural factors: aldehyde-rich, monoterpene-rich and I-OOMs-rich (Extended Data Fig. 8, Supplementary Figs. 7 and 8 and Supplementary Text 5). All three factors peak in the afternoon, although at different hours. The concentration of the I-OOM-rich factor represents, on average, ~10% of total OOMs, and we identified an iodine-organic aerosol (IOA) factor in the aerosol mass spectrometer (AMS) that increases in concentration as particles grow (Supplementary Methods 12 and Supplementary Fig. 9).

**Extended Data Fig. 9:**
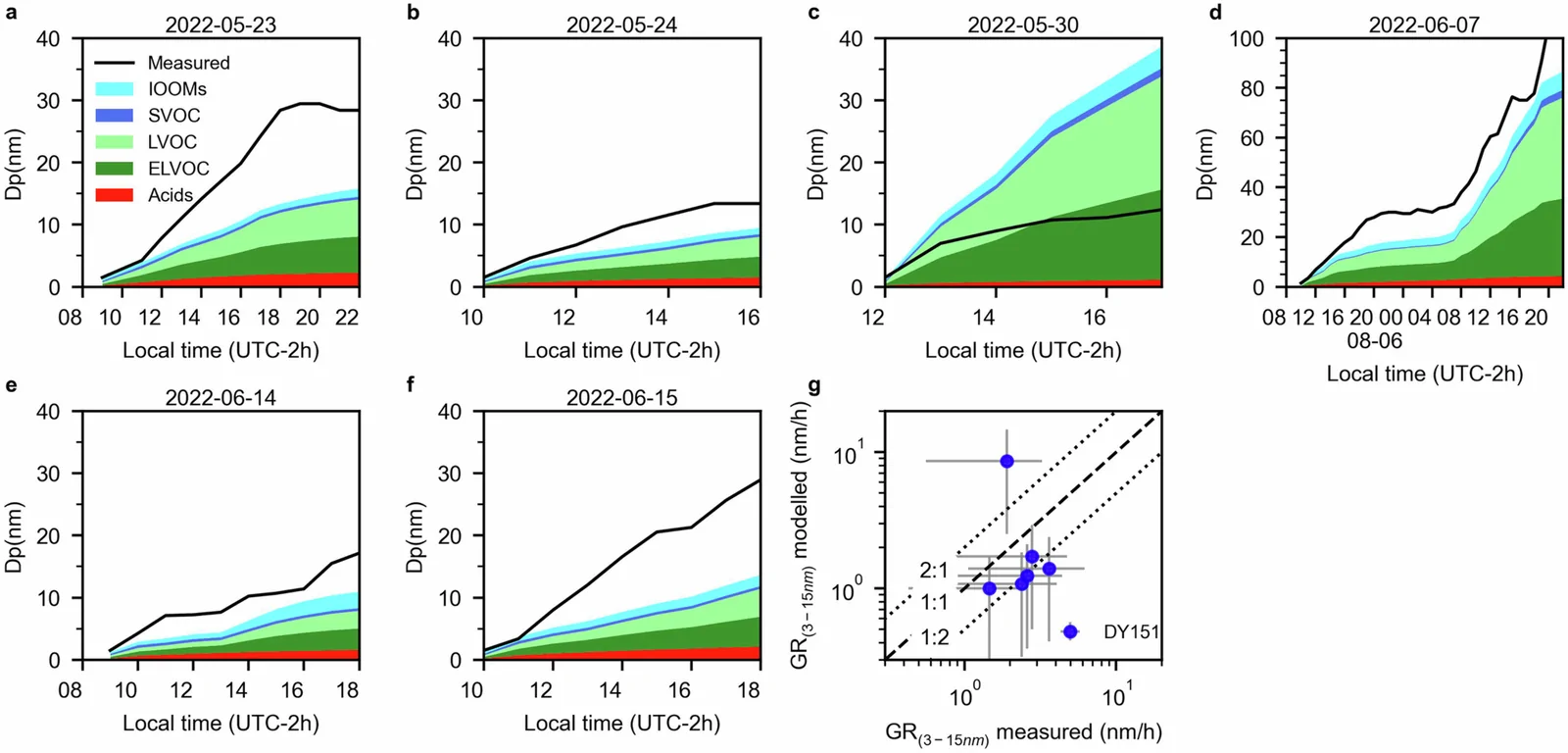
Particle growth modelling during NPF events. Simulated and measured particle growth are compared for NPF events. The model uses concentrations of acids, OOMs, and I-OOMs measured by NO_3_^−^ CIMS after subtracting the PMF noise factor. Contributions from H_2_SO_4_, MSA, HIO_3_, SVOCs, LVOCs, ELVOCs, and I-OOMs are shown in distinct colours, while the black line indicates measured modal diameter for individual days (panels **a** through **f**). In panel (**g**), modelled and measured 3–15 nm growth rates are compared with uncertainty represented by error bars. Source data

We observed six events with measurable particle GR. Particles formed during these NPF events consistently grew to 20 nm within a few hours. GRs for particles of diameters 3–15 nm varied between 1.5 nm h^−1^ and 3.6 nm h^−1^ (Supplementary Table 1), which are relatively high among those observed in long-term data in the Arctic (Supplementary Fig. 3), but akin to measurements made in the Canadian Arctic^40^. A dynamic aerosol growth model incorporating concentrations of gas-phase acids (H_2_SO_4_, MSA and HIO_3_) and OOMs^41^ (Supplementary Methods 11) shows that typically half or more of the observed GR were accounted for (Extended Data Fig. 9). The underprediction is probably due to the low sensitivity of NO_3_- CIMS in detecting semi-VOCs (SVOCs), multiphase reactions, coagulation growth and potential change in meteorological conditions (Supplementary Text 6).

The modelled particle GRs consistently show that OOMs, particularly low volatility (LVOC) and extremely low volatility (ELVOC) fractions, drive particle growth, with I-OOMs estimated to contribute 7–23% to modelled GRs (Extended Data Fig. 9). The fact that inorganic acids such as H_2_SO_4_ and HIO_*x*_ contribute little to particle growth and that this lower-limit OOM concentration can explain such a large fraction of the observed growth provides strong evidence that OOMs are indeed the dominant drivers of particle growth. On one occasion (30 May), there was an overprediction of growth, probably due to meteorological factors not being captured by the model.

Our findings provide conclusive evidence linking OOMs, including those containing iodine, to the growth of new particles in the Arctic.

## Growth to CCN

Throughout our campaign, we regularly observed CCN concentrations exceeding 500 cm^−3^ at 0.5% supersaturations (Extended Data Fig. 1). These elevated concentrations, coinciding with either NPF or the appearance of a mode of particles around 50 nm in size, were distinguishable from anthropogenic tracers (such as BC or NO_*x*_) or sea salt (Supplementary Figs. 2 and 9) and thus probably of secondary origin. New particle growth to CCN was directly observed during two events (23–24 May and 7–8 June).

**Fig. 4:**
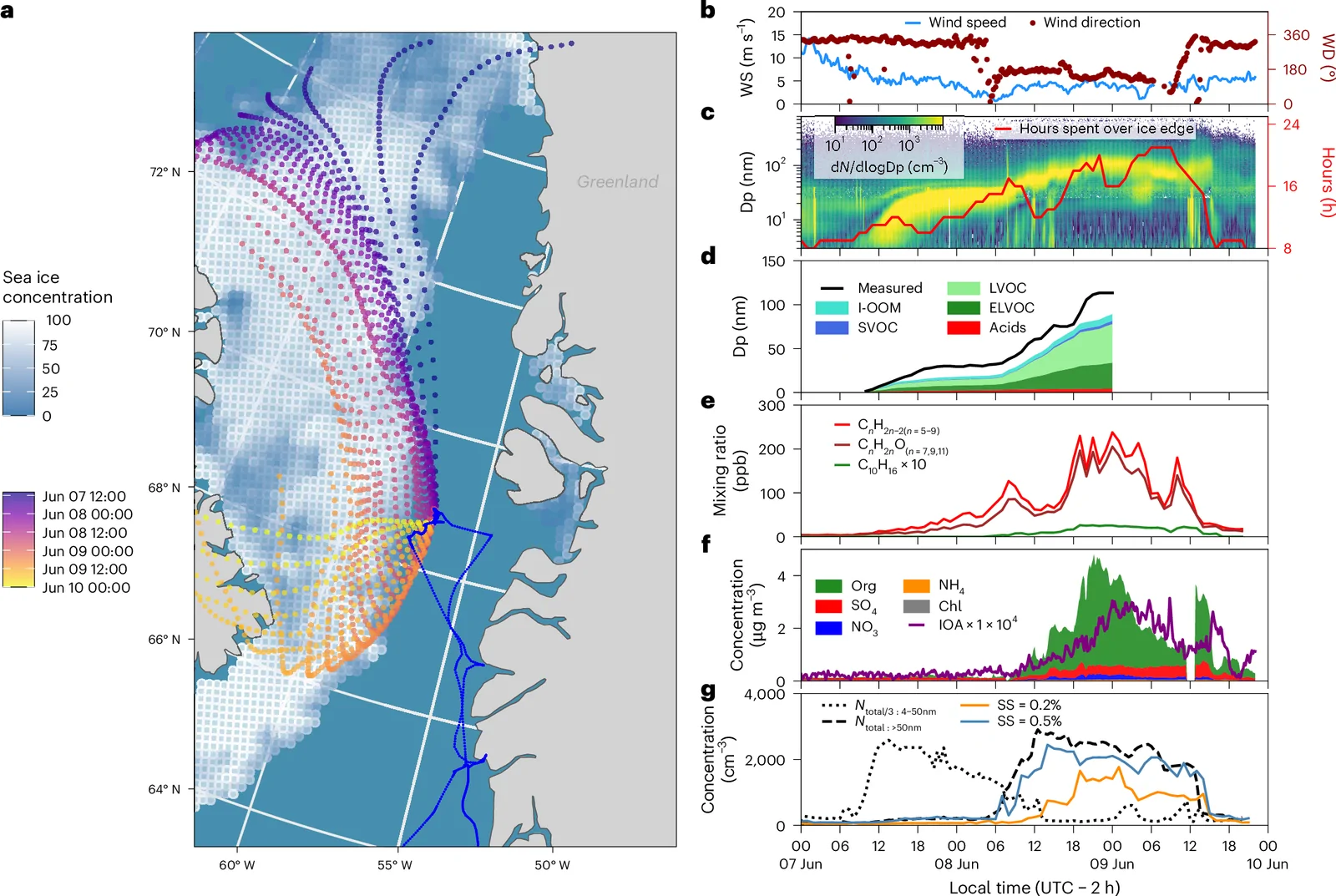
Dynamics of particle growth for NPF event at the marginal ice zone. **a**, Forty-eight-hour back-trajectories to the ship’s position on 7 to 9 June 2022. The blue line indicates our ship track. **b**, Wind speed (WS) and direction (WD). **c**, PNSD and time air masses spent over the sea ice edge. The sea ice edge is defined as the regions bordering open ocean with 1–30% sea ice concentration in each 25-km grid square. **d**, Measured and simulated particle growth on 7 and 8 June. The measured modal diameters are indicated by the black line. OOMs without iodine are separated into semi-volatile, low-volatility and extremely low-volatility organic compounds (SVOC, LVOC and ELVOC). Acids include HIO_3_, H_2_SO_4_ and MSA. The simulation ended when the particle diameter mode did not change or became smaller. **e**, Mixing ratios of VOCs and OVOCs measured via an online PTR-MS. **f**, Concentrations of organic and inorganic species measured by the AMS. The IOA factor, identified by the AMS PMF analysis, is multiplied by 10,000, due to low signals of iodine containing fragments (Supplementary Methods 12). **g**, CCN concentration at selected supersaturation (SS) levels, alongside the counts of particles under and over 50 nm in diameter. Source data

**Extended Data Fig. 10:**
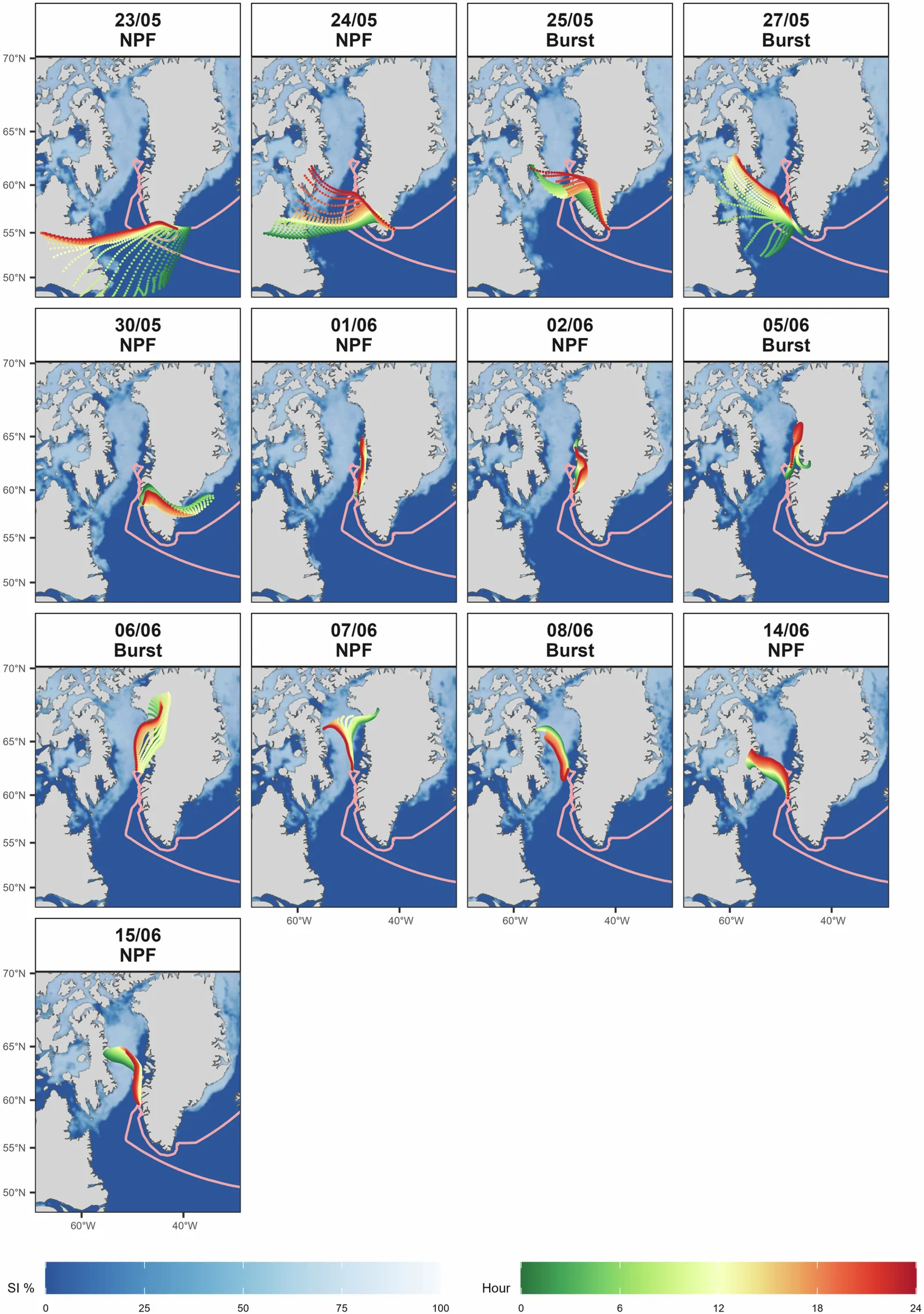
Forty-eight-hour air mass back trajectories. Forty-eight-hour air mass back trajectories were calculated using the NOAA HYSPLIT model for air masses arriving at the ship site. Trajectories were calculated hourly arriving at the ship position, with nucleation-event dates shown in each subplot. The pink line represents the cruise track during the campaign. Source data

The event on 7–8 June occurred as the air masses flowed over the marginal ice zone (the region with sea ice concentration between 10% and 80%), allowing us to examine NPF precursors and particle growth in an environment influenced by sea-ice-edge processes (Fig. 4a,c and Extended Data Fig. 10). More than 90% of these trajectories have a height <200 m. Over the 2 days, concentrations of VOCs increased to the highest levels observed during the campaign (Extended Data Fig. 1d and Supplementary Text 7). This is particularly prominent for aldehydes, as measured by proton transfer reaction mass spectrometry (PTR-MS; Fig. 4e). Aldehydes are common products of seawater ozonolysis^42^ and undergo fragmentation in the PTR-MS, producing ions such as C_5_H_8_H^+^ (Supplementary Text 7)^42–45^; therefore, we report the summed concentration of aldehyde-related signals.

The total concentration of C_7–12_ aldehydes and their fragments reached a peak of 420 pptv during this NPF event, although these fragments may have non-aldehyde sources, and this is therefore an upper-limit estimate of the total concentration. Off-line analysis using two-dimensional gas chromatography mass spectrometry (Supplementary Fig. 8) confirmed the presence of aldehydes, which are dominated by C_9_, C_12_ and C_13_. While the aldehyde-to-monoterpene ratios are high (up to 140 v/v; Fig. 4e), PMF analysis of the CIMS data (Extended Data Fig. 8 and Supplementary Fig. 7) indicates that both aldehyde- and monoterpene-derived OOMs were highly abundant during this period.

On 7 June, OOMs drove growth of new particles to ~30 nm, followed by further growth to ~100 nm on 8 June (Fig. 4c), when the air mass flowed along the ice edge for up to 24 h, sampling high VOC emissions. As the new particles grew, condensation of more volatile OOMs became increasingly important, enhancing GRs to ~5 nm h^−1^. Once the particles surpassed 50 nm, they became measurable by the AMS, which showed that organic matter dominated the particle mass, with a concurrent but smaller increase in sulfate mass. This faster growth at larger sizes is consistent with the phenomenon observed by Burkart et al.^22^ in the Canadian Arctic Archipelago, which they hypothesized was driven by SVOCs. Our chemical observations confirm that, as particles get larger, LVOCs contribute more to growth than ELVOCs (Fig. 4d). The IOA factor in the AMS data increased as particles grew larger (Fig. 4f and Supplementary Fig. 9), further confirming the contribution of I-OOMs to particle growth. Concurrently with the increase in organic and sulfate mass, CCN counts increase from approximately 50 to 1,500 cm^−3^ (0.2% supersatuation, SS) or 100 to 2,500 cm^−3^ (0.5% SS), corresponding with an increasing number concentration of >50-nm particles (Fig. 4g). These CCN counts are higher than those reported in previous Arctic campaigns, such as NETCARE^7,24,46^. Note that, even when no CCN enhancement was directly observed following nucleation events, newly formed particles may still contribute to the CCN budget downwind as they grow larger^24^. Similarly, some of our observed background CCN concentrations may themselves come from NPF processes^11,47^.

Our findings provide direct observational evidence that organic compounds, including previously unrecognized classes such as OOMs derived from aldehydes and iodine-containing OOMs, contribute to NPF in the Arctic and drive the growth of the resulting particles to CCN (see also Supplementary Text 1).

## Sources, processes and atmospheric implications

**Fig. 5:**
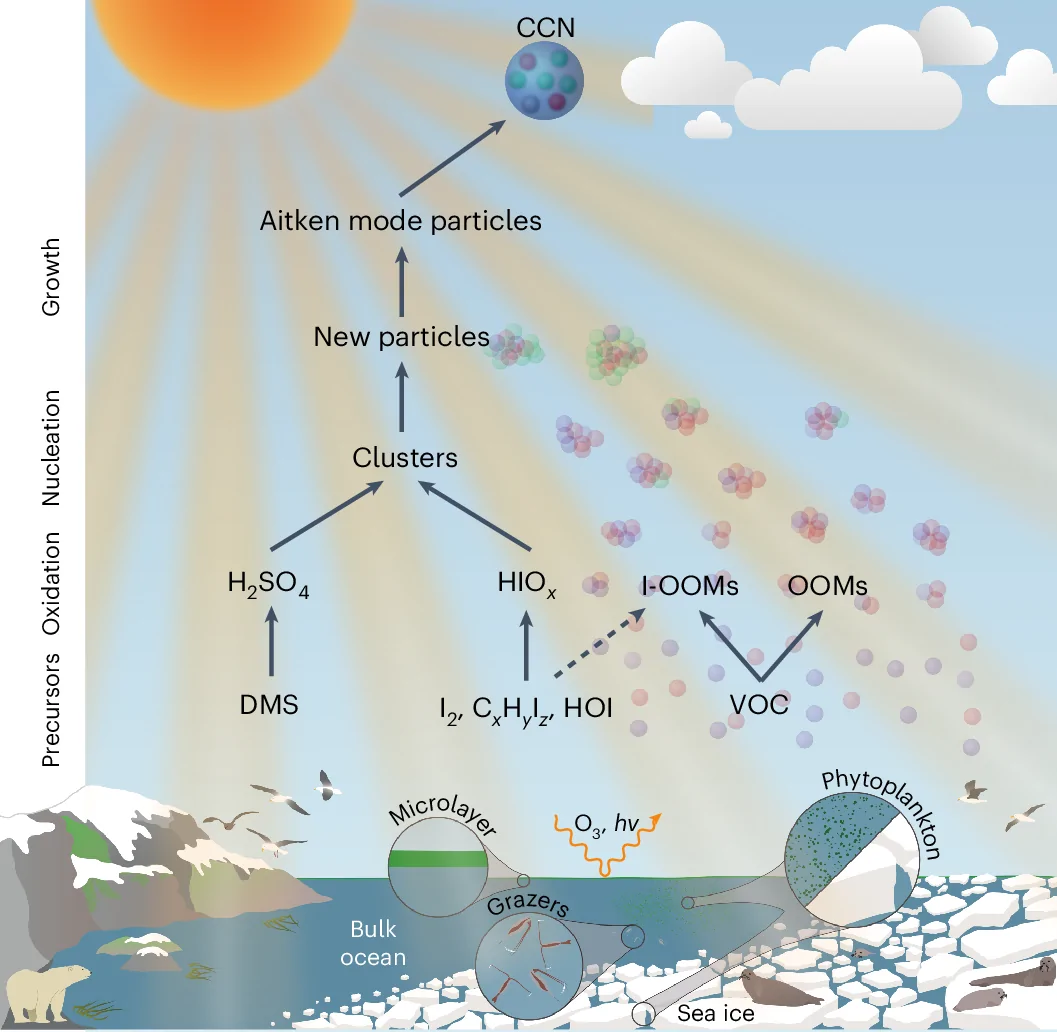
Schematic representation of natural nucleation and growth of new particles to CCN in the Arctic marine boundary layer. Volatile gases are emitted from diverse sources including sea ice, open ocean, coastal systems and bird colonies via both biotic and abiotic processes. They then undergo oxidation to produce NPF precursors such as HIO_3_ (ref. ^63^), HIO_2_ (ref. ^17^) and H_2_SO_4_, driving particle formation, and OOMs^51^ and I-OOMs, which are the primary drivers of particle growth.

Our observations demonstrate that HIO_*x*_ and H_2_SO_4_ synergistically drive nucleation. Subsequent particle growth to CCN is propelled by a range of OOMs, including I-OOMs. We observed nucleation on over 80% of days when peak solar radiation exceeded 600 W m^−2^, suggesting that this process is widespread across the region, which could have been missed without sub-5-nm particle observations. The precursor vapours emerge from diverse natural sources (Fig. 5), including open ocean, ice, marginal ice zone and coast.

H_2_SO_4_ and HIO_*x*_ primarily originate from the oxidation of DMS and iodine-containing species (for example, I_2_, C_*x*_H_*y*_I_*z*_ and HOI), respectively. Elevated concentrations of both species were observed near the marginal ice zone, particularly when air masses were exposed to the ice edge for extended periods, identifying this region as a key hotspot. This enhancement is probably driven by combined emissions from open leads, intensified biological activity and active photochemistry^48,49^. Consistent with this, thriving under-ice algae and elevated oceanic chlorophyll *a* were observed (Extended Data Fig. 1g and Supplementary Fig. 10), which are indicators of sources of DMS, and therefore H_2_SO_4_ and MSA^11,26,50^ (Supplementary Fig. 11).

The highest HIO_*x*_ concentrations occurred in coastal Greenland, including fjords, indicating strong local iodine sources. In addition to the marginal ice zone, the open ocean represents a broader source of both DMS and iodine-containing species^25,26^, although emission fluxes in this study region remain unconstrained. Iodine emissions may also arise from ice and snow surfaces through photochemical recycling of deposited iodine^48,49^.

Previous Arctic studies have identified H_2_SO_4_–NH_3_ and HIO_*x*_ nucleation as key processes^13,14^. HIO_*x*_, H_2_SO_4_ and sometimes mixed iodine–sulfur clusters were also detected^13,14^. This suggests that the HIO_*x*_–H_2_SO_4_ nucleation process may have been present in their observations, although they lacked the opportunity to identify its potential importance. The relative importance of different nucleation mechanisms in the wider Arctic probably depends on season and location^14^ (Supplementary Fig. 1), which control emission and oxidation processes, and thus the concentration of precursor gases^51^ (such as DMS and I_2_; Fig. 5). To better quantify nucleation mechanisms across the Arctic, comprehensive observations at multiple platforms and locations are needed.

The OOMs and I-OOMs that drive growth of new particles are formed from oxidation of VOCs and OVOCs that were observed at highest concentrations when air masses flowed over the ice edge. Aldehyde-OOMs originate from aldehydes, which are formed by oxidation of fatty acids in the sea-surface microlayer^42,52^. Monoterpenes are emitted from the ocean through diffusion, although at lower rates than from most land regions^53,54^. Both aldehydes and monoterpenes readily undergo autoxidation^51,55^ to form the OOMs required for particle growth. We propose that I-OOMs form through regular autoxidation, with iodine-containing radicals serving as autooxidation terminators (Supplementary Text 5). The strong correlations between HIO_3_ and I-OOMs (Supplementary Fig. 5) indicate that they share similar sources.

Like nucleation precursors, the growth of new particles is dependent on season and location, which dictate the availability of VOCs and OVOCs and, thus, OOMs. The high Arctic, including the central pack-ice region, has high abundance of HIO_3_ (ref. ^14^). Conversely, VOC and, thus, OOM concentrations appear to be low (Supplementary Text 1), leading to limited new particle growth and CCN formation^7,10,13,14^. Meanwhile, OOMs seem to be more abundant over the Arctic and sub-Arctic seas and marginal ice zone, resulting in faster growth of new particles (Fig. 4 and Extended Data Fig. 9), and contribute to CCN formation (Fig. 5).

In the Arctic atmosphere west of Greenland, synergistic interaction between sulfur, iodine and organic compounds drives efficient particle and CCN formation (Fig. 5), demonstrating that ocean emissions of volatile gases can promote CCN formation in a pristine marine environment. The fundamental process underpinning this link is consistent with a key segment of the CLAW hypothesis^56^, although the link we found here is more complex given the involvement of not just oceanic sulfur species but also iodine and organic compounds.

CCN formation from these natural precursors has the potential to influence Arctic cloud droplet number and reflectivity, particularly important in the pristine Arctic atmosphere, where clouds are often sensitive to CCN perturbations^3,5,24^. While conditions considered to be strongly CCN-limited (for example, CCN <10 cm^−3^) occur periodically across the wider Arctic in May to June (Supplementary Fig. 12), background concentrations in our study region remained above this threshold (Extended Data Fig. 1). The order-of-magnitude CCN increases we observed during NPF represent a substantial perturbation to the regional aerosol population with a complex net radiative effect, potentially leading to surface warming over high-albedo snow and sea ice or a cooling effect over the open ocean^56,57^.

Accelerating Arctic warming increases sea ice and glacier melting, leading to more frequent under-ice phytoplankton blooms^50^, enhanced river runoff^27^ and enhanced ocean productivity^27^, potentially increasing emissions of iodine, sulfur and VOCs, and therefore NPF event frequency^12^. Warming will also drive marginal ice zones northwards and widen them; while their total area has remained stable^58–60^, models project they will dominate the summer Arctic in future^61,62^, extending marginal ice zone-related NPF to higher latitudes with potential impacts on ice melting and thereby regional and global climate.

Considering that sulfur, organic and iodine sources are widespread globally, the mechanisms we showed here are probably relevant beyond the Arctic (Supplementary Fig. 13). A key obstacle is representing this chemical complexity in global models: incorporating synergistic H_2_SO_4_–HIO_*x*_ and key stabilizing organic and inorganic species, supported by laboratory kinetic studies, would better represent observed NPF. This moves beyond treating these as separate pathways and reflects the synergistic mechanism now observed in both the laboratory and the field. Such updates must be underpinned by improved emission inventories, especially in the rapidly transforming Arctic, where marine iodine and DMS emissions are increasing^26^ alongside anthropogenic perturbations such as SO_2_. Furthermore, progress will require coordinated efforts combining intensive and long-term field measurements at key locations—capturing the full NPF sequence from precursor emissions through nucleation, growth and CCN formation—with laboratory studies designed to reproduce real-world chemical complexity, including those investigating aldehyde-derived OOM and iodine-organic chemistry. Such integrated approaches are essential for understanding the temporal and spatial variability of these processes and quantifying their implications for clouds and climate.

## Methods

### Campaign description and physical aerosol measurements

Measurements were conducted aboard the RRS *Discovery* during the DY151 cruise. The data presented here focus on the pristine Arctic marine boundary layer and marginal sea ice zone between 23 May and 16 June 2022 (Supplementary Methods 1). Continuous PNSDs from 3 to 736 nm were measured by combining data from a Neutral cluster and Air Ion Spectrometer (NAIS, Airel Ltd) and two mobility particle size spectrometers (nano-MPSS and long-MPSS, TSI; Supplementary Methods 2).

### Precursor gas and cluster characterization

Neutral OOMs, acids (H_2_SO_4_, MSA, HIO_3_ and HIO_2_) and their molecular clusters were quantified at high temporal resolution using NO_3_^−^ CIMS (Aerodyne; Supplementary Methods 3). Peak fitting allowed the identification of multicomponent clusters and a newly detected class of I-OOMs. VOCs, including OVOCs such as aldehydes, were monitored using a PTR-MS (Ionicon), complemented by 24-h integrated speciation via two-dimensional gas chromatography mass spectrometry (Supplementary Methods 4).

### Environmental parameters, trace gases and aerosol composition

To identify and exclude periods contaminated by ship stack emissions or anthropogenic transport, we continuously monitored background trace gases (O_3_, NO_*x*_, CO, SO_2_ and NH_3_; Supplementary Methods 5) and BC using a single-particle soot photometer (SP2-XR, DMT; Supplementary Methods 6). Bulk aerosol chemical composition including organics, sulfate, nitrate, ammonium and chloride was determined using a high-resolution time-of-flight AMS (Aerodyne; Supplementary Methods 7). CCN number concentrations were measured using a CCN-100 Counter (DMT) at supersaturations ranging from 0.1% to 0.7% (Supplementary Methods 8). Furthermore, surface seawater chlorophyll-*a* concentrations were continuously monitored using underway absorption spectrophotometry (Supplementary Methods 9).

### Data analysis and modelling

Observation days were classified into three categories: NPF events (formation of 3–5-nm particles with continuous growth beyond 20 nm); burst events (transient increases in sub-10-nm particles without sustained growth); and non-events (Supplementary Methods 10). *J* and GR were derived from the harmonized PNSD data. Because sub-2-nm distributions were not directly measured, *J*_1.7_ was extrapolated from the 4-nm formation rate using a survival probability framework. See Supplementary Methods 11 for the full derivations, coagulation sink calculations and associated error propagations.

To quantify the drivers of particle growth, we employed a dynamic aerosol growth model. OOMs identified by the NO_3_^−^ CIMS were categorized by volatility (SVOC, LVOC and ELVOC) based on their elemental composition (carbon, oxygen, nitrogen and iodine content). The model simulated particle GR via the condensation of these OOMs alongside inorganic acids (H_2_SO_4_, MSA and HIO_3_), accounting for mass-dependent transmission efficiencies and transition-regime diffusion kinetics (detailed in Supplementary Methods 11).

PMF was utilized to deconvolve sources of both gas-phase NPF precursors and aerosol particles (Supplementary Methods 12). PMF was applied to the NO_3_^−^ CIMS gas-phase time series to separate distinct OOM sources (for example, aldehyde-rich, monoterpene-rich and I-OOM factors). Similarly, PMF was applied to the high-resolution time-of-flight AMS in two separate PMF models: one to deconvolve the organic mass spectra, and one incorporating specific sea salt and iodine fragment ions to isolate the IOA factor from primary sea salt.

## Online content

Any methods, additional references, Nature Portfolio reporting summaries, source data, extended data, supplementary information, acknowledgements, peer review information; details of author contributions and competing interests; and statements of data and code availability are available at https://doi.org/10.1038/s41561-026-02062-6.

## Supplementary information

Supplementary Information Supplementary Methods, Text 1–7, Figs. 1–14, Tables 1–5 and References.

Peer Review File

## Source data

Source Data Fig. 1 Statistical source data.

Source Data Fig. 2 Statistical source data.

Source Data Fig. 3 Statistical source data.

Source Data Fig. 4 Statistical source data.

Source Data Extended Data Fig. 1 Statistical source data.

Source Data Extended Data Fig. 2 Statistical source data.

Source Data Extended Data Fig. 3 Statistical source data.

Source Data Extended Data Fig. 4 Statistical source data.

Source Data Extended Data Fig. 5 Statistical source data.

Source Data Extended Data Fig 6 Statistical source data.

Source Data Extended Data Fig 7 Statistical source data.

Source Data Extended Data Fig 8 Statistical source data.

Source Data Extended Data Fig 9 Statistical source data.

Source Data Extended Data Fig 10 Statistical source data.

## Data Availability

Data for Figs. 1–5, Extended Data Figs. 1–10 and Supplementary Figs. 1–14 are available via Zenodo at https://doi.org/10.5281/zenodo.13253811 (ref. ^64^). Source data are provided with this paper.
