# Supplementary material for: Arctic cloud condensation nuclei enhanced by iodine, sulfur and organic precursors
Source: Nat Geosci. 2026 Aug 5;19(9):1039–46. doi: 10.1038/s41561-026-02062-6 (PMC13541622; doi:10.1038/s41561-026-02062-6)
Supplement: Supplementary file 1 [file 41561_2026_2062_MOESM1_ESM.pdf]

nature geoscience

Supplementary information https://doi.org/10.1038/s41561-026-02062-6

Arctic cloud condensation nuclei enhanced by iodine, sulfur and organic precursors

In the format provided by the authors and unedited

Contents

Supplementary Methods 3
1. DY151 campaign description 3
2. Aerosol properties – particle number size distribution (PNSD) 4
Neutral cluster and Air Ion Spectrometer (NAIS) 4
Scanning mobility particle sizers (SMPSs) 4
3. Molecule and cluster composition 6
Nitrate chemical ionization mass spectrometry (NO_3^- CIMS) 6
Determination of acid and oxygenated organic molecule concentrations 10
OOM volatility estimation 13
4. Volatile organic compound (VOC) measurements 14
PTR-MS 14
2D-GC-MS 15
5. Trace gases (O_3, NO_x, CO, SO_2 and NH_3) measurements 16
O_3, NO_x and CO analyzers 16
Laser-induced fluorescence (LIF) 17
6. Single Particle Soot Photometer-Extended Range (SP2-XR) 18
7. Aerosol composition: High-resolution time-of-flight Aerosol Mass Spectrometer (HR-ToF-AMS) 18
8. Cloud Condensation Nuclei (CCN) concentration: CCN-100 Counter 19
9. Chlorophyll-*a* (Chl-*a*) measurement from Absorption spectrophotometry 19
10. Classification of nucleation events 20
11. Determination of particle formation and growth rates 21
Formation rate 21
Growth rate (GR) calculation 24
Dynamic aerosol growth modelling: organic vapours 24
Dynamic aerosol growth modelling: acids 26
12. Source apportionment of gas-phase OOMs and aerosol particles: Positive Matrix Factorization (PMF) 27
Gas-phase OOMs 27

Supplementary Text 31
1. Advancing the Understanding of Arctic NPF: Context and Future Directions 31
1.1 Nucleation 31
1.2 Growth of new particles 32
1.3 Molecular understanding from nucleation and growth to CCN 34
1.4 Remaining gaps and future work on molecular understanding from nucleation and growth to CCN in polar regions 35
2. Identification of contamination from ship's plume and calculation of background tracer concentrations 35
3. Limit of detection for NO_3^- CIMS and influence of ambient ions 37
4. Insignificant role of ammonia and HNO_3 in nucleation 39
5. Interpretation of PMF factor for OOMs 41
6. Uncertainties of particle growth modelling 45
7. VOC composition 46

Supplementary Methods

1. DY151 campaign description

The DY151 cruise on the *Royal Research Ship (RRS) Discovery* started on 19th May and ended on 26th June 2022. The expedition departed Reykjavík, Iceland, and sailed across the North Atlantic Ocean, southeast Greenland, to the Labrador Sea and then Davis Strait (next to melting sea ice) before returning to Southampton, UK (Figure 1a). Here, we present the data collected from 23rd May to 16th June. Outside of these dates, the ship was on passage. An interdisciplinary team of scientists from various institutions utilized a suite of instruments to measure trace gas concentrations, the physical and chemical properties of aerosol particles and chlorophyll-*a*. A key objective was to investigate molecular mechanisms of new particle formation (NPF) and their contribution to Cloud Condensation Nuclei (CCN) in these regions.

Most instruments were housed in containers on the front deck of the cruise. The inlets for the particle number size distribution (PNSD, see below) and nitrate chemical ionization mass spectrometry (NO_3^- CIMS) faced out of the side of the container to keep inlet lengths as short as possible. The inlet for the Proton Transfer Reaction-Quadrupole interface Time-Of-Flight Mass Spectrometer (PTR-MS) was positioned on the front mast. All other gas and aerosol measurements were taken using inlets coming from the top of the container. Meteorological parameters such as temperature, relative humidity, wind speed and wind direction, and solar radiation were measured on the meteorological platform at the front mast of the ship. The core instruments utilized in this study are detailed in the following section. The cruise track, alongside the location of each nucleation event is illustrated in Figure 1a.

2. Aerosol properties – particle number size distribution (PNSD)

We measured the PNSD using three mobility particle size spectrometers (MPSS)

Neutral cluster and Air Ion Spectrometer (NAIS)

The Neutral cluster and Air Ion Spectrometer (NAIS, Airel Ltd. Estonia) is an MPSS used to measure the PNSD of naturally charged and neutral particles. NAIS is an ion mobility spectrometer that covers a mobility range of 3.2 to 0.0014 $cm^2\ V^{-1}\ s^{-1}$, corresponding to particle diameters from 0.8 to 42 nm [1,2]. It has two parallel multichannel electrical mobility analyzer columns that allow simultaneous detection of positive and negative ions and neutral particles with a maximum time resolution of 1 second. The NAIS operates in three modes: ion mode, particle mode, and offset mode. In ion mode, it measures the naturally charged particles. In particle mode, it charges the neutral particles with a corona charger and measures the total particle number concentration. This is the sum of neutral and charged particles. In offset mode, it measures the electrometer noise levels. The distribution of corona ions has been measured with a diameter in the range of 1-2 nm [3]. The ions in these ranges are discarded in our data analysis as they are generated by the charger and do not reflect the ambient aerosol population. The NAIS was run at its standard high sample flow rate of 54 L min^{-1} and sheath flow rate of 60 L min^{-1}.

Scanning mobility particle sizers (SMPSs)

Two scanning mobility particle size spectrometers (SMPSs, nano-SMPS and long-SMPS, TSI Inc, USA, hereon referred to as the nano and long MPSSs) were employed to measure the number size distribution of aerosol particles. The nano-MPSS, comprising a nano-differential mobility analyser (nano-DMA) and a condensation particle counter (CPC) 3776 (TSI Inc, USA), was used to measure the PNSD from 4.5 nm to 65 nm in mobility diameter. The long-MPSS,

consisting of a long-DMA and a CPC 3775 (TSI Inc, USA), covers the PNSD from 14 nm to 736 nm. Both instruments were operated with a time resolution of 5 minutes. The sample flow rates were set to 1.5 L min^{-1} for nano-MPSS and 0.3 L min^{-1} for long-MPSS. A sheath-to-sample flow rate ratio of 10 was applied to ensure the laminar flow of the samples. To neutralize the electrostatic charges on aerosol particles, X-ray neutralizers were utilized on the two MPSSs. The MPSS data were subsequently corrected for single charging efficiency, multiple charges, and diffusion losses to ensure accurate results using the aerosol instrument manager (AIM) software (TSI Inc, USA).

Utilizing the data from the three MPSS systems, we produced a conjoined PNSD for the entire campaign utilizing the NAIS from 3 to 25 nm, the nano-MPSS from 25 to 40 nm, and the long-MPSS from 40 to 736 nm. To harmonise the PNSDs across the instruments, the NAIS and Nano-SMPS data were scaled to match the Long-SMPS measurements within their respective overlapping size regions. As the NAIS and Nano-SMPS routinely over-report particle counts, we applied constant reduction factors of 1.20 and 1.38, respectively, across the entire campaign to align them with the Long-SMPS. The stability of these static scaling factors was validated using reference condensation particle counter data from deployments immediately preceding and following this cruise. We estimate a 30% relative uncertainty in our quantification of particle number related to both uncertainty in measurement, and uncertainty associated with harmonizing measurements from these different instruments.

3. Molecule and cluster composition

Nitrate chemical ionization mass spectrometry (NO_3^- CIMS)

The NO_3^- CIMS (Aerodyne Research Inc, USA) was utilized to measure neutral oxygenated organic compounds (OOMs), acids, and their molecular clusters with high temporal resolution and resolving power [4-6]. The instrument's front end consists of a chemical ionization system that draws a sample flow of 10 L min^{-1} through a 1 m long, 1.9 cm outer diameter stainless steel tube to the ion drift tube. In parallel, nitric acid vapour is volatilized from a glass tube containing liquid HNO_3 and carried by a mixture of 3 cm^3 min^{-1} N_2 and 20 L min^{-1} pristine Arctic ambient air as a sheath gas. The nitric acid vapour is then exposed to soft X-rays to produce primary ions $(HNO_3)_{0-2}NO_3^-$. The introduced sample air is concentrically surrounded by sheath gas. An electrostatic field is employed to direct the reagent ions towards the center of the sample flow, facilitating their interaction with the sampled compounds. The mixed flows enter the critical orifice at the front-end of the instrument and are guided through several differentially pumped chambers before reaching the ToF analyser. Two of these chambers contain quadrupoles (Small-Segmented Quadrupole and Big-Segmented Quadrupole) that focus the ion beam and enhance sensitivity for certain mass ranges.

Data analysis was conducted using the Tofware package (Tofwerk AG) and Igor Pro 9.0.4 (Wavemetrics Inc.). The mass spectrometer's resolution was 3700 at m/Q 201. Peak fitting was performed following the procedure outlined in Tofware, after which molecular formulas were assigned. The fitting process is initiated by defining a mass calibration, before the instrumental peak shape and width is established from well-isolated peaks across the mass spectrum. Following this, mass calibration is re-applied and peaks are fit. For spectrally complex regions

with overlapping signals, a multi-peak fitting procedure was employed [7], which always accounted for almost all of the signal at this mass (see peak fits in Extended Data Figs. 3 & 6, Figs. S4,), after which molecular formulas were assigned. As peaks were assigned, the mass-calibration was refined, ultimately utilising NO_3^-, $HNO_3NO_3^-$, HSO_4^-, $C_6H_5NO_3NO_3^-$, and $C_{10}H_4F_{12}ONO_3^-$. $C_{10}H_4F_{12}ONO_3^-$ is a contaminant likely from the tube walls. Fluctuations in mass calibration resulted in no more than a 5% variation in the total fitted intensity for a representative OOM. The mass calibration and precision of peak separation follow Stark et al. [7] and Cubison et al. [8], respectively.

Our assigned OOM formulae avoided hypervalent molecules, molecules with H:C ratios below 0.5, or O:C ratios exceeding 2, which is the upper limit observed in laboratory studies. Molecules that would not be detected by NO_3^- CIMS (less than four oxygen atoms), as well as deprotonated OOMs (as opposed to clusters with NO_3^-), were deprioritised. Due to the increasing difficulty in confidently assigning molecular formulas at higher masses with our instrumental resolution, we did not assign peaks beyond an m/z of ~550. Iodine-OOMs were assigned when no reasonable non-iodine containing molecular formulas matched.

In assigning I-OOMs, we have considered several potential contributors. Firstly, Bin-PMF [9] was utilised to identify distinct factors within the data. This analysis revealed a factor containing several large peaks with no diurnal cycle, including species with negative mass defects such as a prominent peak at 362 Th (e.g., $C_5F_{10}H_2O_3$ NO_3^-). The factor lacked the homologous series characteristic of OOMs (e.g., $C_{10}H_{16}O_{2-8}$), indicating that these species were likely fluorine-containing contaminants rather than atmospheric OOMs. A separate factor containing I-OOMs was identified, exhibiting a distinct time series and correlating with HIO_3. Many of the peaks in this second factor could not be assigned using only C, H, O, N, and F, supporting the presence of

iodine-containing species. Next, the possibility of sulfur-containing compounds was assessed. Given the exact mass of sulfur-32 (32.97) and its mass defect (~0.3), it was determined that sulfur incorporation would require 2–3 atoms per molecule, or more, to match the observed exact masses. For instance, the peak at 376 Th would require a composition such as $C_7H_{10}N_2O_6S_3$ NO_3^-, which is chemically implausible. No known gas-phase VOCs contain multiple sulfur atoms; we measured no such VOCs in our PTR-MS and there are no established oxidation pathways that could account for such compositions. Sulfur was considered an unlikely contributor. Correlation with HIO_3 and reasonable compositions derived from iodine chemistry further supported iodine as a more likely component of these OOMs.

Furthermore, to assess the potential involvement of chlorine (Cl) and bromine (Br), all I-OOMs offset by 2 Th were examined. Each case was evaluated against three criteria: 1) whether the peaks shared the same mass defect, 2) whether 1–2 Cl or Br atoms could explain the relative intensities, and 3) whether they exhibited the same time series. No peaks satisfied all three criteria, ruling out Cl and Br as likely contributors.

A table of likely clusters observable in the NO_3^- CIMS was constructed based on chamber measurements [10,11] and other field studies [12-14]. For combinations of compounds not experimentally studied, clusters with the formulas $A_{1,2,3}B_{1,2,3}(HNO_3)_{0\text{-}2}(NO_3)_{0\text{-}1}$, where A represents acids (H_2SO_4, MSA, HIO_3) and B represents possible bases (HIO_2, NH_3, dimethylamine, and trimethylamine), were searched. Following the OOM peak fitting and assignment, peaks with missing intensity at m/Q corresponding to these clusters were identified. If a cluster appeared during NPF periods but not before NPF, the cluster was assigned and included in Figure 2. Uncertainties in mass calibration were consistently less than 15 ppm,

ensuring the robustness of peak assignments. Formulas were assigned within 15 ppm of the fitted peaks.

$C_2H_7N^-HIO_2^-IO_3^-$ peak has been identified, i.e., when the adjacent interfering peak ($C_7H_{11}IO_6NO_3^-$) is relatively small (Fig. S4). Considering that HIO_3 and HIO_2 (which is correlated with HIO_3, Fig. 3a) are key components of this cluster, its identification is further supported by (1) $C_2H_7N^-HIO_2^-IO_3^-$ and IO_3^- raw signals are well correlated (R^2=0.74); and (2) the time series of the two also matched well (Fig. S5). Furthermore, we estimated that the uncertainty in quantification of the $C_2H_7N^-HIO_2^-IO_3^-$ peak due to our m/Q calibration of 15 ppm is ~7%, and the estimated precision on fitted intensity is 3%. The proximity to the closest peak is 1.1 peak half-widths. We further carried out sensitivity analysis of the entire time series to mass calibration. The results show that the time series is highly correlated with one that was perturbed by a 15 ppm mass calibration offset. This high correlation means that the uncertainty in mass calibration will not change our overall results.

The NO_3^- CIMS was calibrated for H_2SO_4 by generating H_2SO_4 from SO_2 according to Kürten et al. [5]. We derived a calibration coefficient of 3.19×10^9 cm^{-3} with a systematic uncertainty of +50%/-33% [12]. As no method exists to produce standards for OOMs, we did not conduct a direct OOM calibration, but presume that they are charged upon collision similarly to H_2SO_4 and apply the same calibration coefficient to them. Similarly, the same calibration coefficient is applied to other strong acids (HIO_3, MSA).

NO_3^- CIMS quantitatively measures individual gas species, critical to NPF processes. As demonstrated by He et al [15], Brean et al. [12] and Yao et al. [15], the NO_3^- CIMS also captures the composition of small clusters in the atmosphere; we detected clusters containing iodine, sulfur, and nitrogen. The clusters are expressed as raw signal intensity only. Because the instrument was

operated without an inlet ion filter, these cluster signals represent a combination of chemically ionised neutral clusters, naturally charged ambient ions, and potential fragmentation products of larger clusters. As detailed in Supplementary Text 3, kinetic modelling suggests that at the measured formation rates, signals for specific strongly bound trimers (e.g., HIO_2-H_2SO_4-H_2SO_4) is potentially dominated by ambient natural ions rather than neutral clusters. However, because chamber studies demonstrate that the presence of a charge has minimal impact on the nucleation rates of these strongly bound systems, the detection of these clusters remains an indicator of the underlying chemistry

Determination of acid and oxygenated organic molecule concentrations

In the NO_3^- CIMS, compounds can undergo ionization either through proton-transfer reactions, where protons are taken from compounds such as H_2SO_4 to form HSO_4^-, or by forming clusters with the primary ions, forming clusters such as $H_2SO_4(HNO_3)_{0,1,2}NO_3^-$. Strong acids typically both deprotonate and cluster, while OOMs typically just cluster. $(HNO_3)_{0,1,2}NO_3^-$ concentrations also vary over time. To account for these variations in the total reagent ion counts, the neutral sulfuric acid concentration was quantified using the following equation [16]:

$$[\mathrm{H_2SO_4}] = \mathrm{C} \times \mathrm{SL_{H_2SO_4}} \times \ln\left(1 + \frac{[\mathrm{HSO_4^-}] + [\mathrm{HSO_4^-} \cdot \mathrm{HNO_3}]}{\sum_{i=0}^{2} [\mathrm{NO_3^-} \cdot (\mathrm{HNO_3})_i]}\right) \quad (1)$$

Where *i* equals 0, 1, or 2. *C* is the calibration coefficient. SL_{H2SO4} is the line losses, which were calculated from empirical equations for straight circular tubes with a laminar flow [17]. Here, MSA, HIO_3 and HIO_2 are assumed to have similar ionization efficiency and sensitivity in the NO_3^- CIMS to H_2SO_4 and therefore the same calibration coefficient was used to calculate the concentration of MSA, HIO_3 and HIO_2.

After obtaining the signal of OOMs from NO_3^- CIMS, the concentration of OOMs was estimated by the following equation [16]:

$$[\mathrm{OOM}_i] = \mathrm{C} \times T_i \times \mathrm{SL}_{\mathrm{OOM}_i} \times \ln\left(1 + \frac{[\mathrm{OOM}_i \cdot \mathrm{NO}_3^-]}{\sum_{i=0}^{2} [\mathrm{NO}_3^- \cdot (\mathrm{HNO}_3)_i]}\right) \qquad (2)$$

Where *i* equals 0, 1, or 2. *C* is the calibration factor. The concentration of I-OOMs is calculated the same way as in Equation (2). Here, we presume that every collision between an OOM and NO_3^- results in a cluster formation, and therefore use the same calibration factor as for H_2SO_4 [16]. The charging efficiency of certain OOMs with less functionalization is lower than that of H_2SO_4, as efficient clustering only occurs with molecules with two hydrogen bond donating groups [18]. As a result, the total OOM concentration obtained by NO_3^- CIMS is likely a conservative estimate [18,19]. T_i is the mass-dependent transmission efficiency of the instrument, which was estimated based on a previous study [20]. SL_{OOM} is used to correct the sampling line loss, which was also estimated based on previous study [17]. The diffusion coefficients are 0.0297 $cm^2\ s^{-1}$ for molecules following the formula $C_xH_yN_{0\text{-}2}O_z$ (with x = 3-10, y = 4-16 and z = 3-12) and 0.0240 $cm^2\ s^{-1}$ for molecules following the formula $C_xH_yN_{0\text{-}2}O_z$ (with x = 11-20, y = 10-32 and z = 3-18) [16].

Uncertainties in CIMS measurements are derived from chamber experiments and subsequently adapted for field conditions. For inorganic acids, while CLOUD chamber studies estimate sulfuric acid uncertainties at approximately a factor of 2 (+100%/−50%) [11], we adopt a conservative factor of 3 (+200% / -67%) to account for the additional inlet turbulence and environmental variability inherent to ship-borne measurements.

The uncertainty in OOM measurements arises from several compounding physical sources, including the application of the sulfuric acid calibration coefficient, mass-dependent

transmission efficiency, sampling line losses, and the charging efficiency of OOMs by nitrate ions. Because the calibration factor *C* is estimated based on the collision-limited sensitivity of H_2SO_4, it provides a lower-limit concentration for species charged less efficiently, such as OOMs [21]. Chamber studies reflect this limitation, suggesting ELVOC uncertainties of a factor of 1.8 (+80%/−45%), whilst acknowledging that less oxidised species (LVOCs/SVOCs) may be underestimated by up to a factor of 4 (+300% / -75%) due to lower charging efficiencies [16]. To comprehensively encompass these errors, we apply an overall uncertainty of a factor of 5 (+400% / -80%) to the total measured OOM concentrations.

For the comparisons with CLOUD chamber data, the systematic instrument uncertainties (factor of 3) for the quantification of HIO_x and H_2SO_4 were combined in quadrature with the relative atmospheric standard deviation of the measurement for each nucleation event ($\frac{\sigma_{std,X}}{X}$).

For each individual species (Extended Data Fig. 5) the total relative fractional uncertainties (δ_{tot}) were calculated as:

$$\delta_{\mathrm{tot}} = \sqrt{(\ln 3)^2 + \left(\frac{\sigma_{\mathrm{std,X}}}{\mathrm{X}}\right)^2}$$

For the multi-component plot (Figure 3b, $X = HIO_2 \times (H_2SO_4 + HIO_3)$), because the systematic uncertainties of the individual components are derived from the same instrument, they were treated as systematically correlated. The log-space errors compound linearly, resulting in a combined systematic variance of $2ln(3)$. This was combined in quadrature with the relative atmospheric standard deviation using the Gaussian formula:

$$\delta_{tot} = \sqrt{(2\ln 3)^2 + \left(\frac{\sigma_{std,X}}{X}\right)^2}$$

The absolute upper and lower bounds for all vapour concentrations were then determined by multiplying and dividing the total value X by the respective geometric error factor $\exp(\delta_{tot})$. This gave an average of a factor of 8.4 uncertainty on the x-axis of Figure 3b.

OOM volatility estimation

In the absence of sufficient molecular structural data, the volatility of OOMs was estimated using volatility parameterisation techniques that consider the number of carbon, oxygen, and nitrogen atoms in the molecule. This study utilized a widely accepted volatility parameterisation approach based on elemental composition, as supported by a recent study [19].

Given the lack of studies on the volatility of I-OOMs, the presence of iodine was presumed to reduce the saturation pressure by two orders of magnitude, equivalent to the effect of an ROH or ROOH functionality [22]. This assumption is based on iodine's heavy atomic weight, its small impact on molecular polarity, and the resulting enhanced intermolecular interactions, which collectively lead to significantly reduced but uncertain volatility.

$$\log_{10} C_i^*(300K) = (n_0 - n_\mathrm{C})b_\mathrm{C} - (n_\mathrm{O} - 3n_\mathrm{N})b_\mathrm{O} - 2\frac{(n_\mathrm{O}-3n_\mathrm{N})n_\mathrm{C}}{(n_\mathrm{C}+n_\mathrm{O}-3n_\mathrm{N})} b_\mathrm{CO} - n_\mathrm{N}b_\mathrm{N} - (n_\mathrm{I}b_\mathrm{I}) \quad (3)$$

where $n_0 = 25$, $b_C = 0.475$, $b_O = 0.2$, $b_{CO} = 0.9$, $b_N = 2.5$, and $b_I = 2.0$. Here, n_C, n_O, n_N, and n_I represent the number of carbon, oxygen, nitrogen, and iodine atoms in the compound respectively. Further details can be found in previous studies [19].

This parameterisation method is for the temperature of 300 K. The impact of temperature on C^* is corrected for different temperatures by the following equation [23]:

$$\log_{10} C_T^* = \log_{10} C_{300K}^* + \frac{\Delta H_\mathrm{vap}}{\mathrm{R} \times \ln(10)}\left(\frac{1}{300} - \frac{1}{T}\right) \quad (4)$$

R refers to the molar gas constant, and the value is 8.314 $J\cdot mol^{-1}\cdot K^{-1}$. The enthalpy changes associated with the evaporation of organic vapours are denoted by ΔH_{vap} and are calculated below [23,24]:

$$\Delta H_{\mathrm{vap}} = -5.7 \times \log_{10} C^{*}_{300K} + 129 \tag{5}$$

The ability of an OOM to condense on an aerosol depends on how much its concentration exceeds the saturation concentration. Practically, the Kelvin effect dictates that lower volatility is necessary for OOMs to condense onto smaller particles. The OOMs were categorized into extremely low-volatility organic compounds (ELVOC, with C* less than 10^{-4} $\mu g\cdot m^3$), low-volatility organic compounds (LVOC, with C* in the range of 10^{-4}-10^{-1} $\mu g\cdot m^3$) and semi-volatility organic compounds (SVOC, with C* in the range of 10^{-1}-$10^{-2.5}$ $\mu g\cdot m^3$) based on the $\log_{10}(C^*)$ [16].

4. Volatile organic compound (VOC) measurements

VOCs were measured using two complementary instruments: PTR-MS (Ionicon Analytik, Innsbruck) and two-dimensional gas chromatography time-of-flight mass spectrometry (2D-GC-MS). We consider OVOCs, such as aldehydes, as a subset of VOCs.

PTR-MS

PTR-MS provides high-time resolution measurements of VOCs and was housed in an air-conditioned container. Detailed descriptions of the instrument are provided by Sulzer et al. [25]. The PTR-MS sampled from a 30 m long common inlet line (10 mm outer diameter) running from a mast on the ship's bow to the container, with a flow rate of 35 L min^{-1}. The PTR-MS was operated with an inlet flow rate of 100 sccm and an E/N ratio (where E is the electric field strength and N is the buffer gas density) of 120 Td.

The PTR-MS was switched to sampling zero air produced by a Teledyne 701 zero-air generator (Teledyne API, San Diego) twice daily for 10-15 minutes and was calibrated twice weekly. Calibration was performed weekly using a 19-component VOC standard (Apel-Riemer Environmental Inc., Miami), which was dynamically diluted in zero air to provide a six-point calibration. The ToF power supply was tuned on June 2, 2022, and data were analyzed in two sections: before and after tuning. PTRViewer software version 3.2.7 (Ionicon Analytik, Innsbruck) was used to derive the VOC mixing ratios. The final VOC mixing ratios were obtained by subtracting the zero air concentrations from the ambient concentrations for each ion. Data were acquired by the PTR-MS at a 10 Hz time resolution.

2D-GC-MS

2D-GC-MS was utilized for the semi-quantitative analysis of isometrically separated molecules with a 24-hour time resolution. Gas-phase species were first collected onto a stainless-steel thermal adsorption tube at a flow rate of 1.5 L min^{-1} every 24 hours. The tube was packed with 1 cm of quartz wool and 300 mg of Carbograph 2TD 40/60 (Markes International, Llantrisant, UK), for subsequent analysis on the thermal desorbed (TD) coupled to the 2D-GC-MS, as detailed in the previous study [26]. Adsorption tubes were desorbed using a Thermal Desorption unit (Unity 2, Markes International, Llantrisant, UK) and subsequently analyzed using a gas chromatograph (GC, 7890B, Agilent Technologies, Wilmington, USA) equipped with a Zoex ZX2 cryogenic modulator (Houston, USA), interfaced with a Bench ToF-Select time-of-flight mass spectrometer (Markes International, Llantrisant, UK) using electron impact (EI) ionization at 70 and 14 eV. The adsorption tubes were desorbed onto the cold trap at 300 °C for 10 min, then purged onto the column. The initial temperature of the primary oven was held at 110 °C for 10 minutes, then increased at 3 °C min^{-1} to 300 °C, followed by an increase of 2 °C min^{-1} to

330 °C, and subsequently held for 3 minutes. The secondary oven was held at 110°C for 10 minutes, then ramped to 210 °C at 3.5 °C min^{-1}, followed by an increase of 2.5 °C min^{-1} to 330 °C, and subsequently held for 10 minutes. The transfer line temperature was set to 325 °C, and the ion source temperature was also maintained at 325 °C. A modulation time of 13 seconds was used, with a total run time of 91 minutes per sample. Separation was performed using primary (SGE BPX5, 30 m × 0.25 mm inner diameter (ID) × 0.25 µm-5% phenyl polysilphenylene-siloxane) and secondary (SGE BPX50, 4.0 m × 0.1 mm ID × 0.1 µm-50% phenyl polysilphenylene-siloxane) columns. Data analyses were conducted using GC Image v2.3 software (Zoex Corporation, Houston, USA). For the semi-quantification of aldehydes, we performed an eight-point external calibration using authentic standards.

5. Trace gases (O_3, NO_x, CO, SO_2 and NH_3) measurements

O_3, NO_x and CO analyzers

The sampling inlets were positioned on the roof of the container located on the fore-deck, utilizing three separate inlets. Each inlet was equipped with a funnel to prevent water from entering the lines.

The O_3 was measured using a Thermo 49iQ ozone analyzer, equipped with an internal pump and operated at a flow rate of 1 L min^{-1}. The detection limit for O_3 during the campaign was 0.5 ppbv.

NO_x was measured using a Teledyne T200UP analyzer, which underwent automatic zeroing for 10 minutes every 23 hours using an NO_x trap. The NO_x analyzer was calibrated weekly during the campaign using a NO gas cylinder (BOC, 98 ± 5% ppbv NO, > 0.001 ppbv NO_2, verified against the National Physical Laboratory (NPL) standard in York). An external pump with a flow

rate of 1 L min^{-1} was employed for the NO_x instrument. The detection limit for this campaign is established at 50 pptv.

The CO measurements were conducted using an AL5005 Aerolaser, which was automatically calibrated and zeroed daily throughout the campaign. This Aerolaser model utilized an external pump that sampled at a flow rate of 180 ml min^{-1}. The detection limit for this device is 1 ppbv.

Laser-induced fluorescence (LIF)

Highly sensitive measurements of sulfur dioxide (SO_2) were conducted using a custom-built LIF instrument developed by the University of York. This instrument is based on the system originally demonstrated by Rollins et al. [27]. The sample flow rate was maintained at 2 slpm, with the measurement and reference LIF cells operated at low pressure (~600 mbar) to maximize the SO_2 fluorescence lifetime. The instrument inlet was located on the roof of the container, and a critical orifice was used to reduce the pressure in the ~10 m inlet line, minimizing wall effects. Multi-point calibrations were conducted approximately every 1.5 hours using a 5 ppm SO_2 standard (BOC gases) across the expected concentration range to ensure accurate instrument sensitivity characterization and standard additions used to characterize inlet effects. During DY151 deployment, the LIF-SO_2 instrument achieved a 3σ limit of detection of 15 pptv with a 5-minute averaging time and a mixing ratio uncertainty of 15 % + 5 pptv. As this deployment was one of the first field uses for the University of York LIF-SO_2 instrument and encountered several issues impacting data coverage, approximately two weeks of data were collected between 5th and 19th June 2022.

Ammonia analyser

The NH_3 measurement was carried out by an Automated Ammonia Analyzer (AiRRmonia, Mechatronics B.V., NL). The AiRRmonia employs a wet-chemistry approach for NH_3 analysis, utilizing a selective diffusion membrane in combination with conductivity measurements. The detection limit is around 0.05 µg m^{-3} (equivalent to ~65 pptv at STP, 0 °C) [28].

6. Single Particle Soot Photometer-Extended Range (SP2-XR)

Single Particle Soot Photometer-Extended Range (SP2-XR) (DMT, US) was utilized to measure black carbon (BC) with an intracavity Nd:YAG laser (1064 nm). This instrument applies laser-induced incandescence to determine the refractory BC mass of single particles, independent of their mixing state. It measures both incandescence and light scattering from particles, enabling simultaneous assessments of BC mass and optical particle size. The SP2-XR operates across particle sizes ranging from 50 to 800 nm for incandescence detection and 100 nm to 500 nm for scattering, with a data acquisition rate of up to 50,000 particles cm^{-3}. The flow rate was set at 0.03 L min^{-1}. In this paper, we used the total BC mass concentration only.

7. Aerosol composition: High-resolution time-of-flight Aerosol Mass Spectrometer (HR-ToF-AMS)

The mass concentrations of aerosol chemical species were determined using a high-resolution time-of-flight (ToF) aerosol mass spectrometer (AMS). The AMS measured organics, sulfate, nitrate, ammonium, and chloride. Data were analyzed with Squirrel Toolkit 1.62D for unit-mass resolution and Pika Toolkit 1.22D for high resolution. Mass spectra were fitted from 12 amu to 180 amu. Detailed descriptions of AMS standard calibrations are provided in Canagaratna et al. [29] and Zhang et al. [30]. A default collection efficiency of 0.5 was used in this study. Sea salt quantification by AMS was based on generating a known concentration of sea salt standard

solution with the specific ion $NaCl^+$ as a representative ion for sea salt samples. Relative ion efficiency and scaling factors were determined to quantify the sea salt mass concentration. Further details on AMS measurements and chemical species quantification are available in Zhang et al. [31]. For further details of AMS positive matrix factorization (PMF), please see SI section 12.

8. Cloud Condensation Nuclei (CCN) concentration: CCN-100 Counter

The CCN-100 with a computer display, DOC-0086 Revision M (DMT, US) was used to measure the number of CCN [32]. The CCN Counter exposes an aerosol sample flow to controlled levels of water supersaturation (SS) of 0.1%, 0.2%, 0.3%, 0.5%, and 0.7%. The measurement time for SS=0.1% was 20 min, while it was 10 min for each other supersaturation level. The sample flow rate used in the CCN Counter is 0.5 L min^{-1}. To reduce the uncertainty of the instrument due to the change of SS, the data at the beginning of 5 min at each SS were discarded.

9. Chlorophyll-*a* (Chl-*a*) measurement from Absorption spectrophotometry

Absorption-attenuation instruments were integrated into the ship's clean flow-through system, which supplies seawater from a depth of approximately 6 meters below the hull. The method for determining Chl-*a* using underway absorption spectrophotometry is detailed in Dall'Olmo et al [33]. Briefly, seawater was first passed through a debubbler (Vortex) and then through the WETLabs Absorption and Attenuation Sensor (ACS) system (hyperspectral data 400–750 nm, 5 nm spectral resolution, 15 nm band pass). The seawater also passed through a Cole Parmer 0.2 μm cartridge filter for 10 min on every hour), and the 0.2 μm filtered absorption was used as a baseline to determine particulate absorption, ap(λ) [34]. These data were then converted into 1 min

median-averaged bins. With an average ship speed of ~18 km h^{-1}, each 1-min binned median represents approximately 0.3 km of the transect (except at stations) [34] .

ap(λ), data at 650, 676 and 715 nm were then used to estimate Chl-*a* concentrations using the line height method of Boss et al. [35] as follows:

$$\text{Chl-}a^{\text{ACS}} = \left[a_p(676) - \frac{39}{65a_p(650)} - 26/65a_p(715)\right]/0.014 \qquad (6)$$

where $Chl\text{-}a^{ACS}$ is the estimate of Chl-*a* from the ACS and 0.014 m^2 mg^{-1} Chl-*a* is a typical estimate of Chl- *a* specific absorption at 676 nm. Past validation line-height Chl- *a* values with high performance liquid chromatography (HPLC) has shown that the measurement uncertainty is typically 10-15% for the system operated.

10. Classification of nucleation events

This study focuses on a 24-day period, from 23rd May to 15th June. The broader expedition encompasses a total of 38 days, which includes sailing time across the North Atlantic Ocean (from 19th to 22nd May) and the return trip to Southampton (from 16th to 26th June). We have identified three distinct event types that will be the primary focus of our analysis based on the established criteria [36-38]. These event types are:

- New Particle Formation (NPF) Event: These events are characterized by the formation of small particles, notably in the 3–5 nm range. Such events typically exhibit minimal or no pre-existing particles within the smallest size categories at their onset and continuous growth for at least several hours. The particles usually grow to a diameter exceeding 20 nm.

- Burst Event: These events are marked by the formation of small particles. There is a noticeable increase in ion numbers within the 3-5 nm range; however, these particles do not exhibit subsequent growth.
- Non-event Event: These observations are characterized by an absence of both particle nucleation and growth.

N.b., days that are heavily influenced by the ship's own emissions are excluded in this category.

11. Determination of particle formation and growth rates

Formation rate

The particle formation rates (J_{d_p}) were quantified using the equation [36]:

$$J_{d_\mathrm{p}} = \frac{dN_{d_\mathrm{p}}}{dt} + \mathrm{CoagS}_{d_\mathrm{p}} \cdot N_{d_\mathrm{p}} + \frac{\mathrm{GR}}{\Delta d_\mathrm{p}} \cdot N_{d_\mathrm{p}} \tag{7}$$

The parameter $CoagS_{d_p}$ represents the particle sink due to coagulation, while N_{d_p} refers to the particle concentration within a specific size range [*dp, dp+Δdp*]. Meanwhile, *GR* denotes the rate of particle growth within the same size range. The first term on the right-hand side of the Eq. (7) represents the rate at which particles reach a size of d_p. The latter two terms represent losses from this size due to coagulation ($CoagS_{d_p}$) and growth, respectively. More discussion about the J_{d_p} calculation can be found in reference [36]. To determine $CoagS_{d_p}$ for particles with a specific diameter d_p, the number size distribution obtained from the NAIS (4 to 25 nm) particle mode and two MPSSs (nano-MPSS: 25 to 40 nm and long-MPSS: 40 to 736 nm) were used. The equation for the $CoagS_{d_p}$ estimation is shown below:

$$\mathrm{CoagS}_{d_\mathrm{p}} = \int K(d_\mathrm{p}, d'_\mathrm{p}) n(d'_\mathrm{p}) dd'_\mathrm{p} \cong \sum_{d'_\mathrm{p}=d_\mathrm{p}}^{d'_\mathrm{p}=max} K(d_\mathrm{p}, d'_\mathrm{p}) N_{d'_\mathrm{p}} \tag{8}$$

where $K(d_\mathrm{p}, d'_\mathrm{p})$ is the coagulation coefficient between particles of d_p and d_p' sizes and $N_{d'_\mathrm{p}}$ is the particle concentration in the size bin $[d_\mathrm{p}, d'_\mathrm{p}]$. To account for the many physical and instrumental uncertainties inherent to measurement and description of sub-10 nm particles including unconstrained variations in the ultrafine coagulation sink [39] we applied a conservative baseline systematic relative uncertainty of a factor of 2 (100% / -50%) to J_4.

The condensation sink (CS) describes the rate of vapour condensation to the existing particle population. It is estimated based on the following equation:

$$\mathrm{CS} = 2\pi D \int_0^{d_\mathrm{p}^\mathrm{max}} \beta_\mathrm{m}(d'_\mathrm{p}) d'_\mathrm{p} N_{d'_\mathrm{p}} dd'_\mathrm{p} = 4\pi D \sum_{d'_\mathrm{p}} \beta_{m,d'_\mathrm{p}} d'_\mathrm{p} N_{d'_\mathrm{p}} \tag{9}$$

Where D represents the diffusion coefficient of the condensing vapour, which is here assumed to be sulfuric acid. The transitional regime correction factor is denoted by $\beta_{m,dp}$ which is estimated based on the equation of

$$\beta_{\mathrm{m},d\mathrm{p}} = \frac{1 + Kn}{1 + 1.677Kn + 1.333Kn^2} \tag{10}$$

which $Kn = \frac{2\lambda}{d_p}$ is defined as a function of Knudsen number [36].

The rate of formation (J_4) was calculated for the particle mode of NAIS within the size range of [4 nm, 7 nm]. The relationship between the particle formation rate J_4 and $J_{1.7}$ was calculated according to Cai *et. al.* [39]:

$$J_{1.7}(t) = J_4(t) \times \exp\left[\int_{d_4}^{d_{1.7}} -\frac{\mathrm{CoagS}(d_\mathrm{p})}{\mathrm{GR}(d_\mathrm{p})} \cdot \mathrm{d}d_\mathrm{p}\right] \tag{11}$$

Where $GR(d_{p4})$ was derived from the NAIS dataset using the maximum-concentration method (as described in the growth rate section). $GR(d_{p1.7})$ is obtained from condensation of acids (H_2SO_4, HIO_3, MSA) to estimate growth rates for diameters from 1.7-4 nm according to the dynamic aerosol growth modelling [12] (as described in the dynamic aerosol growth modelling section). Then, the growth rate was obtained by the least-squares fitting method.

Because $J_{1.7}$ were inferred rather than measured directly, uncertainties were propagated through the survival probability framework following Cai et al. (2022) [39]. The overall uncertainty for $J_{1.7}$ comprises the baseline uncertainty in J_4 (factor of 2, see above). The errors regarding CoagS were approximated to be equivalent to the errors equivalent to quantification of particle number (30%) and those relating to the growth rate to be 50%.

Due to the exponential nature of the survival probability scaling, the resulting uncertainties are log-normally distributed. The relative systematic uncertainty ($\delta_{sys,J}$) was calculated using the Gaussian error propagation formula, combining the log-normal variance of J_4 (ln 2) with the fractional variances of the sink and growth rate:

$$\delta_{\mathrm{sys,J}} = \sqrt{(\ln\ 2)^2 + \mathrm{E}^2\,[\,0.30^2 + 0.50^2\,]}$$

Where E is the integrated ratio of the coagulation sink to the growth rate across the 1.7 to 4 nm interval ($E = \Delta d_p \frac{CoagS}{GR}$). To determine the total fractional uncertainty ($\delta_{tot,J}$) visualised as error bars in Figure 3b and Extended Data Fig. 5, this systematic error was combined in quadrature with the relative standard deviation ($\frac{\sigma_{std,J}}{J_{1.7}}$) of the measurement period:

$$\delta_{tot,J} = \sqrt{\delta_{sys,J}^2 + \left(\frac{\sigma_{std,J}}{J_{1.7}}\right)^2}$$

Using this methodology, we estimate our uncertainty in our J values to be approximately a factor of 3.2 This falls within the uncertainty range of factor 2–5 reported in recent polar aerosol studies [40].

Growth rate (GR) calculation

The GR of particles is defined as the rate of change of their diameter using the following equation [36]. In this study, the maximum-concentration method was used to determine particle growth rates for different diameter ranges based on PNSD data [36]. With this method, for each measurement, the diameter of the growing mode is defined as the center of the size bin with the maximum particle concentration. Growth rates were calculated as the slopes of linear fits to diameter-versus-time data using the least-squares minimization method.

$$\mathrm{GR} = \frac{\mathrm{d}d_{\mathrm{p,mode}}}{\mathrm{d}t} \tag{12}$$

Uncertainties in GR determination are estimated to be approximately ~50% [41]. Because these measurements were conducted on a mobile shipborne platform, the inferred apparent growth rates reflect the net contribution of multiple processes, including vapour condensation, coagulation, continuous nucleation, and the transport and dilution of spatially inhomogeneous aerosols.

Dynamic aerosol growth modelling: organic vapours

We modelled the growth rates of new particles due to the condensation of OOMs as measured by NO_3^- CIMS using a dynamic aerosol growth model. 591 OOMs and 91 I-OOMs were identified

in the *m/z* range of 200−550 during the campaign, and their saturation vapour concentrations were estimated according to Mohr et al. [19]. We utilize all OOMs with $\log_{10}C^* < 10^{2.5}$, including SVOCs, LVOCs, and ELVOCs. The model was run from the onset and until the end of the NPF event, defined as when the new mode of particles disappears.

$$\frac{\mathrm{d}d_p}{\mathrm{d}t} = \left(\frac{d_p + d_i}{d_p}\right)^2 \frac{\bar{c}_{i,p}}{2\rho} \alpha_{i,p}\beta_{i,p}\left[C_i^v - a_{i,p}C_i^0\right] \tag{13}$$

where dp and di are the diameters of particle and condensable vapour in meters; $\bar{c}_{i,p}$ is the mean relative thermal velocity between a condensable vapour molecule and a particle in $m \cdot s^{-1}$ and is calculated based on Eq. (14); ρ is the density of particle and is assumed to be 1.4×10^6 $g \cdot m^{-3}$ [16]; $\alpha_{i,p}$ is the accommodation coefficient and assumed to be 1; $\beta_{i,p}$ is Kinetic oriented transition-regime diffusion correction (Eq. 15); C_i^v and C_i^0 are the vapour concentration and saturation vapour concentration, respectively in m^{-3}; and $a_{i,p}$ is the particle phase activity.

$$\bar{c}_{i,p} = \sqrt{\frac{8RT\left(M_i + M_p\right)}{\pi M_i M_p}} \tag{14}$$

where R is the molar gas constant, $J \cdot mol^{-1} \cdot K^{-1}$; T is temperature, K; M_i and M_p are the molar mass of organic vapours and particles, respectively, $g \cdot mol^{-1}$. $\beta_{i,p}$ is the function of Knudsen number (Kn) and is calculated based on the following equation:

$$\beta_{i,p} = \frac{Kn(1 + Kn)}{Kn^2 + Kn + 0.283Kn\alpha_{i,p} + 0.75\alpha_{i,p}} \tag{15}$$

$Kn = \frac{2\lambda}{d_p}$, where λ is the mean free path, m. $a_{i,p}$ is the particle phase activity and calculated using the equation: $a_{i,p} = X_{i,p}\gamma_{i,p}K_{i,p}$ where $X_{i,p}$ is the mass fraction of vapour *i* in particle phase, $\gamma_{i,p}$ is the mass-based activity coefficient in the organic condensed phase and assumed to be 1, and

$K_{i,p}$ is the Kelvin coefficient, estimated as $K_{i,p} = 10^{\frac{5e9}{d_p}}$. $X_{i,p} = \frac{C_{i,p}^S}{C_p^S}$ represents the mass fraction of vapour *i* in particle phase, where $C_{i,p}^S$ is the mass of vapour *i* in particle *p*, and C_p^S is the sum of $C_{i,p}^S$ for all the condensed vapours on particle p. We simulated $X_{i,p}$ through calculating the cumulative mass of organics on particles ($C_{i,p}^S$). The change of $C_{i,p}^S$ can be obtained via the equation of condensation flux $\emptyset_{i,p} = N_p(d_p + d_i)^2 \frac{\pi \cdot \bar{c}_{i,p}}{4} \alpha_{i,p}\beta_{i,p}[C_i^v - a_{i,p}C_i^0]$. where N_p is the number concentration of particle *p*, cm^{-3}. Based on the cumulative mass of $C_{i,p}^S$, we calculated its fraction in the total particulate mass and thus obtain the $X_{i,p}$ matrix for each time points at each particle size. $X_{i,p}$ is assumed to be 0 at the starting point. C_i^0 is the saturation concentration of vapours and was estimated using Eqs. (3-5), which considers the chemical compositions of the vapour species.

Dynamic aerosol growth modelling: acids

To estimate the growth of aerosol particles due to the condensation of sulfuric acid, MSA and iodic acid, we utilize Eq. (16) [42,43]. Specifically, the vapour concentration data from the NO_3^- CIMS was used to estimate the rate at which these acids condensed onto the sampled particles.

$$GR = \frac{4k_{\text{coll}}\,\alpha_{i,p}V_i^v C_i^v}{\pi d_p^2} \quad (16)$$

d_p is the growing particle mass diameter. k_{coll} is the is the kinetic collision rate between particle and vapour (m^3 s^{-1}), which is corrected by the London-van-der-Waals forces and estimated based on the Eq. (18), m^3 s^{-1}. $\alpha_{i,p}$ is accommodation coefficient, which is assumed to be 1; V_i^v is the volume of vapour molecule, m^3; C_i^v is the vapour concentration (m^{-3}).

$$k_{\text{coll}}\left(d_i, d_p\right) = k_K\left(\sqrt{1+\left(\frac{k_K}{2k_D}\right)^2}\right) - \frac{k_K}{2k_D} \tag{17}$$

where k_K and k_D are the kinetic collision rates for the free molecule and continuum regime, respectively, and are calculated as follows:

$$k_K = \frac{\pi}{4} \cdot \left(d_i + d_p\right)^2 \cdot \left(\frac{8kT}{\pi}\right)^{1/2} \cdot \left(\frac{1}{M_i} + \frac{1}{M_p}\right)^{1/2} \cdot E(\infty) \tag{18}$$

$$k_{\text{D}} = 2\pi \cdot \left(d_i + d_p\right) \cdot \left(D_i + D_p\right) \cdot E(0) \tag{19}$$

d_i is the diameter of vapour molecules, m. M_i and M_p are the molar masses of condensing species and particle, g mol^{-1}; D_i and D_p are the diffusion coefficients of the condensing species and particles, respectively, m^2 s^{-1}. $E(\infty)$ and *E(0)* are collision enhancement factors due to London-van-der-Waals forces, which are detailed in a previous study [44].

12. Source apportionment of gas-phase OOMs and aerosol particles: Positive Matrix Factorization (PMF)

Gas-phase OOMs

PMF is a well-established receptor model used to solve functional mixing models when the source profiles are unknown and presumed to be constant [45]. PMF solutions are constrained to be non-negative, and a least squares algorithm is applied which accounts for uncertainties in the dataset, downweighing uncertain elements. PMF is therefore quantitative and identifies physically meaningful sources. The number of factors is determined based on the interpretability of the results. These benefits make it suitable to source-apportion NO_3^-CIMS data. PMF analysis here is performed in the PMF2 program on the time series of all OOMs with more than 4 carbon

atoms and 3 oxygen atoms, excluding small organic acids, H_2SO_4, HIO_3, MSA, and the reagent ions. The mass balance as applied to mass spectral data can be described as follows:

$$X_{ij} = \sum_{k=1}^{p} g_{ik} \cdot f_{kj} + e_{ij} \quad (20)$$

Where the jth compound on the ith observation is represented by x_{ij}. The first term on the right-hand side, g_{ik} represents the contribution of the kth factor (of a total of p factors) to the receptor on the ith hour. The second term, f_{kj}, represents the fraction of the total of the jth compound to the kth factor. The third term, e_{ij}, is the residual for the measurements in the jth compound on the ith hour. Here, the G matrix represents the time-series of the strength of source k, and the F matrix represents the mass spectrum produced from source k. The PMF program runs to minimize the sum of squared residuals scaled by their measurement uncertainty (Q), meaning the results are heavily determined by the relative input uncertainties. Q is defined as:

$$Q = \sum_{i=1}^{m} \sum_{j=1}^{n} \left(\frac{e_{ij}}{s_{ij}}\right)^2 \quad (21)$$

Where s_{ij} is the input measurement uncertainty of compound j at time i, and e_{ij} is the model residual of compound j at time i. Here, PMF is run in *robust mode*, where strong outliers with $e_{ij}:s_{ij} > \alpha$ are reduced to α. In all model runs here, $\alpha = 4$. Error estimates are not readily available for Nitrate CIMS data, so here we take the error matrix to be equal to

$$s_{ij} = \sqrt{I_{ij}} \cdot \alpha \quad (22)$$

Where I is the signal intensity of species j and time i, and α is a constant [46]. The Q/Q^{theory} values for our chosen solutions are near-unity.

Aerosol particles

PMF is also used for the source apportionment of organic aerosol measured by the AMS. High-resolution organic mass spectra and their uncertainties, extracted from the AMS data [47], were inputted into the Igor-based software package, Source Finder (SOFI-8) [48], to conduct the PMF analysis. The organic mass matrix and its error matrix were examined before running the PMF model, during which incorrect, noisy, and very low signals were eliminated. PMF solutions with unconstrained factor numbers ranging from 2 to 15 were tested. Despite the non-negativity constraints that limit allowable rotations, numerous potential rotations and corresponding solutions were evaluated; however, no significant differences among these solutions were noted. The optimal solution was identified as a seven-factor model describing different kinds of organic aerosol (OA), consisting of hydrocarbon-like OA (HOA), cooking OA (COA), marine secondary OA (MSOA), sea salt OA, two types of semi-volatile OA (SVOA and SVOA1), and low-volatile OA (LVOA) based on time series, diel patterns, factor profiles (especially by representative markers), and correlation with other measurements in this campaign. Normally, m/z 55 and m/z 57 are the markers for HOA, m/z 39, 41 and 55 are the markers for COA, the relative intensity of m/z 43 and m/z 44 are used to identify SVOA and LVOA.

Furthermore, a specialized input matrix was constructed for analysis by the PMF model to enable separation of iodine originating from sea salt spray and regional iodine. Specific ion signals from sea salt components (Cl^+, HCl^+, $NaCl^+$) and various iodine species related ions (I^+, HI^+, CH_2I^+) were input into the model. The model successfully distinguished between iodine-related organic aerosols (named IOA) and sea salt factors. The sea salt contains little iodine, while the IOA factor contains the majority of the iodine-containing signals, with highest signals coincident with new particle growth to sizes detectable by the AMS (2nd and 9th June). Temporal variations and mass spectrum profiles for these factors are presented in Fig. S9.

13. Back trajectories & maps

Back-trajectories were calculated using the NOAA HYSPLIT (Hybrid Single-Particle Lagrangian Integrated Trajectory) model [49], which tracks air parcels backward in time from a receptor location using three-dimensional wind fields. Trajectories were driven by Global Data Assimilation System (GDAS) meteorological reanalysis data [50]. Sea ice concentration data were obtained from the National Snow and Ice Data Center (NSIDC) passive microwave sea ice concentration product on a 25 km grid [51]. Base map data used for all visualisations are from Natural Earth (naturalearthdata.com), which are in the public domain.

Supplementary Text

1. Advancing the Understanding of Arctic NPF: Context and Future Directions

1.1 Nucleation

Figure S1a illustrates our current understanding of nucleation mechanisms in the Arctic, based on respective statements made in published papers and our new results. Note that this figure is slightly different to Figure 1 in Schmale and Baccarini [52].

Two recent studies provided molecular level information on nucleation processes in the Arctic. Beck et al. [14] observed NPF across two seasons and two Arctic sites. At Ny-Ålesund, they concluded that nucleation is driven by H_2SO_4 and NH_3; at Villum, they reported that springtime nucleation is driven by iodic acid, while in summer, nucleating clusters consist of H_2SO_4 and NH_3. Baccarini et al. [13] reported that all the new particle formation events observed over the central Arctic pack ice during their late-summer/early-autumn campaign were driven primarily by HIO_3, with little contribution from sulfuric acid. Their measurements therefore identify HIO_3 as the dominant nucleating vapour during their campaign.

The Antarctic differs significantly from the Arctic in terms of atmospheric chemistry. Sources of molecular iodine and organics are considerably weaker, and NPF appears to occur exclusively via sulfuric acid nucleation with ammonia and amines [12,40,53,54]. This contrast is likely due to the distinct nature of the Arctic, including its extensive coastlines with high freshwater discharge, distinct oceanic biology, and larger annual ice melt.

These studies showed multiple components contributing to nucleation in polar regions, with a dependence on the season and location – they significantly advanced our understanding of NPF processes in a region where large-scale in-situ observations are highly challenging.

Here, we found in-situ evidence of synergistic, multi-component nucleation (i.e., multiple components nucleate simultaneously) in the Arctic atmosphere, specifically HIO_3, HIO_2 and H_2SO_4. This validates the newly proposed mechanism by He et al. [10] through the CLOUD chamber experiment. This connection was only possible because the laboratory characterisation preceded our ambient measurements; earlier field studies, even where the relevant precursors were detected, lacked the framework or necessary data needed to identify it.We also detected a DMA-containing cluster. Although this is unambiguously detected only during a short period of time in this campaign, indicating that amines are present at low concentrations. Nonetheless, its presence indicates that trace amines may participate in the clustering process in this region. DMA is capable of enhancing nucleation rates at sub-pptv concentrations [55]. Finally, we observe high concentrations of OOMs, which have been shown to be capable of enhancing the nucleation rate of sulfuric acid clusters [55,56]. Our analysis did not provide concrete evidence to support or refute their role in nucleation.

1.2 Growth of new particles

Figure S1b illustrates our current understanding of new particle growth mechanisms in the Arctic, based on respective statements made in published papers and our new results. Note that this figure is slightly different to Figure 1 in Schmale and Baccarini [52].

Figure S1b suggests that there is emerging evidence that organics contribute to particle growth in the Arctic, including two sets of CIMS measurements. For example, Beck et al. [14], suggested that

OOMs likely contribute to summertime particle growth at their Svalbard site, although the molecular composition of the condensing vapours were not determined. In contrast, Baccarini et al. [13] reported that the NPF events they observed over the central Arctic pack ice were driven by HIO_3, with negligible contribution from sulfuric acid. This indicates that particle growth mechanisms vary regionally across the Arctic.

There is also a large body of literature regarding the growth of new particles or pre-existing particles in the Arctic. For example, Willis et al. [57] observed nucleation-mode particles between 5-20 nm growing into sizes above ~50 nm during a summertime particle growth event at the Canadian archipelago; the growth was correlated with organic compounds measured in particles of ~80nm and larger by an HR-AMS (>70 nm). Furthermore, based on AMS observations (~80nm and larger), Willis et al. [58] reported that organic aerosol and MSA:sulfate ratios increased toward the surface and were positively correlated with air-mass residence time over open water in the Canadian Arctic; based on these correlations, they interpreted the aerosol as being strongly influenced by marine-biogenic sources and suggested that such organic material contributes to the summertime aerosol population that reaches CCN-active sizes. Burkart et al. [59] suggested that the growth of two modes of particles is largely via organic condensation, based on a comparison of modelling with the evolution of PNSDs. Croft et al. [60] used an aerosol microphysical model to show that condensable organic vapours are important for reproducing observed summertime aerosol size distributions above ~20 nm in the Canadian Arctic. They suggested that these vapours may have a marine, likely biogenic origin, while noting that the specific precursor emissions and formation mechanisms remain uncertain. Other work has pointed towards sea-surface microlayer derived aldehydes as a potential precursor for these organic vapours [61,62]. These led to the prevailing hypothesis that organic compounds facilitate particle growth to CCN sizes in the Arctic.

Our data supported the above hypothesis, confirming that particle growth is mainly driven by OOMs rather than inorganic acids, with low volatility (LVOC) and extremely low volatility (ELVOC) playing a key role. Furthermore, we estimated that the new class of OOMs that contain iodine contributes substantially to modelled GRs.

1.3 Molecular understanding from nucleation and growth to CCN

A key challenge in Arctic studies has been to observationally connect an NPF event all the way through to a measurable increase in CCN concentration. Previous studies typically estimated a size that would be CCN active [13,14,63]. For example, Beck et al. [14] speculate that atmospheric NPF observed at Ny-Ålesund, despite a rather slow consequent growth to Aitken mode size aerosol, may also contribute to the regional CCN budget. Baccarini et al. [13] provide a size distribution of cloud residuals, many of which have crossover with the diameter of growing particles, and therefore inferred that iodine NPF has the potential to influence CCN and cloud properties. PNSDs down to 5 nm and CCN measurements were made during NETCARE campaigns [63], and they suggested that marine secondary organic aerosol was a major CCN source, and that these particles could become CCN active at <50 nm, attributed to the low concentrations of larger particles. Song et al. [64] also found significant contribution of NPF to particles that could be CCN active in Svalbard. Therefore, previous evidence strongly suggests that NPF often contributes substantially to CCN in the Arctic. However, most available datasets do not track the same particle population continuously from nucleation to CCN activation in situ.

Our work provides a complete observational closure by tracking the full progression within a single particle population, from nucleation, to growth and CCN, at molecular level. This closes the remaining evidential gap by directly demonstrating that nucleated particles grow to CCN-active sizes in the ambient Arctic atmosphere.

1.4 Remaining gaps and future work on molecular understanding from nucleation and growth to CCN in polar regions

While our study provides a more complete picture of Arctic NPF, it also highlights several key uncertainties that require further investigation.

Future work combining targeted field campaigns, capturing the whole NPF process (from emissions of precursors gases to nucleation, to growth, and CCN), with laboratory studies and regional modelling is needed to address these gaps and accurately represent these complex processes in climate models, for example

1) Spatially and temporally resolved emission fluxes of precursors like iodine, DMA, and aldehydes in the Arctic
2) The role of amines and ULVOC in nucleation processes, involving both iodine and sulfur species, through both laboratory simulations and field observations
3) The formation mechanism and atmospheric fate of the I-OOMs and aldehyde-OOMs, and their contribution to new particle growth spatially and temporally
4) Relative importance of NPF to the CCN budget spatially and temporally, and ultimately to clouds and radiative forcing in polar regions

2. Identification of contamination from ship's plume and calculation of background tracer concentrations

The nature of ship observations means that there is potential influence of ship plume emissions on the atmospheric observations, particularly during sailing when there is limited to no control of the relative direction between the ship and wind. This can be seen clearly on 3rd-4th and 13th June (Fig. S2).

The RRS Discovery has a dynamic positioning system to ensure that the front of the ship always faces the wind while stationary to minimize the impact of ship's stack emissions. However, this does not always prevent contamination, particularly when the wind speed is low and wind direction changes frequently. Distinct spikes in various air pollutant tracers are depicted in Fig. S2. We found more spikes in NO, NO_2, and BC (Figs. S2a and 3c), than those of the aromatic hydrocarbons ($C_7H_8H^+$ and $C_8H_{10}H^+$) from PTR-MS (Fig. S2d). As the PTR inlet was on the front mast, while all other instruments were in the container, we can confirm that the front mast experienced a much lower frequency of ship-stack emissions. This is particularly evident on 7th and 8th June, when we only see two small spikes for the aromatic hydrocarbons from the PTR-MS (Fig. S2d). Such a phenomenon can be explained by the turbulent circum-ship transport of stack emissions towards the containers, on top of which the inlets for all instruments except PTR-MS inlets were installed, but there is more limited pollution to the PTR-MS inlet which was installed at the front mast, a few meters higher than the top of the container and 30 m further away from the ship stack than the containers. Our PTR measurements are therefore minimally influenced by ship stack emissions. We see no evidence in the PTR-MS that the ship influenced the regional background of VOC emissions on 7th and 8th June (Fig. S2). We recommend that future ship-borne atmospheric observations use the front mast as inlets when possible. However, this does not apply to fast-response species, which require shorter inlets as ship stack contamination can be filtered out for these species.

All NPF events occurred while the ship was stationary. Although during these events sporadic ship plumes were observed, they disappeared quickly, and we saw no evidence that they affected particle growth rates, i.e., no sudden changes (Extended Data Fig. 9). Furthermore, we did not observe any concurrent spikes or sudden changes in anthropogenic OOMs factor during such

contamination periods (Extended Data Fig. 8), further illustrating that ship stack emissions do not have detectable effects on either the nucleation or growth of new particles. Consistent with these results, the ship-stack emission typically take less than 1 minute to reach the container, but oxidation and condensation take place over the course of hours; thus we would not expect any measurable influence of ship-stack emissions on our observed NPF.

To obtain the background concentrations of key gaseous species and BC, we identified periods when ship stack emissions were being sampled based on meteorological data, mixing ratios of NO_x and SO_2 and BC (Fig. S2). The relative wind speed (RWS) and relative wind direction (RWD) were used to determine when the emissions from the RRS Discovery were being blown towards the sample inlets (RWD between 157.5° and 202.5°) or when, due to low RWS (below 3 m s^{-1}), the ship emissions were not rapidly dispersed away from the ship. Spikes in the NO_x, SO_2 and BC time series were then used to identify periods when emissions from other vessels or from land-based human activity were being sampled. The plume periods were identified by concurrent increases in the concentration of ship stack tracers: NO and NO_2 (Fig. S2a), SO_2 (Fig. S2b), and BC (Fig. S2c).

3. Limit of detection for NO_3^- CIMS and influence of ambient ions

The LOD for the NO_3^- CIMS as has been published previously is about 10^4 cm^{-3} [6]. In practice, this depends on averaging times, backgrounds, and interferences. Of backgrounds and interferences, backgrounds are more significant, as interferences can be accounted for via peak fitting with longer averaging times. The sensitivity of our instrument is around 240 ions·$pptv^{-1}$.

The detection limits for clusters involving H_2SO_4, HIO_3, HIO_2, and DMA were influenced by both instrumental noise and interference from adjacent peaks in the mass spectra. Our clusters are often

separated from the adjacent peaks due to the negative mass defect of iodine and sulfur, however, their effective LODs are still higher than those derived purely from instrumental noise and vary over time depending on these overlapping signals. For example, when the signals from interfering species at the same nominal mass are stronger than those of the target clusters, reliable fitting becomes challenging, as illustrated in Fig. S4. The high background concentration of H_2SO_4 observed in the time series are likely due to solar radiation at high latitudes during the study period, as illustrated in Fig. S14. At low latitudes of our study, the diurnal minimum of H_2SO_4 was about 10^4 cm^{-3}. As we sailed towards higher latitudes and the year moved towards the June solstice, the sun remained above the horizon continuously. Consequently, the diurnal minimum of H_2SO_4 rose to above 10^5 cm^{-3}. Similar trends have been documented in previous studies (e.g., Beck et al. [14]).

We cannot be certain what fraction of our cluster signals arise from species charged via interaction with NO_3^- versus that which arises from ambient ionisation. Chamber studies have demonstrated that ionising radiation accelerates nucleation rates for species which form weakly-bound neutral clusters, such as H_2SO_4 and NH_3 [65]. However, for systems that form strongly bound neutral clusters—such as HIO_3, HIO_2, and H_2SO_4 [10], as well as H_2SO_4 and DMA [11] —ionising radiation has minimal impact on nucleation rates. Therefore, for our purposes, the distinction between neutral and charged clusters is largely inconsequential; what matters is their presence. Furthermore, our results quantitatively agree with the results of He et al. [10] under ion-pair-production rates similar to that in the Arctic atmosphere (Fig. 3b).

To evaluate the feasibility of neutral cluster detection, we built a kinetic model based on the Atmospheric Cluster Dynamics Code (ACDC) to simulate the birth-death equations, utilizing binding energies from refs [10] and [66]. The simulations indicate that under the observed atmospheric

conditions and formation rates, the neutral concentration of the inorganic trimer ($HIO_2 \cdot H_2SO_4 \cdot H_2SO_4$) would likely fall well below our instrumental LOD. Consequently, the observed signal for this specific cluster is likely sourced primarily from ambient natural ions or the fragmentation of larger clusters upon charging with NO_3^-. In contrast, the model suggests the neutral $C_2H_7N \cdot HIO_2 \cdot HIO_3$ cluster could theoretically exceed the LOD at approximately 1 pptv of DMA. Regardless of the charge state, the presence of these strongly bound clusters confirms the active chemical pathways, as ionising radiation has been shown to have minimal impact on the nucleation rates of these systems

4. Insignificant role of ammonia and HNO_3 in nucleation

Our results suggest that NH_3 does not play a significant role in the nucleation of new particles in our study. While certain regions of the Arctic contain important sources of NH_3, as suggested by Croft et al. (2016) [67] and relatively high concentrations of NH_3 (e.g., occasionally exceeding 100 pptv) were measured during a NETCARE cruise [68], vast areas of the Arctic have low NH_3 emissions (see Figure 2 in Croft et al. (2016)) [67]. Our results show NH_3 is below detection limit (~65pptv) throughout the campaign, as measured by the AiRRmonia instrument. This finding is consistent with modelling results by Croft et al. (2016) [67], who predicted low concentrations (<100 pptv) in the lower altitude Arctic. Our aerosol-phase AMS measurements also corroborate with this, showing extremely low NH_4^+ concentrations (0.03 ±0.02 ug m^{-3}). Additionally, as demonstrated by He et al. [10], sub-pptv levels of HIO_2 can stabilize H_2SO_4 as effectively as 500 pptv of NH_3, further suggesting a limited role of NH_3 in nucleation. These results indicate that the concentration of NH_3 is not sufficiently high to significantly influence particle formation, though we cannot completely rule out its participation in the NPF.

Several factors make it highly unlikely that HNO_3 plays a role in our study: First, the molecular mechanism by which HNO_3 initiates new particle formation is well understood, and under our ambient conditions (~273 K), we would not expect any contribution of HNO_3 to nucleation. The CLOUD chamber study in 2020 [69] observed significant nucleation only at much lower temperatures (257 K) and elevated concentrations of NH_3 (2×10^3 pptv) and HNO_3 (2×10^2 pptv), levels far more characteristic of a polluted megacity than the Arctic. Even under these elevated concentrations, no nucleation was observed at 278 K. A subsequent investigation in 2022 [70]likewise reported that multicomponent nucleation involving HNO_3, NH_3, and H_2SO_4 only becomes appreciable below 258 K; indeed, these authors note that "the multicomponent nucleation is quenched ($J_{1.7} < 0.01$ cm^{-3} s^{-1}) above 268 K." Consequently, HNO_3-driven nucleation is unlikely to contribute meaningfully under the relatively warmer temperatures (~273 K) recorded in our measurements.

Second, the Arctic atmosphere is characterized by very low levels of NO_x, with an averaged value of 0.067 ppbv in our study region. This scarcity of nitrogen oxides, combined with the clean nature of the Arctic air, makes the presence of sufficient HNO_3 to drive nucleation highly unlikely. Furthermore, AMS data confirms that there is very little nitrate in the particle phase relative to the sulfate and organic mass. Similarly, we infer from the AMS data that ammonia concentrations remain very low in this environment (consistent with our gas-phase ammonia measurements being consistently below LOD by AiRRmonia instrument). Given that nitrate-based nucleation typically requires large amounts of both HNO_3 and NH_3, their scarcity in the Arctic atmosphere makes it highly unlikely that nitrate-driven nucleation could be a significant contributor to the new particle formation events we observed.

Third, we note that Chamba et al. [71] offered limited direct evidence for nitrate-based nucleation, as they relied primarily on the detection of nitrate ions in a chamber experiment that was half filled with seawater and half filled with particle-free ambient marine air. They also measured urea and C_2 and C_4 amines. While the authors interpret the presence of nitrate ions to indicate nitric acid, they also acknowledge that nitric acid does not form stable clusters with amines or urea, undercutting the argument for a HNO_3-based pathway.

In conclusion, we found no evidence of HNO_3 involved in nucleation during our study.

5. Interpretation of PMF factor for OOMs

In this study, PMF was utilized to identify distinct factors (five factors, Extended Data Fig. 8, Fig. S7) within our NO_3^- CIMS data, as detailed below:

Factor 1 is anthropogenic in origin (dubbed "Anthro"), and has the largest signals at midday on 3rd and 4th June (Extended Data Fig. 8), when air masses originated from populated coast of Greenland. This anthropogenic OOM source has the highest mean volatility among all five factors, being dominated by C_8 and C_9 compounds, which are likely the oxidation products of anthropogenic VOCs [72]. The peaks with the highest signals in this factor are $C_8H_{12}O_5\ NO_3^-$, $C_9H_{17}NO_8\ NO_3^-$, and $C_9H_{16}O_6\ NO_3^-$.

Factor 2 is dominated by I-OOMs, referred to as "I-OOM" factor. This factor correlates well with iodic acid (Extended Data Fig. 7) with midday peaks and is highest on the 1st and 2nd June. The I-OOMs in this factor are dominated by C_8 and C_{10} compounds. The largest peaks in this factor are $C_7H_6O_7\ NO_3^-$, $C_8H_{13}NO_7\ NO_3^-$, $C_9H_{15}NO_6\ NO_3^-$, and the largest iodine-containing peaks are $C_8H_{11}IO_5\ NO_3^-$, $C_{10}H_{15}IO_9\ NO_3^-$, and $C_8H_{12}INO_8\ NO_3^-$ (Table S3). Some of the peaks in this factor

do not contain iodine. This could be related to the reaction pathway that forms them involves iodine but does not necessarily impart iodine into the resultant OOM, and/or some I-OOMs lost the iodine molecule later.

We propose a hypothesis for I-OOM formation. The diurnal profiles of these compounds indicate daytime formation, implicating photochemical processes. Given that reactions between iodine-containing species and VOCs are significantly slower than those involving other halogen radicals such as Br and Cl [73], direct reactions between VOCs and iodine radicals are unlikely pathways for I-OOM production, especially as we do not anticipate substantially higher iodine radical concentrations relative to chlorine and bromine, as indicated by their relative oxidation product concentrations during recent Arctic campaigns [74,75]. Molecular composition offers further insights: for instance, $C_{10}H_{15}IO_{3-9}$ matches well-characterized OOMs ($C_{10}H_{16}O_{3-9}$) formed via monoterpene oxidation in iodine-free environments [22], differing only by the substitution of a hydrogen atom with iodine. Typical OOM formation proceeds through autoxidation, wherein the radical chain is terminated either by radical–radical collisions or propagated via self-reactions. These terminating radicals are often OH, HO_2, or NO. In the underexplored marine atmosphere, we hypothesize that termination may involve iodine-containing radicals such as IO or organic iodine species (CxHyI). Arctic observations report IO levels of ~3 pptv [76], sufficient to intercept a significant fraction of autoxidation reactions and yield the observed I-OOMs, as these concentrations are comparable with OH and HO_2 concentrations in the Arctic [77]. Hence, I-OOM formation likely adheres to established autoxidation mechanisms, diverging in the identity of the terminating radical. Further laboratory validation is warranted to substantiate this mechanism.

Factor 3 and 4, referred to as "Monoterpene-rich" and "Aldehyde-rich" factors, contain minimal amounts of iodine. The two factors are distinct, as supported by the distinct mass spectra and time series (Extended Data Fig. 8, Fig. S7), although both factors exhibit midday/afternoon peaks. Of the 50 largest peaks in each spectrum (accounting for 70% of the concentration for these factors), only 12 are found in common between both factors. These shared peaks account for 22% of the concentration of the monoterpene-rich factor and 11% of the concentration of the Aldehyde-rich factor. That is to say, these factors are dominated by different sets of OOMs.

The chemical link between aldehydes and our aldehyde-rich factor is established by the clear overlap in carbon number distribution between these measured precursors and their oxidation products; the aldehyde-rich PMF factor is dominated by C_9 to C_{15} OOMs, a range that is fully consistent with the oxidation of the aldehydes we measured (see Fig. S8). Furthermore, the time series of this PMF factor is highest on days when high concentrations of gas-phase aldehydes were observed in airmasses originating from the marginal sea ice zone, strengthening the link between the precursors, their sources, and their ultimate contribution to particle growth. In the aldehyde-rich factor, we observe a higher abundance of compounds with >10 carbon atoms and a distinct volatility distribution (Fig. S7). These are likely due to distinct OOM sources. Furthermore, monoterpenes and aldehydes have different reactivities towards OH and O_3, as well as different OOMs yields. Many of the most common monoterpenes are highly reactive [78] and readily undergo autoxidation [22]. Aldehydes are generally less reactive with OH and much less reactive with O_3 [78,79], and autoxidation [80]. OOM yields of aldehydes are therefore likely lower, depending on temperature and oxidants [80]. This likely explains the similar concentrations of the factors, despite different concentrations of precursors.

Factor 3 is dominated by C_{10} and some C_9 compounds. The dominant species in this factor are $C_{10}H_{16}O_{10}$ NO_3^-, $C_9H_{15}NO_7$ NO_3^-, $C_9H_{15}NO_6$ NO_3^- (Table S4). These compounds are common oxidation products of monoterpene oxidation [22]. We tentatively assign this factor to be primarily related to monoterpene oxidation. Although mixing ratio of monoterpene is low during the campaign (Extended Data Fig. 1d), it produced high signals during about a third of the observation days (Extended Data Fig. 8).

Factor 4 has a substantial contribution from compounds ranging from C_9 to C_{15}, which we attribute to the oxidation of aldehydes. The dominant species in this factor are $C_9H_{15}NO_9$ NO_3^-, $C_{11}H_{21}NO_8$ NO_3^-, and $C_9H_{17}NO_8$ NO_3^-. We attribute this factor to the atmospheric oxidation of aldehydes. Aldehydes readily produce OOMs, as shown in a laboratory study of decanal autooxidation [80]. We measured high gas-phase concentrations of aldehydes in the C_9 to C_{13} range in PTR-MS (Fig. 15). The significant concentrations of aldehydes on days when air masses flow over the sea ice edge suggests that the aldehyde precursors are abundant at the melting sea ice edge. Notably, this factor exhibits the lowest mean volatility.

Factor 5 is identified as a noise factor, exhibiting no diel variation throughout the entire campaign, as illustrated in Extended Data Fig. 8. Similar noise factors have also been recognized in other studies [14,61]. For example, this factor includes species with negative mass defects such as a prominent peak at 362 Th (e.g., $C_5F_{10}H_2O_3$ NO_3^-). The factor lacked the homologous series characteristic of OOMs (e.g., $C_{10}H_{16}O_{2-8}$), indicating that these species were likely contaminants rather than atmospheric OOMs.

6. Uncertainties of particle growth modelling

Our modelled growth rates do not match the measured GRs from the PNSD for every event. There are several assumptions made, and a few uncertainties we cannot account for in the model. Most importantly, box models (including this dynamic aerosol growth model) presume that there is no dilution or advection of air masses to/from the measurement point. This does not hold true for the real environment, and explains some of the differences, especially on measurement days where air masses changed direction frequently, or were particularly turbulent (Extended Data Figs. 1 & 10). At diameters larger than 25 nm, a role of solute phase chemistry with multiphase processes including salt formation, oligomerization, and other heterogeneous reactions in particle growth becomes possible, which is not dealt with in the model [81,82]. We neglect coagulation growth, although this will be a relatively small contributor to overall growth. We also presume that particle-phase reactions cause no change in partitioning, although these reactions are relatively fast and likely result in a net decrease in evaporation rates of condensed phase OOMs [83].

While our model captures the primary drivers of growth, we acknowledge that complex multiphase chemistry such as the heterogeneous reactions between organics and iodine-oxide seeds [82] may contribute to growth during periods of model underestimation. However, the precise role of such reactions in organic-dominated, multi-component Arctic particles remains an open question [84] and warrants further targeted study.

Condensational growth is a competition between the rates of condensation and evaporation. The abundance of gases largely governs the former, while the volatilities of the involved species are more important in governing the latter [84]. With the NO_3^- CIMS, we solely measure formulae, rather than structures, and we assume certain functionalities and their effect on saturation vapor concentration [22]. Different methods come up with different values for saturation vapor

concentration [85], which is another source of uncertainty. The NO_3^- CIMS lacks sensitivity to certain OOMs, such as those with only one hydrogen bond donor group [22], and our OOM concentrations are therefore a lower limit on the real OOMs concentration in the environment. Specifically, it has a well-known lower sensitivity to less oxygenated, more volatile species like semi-volatile organic compounds (SVOCs), which were therefore not constrained by our measurements. Despite those, our modelled condensation rates match the evolution of the PNSD within ~50%, and the resultant mass concentrations match the sulfate to organic ratio we measure in the AMS. We therefore capture the key processes relevant to particle growth.

7. VOC composition

Recent publications have highlighted the fragmentation of aldehydes to produce interfering ions in the PTR-MS ionization system [86-88], and therefore, the 2D-GC-MS was applied to validate the results from the PTR-MS. The concentrations of aldehydes up to C_{13} were measured by both PTR-MS and 2D-GC-MS. The highest concentrations with carbon number larger than 4 are C_7, C_9, and C_{11} in the PTR-MS, and C_9, C_{12} and C_{13} in the 2D-GC-MS (Fig. S8). The fragmentation patterns of individual aldehydes are complex [86]. As we have multiple aldehydes as part of a mixture of many VOCs, working out the individual aldehyde concentrations is not possible from our data. For this reason, we report the total concentration of aldehyde fragments instead.

We analyzed 16 24-hour resolution diffusion tubes. However, we found signals of naphthalene, an anthropogenic tracer, in our samples. This is likely because VOCs from ship stack emissions were collected on the tubes alongside ambient VOCs . Because of the low limits of detection of the 2D-GC-MS, even low concentrations of naphthalene, including from short spikes related to ship stack emissions, can be detected. We therefore report the distribution of VOCs from the tube collected

from 11 am on 26th to 11:00 on 27th May 2022 (Fig. S8) with the highest nonanal:naphthalene ratio. The absence of BC spikes during this sampling period (Fig. S2c) suggests minimal impacts from the ship stack emissions. This indicates that the distribution of VOCs in this tube is more representative of the real atmosphere.

Mean mixing ratio of aldehydes and aldehyde fragments (see Table S4) measured by PTR-MS (6.8 pptv) was substantially higher than that for monoterpenes (0.45 pptv) across the entire campaign. Similarly, hourly peak concentration of aldehydes and their fragments in the PTR-MS was also substantially higher than monoterpenes (420 pptv versus 3 pptv). The high correlation coefficient between $C_9H_{19}O^+$ (likely nonanal) and $C_5H_9^+$, a common aldehyde fragment [86] ($R^2 = 0.94$, Fig. S8), suggests that most $C_5H_9^+$ is a fragment of aldehydes rather than isoprene. Aldehydes can be produced from the ozonolysis of fatty acids at the surface microlayer [89-91], making these fatty acids likely precursor candidates for our measured aldehydes.

Strong signals from toluene and xylene fragments (Fig. S2d) were detected during "ship emission days". We only detected two small spikes of these fragments (Fig. S2d) on 7-8th June in PTR-MS, and their background concentrations did not change during this period, suggesting no detectable influence of ship stack emissions on background VOC concentrations measured by the PTR-MS on these days.

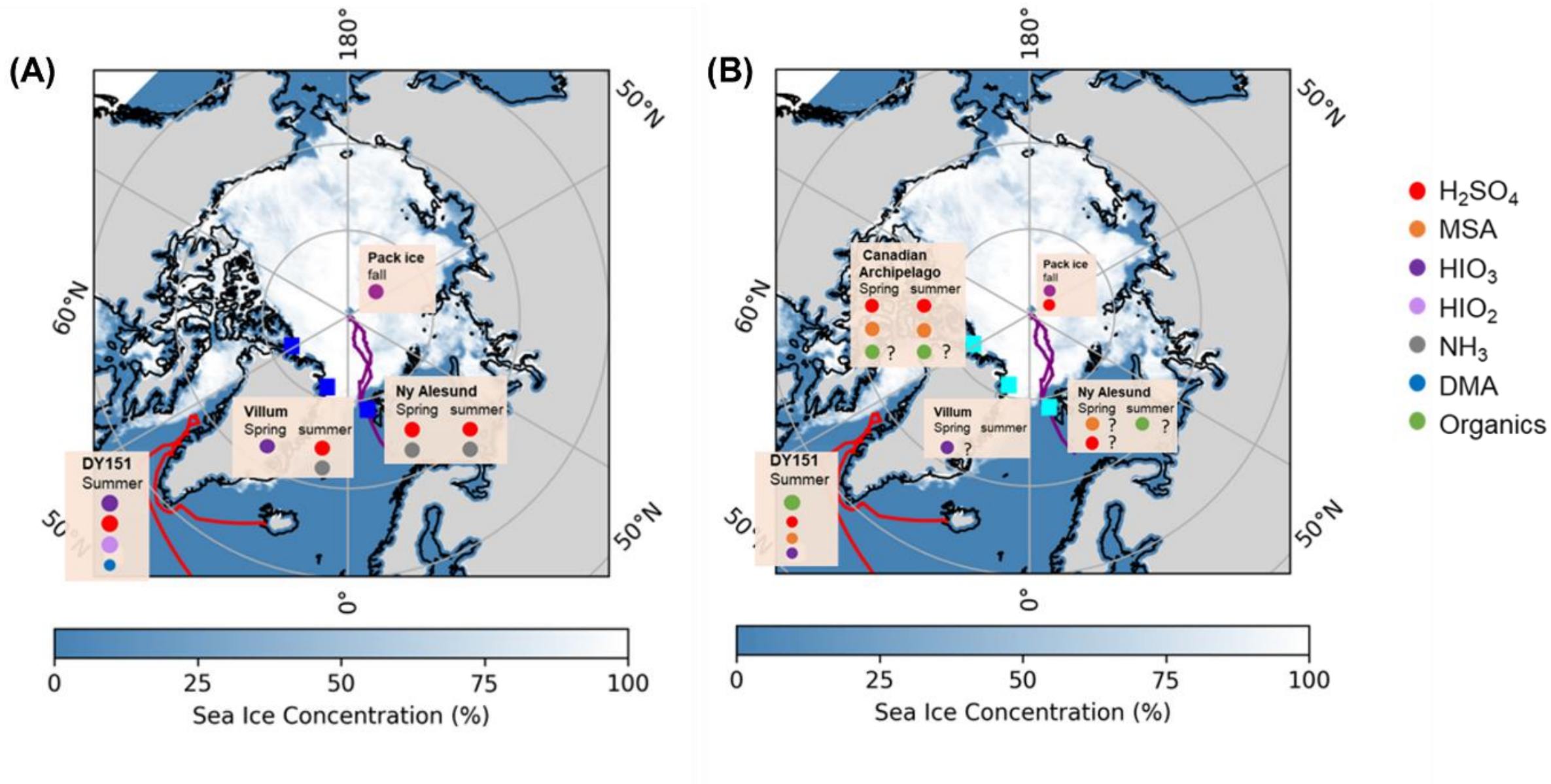

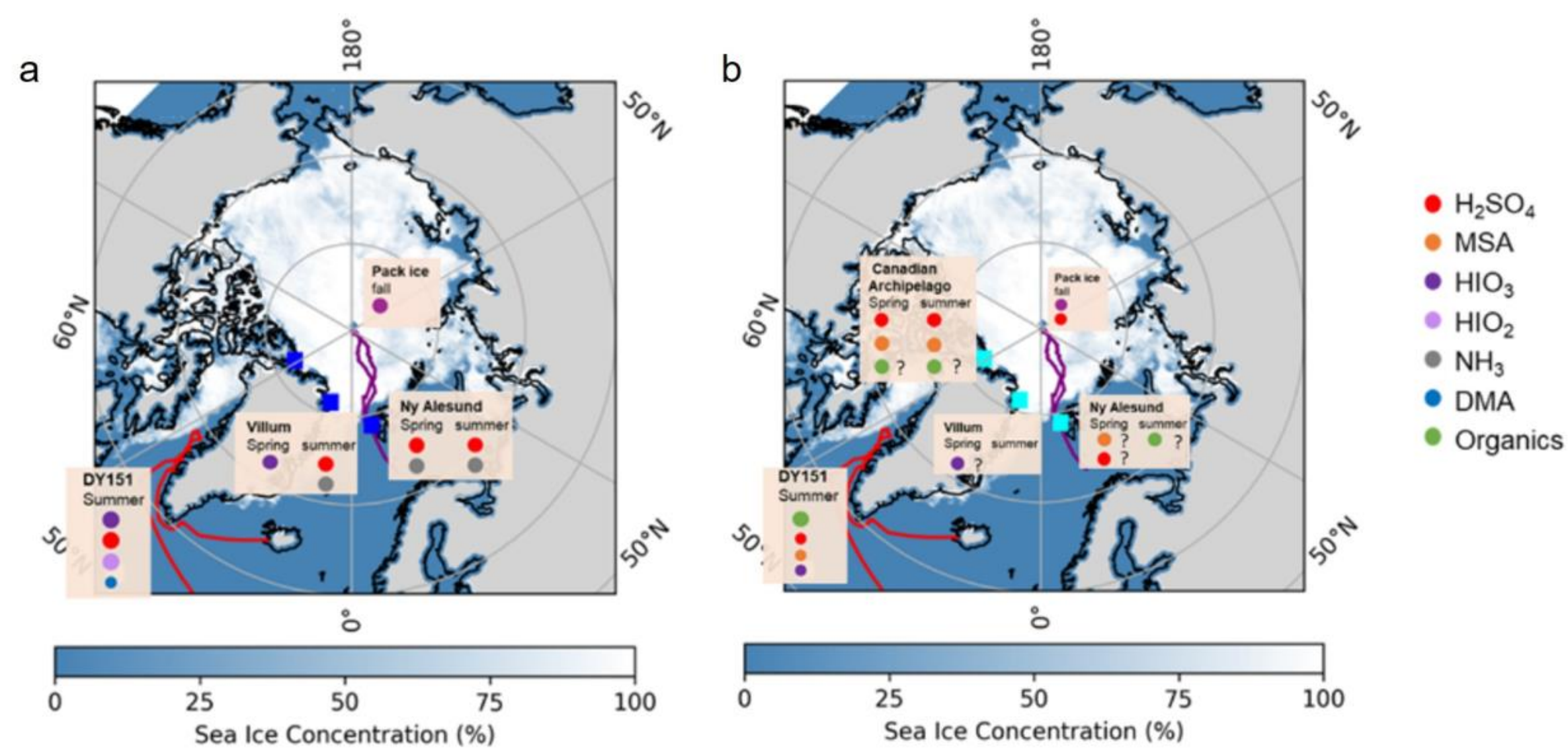

Figure S1: Nucleation and growth Mechanisms previously identified in the Arctic. **(a)** Nucleation mechanism in the Arctic. Pack Ice Area: Baccarini et al.[13]. Villum and Ny-Ålesund: Based on findings from Beck et al.[14]. This underscores gaps in our understanding of nucleation mechanisms and their regional significance in the Arctic. The smaller size of the symbols represents the potentially smaller roles of nucleation mechanisms in certain regions. **(b)** Particle

Growth Mechanism identified in Arctic Regions. The question marks behind "organics of Canadian Archipelago" indicate the absence of molecular information about organics to explain the detailed particle growth. Similarly, the question marks for Ny-Ålesund and Villum represent the lack of quantitative evidence to elucidate particle growth mechanisms. This figure highlights gaps in our understanding of specific particle growth mechanisms in these regions. Canadian Archipelago: Referenced from studies by Chang et al. [92], Ghahreman et al.[93] , Leaitch et al. [94], Sharma et al. [95], and Tremblay et al. [96]. Pack Ice Area: Cited from Baccarini et al. [13]: "Iodic acid largely explains the growth of ultrafine particles (UFP) in most events". Villum and Ny-Ålesund: Based on findings from Beck et al. [14]. They suggested that "concentration (of iodic acid) was insufficient to grow nucleated particles to CCN sizes in Villum", and "growth mainly by SA and MSA condensation during springtime and highly oxygenated organic molecules during summertime". We note that a non-standard approach was used to model particle growth Beck et al. [14], as no OOMs were identified in the papers, and the results exhibit substantial uncertainties.

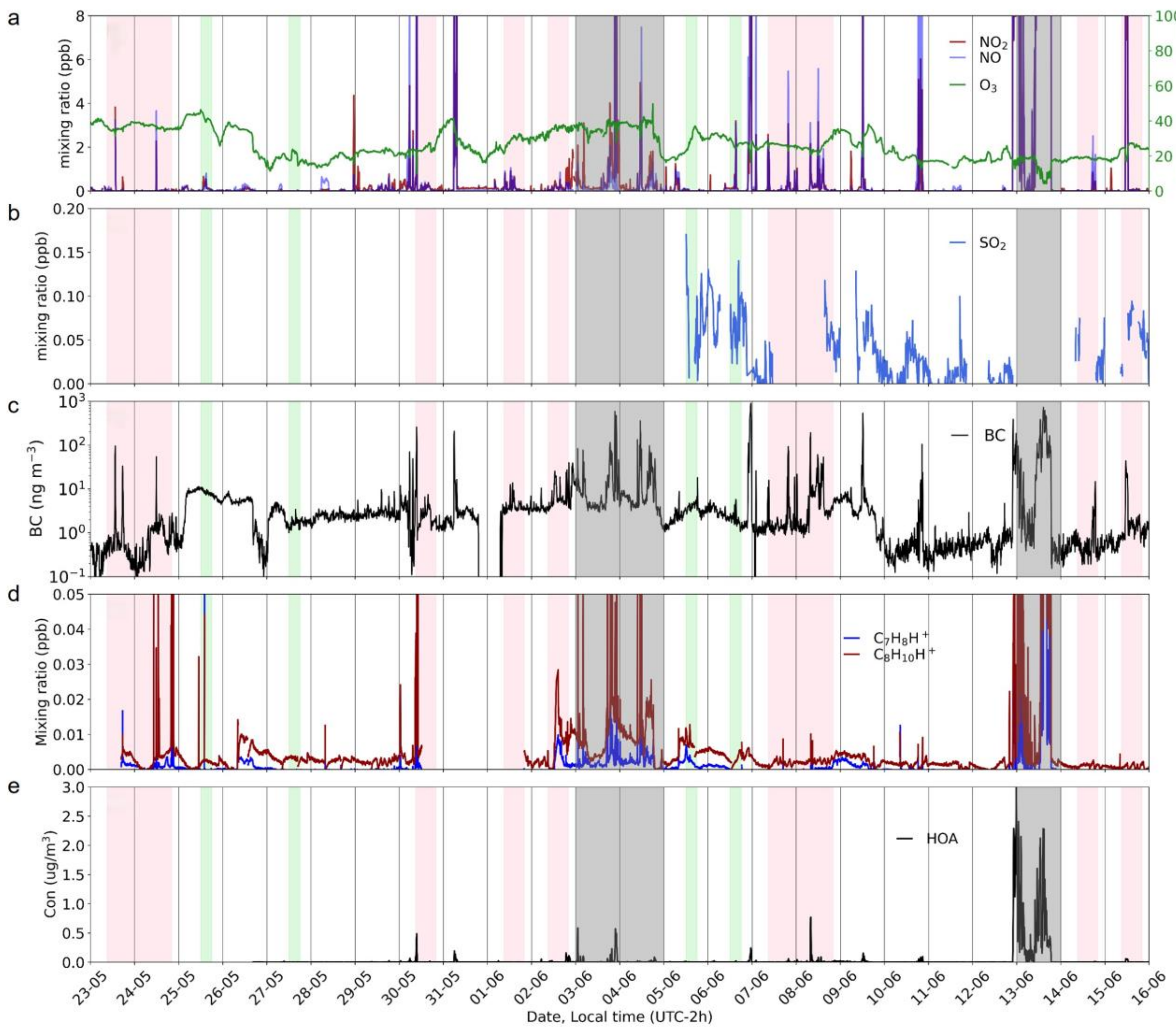

Figure S2: Time series of anthropogenic tracers. (**a**) Mixing ratios of nitrogen dioxide (NO_2), nitric oxide (NO), and ozone (O_3). (**b**) Mixing ratios of SO_2 and CO. (**c**) Concentrations of BC. (**d**) Signals for volatile organic compounds including ions $C_7H_8H^+$ (attributed to toluene) and $C_8H_{10}H^+$ (attributed to xylene) from PTR-MS measurements. (e) Concentrations of AMS PMF HOA factor. Pink shaded areas across all panels indicate NPF event days, and green shaded areas indicate burst event days. Gray shaded areas indicate ship emission days. Blank periods in some of the figures, e.g., Fig. S2b and **e**, represent missing data (e.g., instrument breakdown).

1000

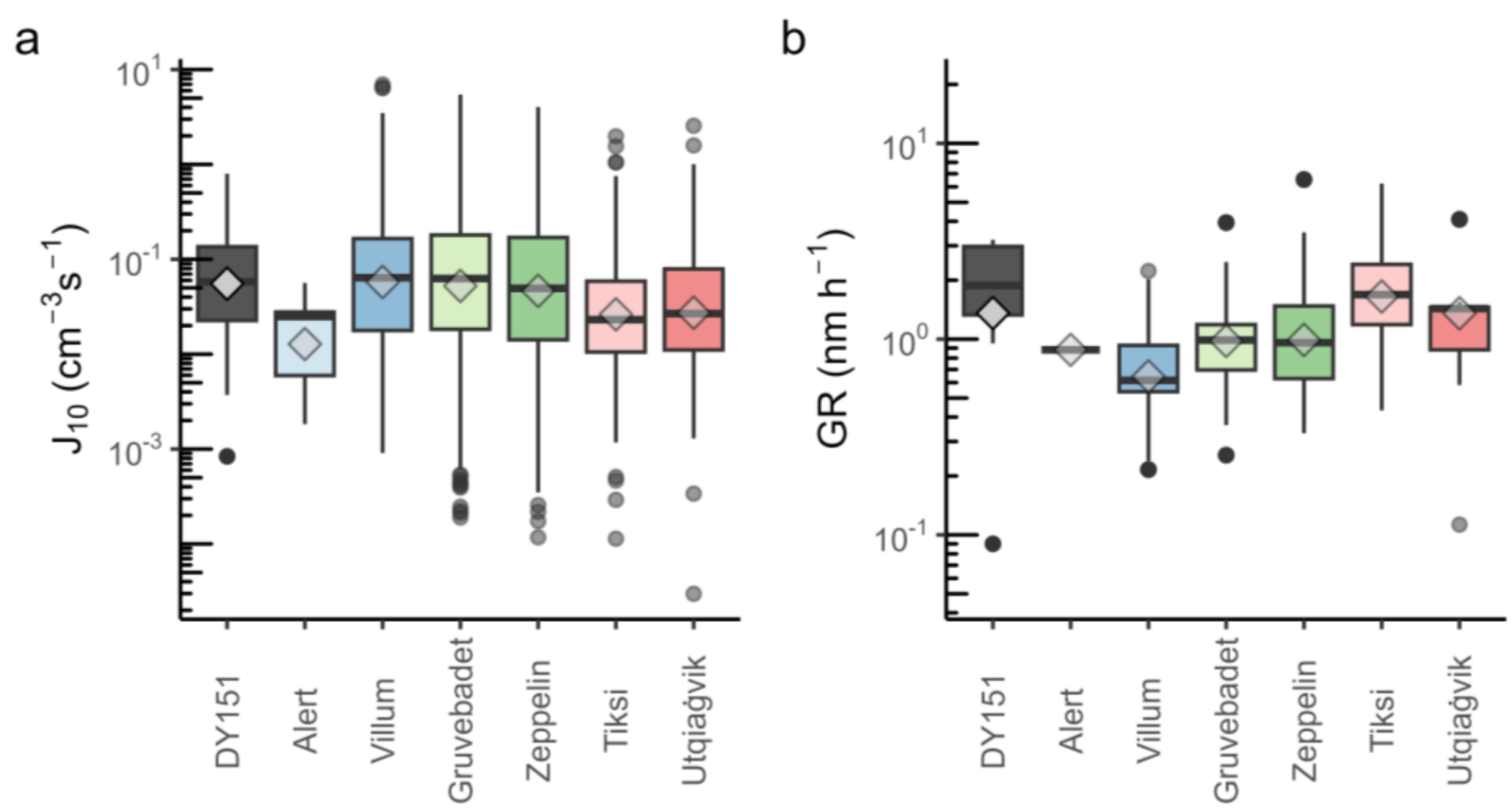

Figure S3: Comparison of Formation and Growth Rates. DY151 alongside other Arctic research stations. (**a**) Formation Rate; (**b**) Growth Rate. J_{10} denotes the formation rate of particles at 10 nm utilizing the 10-25 nm size range, while GR is computed for particles exceeding 10 nm. Long-term datasets from key Arctic stations—Alert GAW Observatory (Alert), Utqiaġvik (previously Barrow), Gruvebadet, Villum Research Station (Villum), Tiksi, and Zeppelin Mountain (Zeppelin) are depicted in grey box plots (source: Brean et al. [97]). Data for May and June exclusively plotted for all available years. DY151 NPF formation and growth rates are illustrated in dark grey box plots. In each plot, the black line indicates the median value, while the box represents the interquartile range (25th to 75th percentiles). The whiskers extend to data points within 1.5 times the interquartile range, and grey dots show outliers beyond this range. All data points for J_{10} are selected between 10 a.m. and 3 p.m. (UTC time) each day to indicate the nucleation periods. Most NPF events for Tiksi occurred under polluted continental airmasses and thus are not representative of the Arctic marine environment. Sample sizes are: DY151 n=40, Alert n=20, Villum n=192, Gruvebadet n=687, Zeppelin n=1253, Tiksi n=436, Utqiaġvik n=76 hourly

measurements for panel (**a**), and DY151 n=7, Alert n=1, Villum n=24, Gruvebadet n=61, Zeppelin n=115, Tiksi n=39, Utqiaġvik n=12 days for panel (**b**).

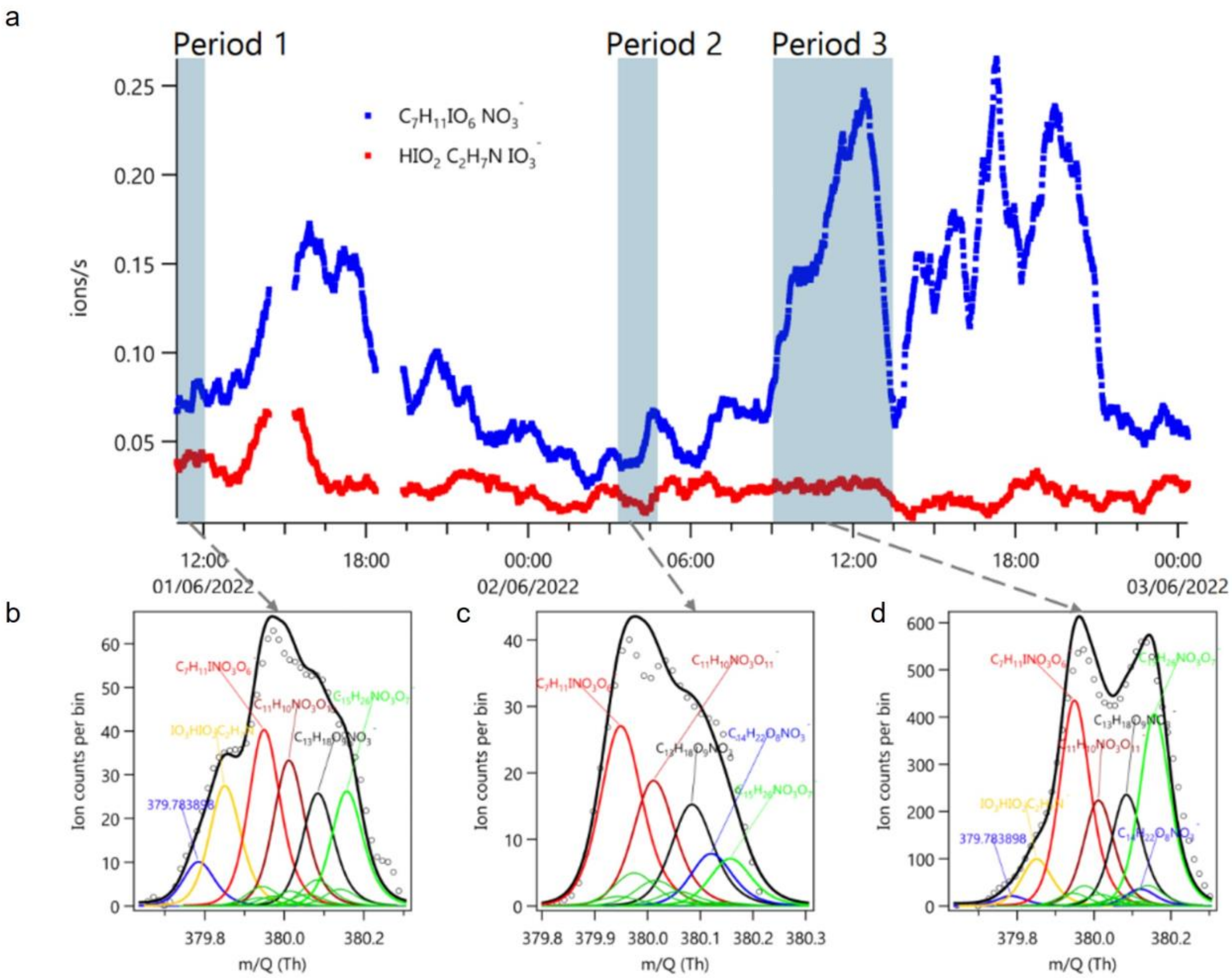
a
b
c
d
Period 1
Period 2
Period 3
$C_7H_{11}IO_6\ NO_3^-$
$HIO_2\ C_2H_7N\ IO_3^-$
ions/s
0.05
0.10
0.15
0.20
0.25
12:00
01/06/2022
18:00
00:00
02/06/2022
06:00
12:00
18:00
00:00
03/06/2022
Ion counts per bin
m/Q (Th)
379.8
380.0
380.2
379.9
380.1
380.3
$C_7H_{11}INO_3O_6$
$C_{11}H_{10}NO_3O_{11}$
$C_{13}H_{18}O_9NO_3^-$
$C_{14}H_{22}O_8NO_3^-$
379.783898

Figure S4: Identification of C_2H_7N-HIO_2-IO_3^-cluster and examples of adjacent peak $C_7H_{11}IO_6NO_3^-$ interference in the C_2H_7N-HIO_2-IO_3^-cluster. (a) Time-series of the C_2H_7N-HIO_2-IO_3^- cluster (red line), the adjacent peak (blue line) on 1st and 2nd June, the days with the greatest IO_3^- signal. (b) Peak fitting for the selected Period 1, where the ratio of the cluster: adjacent peak ions intensity was the highest during the period shown above; this allows a firm identification of C_2H_7N-HIO_2-IO_3^- cluster (as a peak). (c) Peak fitting for the selected Period 2 during midnight which C_2H_7N-HIO_2-IO_3^- peak is not identifiable. (d) Peak fitting for selected Period 3 on 2nd June where the ratio of the cluster versus it's adjacent peak is increasing, and the cluster: adjacent peak ion intensity is relatively low.

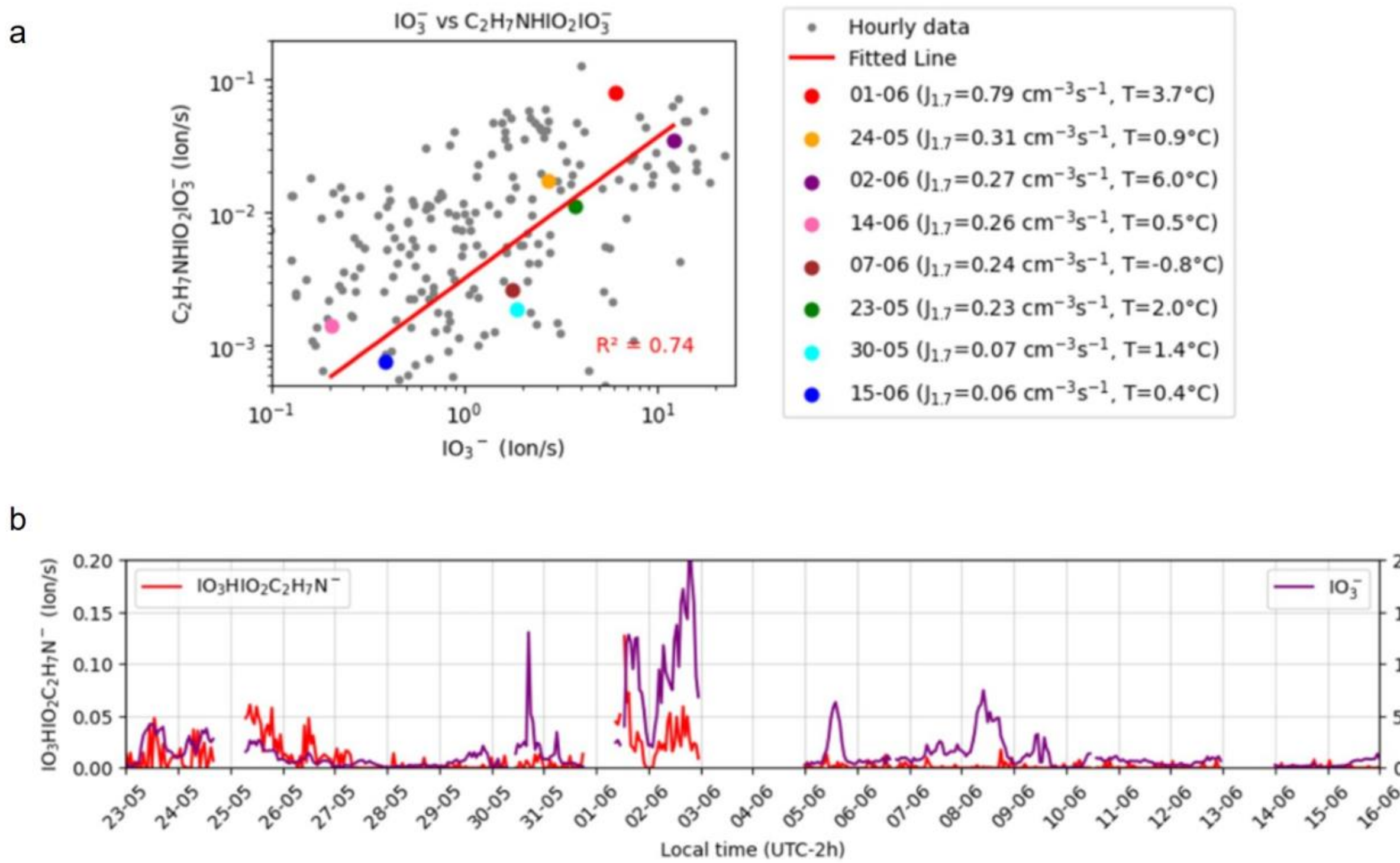

Figure S5: Raw ion intensity of IO_3^- and C_2H_7N-HIO_2-IO_3^-. (a) correlation relationship between ions of IO_3^- and C_2H_7N-HIO_2-IO_3^-. Grey data points represent hourly averages. Larger colored data points are mean ion counts during nucleation period for each NPF or burst event. (b) Time-series of IO_3^- and C_2H_7N-HIO_2-IO_3^- . Data points from ship emission periods have been filtered out, leaving gaps in the plot.

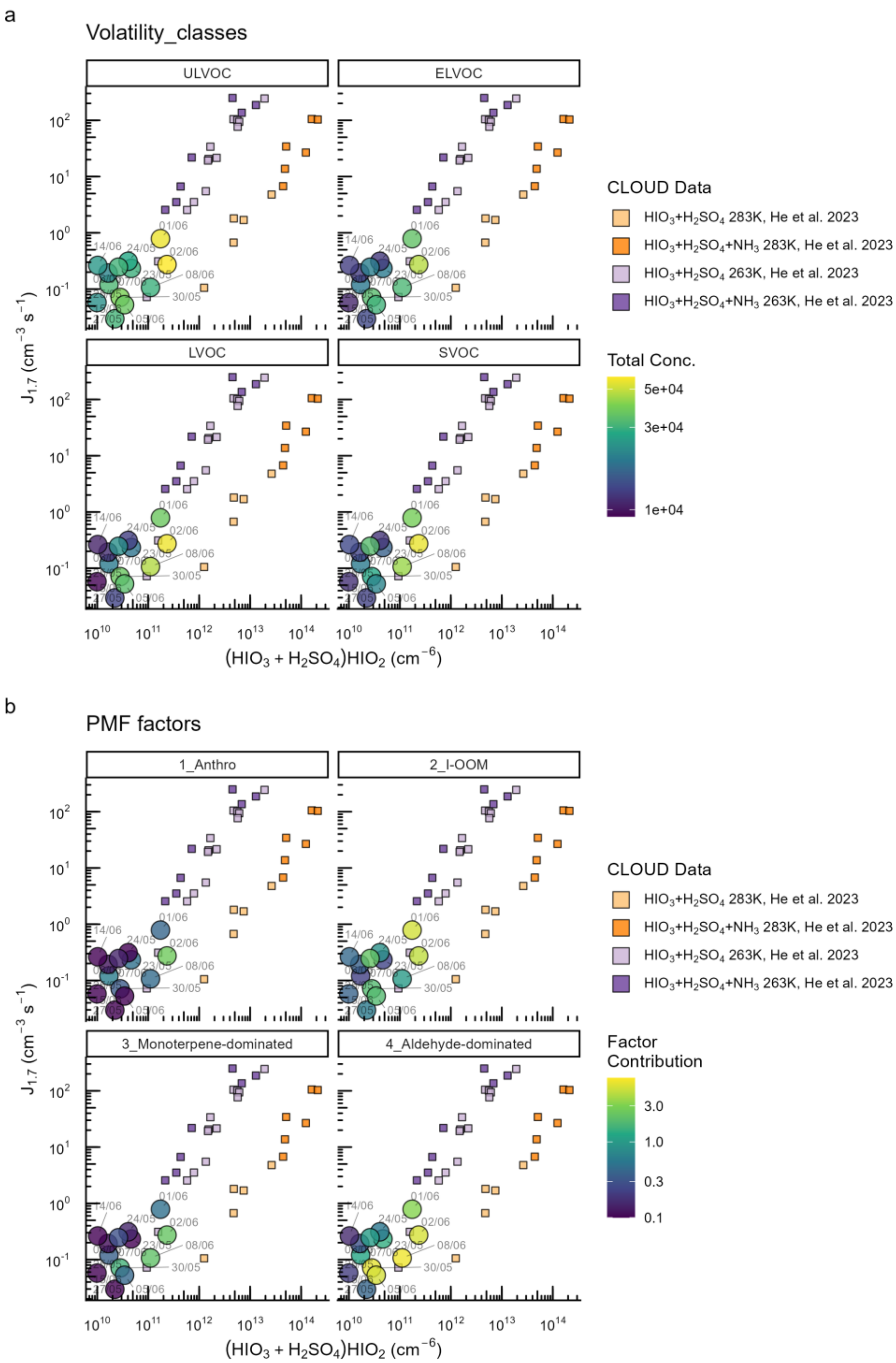

Figure S6: As Figure 3b, coloured by (a) different volatility classes of OOMs & I-OOMs, and (b) different PMF factors. We see no relationship between the enhancement to NPF relative to He et al. (2023)[10] and our observed OOMs & I-OOMs.

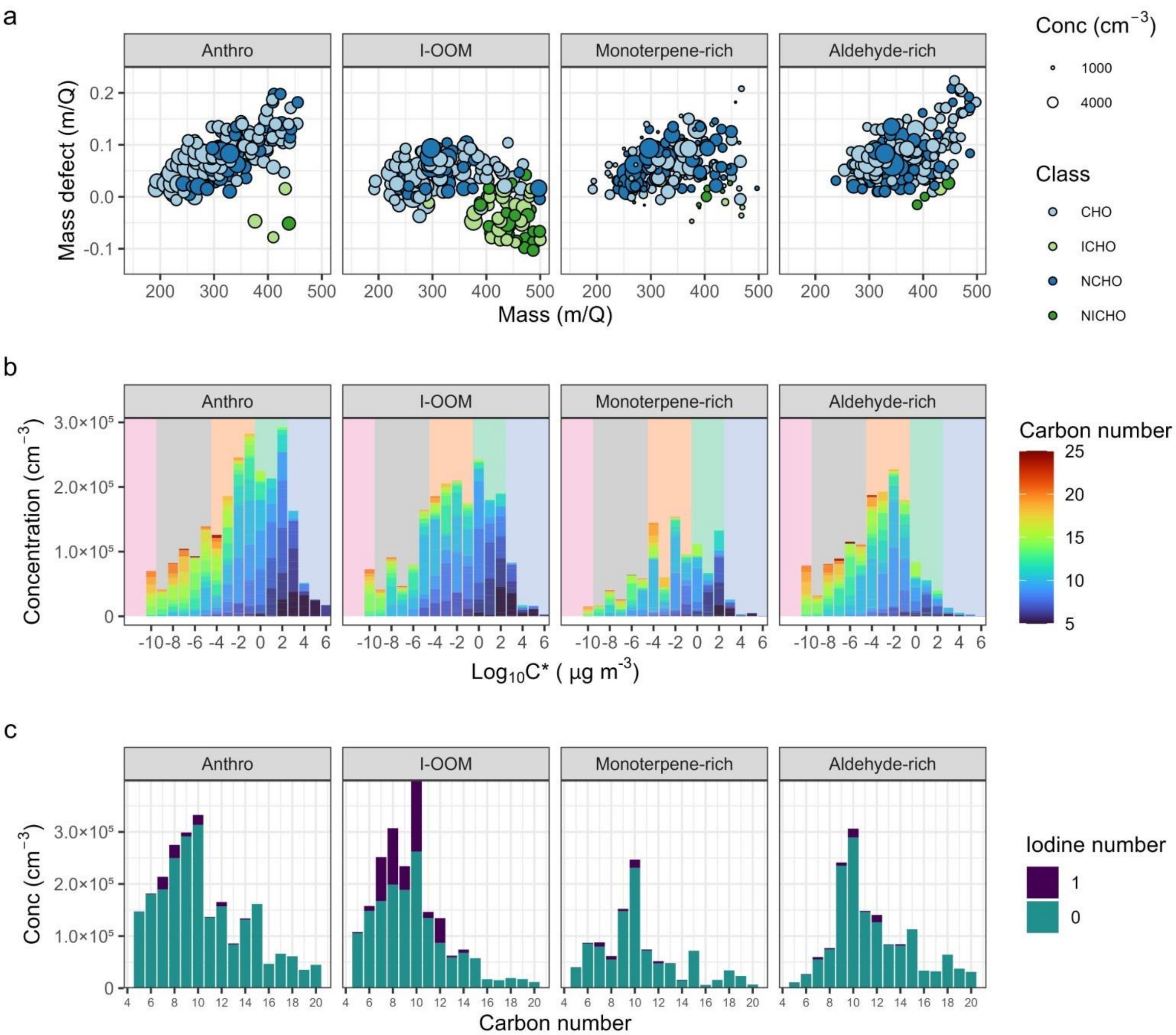

Figure S7: Characteristics of OOM sources. Output from PMF analysis of NO_3^- CIMS data showing: (a) The mass defect for each of the four factors, colored by the family (CHO corresponding to molecules that solely contain carbon, hydrogen, and oxygen; CHONI corresponding to those with carbon, hydrogen, oxygen, nitrogen, and iodine, and so on). Only the top 25% of signals per factor are shown. (b) The volatility distribution of each factor, colored by carbon number. The coloured regions correspond to (pink) ULVOC, (grey) ELVOC, (yellow) LVOC, (green) SVOC, and (blue) IVOC. (c) The average signal per carbon number in each factor, colored by the number of iodine molecules. The noise factor was not included here.

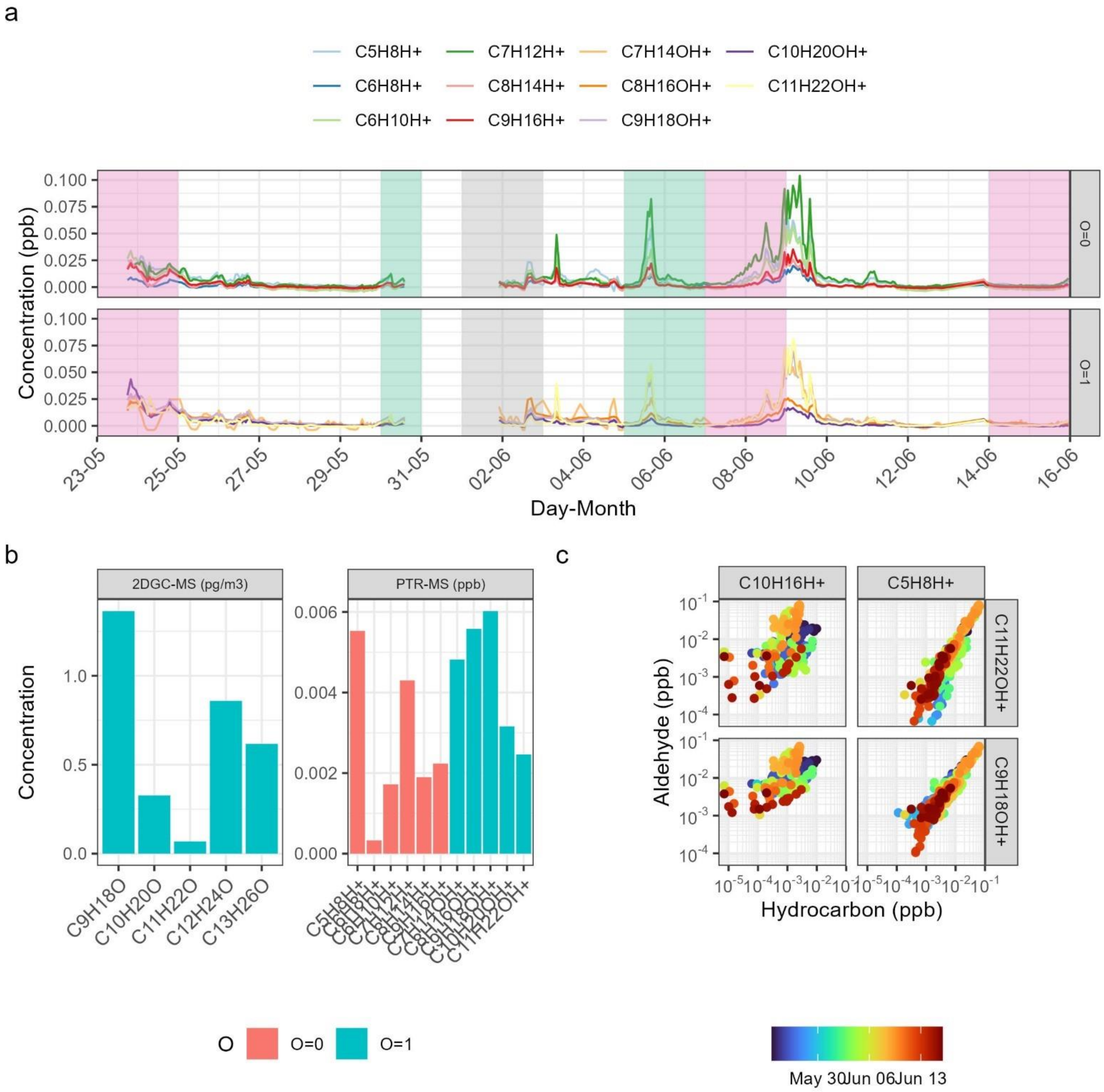
a
C5H8H+
C7H12H+
C7H14OH+
C10H20OH+
C6H8H+
C8H14H+
C8H16OH+
C11H22OH+
C6H10H+
C9H16H+
C9H18OH+
Concentration (ppb)
O=0
O=1
Day-Month
b
2DGC-MS (pg/m3)
PTR-MS (ppb)
Concentration
C9H18O
C10H20O
C11H22O
C12H24O
C13H26O
O
O=0
O=1
c
C10H16H+
C5H8H+
C11H22OH+
C9H18OH+
Aldehyde (ppb)
Hydrocarbon (ppb)
May 30
Jun 06
Jun 13

Figure S8: Aldehydes and their fragments based on the PTR-MS and comparison with 2D-GC-MS. (a) Time series of the oxygen and non-oxygen containing aldehyde fragments summed in Figure 4. (b) Average concentration of these species from 11 am on 26th to 11:00 on 27th May 2022 from the PTR-MS and 2D-GC-MS. This corresponds to the tube least influenced by ship stack, as determined from the aldehyde: naphthalene ratio, and (c) the correlation between C_9 and C_{11} aldehydes, and $C_5H_8H^+$ (commonly attributed to isoprene) and $C_{10}H_{16}H^+$ (commonly attributed to monoterpenes) in the PTR-MS. Ship emissions have been filtered out from the PTR-MS.

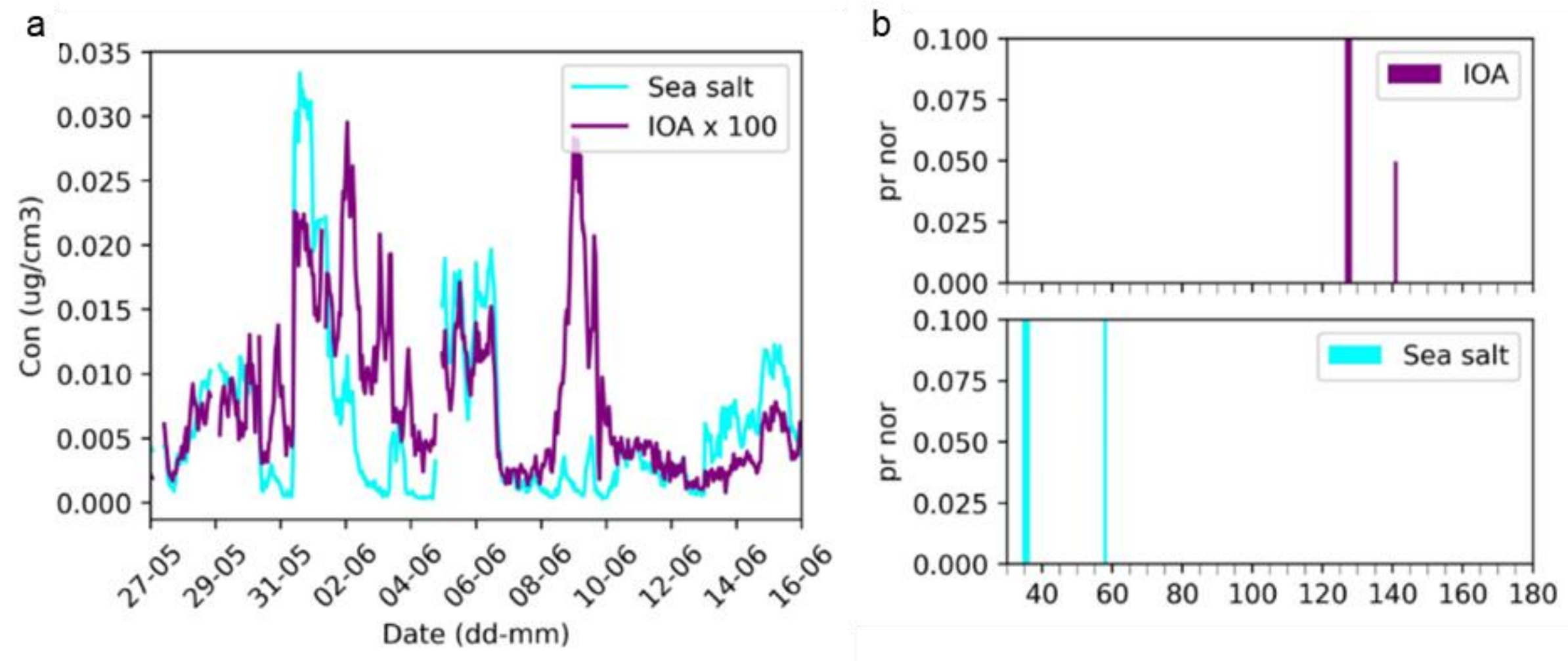

Figure S9: AMS PMF derived sea salt OA and iodine OA (IOA). (a) Time series of two factors: IOA, and sea salt OA. (b) Mass spectrum profiles for the two factors.

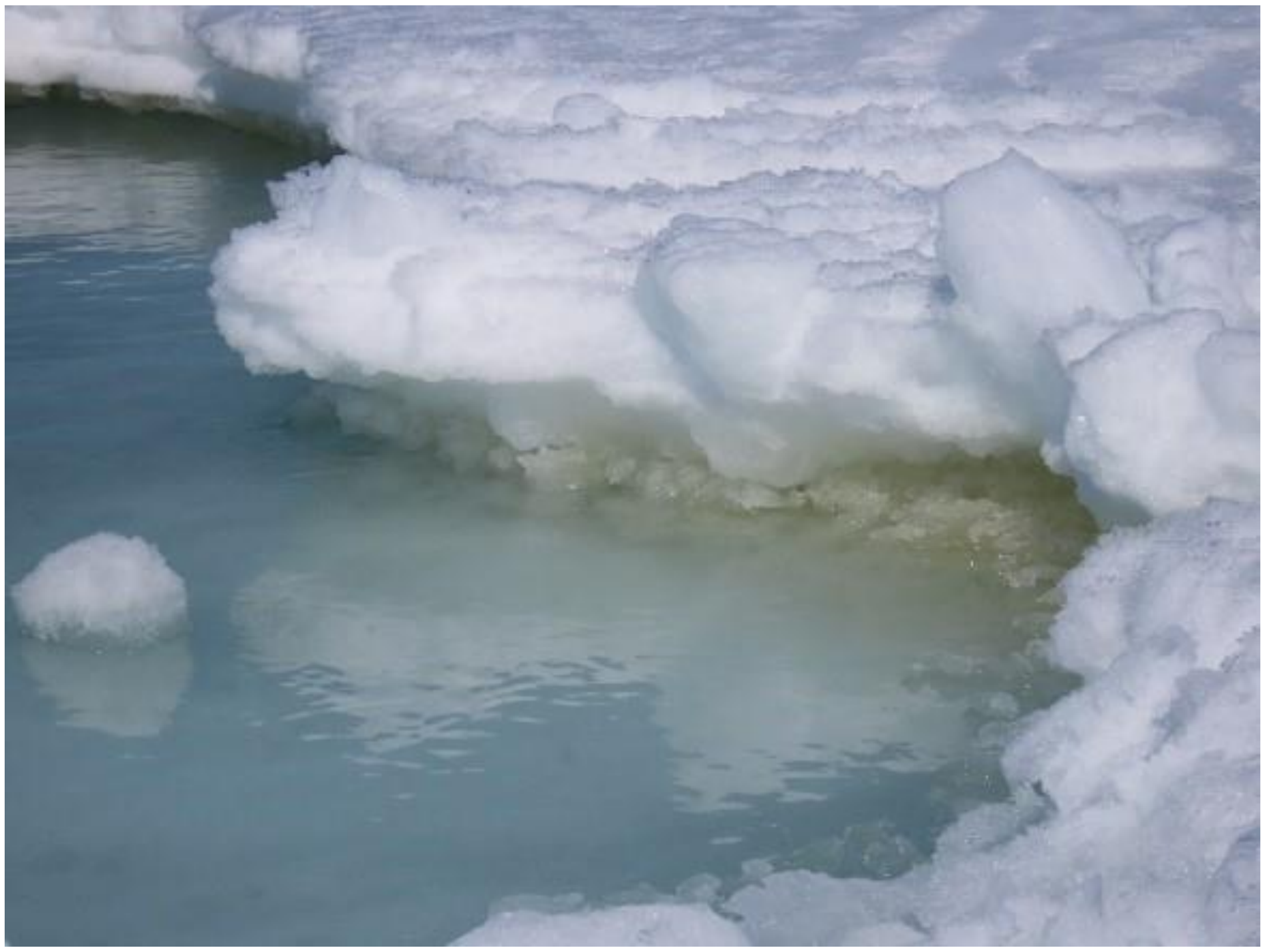

Figure S10: Algae underneath the melting sea ice.

1068 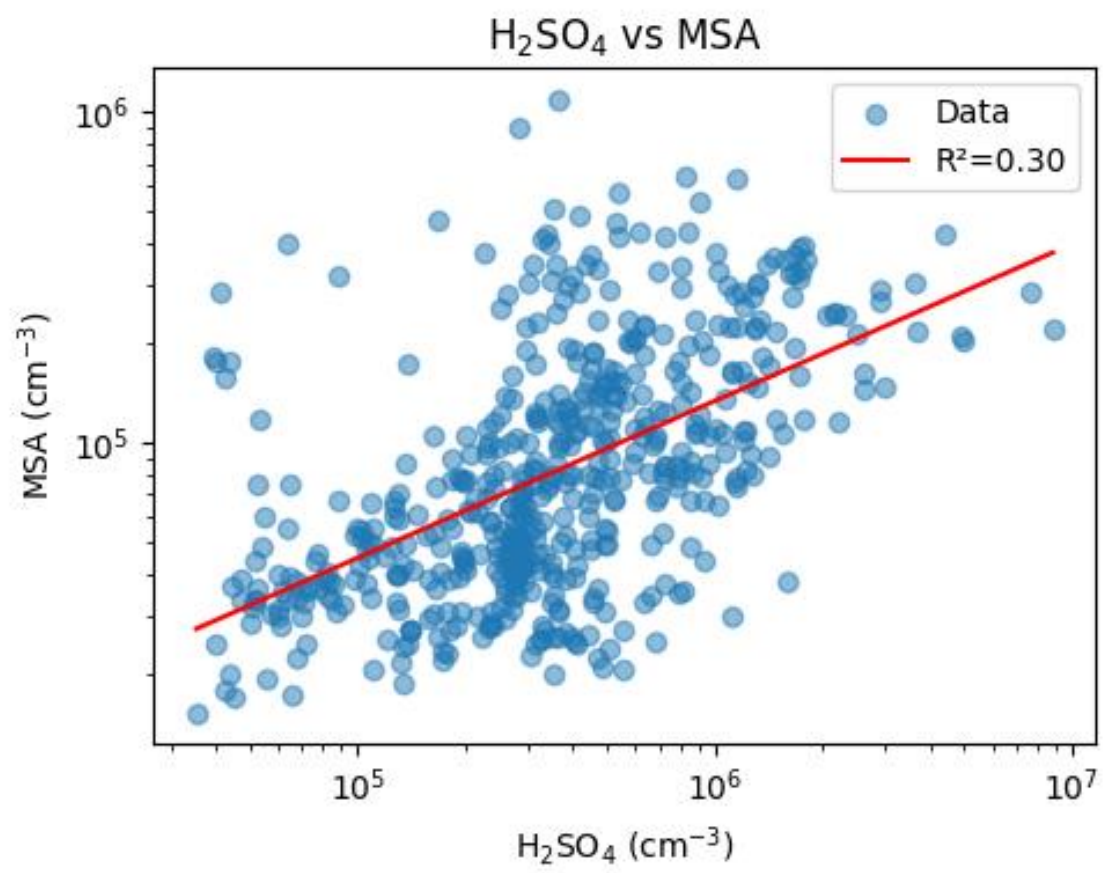

1069 **Figure S11:** Correlation relationship between MSA and H_2SO_4 during the whole campaign.

1070

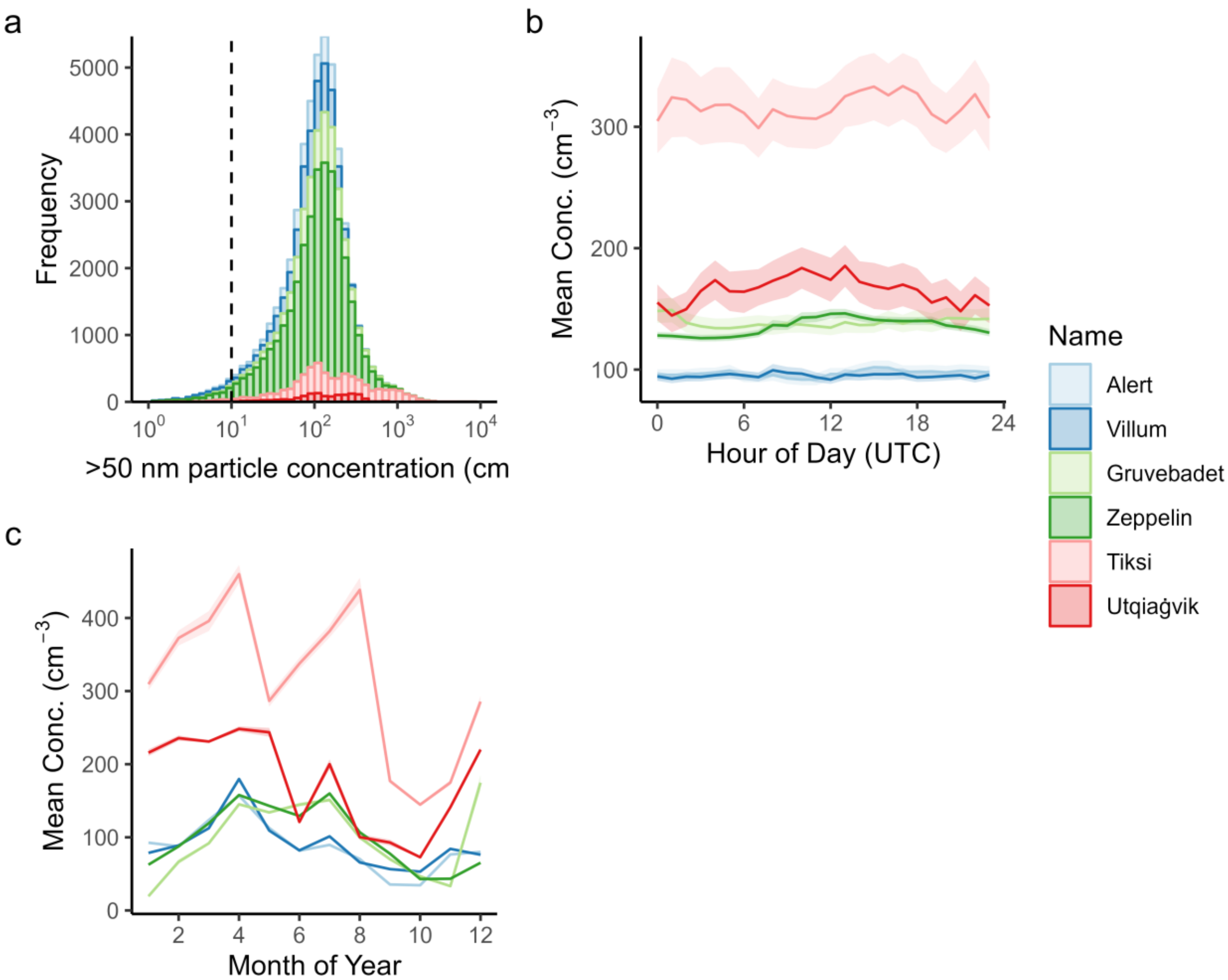

Figure S12: (a) The frequency of different >50 nm particle concentrations in the Arctic for the months of May and June.. Dashed line represents 10 cm^{-3}. The frequency with which particle concentrations are below this threshold are: Alert, 4.10%; Villum Research Station, 6.51%; Gruvebadet, 1.47%; Mt. Zeppelin, 2.79%; Tiksi 2.42%; Utqiaġvik 1.01%. (b) the diurnal cycle of >50 nm particles. (c) the seasonal cycle of >50 nm particles. Dataset is as in Brean et al., (2023) [97]. Shaded region in (b) and (c) represents one standard error on the mean.

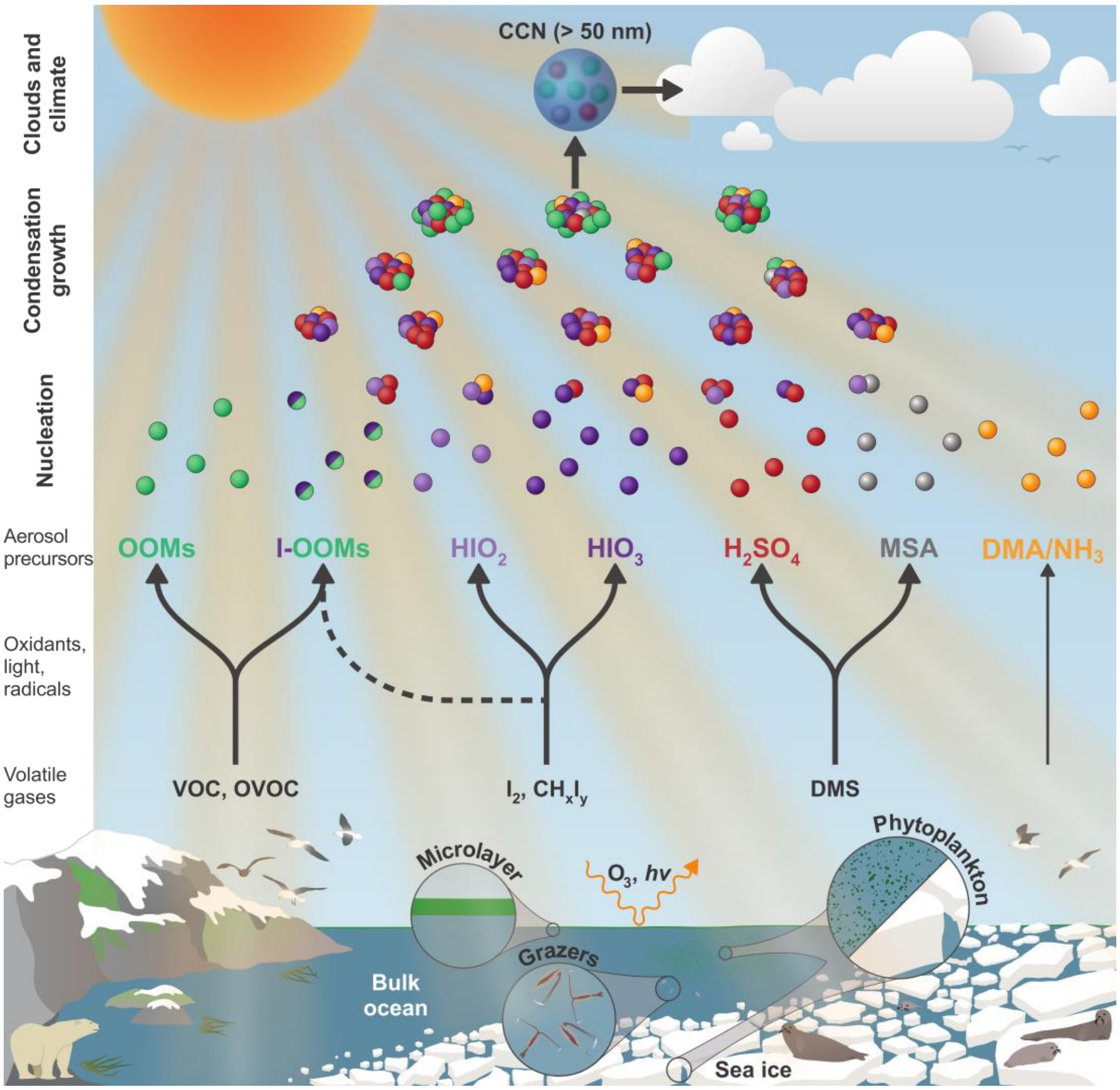

Figure S13: As Figure 5, but generalized for the whole Arctic. Emissions of VOCs, DMS, I_2, and CH_xI_y undergo oxidation to produce OOMs, I-OOMs, H_2SO_4, MSA, and HIO_x. These combine with emissions of alkaline gases (NH_3/DMA) to produce and grow new particles.

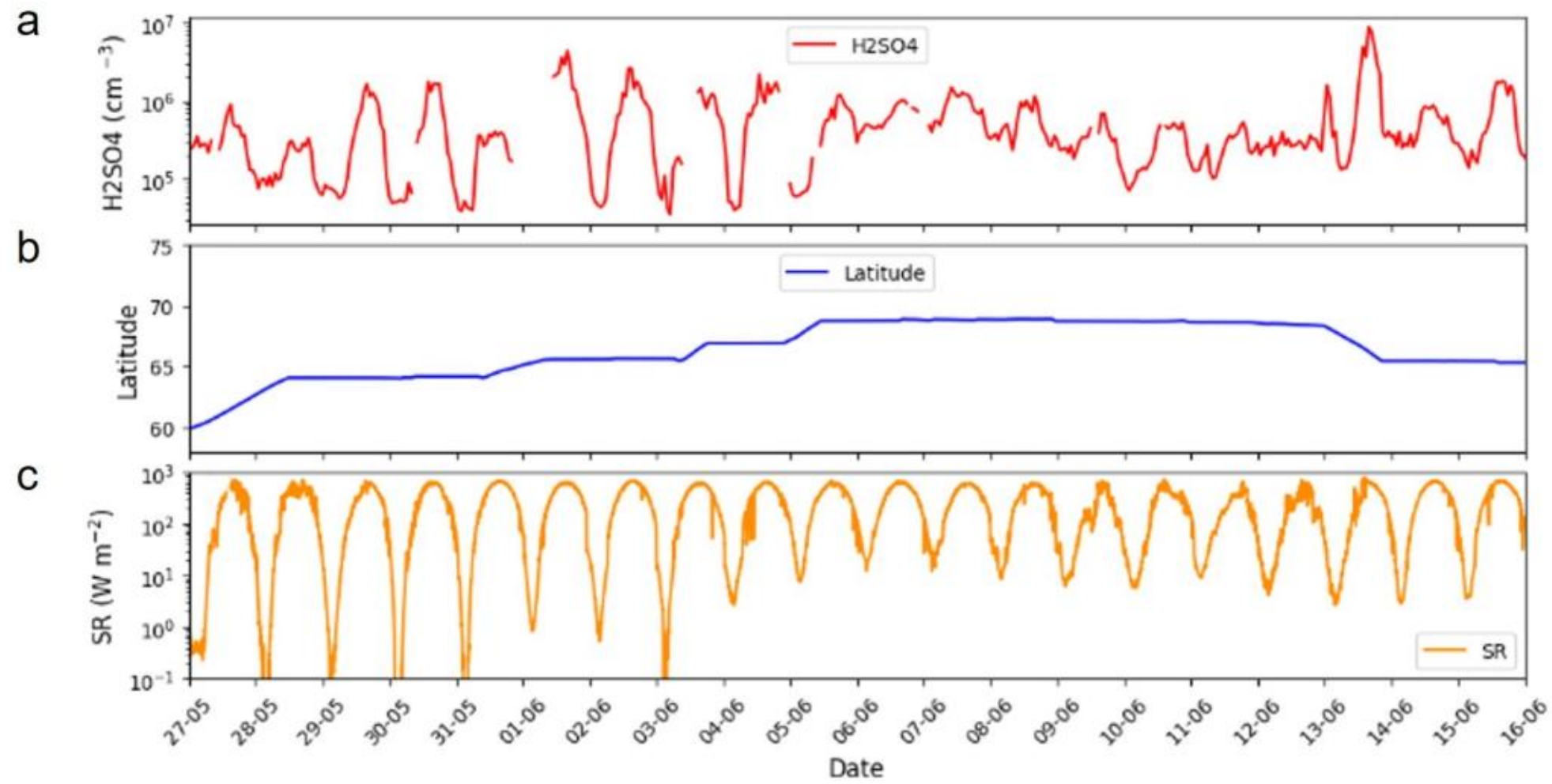

Fig S14: Time-series of (a) H_2SO_4, (b) latitude and (c) solar radiation.

Tables

Table S1. Statistical details of the analyzed data for each particle nucleation event. Date, averaged formation rates ($J_{1.7}$ and J_4) during nucleation periods, growth rate ($GR_{3\text{-}15nm}$), condensation sink (CS), wind speed (WS), temperature, relative humidity (RH), H_2SO_4 concentration, MSA concentration and HIO_3 concentration.

Event Type	Date	$J_{1.7}$ (cm^{-3} s^{-1})	J_4 (cm^{-3} s^{-1})	$GR_{(3\text{-}15nm)}$ (nm h^{-1})	CS (s^{-1})	T (°C)	Wind Speed (m/s)	RH (%)	H_2SO_4 (cm^{-3})	MSA (cm^{-3})	HIO_3 (cm^{-3})
NPF event	23/05/2022	0.23	0.195	2.58	0.0003	2.0	5.6	75.5	4.58E+05	3.26E+05	8.24E+05
	24/05/2022	0.31	0.14	2.38	0.0010	0.9	2.4	87.0	5.55E+05	2.20E+05	6.92E+05
	30/05/2022	0.07	0.04	1.9	0.0011	1.4	5.8	93.4	1.27E+06	3.02E+05	3.94E+05
	01/06/2022	0.79	0.39	N.A	0.0013	3.7	1.7	70.0	3.26E+06	3.11E+05	1.29E+06
	02/06/2022	0.27	0.16	N.A	0.0011	6.4	1.2	55.2	1.77E+06	1.13E+05	2.02E+06
	07/06/2022	0.25	0.19	2.78	0.0004	-0.9	6.3	79.1	1.26E+06	1.16E+05	4.72E+05
	14/06/2022	0.26	0.19	1.46	0.0003	0.5	13.7	79.2	5.83E+05	6.91E+04	1.36E+05
	15/06/2022	0.06	0.04	3.62	0.0004	0.4	10.4	84.4	8.29E+05	1.35E+05	1.42E+05
Burst event	25/05/2022	0.12	0.0	N.A	0.0010	2.8	5.0	63.2	6.15E+05	2.19E+05	4.93E+05
	27/05/2022	0.03	0.02	N.A	0.0003	-0.8	3.1	68.8	7.10E+05	9.46E+04	1.75E+05
	05/06/2022	0.05	0.016	N.A	0.0021	1.3	3.1	90.6	6.50E+05	2.45E+05	8.08E+05
	06/06/2022	0.19	0.09	N.A	0.0009	-0.8	12.4	82.0	9.50E+05	1.01E+05	2.97E+05

	08/06/2022	0.11	0.04	N.A	0.0018	-0.21	2.4	86.0	8.17E+05	1.42E+05	1.30E+06

Note: N.A. means Not available (i.e., cannot be calculated)

Table S2. Summary of identified neutral acids and clusters from the NO_3^- CIMS.

Ion	m/z (Th)
HSO_4-	96.9601
$H_2SO_4NO_3$-	159.9563
$H_2SO_4HNO_3NO_3$-	222.9514
$H_2SO_4HSO_4$-	194.928
CH_3SO_3-	94.9808
$CH_3SO_3HNO_3$-	157.977
$CH_3SO_3HHNO_3NO_3$-	220.9727
IO_3^-	174.8892
HIO_3NO_3-	237.886
$HIO_2HNO_3NO_3$-	284.8856
$H_2SO_4HSO_4^-$	194.9269
$HIO_3HNO_3NO_3^-$	300.8816
$HIO_3HSO_4^-$	272.8565
$HIO_2H_2SO_4HSO_4^-$	354.8291
$C_2H_7NHIO_2IO_3^-$	379.8492
$HIO_3HIO_2NO_3^-$	397.7875

Table S3. Top iodine-containing signals in the I-OOM factor, ordered by decreasing intensity.

Factor	Ion	Volatility class	m/z (Th)
**I-OOM**	$C_8H_{11}IO_5\ NO_3^-$	SVOC	375.953
**I-OOM**	$C_{12}H_{19}IO_5\ NO_3^-$	LVOC	432.016
**I-OOM**	$C_{10}H_{15}IO_9\ NO_3^-$	ELVOC	467.964

**I-OOM**	$C_8H_{12}INO_8\ NO_3^-$	LVOC	438.949
**I-OOM**	$C_{10}H_{17}IO_9\ NO_3^-$	ELVOC	469.980
**I-OOM**	$C_8H_{11}IO_8\ NO_3^-$	LVOC	423.938
**I-OOM**	$C_8H_{13}IO_5\ NO_3^-$	SVOC	377.969
**I-OOM**	$C_{10}H_{13}IO_9\ NO_3^-$	ELVOC	465.948
**I-OOM**	$C_7H_{10}INO_{11}\ NO_3^-$	LVOC	472.918
**I-OOM**	$C_{12}H_{17}IO_5\ NO_3^-$	LVOC	430.000

Table S4. Top signals in each OOM PMF factor, ordered by decreasing intensity.

Factor	Ion	Volatility class	m/z (Th)
Anthro	$C_8H_{12}O_5\ NO_3^-$	SVOC	250.056
	$C_9H_{17}NO_8\ NO_3^-$	LVOC	329.083
	$C_9H_{16}O_6\ NO_3^-$	SVOC	282.083
	$C_5H_8N_2O_5\ NO_3^-$	VOC	238.031
	$C_8H_{13}NO_7\ NO_3^-$	SVOC	297.057
	$C_7H_{10}O_4\ NO_3^-$	IVOC	220.046
	$C_8H_{10}O_6\ NO_3^-$	SVOC	264.036
	$C_9H_{17}NO_7\ NO_3^-$	LVOC	313.088
	$C_{10}H_{16}O_6\ NO_3^-$	LVOC	294.083
	$C_{12}H_{11}NO_6\ NO_3^-$	LVOC	327.046
I-OOM	$C_7H_6O_7\ NO_3^-$	SVOC	263.999
	$C_8H_{13}NO_7\ NO_3^-$	SVOC	297.057
	$C_9H_{15}NO_6\ NO_3^-$	SVOC	295.078
	$C_6H_{11}NO_6\ NO_3^-$	SVOC	255.046
	$C_9H_{17}NO_6\ NO_3^-$	SVOC	297.093
	$C_9H_{16}NO_8\ NO_3^-$	LVOC	328.075
	$C_8H_{11}IO_5\ NO_3^-$	SVOC	375.953
	$C_5H_6O_{10}\ NO_3^-$	SVOC	287.984
	$C_{12}H_{19}IO_5\ NO_3^-$	LVOC	432.016
	$C_8H_{10}O_6\ NO_3^-$	SVOC	264.036
Monoterpene-rich	$C_{10}H_{16}O_{10}\ NO_3^-$	LVOC	358.062
	$C_9H_{15}NO_8\ NO_3^-$	LVOC	327.067
	$C_{10}H_{17}O_7\ NO_3^-$	LVOC	312.093

	$C_9H_{17}NO_6\ NO_3^-$	SVOC	297.093
	$C_{10}H_{17}NO_{10}\ NO_3^-$	LVOC	373.073
	$C_6H_{11}NO_6\ NO_3^-$	SVOC	255.046
	$C_{11}H_{11}NO_4\ NO_3^-$	SVOC	283.057
	$C_7H_{13}NO_6\ NO_3^-$	SVOC	269.062
	$C_{13}H_{24}O_9\ NO_3^-$	ELVOC	386.130
	$C_6H_9NO_7\ NO_3^-$	SVOC	269.026
Aldehyde-rich	$C_9H_{15}NO_9\ NO_3^-$	LVOC	343.063
	$C_{11}H_{21}NO_8\ NO_3^-$	LVOC	357.115
	$C_{10}H_{17}NO_{10}\ NO_3^-$	LVOC	373.073
	$C_9H_{17}NO_8\ NO_3^-$	LVOC	329.083
	$C_{11}H_{19}NO_9\ NO_3^-$	LVOC	371.094
	$C_9H_{14}O_8\ NO_3^-$	LVOC	312.057
	$C_{10}H_{16}O_8\ NO_3^-$	LVOC	326.072
	$C_{13}H_{13}NO_7\ NO_3^-$	LVOC	357.057
	$C_{13}H_{24}O_9\ NO_3^-$	ELVOC	386.130
	$C_9H_{14}O_7\ NO_3^-$	LVOC	296.062

Table S5. Major ion peaks from PTR-MS.

m/z	ions
69.06544	$C_5H_8H^+$
67.0532	$C_5H_6H^+$
71.07669	$C_5H_{10}H^+$
83.08315	$C_6H_{10}H^+$
81.06957	$C_6H_8H^+$
85.09795	$C_6H_{12}H^+$
97.09551	$C_7H_{12}H^+$
95.09279	$C_7H_{10}H^+$
111.11234	$C_8H_{14}H^+$
125.12701	$C_9H_{16}H^+$
127.08999	$C_9H_{18}H^+$
137.12681	$C_{10}H_{16}H^+$
171.16283	$C_{11}H_{22}OH^+$
143.13609	$C_9H_{18}OH^+$

References:

1 Mirme, S. & Mirme, A. The mathematical principles and design of the NAIS – a spectrometer for the measurement of cluster ion and nanometer aerosol size distributions. Atmospheric Measurement Techniques 6, 1061-1071, doi:10.5194/amt-6-1061-2013 (2013).

2 Manninen, H. E., Mirme, S., Mirme, A., Petaja, T. & Kulmala, M. How to reliably detect molecular clusters and nucleation mode particles with Neutral cluster and Air Ion Spectrometer (NAIS). Atmospheric Measurement Techniques 9, 3577-3605, doi:10.5194/amt-9-3577-2016 (2016).

3 Manninen, H. E. et al. Characterisation of corona-generated ions used in a Neutral cluster and Air Ion Spectrometer (NAIS). Atmospheric Measurement Techniques 4, 2767-2776, doi:10.5194/amt-4-2767-2011 (2011).

4 Junninen, H. et al. A high-resolution mass spectrometer to measure atmospheric ion composition. Atmospheric Measurement Techniques 3, 1039-1053, doi:10.5194/amt-3-1039-2010 (2010).

5 Kurten, A., Rondo, L., Ehrhart, S. & Curtius, J. Calibration of a chemical ionization mass spectrometer for the measurement of gaseous sulfuric acid. J Phys Chem A 116, 6375-6386, doi:10.1021/jp212123n (2012).

6 Jokinen, T. et al. Atmospheric sulphuric acid and neutral cluster measurements using CI-APi-TOF. Atmospheric Chemistry and Physics 12, 4117-4125, doi:10.5194/acp-12-4117-2012 (2012).

7 Stark, H. et al. Methods to extract molecular and bulk chemical information from series of complex mass spectra with limited mass resolution. International Journal of Mass Spectrometry 389, 26-38, doi:10.1016/j.ijms.2015.08.011 (2015).

8 Cubison, M. J. & Jimenez, J. L. Statistical precision of the intensities retrieved from constrained fitting of overlapping peaks in high-resolution mass spectra. Atmospheric Measurement Techniques 8, 2333-2345, doi:10.5194/amt-8-2333-2015 (2015).

9 Zhang, Y. et al. A novel approach for simple statistical analysis of high-resolution mass spectra. Atmospheric Measurement Techniques 12, 3761-3776, doi:10.5194/amt-12-3761-2019 (2019).

10 He, X. C. et al. Iodine oxoacids enhance nucleation of sulfuric acid particles in the atmosphere. Science 382, 1308-1314, doi:10.1126/science.adh2526 (2023).

11 Almeida, J. et al. Molecular understanding of sulphuric acid-amine particle nucleation in the atmosphere. Nature 502, 359-363, doi:10.1038/nature12663 (2013).

12 Brean, J. et al. Open ocean and coastal new particle formation from sulfuric acid and amines around the Antarctic Peninsula. Nature Geoscience 14, 383-388, doi:10.1038/s41561-021-00751-y (2021).

13 Baccarini, A. et al. Frequent new particle formation over the high Arctic pack ice by enhanced iodine emissions. Nat Commun 11, 4924, doi:10.1038/s41467-020-18551-0 (2020).

14 Beck, L. J. et al. Differing Mechanisms of New Particle Formation at Two Arctic Sites. Geophysical Research Letters 48, doi:10.1029/2020gl091334 (2021).

15 Yao, L. et al. Atmospheric new particle formation from sulfuric acid and amines in a Chinese megacity. Science 361, 278-281, doi:10.1126/science.aao4839 (2018).

16 Trostl, J. et al. The role of low-volatility organic compounds in initial particle growth in the atmosphere. Nature 533, 527-531, doi:10.1038/nature18271 (2016).

17 Kulkarni, P., Baron, P. A. & Willeke, K. Aerosol Measurement: Principles, Techniques, and Applications. (Wiley, 2011).

18 Hyttinen, N. et al. Modeling the Charging of Highly Oxidized Cyclohexene Ozonolysis Products Using Nitrate-Based Chemical Ionization. J Phys Chem A 119, 6339-6345, doi:10.1021/acs.jpca.5b01818 (2015).

19 Mohr, C. et al. Molecular identification of organic vapors driving atmospheric nanoparticle growth. Nat Commun 10, 4442, doi:10.1038/s41467-019-12473-2 (2019).

20 Heinritzi, M. et al. Characterization of the mass-dependent transmission efficiency of a CIMS. Atmospheric Measurement Techniques 9, 1449-1460, doi:10.5194/amt-9-1449-2016 (2016).

21 Ehn, M. et al. A large source of low-volatility secondary organic aerosol. Nature 506, 476-479, doi:10.1038/nature13032 (2014).

22 Bianchi, F. et al. Highly Oxygenated Organic Molecules (HOM) from Gas-Phase Autoxidation Involving Peroxy Radicals: A Key Contributor to Atmospheric Aerosol. Chem Rev 119, 3472-3509, doi:10.1021/acs.chemrev.8b00395 (2019).

23 Epstein, S. A., Riipinen, I. & Donahue, N. M. A semiempirical correlation between enthalpy of vaporization and saturation concentration for organic aerosol. Environ Sci Technol 44, 743-748, doi:10.1021/es902497z (2010).

24 Donahue, N. M., Epstein, S. A., Pandis, S. N. & Robinson, A. L. A two-dimensional volatility basis set: 1. organic-aerosol mixing thermodynamics. Atmospheric Chemistry and Physics 11, 3303-3318, doi:10.5194/acp-11-3303-2011 (2011).

25 Sulzer, P. et al. A Proton Transfer Reaction-Quadrupole interface Time-Of-Flight Mass Spectrometer (PTR-QiTOF): High speed due to extreme sensitivity. International Journal of Mass Spectrometry 368, 1-5, doi:10.1016/j.ijms.2014.05.004 (2014).

26 Alam, M. S. et al. Mapping and quantifying isomer sets of hydrocarbons (≥ C12) in diesel exhaust, lubricating oil and diesel fuel samples using GC × GC-ToF-MS. Atmospheric Measurement Techniques 11, 3047-3058, doi:10.5194/amt-11-3047-2018 (2018).

27 Rollins, A. W. et al. A laser-induced fluorescence instrument for aircraft measurements of sulfur dioxide in the upper troposphere and lower stratosphere. Atmospheric Measurement Techniques 9, 4601-4613, doi:10.5194/amt-9-4601-2016 (2016).

28 Liu, X. et al. Variability of ambient air ammonia in urban Europe (Finland, France, Italy, Spain, and the UK). Environment International 185, 108519, doi:10.1016/j.envint.2024.108519 (2024).

29 Canagaratna, M. R. et al. Chemical and microphysical characterization of ambient aerosols with the aerodyne aerosol mass spectrometer. Mass Spectrom Rev 26, 185-222, doi:10.1002/mas.20115 (2007).

30 Zhang, Y. et al. Seasonal characterization of components and size distributions for submicron aerosols in Beijing. Science China Earth Sciences 56, 890-900, doi:10.1007/s11430-012-4515-z (2012).

31 Zhang, Y. et al. Measurements of particulate methanesulfonic acid above the remote Arctic Ocean using a High Resolution Aerosol Mass Spectrometer. Atmospheric Environment, doi:10.1016/j.atmosenv.2024.120538 (2024).

32 Roberts, G. C. & Nenes, A. A Continuous-Flow Streamwise Thermal-Gradient CCN Chamber for Atmospheric Measurements. Aerosol Science and Technology 39, 206-221, doi:10.1080/027868290913988 (2005).

33 Dall'Olmo, G., Westberry, T. K., Behrenfeld, M. J., Boss, E. & Slade, W. H. Significant contribution of large particles to optical backscattering in the open ocean. Biogeosciences 6, 947-967, doi:10.5194/bg-6-947-2009 (2009).

34 Dall'Olmo, G. & Mork, K. A. Carbon export by small particles in the Norwegian Sea. Geophysical Research Letters 41, 2921-2927, doi:10.1002/2014gl059244 (2014).

35 Boss, E. S., Collier, R., Larson, G., Fennel, K. & Pegau, W. S. Measurements of spectral optical properties and their relation to biogeochemical variables and processes in Crater Lake, Crater Lake National Park, OR. Hydrobiologia 574, 149-159, doi:10.1007/s10750-006-2609-3 (2007).

36 Kulmala, M. et al. Measurement of the nucleation of atmospheric aerosol particles. Nat Protoc 7, 1651-1667, doi:10.1038/nprot.2012.091 (2012).

37 Dal Maso, M. et al. Formation and growth of fresh atmospheric aerosols: eight years of aerosol size distribution data from SMEAR II, Hyytiala, Finland. Boreal Environment Research 10, 323-336 (2005).

38 Kerminen, V.-M. et al. Atmospheric new particle formation and growth: review of field observations. Environmental Research Letters 13, 103003, doi:10.1088/1748-9326/aadf3c (2018).

39 Cai, R. et al. Survival probability of new atmospheric particles: closure between theory and measurements from 1.4 to 100 nm. Atmospheric Chemistry and Physics 22, 14571-14587, doi:10.5194/acp-22-14571-2022 (2022).

40 Boyer, M. et al. Penguin guano is an important source of climate-relevant aerosol particles in Antarctica. Communications Earth & Environment 6, 368, doi:10.1038/s43247-025-02312-2 (2025).

41 Yli-Juuti, T. et al. Growth rates of nucleation mode particles in Hyytiälä during 2003−2009: variation with particle size, season, data analysis method and ambient conditions. Atmos. Chem. Phys. 11, 12865-12886, doi:10.5194/acp-11-12865-2011 (2011).

42 Stolzenburg, D. et al. Enhanced growth rate of atmospheric particles from sulfuric acid. Atmospheric Chemistry and Physics 20, 7359-7372, doi:10.5194/acp-20-7359-2020 (2020).

43 Nieminen, T., Lehtinen, K. E. J. & Kulmala, M. Sub-10 nm particle growth by vapor condensation – effects of vapor molecule size and particle thermal speed. Atmospheric Chemistry and Physics 10, 9773-9779, doi:10.5194/acp-10-9773-2010 (2010).

44 Chan, T. & Mozurkewich, M. Measurement of the coagulation rate constant for sulfuric acid particles as a function of particle size using tandem differential mobility analysis. Journal of Aerosol Science 32, 321-339, doi:10.1016/s0021-8502(00)00081-1 (2001).

45 Paatero, P. & Tapper, U. Positive matrix factorization: A non-negative factor model with optimal utilization of error estimates of data values. Environmetrics 5, 111-126, doi:10.1002/env.3170050203 (1994).

46 Yan, C. et al. Source characterization of highly oxidized multifunctional compounds in a boreal forest environment using positive matrix factorization. Atmospheric Chemistry and Physics 16, 12715-12731, doi:10.5194/acp-16-12715-2016 (2016).

47 Ulbrich, I. M., Canagaratna, M. R., Zhang, Q., Worsnop, D. R. & Jimenez, J. L. Interpretation of organic components from Positive Matrix Factorization of aerosol mass spectrometric data. Atmospheric Chemistry and Physics 9, 2891-2918, doi:10.5194/acp-9-2891-2009 (2009).

48 Canonaco, F., Crippa, M., Slowik, J. G., Baltensperger, U. & Prévôt, A. S. H. SoFi, an IGOR-based interface for the efficient use of the generalized multilinear engine (ME-2) for the

source apportionment: ME-2 application to aerosol mass spectrometer data. Atmospheric Measurement Techniques 6, 3649-3661, doi:10.5194/amt-6-3649-2013 (2013).

49 Stein, A. F. et al. NOAA's HYSPLIT Atmospheric Transport and Dispersion Modeling System. Bulletin of the American Meteorological Society 96, 2059-2077, doi:10.1175/BAMS-D-14-00110.1 (2015).

50 National Centers for Environmental Prediction (NCEP). NCEP Global Data Assimilation System (GDAS) Atmospheric Analyses. NOAA National Centers for Environmental Information, Date Accessed 06-30-2026 (2026).

51 DiGirolamo, N., Parkinson, C. L., Cavalieri, D. J., Gloersen, P. & Zwally, H. J. Sea Ice Concentrations from Nimbus-7 SMMR and DMSP SSM/I-SSMIS Passive Microwave Data (NSIDC-0051, Version 2). NASA National Snow and Ice Data Center Distributed Active Archive Center, doi:10.5067/MPYG15WAA4WX, Date Accessed 06-30-2026 (2022).

52 Schmale, J. & Baccarini, A. Progress in Unraveling Atmospheric New Particle Formation and Growth Across the Arctic. Geophysical Research Letters 48, doi:10.1029/2021gl094198 (2021).

53 Quéléver, L. L. J. et al. Investigation of new particle formation mechanisms and aerosol processes at Marambio Station, Antarctic Peninsula. Atmospheric Chemistry and Physics 22, 8417-8437, doi:10.5194/acp-22-8417-2022 (2022).

54 Jokinen, T. et al. Ion-induced sulfuric acid–ammonia nucleation drives particle formation in coastal Antarctica. Science Advances 4, eaat9744, doi:10.1126/sciadv.aat9744 (2018).

55 Kirkby, J. et al. Atmospheric new particle formation from the CERN CLOUD experiment. Nature Geoscience 16, 948-957, doi:10.1038/s41561-023-01305-0 (2023).

56 Xiao, M. et al. The driving factors of new particle formation and growth in the polluted boundary layer. Atmospheric Chemistry and Physics 21, 14275-14291, doi:10.5194/acp-21-14275-2021 (2021).

57 Willis, M. D. et al. Growth of nucleation mode particles in the summertime Arctic: a case study. Atmospheric Chemistry and Physics 16, 7663-7679, doi:10.5194/acp-16-7663-2016 (2016).

58 Willis, M. D. et al. Evidence for marine biogenic influence on summertime Arctic aerosol. Geophysical Research Letters 44, 6460-6470, doi:10.1002/2017GL073359 (2017).

59 Burkart, J. et al. Organic Condensation and Particle Growth to CCN Sizes in the Summertime Marine Arctic Is Driven by Materials More Semivolatile Than at Continental Sites. Geophysical Research Letters 44, 10,725-710,734, doi:10.1002/2017GL075671 (2017).

60 Croft, B. et al. Arctic marine secondary organic aerosol contributes significantly to summertime particle size distributions in the Canadian Arctic Archipelago. Atmos. Chem. Phys. 19, 2787-2812, doi:10.5194/acp-19-2787-2019 (2019).

61 Mungall, E. L. et al. Microlayer source of oxygenated volatile organic compounds in the summertime marine Arctic boundary layer. Proceedings of the National Academy of Sciences 114, 6203-6208, doi:10.1073/pnas.1620571114 (2017).

62 Schneider, S. R. et al. Abiotic Emission of Volatile Organic Compounds from the Ocean Surface: Relationship to Seawater Composition. ACS Earth and Space Chemistry, doi:10.1021/acsearthspacechem.4c00163 (2024).

63 Leaitch, W. R. et al. Effects of 20–100 nm particles on liquid clouds in the clean summertime Arctic. Atmospheric Chemistry and Physics 16, 11107-11124, doi:10.5194/acp-16-11107-2016 (2016).

64 Song, C. et al. Understanding Sources and Drivers of Size-Resolved Aerosol in the High Arctic Islands of Svalbard Using a Receptor Model Coupled with Machine Learning. Environmental Science & Technology 56, 11189-11198, doi:10.1021/acs.est.1c07796 (2022).

65 Kirkby, J. et al. Role of sulphuric acid, ammonia and galactic cosmic rays in atmospheric aerosol nucleation. Nature 476, 429-433, doi:10.1038/nature10343 (2011).

66 Zu, H., Chu, B., Lu, Y., Liu, L. & Zhang, X. Rapid iodine oxoacid nucleation enhanced by dimethylamine in broad marine regions. Atmos. Chem. Phys. 24, 5823-5835, doi:10.5194/acp-24-5823-2024 (2024).

67 Croft, B. et al. Contribution of Arctic seabird-colony ammonia to atmospheric particles and cloud-albedo radiative effect. Nat Commun 7, 13444, doi:10.1038/ncomms13444 (2016).

68 Wentworth, G. R. et al. Ammonia in the summertime Arctic marine boundary layer: sources, sinks, and implications. Atmospheric Chemistry and Physics 16, 1937-1953, doi:10.5194/acp-16-1937-2016 (2016).

69 Wang, M. et al. Rapid growth of new atmospheric particles by nitric acid and ammonia condensation. Nature 581, 184-189, doi:10.1038/s41586-020-2270-4 (2020).

70 Wang, M. et al. Synergistic HNO3-H2SO4-NH3 upper tropospheric particle formation. Nature 605, 483-489, doi:10.1038/s41586-022-04605-4 (2022).

71 Chamba, G. et al. Evidence of nitrate-based nighttime atmospheric nucleation driven by marine microorganisms in the South Pacific. Proc Natl Acad Sci U S A 120, e2308696120, doi:10.1073/pnas.2308696120 (2023).

72 Wang, S., Wu, R., Berndt, T., Ehn, M. & Wang, L. Formation of Highly Oxidized Radicals and Multifunctional Products from the Atmospheric Oxidation of Alkylbenzenes. Environ Sci Technol 51, 8442-8449, doi:10.1021/acs.est.7b02374 (2017).

73 Saiz-Lopez, A. et al. Atmospheric chemistry of iodine. Chem Rev 112, 1773-1804, doi:10.1021/cr200029u (2012).

74 Tham, Y. J. et al. Widespread detection of chlorine oxyacids in the Arctic atmosphere. Nature Communications 14, 1769, doi:10.1038/s41467-023-37387-y (2023).

75 Boyer, M. et al. The annual cycle and sources of relevant aerosol precursor vapors in the central Arctic. Atmospheric Chemistry and Physics 24, 12595-12621, doi:10.5194/acp-24-12595-2024 (2024).

76 Brown, L. V. et al. Iodine speciation in snow during the MOSAiC expedition and its implications for Arctic iodine emissions. Faraday Discussions 258, 441-472, doi:10.1039/D4FD00178H (2025).

77 Mao, J. et al. Chemistry of hydrogen oxide radicals (HOx) in the Arctic troposphere in spring. Atmos. Chem. Phys. 10, 5823-5838, doi:10.5194/acp-10-5823-2010 (2010).

78 Atkinson, R. & Arey, J. Atmospheric Degradation of Volatile Organic Compounds. Chemical Reviews 103, 4605-4638, doi:10.1021/cr0206420 (2003).

79 Bowman, J. H., Barket, D. J. & Shepson, P. B. Atmospheric Chemistry of Nonanal. Environmental Science & Technology 37, 2218-2225, doi:10.1021/es026220p (2003).

80 Wang, Z. et al. Efficient alkane oxidation under combustion engine and atmospheric conditions. Commun Chem 4, 18, doi:10.1038/s42004-020-00445-3 (2021).

81 Riipinen, I. et al. The contribution of organics to atmospheric nanoparticle growth. Nature Geoscience 5, 453-458, doi:10.1038/ngeo1499 (2012).

82 Huang, R.-J. et al. Heterogeneous iodine-organic chemistry fast-tracks marine new particle formation. Proceedings of the National Academy of Sciences 119, e2201729119, doi:10.1073/pnas.2201729119 (2022).

83 Pospisilova, V. et al. On the fate of oxygenated organic molecules in atmospheric aerosol particles. Sci Adv 6, eaax8922, doi:10.1126/sciadv.aax8922 (2020).

84 Yli-Juuti, T., Mohr, C. & Riipinen, I. Open questions on atmospheric nanoparticle growth. Communications Chemistry 3, doi:10.1038/s42004-020-00339-4 (2020).

85 Hyttinen, N. et al. Comparison of saturation vapor pressures of α-pinene + O3 oxidation products derived from COSMO-RS computations and thermal desorption experiments. Atmospheric Chemistry and Physics 22, 1195-1208, doi:10.5194/acp-22-1195-2022 (2022).

86 Kilgour, D. B., Novak, G. A., Claflin, M. S., Lerner, B. M. & Bertram, T. H. Production of oxygenated volatile organic compounds from the ozonolysis of coastal seawater. Atmos. Chem. Phys. 24, 3729-3742, doi:10.5194/acp-24-3729-2024 (2024).

87 Vermeuel, M. P. et al. Observations of biogenic volatile organic compounds over a mixed temperate forest during the summer to autumn transition. Atmos. Chem. Phys. 23, 4123-4148, doi:10.5194/acp-23-4123-2023 (2023).

88 Coggon, M. M. et al. Identifying and correcting interferences to PTR-ToF-MS measurements of isoprene and other urban volatile organic compounds. Atmos. Meas. Tech. 17, 801-825, doi:10.5194/amt-17-801-2024 (2024).

89 Rontani, J.-F. et al. Use of palmitoleic acid and its oxidation products for monitoring the degradation of ice algae in Arctic waters and bottom sediments. Organic Geochemistry 124, 88-102, doi:10.1016/j.orggeochem.2018.06.002 (2018).

90 Thornberry, T. & Abbatt, J. P. D. Heterogeneous reaction of ozone with liquid unsaturated fatty acids: detailed kinetics and gas-phase product studies. Physical Chemistry Chemical Physics 6, doi:10.1039/b310149e (2004).

91 Zhou, S. et al. Formation of gas-phase carbonyls from heterogeneous oxidation of polyunsaturated fatty acids at the air–water interface and of the sea surface microlayer. Atmospheric Chemistry and Physics 14, 1371-1384, doi:10.5194/acp-14-1371-2014 (2014).

92 Chang, R. Y. W. et al. Relating atmospheric and oceanic DMS levels to particle nucleation events in the Canadian Arctic. Journal of Geophysical Research Atmospheres 116, doi:10.1029/2011JD015926 (2011).

93 Tian, S. L., Pan, Y. P. & Wang, Y. S. Size-resolved source apportionment of particulate matter in urban Beijing during haze and non-haze episodes. Atmospheric Chemistry and Physics 16, 1-19, doi:10.5194/acp-16-1-2016 (2016).

94 Leaitch, W. R. et al. Dimethyl sulfide control of the clean summertime Arctic aerosol and cloud. Elementa: Science of the Anthropocene 1, doi:10.12952/journal.elementa.000017 (2013).

95 Sharma, S. et al. Influence of transport and ocean ice extent on biogenic aerosol sulfur in the Arctic atmosphere. Journal of Geophysical Research: Atmospheres 117, doi:10.1029/2011jd017074 (2012).

96 Tremblay, S. et al. Characterization of aerosol growth events over Ellesmere Island during the summers of 2015 and 2016. Atmospheric Chemistry and Physics 19, 5589-5604, doi:10.5194/acp-19-5589-2019 (2019).

97 Brean, J. et al. Collective geographical ecoregions and precursor sources driving Arctic new particle formation. Atmospheric Chemistry and Physics 23, 2183-2198, doi:10.5194/acp-23-2183-2023 (2023).
