# Supplementary material for: Arctic cloud condensation nuclei enhanced by iodine, sulfur and organic precursors
Source: Nat Geosci. 2026 Aug 5;19(9):1039–46. doi: 10.1038/s41561-026-02062-6 (PMC13541622; doi:10.1038/s41561-026-02062-6)
Supplement: Supplementary file 2 [file 41561_2026_2062_MOESM2_ESM.pdf]

nature portfolio

Peer Review File

Arctic cloud condensation nuclei enhanced by new particle formation from iodine, sulfur, and organics

Corresponding Author: Professor Zongbo Shi

Version 1:

Reviewer comments:

Reviewer #1

(Remarks to the Author)
I have already reviewed the first version of this manuscript, which was originally submitted to Nature. Many thanks to the authors for their careful and comprehensive revisions. The manuscript has improved significantly as a result, and I have no further substantial comments. The manuscript presents data on new particle formation during an Arctic expedition, thus representing an understudied but important region for regional climate change. The interpretation is comprehensive and it is now appropriately cautious. I recommend publication in Nature Geoscience.

Technical comments:
(line numbers refer to file 50911_1_supp_462901_t81ys0_convrt.pdf, manuscript with track changes included).
l. 50, enhancing --> enhance
l. 101: consequent --> subsequent
l. 101: growth to Aitken mode size aerosol --> growth to aerosol sizes that range in the Aitken mode
l. 102: what do you mean when stating “many of which have crossover with the diameter of growing particles”?
l. 132-133: “on all NPF (8 days), burst (5 days), and non-event (8 days) event days” --> “on all NPF event (8 days), burst event (5 days), and non-event (8 days) days”
l. 196: clustered-clustered forms --> clustered forms
l. 210 “solid red line” --> “dashed cyan line” ?
l. 211 and l. 214., not useful to print the same fitting equation twice
l. 224: what is meant by “daily hourly…”
l. 230 Fig S8A says1-2 ppb NH3, here you state 10 ppb NH3. Please correct.
Also, you sometimes use “pptv” and “ppbv”, and sometimes “ppt” and “ppb”. Please use one unit consistently throughout.
l. 342: “MIZ”. Please explain the acronym (or omit the acronym as it is not used in the rest of the paper.
l. 343: Do not start a sentence with “>90 %” --> ”). More than 90%”
l. 363: flew --> flowed
l. 367-371. Long convoluted sentence, please simplify…
l. 377 previous --> previously
l. 379 direct ambient observational evidence --> direct observational evidence
l. 445 flew --> flowed
l. 447 marginal sea ice zone --> the marginal sea ice zone
l. 451-452 repetition of “likely ubiquitous”
l. 457 bird nests --> bird colonies
l. 458 central ocean --> open ocean

Reviewer #6

(Remarks to the Author)
Review comments
Du et al. report comprehensive shipborne measurements of new particle and CCN formation processes in the Arctic, building a critical link between earlier laboratory studies and ambient observations. The primary novelty of this work lies in the full coverage of NPF processes up to CCN sizes, including direct CCN detection, which distinguishes it from many previous studies. This study also validates the iodine-sulfur synergy in aerosol formation proposed by He et al. (2023) within realistic ambient conditions, confirming the relevance of this laboratory-based nucleation mechanism. Furthermore, the authors provide solid results indicating that OOMs, likely derived from aldehydes, which dominate particle growth processes. Additionally, a new category of OOMs, namely I-OOMs, is identified for the first time and proposed to contribute to particle growth, albeit to a lesser extent.

In my view, while each of these advances is valuable, it is not immediately clear that any single contribution alone necessitates publication in Nature Geoscience. The authors appear to recognize this, as the manuscript endeavors to articulate various aspects of novelty relative to existing literature. However, the strategy of aggregating these subtle novelties occasionally detracts from the central narrative. Consequently, the current results do not yet appear sufficient to support several key conclusions - points which seemingly reflect the challenges raised during the initial review process by other reviewers.

Specifically, the manuscript attempts to distinguish this work from previous studies, such as investigations by Beck et al. (2021) and Baccarini et al. (2020), by characterizing them as regional studies to reflect the complexity of specific Arctic environments that cannot be easily extrapolated. However, it effectively remains that the current work is also a regional study, with an observational timeframe that is shorter than many preceding campaigns. This therefore puts a large weight on trying to generalize these results to marginal sea ice zones, but the current justification remains insufficient.

As a reviewer invited for the second round, I initially evaluated the updated manuscript without consulting the previous reviews or response letters. Upon subsequently reviewing the history, I noted that my independent observations largely echoed concerns previously raised by other reviewers. This suggests that the revision has not yet sufficiently addressed the fundamental issues required to clearly improve the manuscript.

Despite the critical nature of the comments above and the detailed discussion below, I consider this study to be an important contribution to our understanding of Arctic aerosol formation processes. Many of the raised items are something that can be addressed by the authors, and I believe the work has the potential to meet the standards of Nature Geoscience provided these issues are thoroughly addressed. I encourage the authors to consider these points, after which I would be willing to reevaluate the manuscript.

Major:

Comment 1:
A significant oversight in the present analysis, which was also not addressed in the previous round of review, is the effect of the condensation sink (CS) - and more specifically the associated coagulation sink - on new particle formation rates. The authors compare their observed nucleation rates with mechanisms derived from the CERN CLOUD experiments, identifying the HIO_x-H_2SO_4 mechanism presented by He et al. (2023) as the best fit. However, the measured values remain roughly an order of magnitude higher than those reported by He et al. (2023). To reconcile this discrepancy, the authors appear to seek explanations involving other mechanisms, such as enhancement by DMA and OOMs. However, as reported in this version of the manuscript, the CS during the cruise expedition was approximately 3-4 times lower than in the CLOUD experiments. Consequently, based on the same HIO_x-H_2SO_4 nucleation mechanism, higher nucleation rates should indeed be expected in the expedition data due to reduced scavenging losses. For example, Zhang et al. (2022; 10.1021/acs.est.2c04328) calculated the impact of CS on the nucleation rate of the iodine oxoacid system. With 1e6 of HIO_3 (and fixed 4.32e5 HIO_2), at typical CLOUD CS conditions (0.002 s^{-1}), the nucleation rate is approximately 0.2 cm^{-3} s^{-1} (Figure 4 of their study). Conversely, if the CS is reduced to 0.0002 s^{-1} under the same vapor conditions (Figure S2 of their study), the rate increases to around 40 cm^{-3} s^{-1}. Although Zhang et al. investigated the pure HIO_x system, it is mechanistically a simplified version of the HIO_x-H_2SO_4 system, suggesting that these quantitative trends are likely applicable here as well. Therefore, it is not surprising that the present study measures rates an order of magnitude higher than the He et al. (2023) fit. On the contrary, when accounting for the differences in sinks, this likely represents a surprisingly good agreement. I believe a major revision is needed in this study to reflect this (e.g., lines 227-234; 235-240 etc); I believe this has the potential to sharpen the message of this study as well.

Comment 2:
Potentially related to my previous major comment, I find it difficult to understand the authors' insistence on positioning the DMA contribution to aerosol nucleation as a key element, particularly given the controversies raised by Reviewers 1, 2, 3, and 4 previously, as well as my own concerns detailed below. Compared to other conclusions in the manuscript, the assertion that DMA is a major player in aerosol nucleation appears fundamentally flawed.
The primary evidence supporting the role of DMA is the detection of C_2H_7N-HIO_2-IO_3^- (Fig. S5). This relies on a single peak which is not unambiguously detected in many events. Unlike the other four peaks discussed - which have periods where they clearly dominate the spectra - this specific DMA cluster is, in most cases, fitted from residual signals, except the short period where the peak is identified (Fig. S9). In my view, it is impossible to use this as concrete evidence for identifying NPF mechanisms, particularly not quantitatively. At maximum, this is a sporadic event that is not regionally important. All previous studies cited in this work (e.g., Almeida et al., 2013; Yao et al., 2018; Xiao et al., 2021) show multiple unambiguous DMA-containing clusters when DMA enhancement is significant. It is also noteworthy that the authors did not measure DMA concentrations but instead measured this cluster, which is uncommon, as cluster concentrations (especially for this trimer) are typically lower than monomer concentrations during NPF events.
Additionally, in the response materials (e.g., Comment 2 of Reviewers 1 & 2), the authors combined AMS mass/volume identification with growth inverse modeling to estimate the gas-phase concentration of DMA, concluding that DMA+TMA might be around 1.8 pptv. I find the identification of C_2H_7N and C_3H_9N in Figure R1 unclear and the quantitative assignment of their concentrations challenging (even worse than the gas phase identification). Furthermore, one cannot apply growth modeling developed for condensable vapors (like organics and H_2SO_4) to amines in this manner. Because amines are not directly condensable vapors (often requiring acid-base stabilization), their accommodation coefficient is highly uncertain; simply relying on volatility ranges for this calculation is inaccurate and methodologically unsound.
Finally, if ~2 pptv of DMA were indeed present in the field observations (levels comparable to urban environments), one

would expect to see significantly more DMA-containing clusters and a much faster nucleation rate than currently reported. The observed rates should be comparable than the Xiao et al. (2021) data (Figure S8) and the Kürten et al. (2018) data - which should be included in Figure S8 for reference. Once again, considering the much lower CS in the Arctic compared with CLOUD, especially during the DMA experiments, if DMA were important, the rates in the field should be significantly higher than Xiao et al. and Kürten et al. The current data contradicts this expectation.
Overall, the slight modification acknowledging that DMA enhancement is merely "potential" (lines 42–43) is insufficient to address the collective concerns of the reviewers, especially since this is still proposed as a major advance of the study (lines 227–234 and Fig. 5). I do not believe the DMA enhancement arguments are solid, and the heavy focus on them seems counterproductive (there are over 20 mentioning of DMA in the manuscript). It makes the manuscript vulnerable by stretching to propose a "new" mechanism not supported by robust evidence. I strongly suggest the authors reevaluate their data and avoid making overly strong claims regarding DMA enhancement, unless they have additional, much more solid evidence.

Comment 3:
Throughout the response documents and the introduction, the authors imply that earlier studies (e.g., Baccarini et al., Beck et al.) are limited by their specific regional and temporal scope, potentially lacking representativeness for the wider Arctic. Conversely, they imply the current study as more representative (lines 101–107) and suitable for predicting the future Arctic. However, this distinction appears overstated given that the present study is similarly constrained to a specific region during a specific season (May–June) for approximately one month. It is not immediately clear that the geographical and temporal coverage here represents a significant advancement over earlier studies in this specific regard.
The authors attempt to associate certain NPF events (e.g., lines 304 onwards) with the marginal sea ice zone and subsequently extrapolate these findings to claim relevance for the entire Arctic (lines 400–408). As discussed in detail by Reviewers 3 and 4, such extrapolation remains tenuous. Furthermore, it is unclear why the limitations cited for Beck et al. (2021) and Baccarini et al. (2020) would not apply here, or conversely, why those studies could not make similar extrapolations using the same logic.
It is likely that the mechanisms proposed in this study and those identified in earlier works coexist, with dominance depending on location and season. However, a genuine distinction and strength of this work lies in the validation of the iodine-sulfur synergy proposed by He et al. (2023). Unlike earlier studies that lacked evidence for this specific interaction, the current measurements provide crucial validation for this mechanism in ambient conditions. Therefore, the discussion should be repositioned to more accurately reflect the study's scope, rather than framing shared methodological limitations as distinct advantages over prior work.

Comment 4:
The section titled "Sources and processes influencing high latitude new particle and CCN formation" reads more like a literature review than a synthesis of results from the present study. It relies heavily on findings from earlier studies; for example, it puts MSA and NH3 as key species in the particle formation events, even though this study provides limited data on these compounds. While it is scientifically plausible - based on the broader literature - that Arctic NPF involves these species, the mechanisms currently depicted in the figure are sufficiently generic that they could describe NPF events in many environments globally, rather than highlighting the unique characteristics of the events observed here. Consequently, the utility of this schematic is limited. I suggest that the authors significantly streamline this section and revise Figure 5 to focus primarily on the direct findings of this study, rather than presenting a generalized overview of potential pathways.

Comment 5:
Another critical limitation of the current study is the absence of natural ion measurements using APi-TOF. While the authors consistently highlighted the comprehensiveness of their dataset compared to earlier studies (Table R1), the APi-TOF remains a critical instrument for atmospheric nucleation studies. This is particularly relevant in pristine environments like the Arctic, where measuring abundant small ionic clusters is often essential for identifying specific nucleation schemes. Without these data, it is difficult to definitively assess the importance of mechanisms such as SA-NH3 and SA-DMA nucleation. Furthermore, neutral measurements obtained using Nitrate-CIMS can be influenced by natural ion detection if an inlet ion filter is not installed. As the manuscript does not mention the use of such an ion filter, I assume one was not present. Without parallel information from an APi-TOF, it becomes difficult to determine if species such as H2SO4-HSO4- are genuine neutral sulfuric acid dimers (which would indicate strong sulfuric acid nucleation) or if they originate from ambient natural ions. Consequently, there is a risk that the clusters presented in Figure S5 - with the exception of HIO3HIO2NO3- - may be sourced from natural ions rather than neutral clusters. In fact, it appears highly probable that the presented trimers are natural ions rather than neutral clusters, given that the reported nucleation rates are primarily below 1 cm-3 s-1. Generally, nucleation events at such low rates do not yield neutral cluster concentrations distinct enough to exceed the typical detection limit of nitrate-CIMS (approximately 1e4 cm-3). I suggest the authors employ a simple kinetic model to simulate the cluster formation process to evaluate this feasibility. When doing so, they should avoid simply assuming kinetic collision rates for HIO3 or H2SO4, given their own argument that the nucleation mechanism involves additional components such as HIO2. This possibility reinforces the concerns raised in Comment 2 regarding the DMA cluster (C2H7NHIO2IO3-); if this signal originates from natural ions, a single peak in the spectra is essentially inconclusive unless a corresponding cluster sequence is observed. Unless the authors observe forms involving the NO3- ion (which would confirm chemical ionization), this uncertainty remains.
I strongly suggest that the authors review their arguments with this limitation in mind and acknowledge this caveat in SI and main text. Additionally, I recommend structuring the narrative to prioritize solid, valid measurements - such as the concentration of neutral acids and observed aerosol formation and growth rates - before expanding into highly uncertain discussions regarding DMA and OOMs, if such discussion is necessary at all.

Comment 6:

Lines 235-240 discuss the potential contribution of OOMs to aerosol nucleation. Based on the review history, this section appears to have been added in response to Reviewers 1 and 2 and was not present in the original manuscript. Issues raised by other reviewers remain regarding whether the VBS parameterization used here can adequately describe OOMs derived from aldehydes. While I commend the authors for choosing a general form over specific targeted forms for monoterpenes, this does not eliminate the substantial uncertainty inherent in the parameterization (imagine that's exactly why targeted forms are needed...). Specifically, the categorization of ELVOCs and ULVOCs - nor arguments based purely on concentration - can be used as direct evidence of whether OOMs are contributing to nucleation.
To address this, I suggest the authors color-code their field observations in Figure 3B by OOM concentrations. This visualization could help verify the role of OOMs in nucleation: if OOMs are indeed critical to the process, one would expect to see a vertical trend associated with OOM levels in Figure 3B. Conversely, if no pattern emerges, OOMs might simply be co-emitted with other vapors without driving the nucleation event itself. Including this analysis as a new SI figure would allow the discussion in this section (and elsewhere in the paper) to be grounded in observational evidence rather than relying heavily on uncertain parameterization calculations.

Comment 7:
I also feel that Reviewer 5's suggestion to link current observations to the CCN budget, as well as to cloud and radiative forcing (presumably via global modelling), falls outside the scope of this study.

Comment 8:
The authors should acknowledge that their acid measurements were conducted under considerably more variable conditions compared to controlled laboratory experiments, which inherently introduces greater uncertainty. Additionally, the calibration procedure for sulfuric acid represents a significant source of error. A factor-of-two uncertainty in acid concentrations can propagate non-linearly, readily resulting in an order-of-magnitude difference in derived aerosol nucleation rates. With this consideration, the data presented in this study are not necessarily in conflict with CLOUD data; on the contrary, given the aforementioned uncertainties, the agreement is actually quite reasonable.

Minor:

CCN is written as 'Cloud Condensation Nuclei (CCN)' in the abstract, but as 'cloud condensation nuclei (CCN)' in the introduction. This is a minor issue, but it would be good to be consistent with capitalization (also for other terms).

Line 87: I think Baccarini et al. (2020) reports both iodine NPF and growth? Could the authors double check?

Line 93: "...not necessarily to new particle formation"

Figure S3: fix the caption of panel D

Lines 158-161: is the correspondence of numbers and classification correct? Should the NPF events be with 0.0005 s-1 CS? Please clarify.

Lines 185-187: Please consider rephrasing this sentence, as it feels somewhat congested. In addition, 'power-law relationship' and 'non-constant ratio conveys somewhat similar concepts.

Line 193: CLOUD chamber, Mace Head and Ny-Ålesund. Also, where is the solid red line? I don't see that in the panel A? Is it the dashed cyan line?

Lines 205-207: is it really true that J1.7 has not been reported in the Arctic? I'm sure that things like J1.5-J1.8 have been reported for example in Beck et al. (2021).

Figure S8: I suggest remove the Almeida data from the comparison. Instead, use the data from Xiao alone or else from Kürten et al. (2018; 10.5194/acp-18-845-2018), which primarily provides a correction to the Almeida et al. (2013) dataset (which is not precise by current standard). For the reasons and details, see Kürten et al. (2018). Few other reviewers also mention this and the responses by the authors are not really convincing... To be frank, the Almeida data is simply not correct and should not be used anymore.

Lines 262-266; 334-336: these seem interesting. Is there a way we can speculate the source of I-OOM? Is it possible that they source from the particles through heterogeneous reaction of organics and iodide/iodate, instead of the other way around? If this is the case, there growth contribution needs to be questioned.

Line 291: ... primary particle growth drivers.

Lines 295-296: these are overstatements - it may be true to the I-OOM. However, other studies have carried out in simple terms by comparing OOM concentrations with GR. These also provided molecular information in the MS. If the statement refers to using the VBS method, please be specific since what "molecular-level" means is rather vague and can easily trigger debates.

Lines 304-306: define MIZ when first appears.

Lines 396-398: I feel the discussion about monoterpene is a bit biased. In the plot, even after scaling of a factor of 10,

monoterpene concentration is still minor. The authors should report the real concentration here as the scaling is a bit confusing as well. The authors should also quite frankly say that it is not so important from their measurements.

Lines 441-443: I think whether this is a warming or cooling in the Arctic is far from clear. Or would the authors argue differently?

Figure S9, the colors of the same peak in panel A and B,C,D should be consistent.

Table S1 07/06/2022 data miss placed

The authors claimed that their results of OOM new particle formation is measured and supported by global modelling. I do not see the global modelling results from this study? Or is it supported by other studies?

Reviewer #7

(Remarks to the Author)
The manuscript by Du et al. presents ship-based measurements conducted from southeastern to western Greenland to investigate atmospheric new particle formation (NPF) in this understudied region. The measurement campaign employs an impressive and comprehensive suite of instruments, spanning precursor gas measurements, molecular cluster observations, particle-phase measurements, and cloud condensation nuclei (CCN) observations. This unique combination enabled the authors to follow the NPF process from its molecular origins through particle growth and directly to CCN formation. According to the authors, these measurements serve as in-situ evidence for HIOx/H2SO4 enhanced nucleation, corroborating previous findings from the CLOUD chamber study by He et al. (Science, 2023). In addition, the study identifies a new class of iodine-containing oxygenated organic molecules (OOMs), which plays a role in particle growth, while speculating that they may also influence early-stage nucleation. The study further demonstrates that the marginal sea-ice zone is associated with elevated concentrations of precursor gases and acts as a significant source region for CCN production.
Overall, the manuscript reports potentially important observations of particle formation and growth that are not readily explained by the measured concentrations of known acidic precursors, together with the identification of a class of iodine-containing OOMs that has not been previously reported. While these findings make the manuscript suitable for publication in Nature Geoscience, several issues must be addressed before the manuscript can be accepted for publication. These are outlined below.
In addition, the authors are encouraged to substantially improve the quality of the Supplementary Material and to correct multiple inconsistencies between the figures and their captions. The Supplement should aid the reader in understanding the results, rather than creating uncertainty about what is being shown.

General comments:

Comment 1: The authors did not directly measure particle number concentrations at 1.7 nm and therefore could not derive the particle formation rate at 1.7 nm from observations. Their measurements using the NAIS extend down to approximately 3 nm. To overcome this limitation, the authors first derived the formation rate at 4 nm (J4) from NAIS measurements after scaling the NAIS size distribution to match that of the nanoMPSS. They then inferred J1.7 from J4 using the survival probability formulation of Cai et al. (2022). It is great to see that the authors employed the most recent survival probability framework. However, because J1.7 is inferred rather than directly measured, the uncertainty associated with this calculation should be quantified and explicitly reported. Cai et al. (2022) demonstrate that a systematic difference in growth rate by a factor of three can lead to changes in the median survival probability exceeding two orders of magnitude. Given this strong sensitivity and considering that the inferred J1.7 values are used to benchmark the campaign observations against CLOUD experiments, it is essential that the J1.7 data presented in Figure 3 include uncertainty estimates. The authors are therefore encouraged to propagate uncertainties in the parameters of the survival probability equation (e.g., scaling factor between NAIS and nano-MPSS, growth rate and coagulation sink of small particle sizes) and assess how these uncertainties affect the derived J1.7 values. These uncertainties should be reflected as error bars or shaded ranges in Figure 3.

Comment 2: The authors emphasize that their measurements provide direct in situ evidence that HIOx/H2SO4 clustering enhances nucleation rates in the real atmosphere, thereby validating the CLOUD results of He et al. (Science, 2023). Upon first reading, this claim led me to expect either (i) formation rates (J1.7) comparable to those reported by He et al. , or (ii) the observation of clustering behaviour analogous to that reported in the CLOUD experiment. However, the results section shows that the J1.7 values reported in this study (at a mean temperature of ~1.2 °C) are in fact higher than would be expected from iodine oxoacid/H2SO4 nucleation alone at this temperature and are instead comparable to the J1.7 values reported by He et al. at –10 °C (Fig. 3b). The authors interpret these elevated formation rates as likely resulting from additional enhancement by DMA and/or oxygenated organic molecules (OOMs). While this interpretation is plausible, it implies that the observed nucleation cannot be attributed to HIOx/H2SO4 clustering alone, and therefore does not constitute a direct confirmation of the CLOUD results. The second line of evidence used to support the authors' claim is the mass defect (MD) analysis shown in Figures 2 and S6. However, these plots reveal only two mixed iodine–sulfuric acid cluster (HIO3HSO4- and HIO2·H2SO4·HSO4-) which appears insufficient to support a claim of direct observational proof of HIOx/H2SO4 -driven nucleation. This limitation is associated with the fact that the MD plots are derived solely from nitrate CIMS measurements of neutral clusters. An observation of a broader suite of mixed sulfuric acid–iodine oxoacid clusters

(which are typically detected as naturally charged clusters using an APi-TOF) is what in fact would give the direct molecular evidence HIOx/H2SO4 -driven nucleation. For example, the CLOUD study by He et al. shows clear sequential clustering of sulfuric acid–iodine oxoacid species extending to masses exceeding 600 Th. Similar sequential growth of iodine-containing clusters to high molecular weights has also been reported in Arctic field measurements at Villum by Beck et al. (2021; their Fig. 3) and by Baccarini et al. (2020; their Fig. 1). These studies reported multiple mixed iodine–sulfuric acid clusters, including HIO3HSO4SO3-, HIO3HIO2IO3H2SO4-, and (I2O5)1-2HSO4- . Baccarini et al. further reported clusters such as I2O5HIO3HSO4- , (I2O5)2HIO3HSO4- and (I2O5)3-4HSO4-.
Therefore, while the present study is indeed original in reporting J1.7 measurements and comparing them to the CLOUD measurements, it is not the first to provide in situ evidence of HIO_x/H_2SO_4 clustering in the atmosphere. Such clustering has already been observed in previous field studies. The authors' claims on validating CLOUD measurements, should therefore be rephrased to align with the existing literature and to more accurately reflect the specific novelty of the present work.

Comment 3: In relation to Comments 1 and 2, the reported J1.7 values appear high, given that the combined concentrations of sulfuric acid, iodic acid, and methanesulfonic acid remained below ~5e6 cm-3 during the measurement period. Specifically, the reported formation rates (0.03–0.8 cm-3s-1) exceed those previously reported for Ny-Ålesund, including J1.5 reported by Beck et al. and J3 measured at the Zeppelin station by Lee et al. (2020; https://doi.org/10.5194/acp-20-13425-2020). The authors attribute these elevated J values to the presence of DMA and OOMs; however, the evidence supporting their involvement remains weak. It is understandable that, due to the inherent difficulty of quantifying low DMA concentrations with a nitrate-CIMS, neither the absolute DMA concentration nor its day-to-day variability during NPF events can be robustly constrained. This limitation makes it difficult to assess whether DMA alone can account for the observed magnitude and variability of J1.7. At the same time, a substantial fraction of OOMs was quantified in this study. Given this, it would be useful to explore more explicitly how these compounds relate to the variability in J1.7. The authors performed a PMF analysis of the OOM measurements and derived a volatility basis set; however, it remains unclear how these PMF factors relate to the measured formation rates. At present, a qualitative inspection of the mass-defect plots, together with comparisons of J across different days, does not reveal a clear link between enhanced cluster abundances and higher nucleation rates, despite Fig. 2 trying to suggest that nucleation is associated with high abundance of clusters. In particular, days with elevated cluster signals do not necessarily correspond to higher J values. Have the authors attempted to correlate the PMF factors or volatility classes directly with the measured J values?
Finally, the NPF event on 14 June appears peculiar: although its J value is comparable to those of other events, the mass-defect plot shown in Fig. S6 exhibits substantially fewer clusters than on other nucleation days. The authors are encouraged to comment on possible reasons for this behaviour and its implications for the proposed nucleation mechanism.

Comment 4: In relation to particle growth and the growth modelling presented in Figure S17 and partly in Figure 4, the authors conclude that oxygenated organic molecules (OOMs), including iodine-containing OOMs (I-OOMs), enhance particle growth to sizes relevant for CCN. However, the modelled growth rates based on acid and organic condensation are, in most cases, unable to fully reproduce the measured growth rates. While this discrepancy may partly arise because growth rates derived from particle number size distributions represent apparent growth rates rather than true condensational growth rates, an additional mechanism may also contribute to the enhanced growth observed. In particular, Huang et al. (2022) [https://doi.org/10.1073/pnas.2201729119] demonstrated that iodine-oxide nanoparticles exposed to organic vapours can undergo accelerated growth through heterogeneous reactions. Could the authors discuss whether such heterogeneous chemistry involving iodine-containing particles and organic vapours may contribute to the enhanced particle growth observed here, especially during periods when the modelled condensational growth underestimates the measured growth?

Comment 5: The authors mainly attribute the enhanced particle growth observed on 8 June to elevated aldehyde concentrations measured by the PTR-MS (Lines 342-344 and the discussion of Fig. 4: Lines 304-323). However, the PMF analysis of the nitrate-CIMS OOM data suggests a different interpretation. Specifically, inspection of the PMF factor time series in Fig. S2c (and Fig. S13) shows that the increase in the aldehyde-rich factor on 8 June coincides with a relatively stable particle mode diameter, indicating limited subsequent growth. In contrast, during periods dominated by the monoterpene-related factor, particles continue to grow to diameters exceeding 100 nm. This apparent discrepancy between the nitrate-CIMS-based PMF results and the PTR-MS gas-phase VOC measurements warrants careful consideration, as it leads to differing conclusions regarding which compound classes are primarily responsible for growing the particle up to CCN size.

Specific Comments:

Comment 6: L49-52: "Our findings demonstrate that iodine, sulfur, and organic precursors in the Arctic enhance the abundance of CCN, through new particle formation, that can potentially influence the earth's cloud cover, radiation balance, and hydrological cycle."
The authors should consider tempering this claim by noting that, while the results indicate a potential enhancement of CCN abundance, the magnitude of this effect and its climatic relevance is yet to be quantified.

Comment 7: L122: Figure 1.
Please clarify the criteria used to select the events displayed in Panel A and explain why the remaining events were omitted, or include all of them.
Second, it is difficult for the reader to relate the events shown in Figure S2 to those presented in Figure 1, as the map does not provide any information on the timing of the measurements. To improve clarity, the authors could consider encoding the measurement date using a colour scale, while using marker shape to distinguish between NPF and burst events, rather than using a single fixed colour for event types.
Third, for readers who may not be familiar with Arctic geography, it would be helpful to indicate the Davis Strait marginal

sea-ice (melting) zone on the map.

Comment 8: L137-140: "Background concentrations of NOx (<50 ppt), SO2 (median 23 ppt) and BC (1.9 ng m-3) were very low, indicating pristine marine conditions."
These values are difficult to verify from Figure S3 as currently presented. Please revise the figure so that the stated concentrations can be clearly identified.

Comment 9: L140-141: "Based on the criteria detailed in Methods 10, nucleation events were observed on 13 days…"
What does 10 (after Methods) stands for?

Comment 10: L188-190: "Using this relationship, we applied the more readily observed HIO3 to estimate HIO2 concentrations when they were not detectable on 23rd and 24th May (see Fig. S2)."
Why is HIO2 not detectable on those days given that it was detectable in 10/06 at even lower HIO3 concentration?

Comment 11: L 191: Figure 3.
It would greatly improve readability if the colours of the markers referring to the CLOUD data were swapped, such that blue represents –10 °C and red represents +10 °C, rather than the opposite. This colour convention is more intuitive and would reduce the potential for confusion (I initially misinterpreted the data until reading the legend).

Comment 12: L205-207: "While J1.7 has not been reported elsewhere around the Arctic, our observed formation rate at 10 nm (J10) is within the observed range 10, although the median is relatively high (Fig. S7)."
Formation rate measurements at smaller particle diameters have previously been reported for Ny-Ålesund. In particular, Beck et al. reported formation rates at 1.5 nm while Lee et al. (2020) (https://doi.org/10.5194/acp-20-13425-2020) reported formation rates at 3 nm. A brief comparison of the present results with these studies would provide more insight into the underlying NPF processes than a comparison limited to J10 across multiple Arctic sites.

Comment 13: L 211: "Our experiments similarly do not match experiments with 4 pptv DMA and 10 ppb NH3"
The ammonia unit is incorrect

Comment 14: L350: Figure 4
Panel A: Although the back trajectories seem to converge at a certain point, it would improve clarity if the ship position is marked clearly on panel A. Remember to add the symbol "A" to this panel.
Panel F please indicate directly on the legend that the IOA trace is a PMF factor

Comment 15: L521: "Utilizing the data from the three MPSS systems, we produced a conjoined PNSD for the entire"
Please include to the methods section an explanation detailing how the NAIS particle size distributions were scaled to match the nano-MPSS data. It would also be helpful to report the range of scaling factors applied during the campaign and to comment on their variability and potential impact on the derived formation rates as requested earlier.

Comment 16: L 530-532: "In parallel, nitric acid vapor is volatilized from a glass tube containing liquid HNO3 and carried by a mixture of 3 cm3 min-1 N2 and 20 L min-1 pristine Arctic ambient air as a sheath gas".
Is this correct? Did you use ambient "uncleaned" air as sheath flow? If yes please explain how you made sure that this air is free of contaminants?

Comment 17: L 555:
Reference should be made to figure S9 instead of Figure S10

Comment 18: L 834-835:
The equation should be 2πD instead of 4πD when dp refers to the diameter of the particle rather than the radii. This was also a typo in the Kulmala et al protocol paper.

Comment 19: L 847: " Growth rate (GR) calculation"
It should be emphasized that the growth rates reported here represent apparent growth rates rather than true condensational growth rates. This is especially important given that the measurements were conducted onboard a moving ship. Growth rates inferred from diameter–time evolution in general reflect the net contributions of many processes that affect the modal diameter, including vapour condensation, coagulation, continuing nucleation and changes due to transport and dilution of spatially inhomogeneous aerosols.

Comment 20: L 912: "performed in the PMF2 program"
Does this refer to the EPA PMF program? If yes, why was an older version without a robust uncertainty analysis used instead of the later releases?

Comment 21: L 941-945: "The optimal solution was identified as a seven-factor model describing different kinds of organic aerosol (OA), consisting of hydrocarbon-like OA (HOA), cooking OA (COA), marine secondary OA (MSOA), sea salt OA, two types of semi-volatile OA (SVOA and SVOA1), and low-volatile OA (LVOA) based on time series, diel patterns, factor profiles (especially by representative markers), and correlation with other measurements in this campaign"
Why do the PMF factors referred to here differ from those presented in the main manuscript and in Section 5 of the Supplement?

Comments of SI Figures:

Comment 22: Figure S2:
o Panel A: while it is clear that the black circles are the fits from the maximum concentration method used to calculate GR, this is not explicityly mentioned in the caption. Please include it
o Panel B is supposed to show the solar radiation as a yellow shading but it is actually not shown
o Panel F: the legend refers to a CCN trace ar 0.7% saturation but this trace is not shown in the plot.
o All panels: Figure S2 shows nine pink-shaded areas corresponding to NPF events and four green-shaded areas corresponding to burst events, whereas the manuscript text and Table S1 refer to eight NPF events and five burst events. The authors should ensure consistent reporting of event numbers throughout the manuscript, figures, and supplementary material (same comment for Figure S3, S12, S13, S15).

Comment 23: Figure S3:
o Panel B: The y-axis scale is too large for the data presented in this panel. In addition, the caption refers to both CO and SO2, while only the SO2 time series is shown.
o Panel C: It is hard to see the baseline BC from this plot. Can the y-axis be cut to a certain value as done with the other sub plots or plot the data in log scale.
o Panel D: Remove the unneeded characters ($, ^) from the legend. Adjust the inconsistency between the figure caption and the y label (one refers to a signal while the other refers to a mixing ratio)

Comment 24: Figure S4: Reference is made to Extended Data Fig. 1 which does not exist.

Comment 25: Figure S6: It seems the panels are sorted by J1.7 rate instead of date. It would actually be helpful to mark the J and GR measured for these events on the subtitles of the subplots.

Comment 26: Figure S7: The figure caption contains several inconsistencies with the plotted data. Growth rates from other locations are described as being shown in grey boxplots, whereas the boxplots in the figure are coloured. In addition, the DY151 data are indicated as blue boxplots, which is not the case in the figure. The caption also refers to a red line that is not present in the plots. Finally, the caption mentions a location labelled “TIK,” which does not appear in the figure and may refer to Tiksi; this should be clarified.

Comment 27: Figure S15: caption of panel (a) refers to Fig. 3 while the correct figure number is 4.

Comment 28: Figure S20: The figure caption refers to purple circles, grey lines, and green and pink crosses; however, these elements are not shown in the figure.

Version 2:

Reviewer comments:

Reviewer #6

(Remarks to the Author)
The authors have convincingly addressed all of my major comments. I recommend publication subject to minor revisions.

Minor comments:

Lines 235–236: It would be preferable to explicitly state the CS values for both this study and the CLOUD experiments, so that readers can assess the difference quantitatively. It is also worth emphasizing that CS has a non-linear effect on J.

Lines 237–241: This issue carries over from the previous revision. The DMA discussion requires additional constraints, consistent with the texts in other parts of the manuscript. The QC data are of interest, but their value is contingent on the reliability of the underlying measurements.
The expanded discussion of the correlation between OOMs and J is very helpful and effectively clarifies OOM's contribution.

Line 375: HOI is the primary form and should be stated explicitly for clarity. Please also add it to Figure 5 to avoid ambiguity when it is referenced later.

Lines 386–393: This is a valid and well-made point - earlier studies may indeed have missed an important opportunity. For instance, the NH3 concentrations in Beck et al. (2021) were back-calculated, which suggests the present study is not necessarily in conflict with some prior work. A sentence or two acknowledging that certain earlier studies may have lacked the opportunity to detect this directly would strengthen the discussion.

Reviewer #7

(Remarks to the Author)

I have reviewed the revised (Round 2) version of the manuscript by Du et al. The authors have addressed my comments and

have made substantial improvements to the manuscript.
In particular, the authors have moderated claims that were previously not fully evidence-supported, improving the overall balance and credibility of the study. They have also better placed their findings in clearer context with the existing literature. They incorporated the requested uncertainty analysis for the J1.7 measurements, which strengthens the comparison of their results to the He et al study. In addition, the manuscript has been effectively restructured, with key material now presented in the main text and supporting details appropriately moved to the Supplementary Information.
Overall, the revisions have enhanced the clarity and rigor of the work, and I recommend it for publication.

Open Access This Peer Review File is licensed under a Creative Commons Attribution 4.0 International License, which permits use, sharing, adaptation, distribution and reproduction in any medium or format, as long as you give appropriate credit to the original author(s) and the source, provide a link to the Creative Commons license, and indicate if changes were made.
In cases where reviewers are anonymous, credit should be given to 'Anonymous Referee' and the source.
The images or other third party material in this Peer Review File are included in the article's Creative Commons license, unless indicated otherwise in a credit line to the material. If material is not included in the article's Creative Commons license and your intended use is not permitted by statutory regulation or exceeds the permitted use, you will need to obtain permission directly from the copyright holder.
To view a copy of this license, visit https://creativecommons.org/licenses/by/4.0/

General Response to Reviewers' Comments

We thank the reviewers for their thorough and constructive comments. These comments helped us to revise the manuscript, better situate our work within previous research, clarify the knowledge gaps that motivated the study, and more clearly distinguish between results that are directly supported by the data and those that are inferred.

1. In-situ evidence of HIO_x/H_2SO_4 clustering enhancing nucleation rates in real atmosphere, confirming the CLOUD study by He et al. (Science, 2023) [1]

1.1 Previous research from which this study built upon, and the research gaps

Chamber studies: He et al. (Science, 2023) [1] demonstrated in the CLOUD chamber that HIO_x dramatically enhances H_2SO_4–NH_3 nucleation, by orders of magnitude, and proposed that this mechanism is atmospherically relevant based on observed ambient HIO_x.
Gap: *While demonstrated in the CLOUD chamber, direct in-situ molecular observations have yet to confirm its occurrence in natural environment where concentrations are typically much lower than chamber conditions.*

Field observations: Across the Arctic, dominant nucleation precursors vary by site and season — e.g., HIO_3-driven events over pack ice in autumn [2]; H_2SO_4–NH_3 nucleation at Svalbard in spring/summer; and iodine-driven in spring and H_2SO_4–NH_3 in summer at Villum [3].
Gap: *Existing field observations have predominantly identified binary nucleation pathways. Direct in-situ evidence for multi-component nucleation (i.e., involving more than two chemical precursors), including the role of HIO_2 in cluster formation, has not yet been reported.*

1.2 What this study found

1. We provide direct, in-situ molecular evidence of HIO_x–H_2SO_4 clusters (e.g., HIO_3–H_2SO_4, HIO_2–HIO_3, HIO_2–H_2SO_4–H_2SO_4) during multiple Arctic NPF events, confirming that this synergistic pathway operates in the real atmosphere — even at HIO_x concentrations much lower than in CLOUD experiments.

2. We showed that our measured nucleation rates were greatly enhanced compared to those expected based on nucleation mechanisms involving H_2SO_4 or HIO_x alone, which is consistent with HIO_x-H_2SO_4 formation mechanism proposed by He et al. (2023) [1] though the observed rates are typically more than an order of magnitude larger than predicted by this mechanism alone.

3. We detected a new DMA-containing cluster (C_2H_7N–HIO_2–HIO_3).

1.3 New insights and understanding this study advanced

1. **Atmospheric validation of a major new pathway**: Our direct molecular evidence in the real atmosphere confirms the HIO_x–H_2SO_4 nucleation mechanism in a climatically sensitive region, closing the key evidential gap following He et al. (2023) [1]. As seen previously when Yao et al. (2018, Science) [4] validated Almeida et al. (2013, Nature) [5]. *Consequently, our findings establish the relevance of this mechanism in the real atmosphere beyond the CLOUD chamber.*

2. **Demonstrating atmospheric importance under low-HIO_x conditions**: HIO_x enhances nucleation rates by orders of magnitude even at concentrations much lower than used in CLOUD, indicating that the mechanism is relevant under a wide range of real-world conditions. *This supports inclusion of iodine chemistry in Arctic aerosol/climate models.*

3. **Revealing additional enhancement beyond existing mechanisms:** Observed nucleation rates exceed CLOUD-based predictions, and detection of DMA-iodine clusters and OOMs suggests additional multi-component chemistry involving amines and/or organics. *This highlights realistic atmospheric*

complexity; consequently, relying on existing laboratory parameterisations only may underestimate particle formation rates.

2. In-situ molecular evidence of new classes of OOMs contributing to new particle growth in an Arctic field campaign

2.1 Previous research from which this study built upon, and the research gaps

The existing body of literature has led to the prevailing hypothesis that oxygenated organic molecules (OOMs) facilitate particle growth to cloud-condensation nuclei (CCN) in the Arctic (hypothesis 1) [3, 6-10]. On the back of recent measurements, some also hypothesised that the OOMs originate from the ocean, with abiotic production of aldehydes from marine fatty acids as potential precursors (hypothesis 2) [11-13].

***Gap: Direct molecular-level evidence from in-situ measurements has been lacking**, preventing full validation of hypothesis 1. No aldehyde OOMs have been directly detected outside of the laboratory nor shown to directly contribute to particle growth anywhere in the globe (testing hypothesis 2).*

2.2 What this study found:

1. We provided direct molecular-level, in-situ evidence that new particle growth in the Arctic is driven by OOMs, supported by growth modelling.
2. We identified molecular signatures consistent with aldehyde-derived OOMs, indicating a pathway from ocean-derived aldehydes to particle growth.
3. We detected 90 iodine-containing OOMs (I-OOMs), representing over 10% of the total number of observed OOMs.

2.3 New insights and understanding this study advanced

1. **Validating a long-standing hypothesis:** We provided direct observational basis evidence to support the hypothesis that new particle growth is driven by oxygenated organic molecules (OOMs) in the study region. *This closes an observational gap central to understanding growth of new particles in the Arctic.*
2. **Supporting the hypothesized ocean-to-atmosphere chemical pathway:** We found a critical role of aldehydes, mostly likely abiotically emitted, in OOM formation, highlighting the overlooked significance of these emissions in current models. *This improves mechanistic understanding of marine biogenic influences on Arctic new particle formation.*
3. **Discovering iodine-containing OOMs:** This suggests formation pathways not known currently, as iodine is typically not considered a contributor to OOM formation. These I-OOMs contribute an estimated ~9–36% of particle growth and subsequently enhance CCN concentrations by virtue of increasing particle size. *This opens a new direction in Arctic OOM and iodine chemistry.*

3. Full coverage of the new particle formation (NPF) processes at molecular level (from molecular components contributing to nucleation, to growth, and to observed CCN) in an Arctic field observation

3.1 Previous research from which this study built upon, and the research gaps

1) NPF → CCN link in the Arctic: Arctic observations suggest that NPF can contribute to CCN. Prior studies tracked the growth of particles as small as 5 nm to CCN while measuring the composition of aerosols once they grow to be >70nm (by aerosol mass spectrometer, AMS). Other studies observed the nucleation and subsequent growth of particles to CCN-relevant sizes (i.e., particles reached diameters that *could* serve as CCN under certain supersaturations; e.g., 30 nm) and indirectly linked CCN formation to nucleation [14].
***Gap:** No study has followed the same particle population from molecular cluster formation through growth to directly measured CCN at the molecular level.*

2) Marginal ice zone as a NPF precursor source: Melting ice has been hypothesised to lead to atmospheric NPF in the Arctic.
Gap: *no direct in-situ evidence is available to link sea-ice-associated precursor emissions to CCN formation.*

3.2 What this study found:

1. **Full coverage of NPF processes**: We provided the first molecular-level observational sequence of the full NPF process in the Arctic — from VOCs and cluster formation, through particle growth, to directly measured CCN. The order-of-magnitude increase in CCN concentrations during two NPF events demonstrates that NPF substantially enhances secondary CCN.
2. **Role of marginal ice zone**: We documented nucleation, growth and subsequent CCN formation near the ice edge while air masses travelled over the ice edge for >12 hours, linking emissions from ice and melting ice zone and the ocean to CCN formation.

3.3 New insights and understanding this study advanced

1) Directly confirming the NPF–CCN connection: By tracking molecular clusters and particle growth to measured CCN in the same air mass, we provided unambiguous in-situ evidence that NPF directly contributes to CCN in the Arctic atmosphere, strengthening and extending earlier indirect observations. *This empirically validates the process linking oceanic emissions to cloud-relevant aerosols.*

2) Demonstrating a marginal sea ice zone-linked CCN source: Our observations provided direct support for the long-standing hypothesis that the ice margin is a source of NPF precursors forming climate-relevant aerosols. *This identifies a climate-sensitive source of cloud-modifying particles in a rapidly changing Arctic.*

Together, these findings establish the first fully resolved molecular-to-CCN pathway in the Arctic atmosphere, confirming a key iodine–sulfur nucleation mechanism, revealing previously unrecognized growth chemistry involving aldehyde and iodine, and demonstrating that marginal sea ice zone emissions fuel CCN formation in a rapidly changing climate. Therefore, they provide firmly supported and novel insights into new particle and CCN formation in remote marine environments, a process of central importance to understanding natural aerosol–cloud–climate interactions.

Importantly, while CLOUD chamber studies have been pivotal in identifying and parameterising new nucleation mechanisms, as demonstrated by He et al. (2023), the atmospheric relevance and significance of such mechanisms ultimately need to be evaluated against real-world atmospheric observations. Our dataset provides, for the first time to our knowledge, the molecular- to-CCN coverage needed for such an evaluation, enabling direct comparison between model representations and the observed sequence of nucleation, growth, and CCN formation. This offers an observational dataset for model validation and a quantitative basis for reassessing the magnitude of iodine-mediated particle and CCN formation, and resulting impacts on clouds and climate, in atmospheric models.

Du et al., Response to reviewer comments

We have thoroughly reviewed all comments, provided a point-by-point response, and revised the manuscript accordingly. Below, we include the reviewers' comments in black, our responses in blue, and any manuscript text in orange. Where edits are made to a larger section, we will include the full section**.**

Reviewers 1 & 2

General comments:

The manuscript by Du et al. reports ship-based measurements of NPF and growth processes in the Arctic. A comprehensive set of instrumentation was operated, which enabled the authors to follow the NPF and growth processes from precursor level up to CCN size. The authors describe multicomponent nucleation from HIO_3, HIO_2, H_2SO_4 and dimethylamine (DMA), as well as aerosol growth driven by oxidized organic molecules (OOMs) including iodine-containing OOMs (I-OOMs). They provide a comprehensive set of analyses and figures to support their findings.

There are several new aspects of this manuscript that did so far not appear anywhere else:

1) The confirmation in the real-world atmosphere of recent findings of the CLOUD experiment regarding iodine oxoacid NPF.
2) The measurement of I-OOMs, which are reported to contribute to aerosol growth.
3) The full coverage of the NPF processes (from molecular components contributing to nucleation up to CCN size) in an Arctic field measurement.

These points do represent important findings, which could, in principle, make it suitable for a publication in Nature or a sister journal like Nature Geoscience or Nature Communications. However, in its current form the manuscript is not ready for publication. There are two major points about the data interpretation with respect to the chemical system responsible for nucleation, where we (this review was prepared by two reviewers from the same research group) do not agree with the authors.

1. Why was the potential role of the 591 different OOMs not considered to participate also in the nucleation process?
2. Instead, DMA is repeatedly claimed to participate in the nucleation, but the evidence for DMA detection is weak (one single "peak", which is unambiguously detected only a few short times (e.g., Fig. S4 Period 1), but it is usually only detected as the broadened flank of a much larger peak and it could easily be misfitted or misidentified (ED Fig 3).

These two points are discussed in more detail below.

Furthermore, while the authors undeniably put a lot of work into the manuscript, it still suffers from a lack of care for detail and it seems that its submission was rushed a bit. However, after the authors address the two main points and all the minor issues, we would be willing to review a revised version of the manuscript.

General response: We thank the reviewers for their thorough and constructive feedback on our manuscript. Below, we address the comments point-by-point.

Comment 1: The whole manuscript mentions the organics identified by nitrate-CIMS (OOMs) only in the context of growth, never in the context of nucleation. However, Fig 1C (concentration of OOMs typically 1 order of magnitude larger than H_2SO_4, MSA, HIO_3), the NPF MD plot (Fig 2A shows the presence of rather large amounts of high-m/z OOMs), and the identification of numerous OOM compounds as ELVOCs (the ELVOCs are responsible for more than a third of the growth in the growth model of ED Fig 7) make a role of the OOMs for nucleation very plausible. You state, based on Fig 3b that your nucleation rates agree with the (H_2SO_4+HIO_3)*HIO_2 parametrization, but you also mention that your J values are a bit higher than CLOUD values for similar conditions. Could you please discuss in how far this opens up the possibility of an ELVOC contribution to nucleation? Could the following be occurring: Either a purely OOMs-driven nucleation (similar to the nucleation of HOMs described by Kirkby et al., Nature, 2016), or an OOMs-driven nucleation boosted by inorganic acids (similar to the nucleation of isoprene-OOMs boosted by H_2SO_4 or HIO_3 as described by Shen et al., Nature, 2024)? Furthermore, due to the fact that the exact role of iodine for reducing the volatility of OOMs is not known, some of the I-OOMs could be ULVOCs, and therefore even more likely to participate in the initial nucleation. To us, such an organic-driven nucleation pathway is quite plausible. At least, it should be discussed, why it is excluded by the authors. Part of the discussion should be to add a plot showing ELVOC concentration vs. J rates. It should also be considered to plot the ELVOC fraction of the OOMs in Fig. 1 (in addition to the total OOMs).

We understand the value of Fig. 2 as an average over all NPF events to derive an MD plot, but this might mask the variability of individual NPF events (which can be somewhat inferred from ED Fig. 3). For example, you could also get the same MD plots as in Fig 2 if for the first half of your campaign you would only have iodine-sulfur-driven NPF and in the second half only OOMs-driven NPF. While you provide numerous figures as ED and supplementary material, you only provide the three MD plots shown in Fig. 2. Can you please provide some additional MD plots that illustrate individual NPF events. We would be especially interested in one or several MD plots that cover the event shown in Fig. 4. The data in Fig 4 is an impressive measurement, but detailed MD plots would shine more light on this discussion.

Response: We thank the reviewers for this comment. We agree that the potential role of oxygenated organic molecules (OOMs) in the initial nucleation step is an important aspect to consider, and that several pieces of evidence in our dataset make this a plausible pathway. In fact, to quote a recent modelling preprint from the *Archibald* group at Cambridge: "*The persistence of low aerosol number concentrations, despite overestimated precursors, suggests missing formation pathways likely involving other species such as iodine, amines, or **organic vapours***" [15] (emphasis our own). There are real world examples of OOMs (or, HOMs) nucleating in the absence of high concentrations of inorganic acids [16-18], alongside chamber studies showing that HOM dimers can nucleate efficiently by themselves [19, 20], as well as enhance the nucleation rate of sulfuric acid-amine systems [21, 22].

Our initial focus on the inorganic pathway was driven by two primary lines of direct evidence from our measurements. Firstly, the most unambiguous signals for clusters contained inorganic species (H_2SO_4, HIO_3, HIO_2). While OOMs were abundant in the gas phase, their direct participation in the smallest clusters was less clear compared to the signals of inorganic acid clusters. However, we must

acknowledge that sulfuric acid-OOM cluster measurements are rare in NO_3^- CIMS data due to the high numbers of OOMs and therefore potential clusters. Secondly, as shown in our original Figure 3b, the observed particle formation rates ($J_{1.7}$) indicated a consistency with with the data of He et al., (2023) [1] ((H_2SO_4+HIO_3)×HIO_2). This consistency, combined with the direct detection of inorganic clusters, promoted us to conclude that multicomponent inorganic nucleation was the dominant identifiable pathway.

Indeed, our observed J-rates are higher than those predicted by the pure HIOx-H_2SO_4 parameterisation from He et al. (2023) [1] at similar temperatures. We agree that this enhancement strongly suggests the participation of other species. While we proposed this could be due to stabilisation by dimethylamine (DMA), we agree that the high abundance of OOMs, particularly ELVOCs, makes them a plausible contributor to this enhancement. It is possible that OOMs are either participating directly in nucleation or stabilising the initial clusters just past 1.7 nm (which would still enhance $J_{1.7}$), a mechanism that would be consistent with findings in other environments.

To address this in the revised manuscript, we make the following arguments (Line 227-240 in the revised new manuscript – without track changes; same for all following resposnes)

"Considering the average ambient temperature of 1.4 °C during nucleation periods, the observed $J_{1.7}$ values are higher than anticipated from the temperature dependence measured in the CLOUD chamber [17] (at -10 and +10 °C). This enhancement strongly suggests the participation of other stabilising species. One plausible contributor is DMA, as evidenced by the detection of DMA clustered with HIO_x (Figs. S5-6 and Fig. S9). The DMA-HIO_2-HIO_3 cluster signal correlates with HIO_3 concentrations, and is highest when HIO_3 is at its maximum (Figs. S9-10). The role of DMA in enhancing nucleation rate is corroborated by quantum chemical modelling studies showing that DMA can accept a proton from HIO_3 as a base, forming a stable cluster (DMA-HIO_2-HIO_3) [37].

Furthermore, given the high abundance of OOMs, particularly ELVOCs (Fig. 1C), it is also plausible that they contribute to the enhancement of nucleation rate, either by participating directly in nucleation or by stabilising the initial inorganic clusters. This would be consistent with studies from other environments showing OOMs can enhance nucleation rates [38]. Furthermore, as the volatility-reducing effect of iodine in our observed I-OOMs is not fully constrained, it is possible that some of the observed I-OOMs are ULVOCs, which may participate in the initial nucleation."

Regarding our mass defect plots, we agree: averaging mass spectra can mask important event-to-event variability, and that it is important to examine the consistency of the mechanism across individual events. To address this, we have generated mass defect plots for individual NPF events, including the event on 7th June (Fig. 4), which are now presented in Fig S6. We interpret these alongside our detailed, day-by-day peak-fitting analysis in Fig. S5. This analysis confirms that the synergistic iodine-sulfur mechanism is a persistent feature of NPF in our study region, although the specific clusters detected vary between events, likely due to fluctuating precursor concentrations and the analytical interference of adjacent OOMs.

Our key finding from this analysis is that on nearly every NPF day, we observe a clear signal for at least one key cluster containing both HIOx and sulfuric acid species. For example, the $HIO_3 \cdot HSO_4^-$ dimer is clearly present on five separate days, while the $HIO_2 \cdot H_2SO_4 \cdot HSO_4^-$ trimer is also detected

on several occasions. The strongest and most complete set of cluster signals was observed on 1st June, coinciding with the highest measured HIO_3 concentrations.

Crucially, this analysis also provides insight into the major growth event of 7th June (Fig. 4). On this day, high concentrations of OOMs created some interference, making some clusters difficult to resolve, such as the $HIO_3{\cdot}HSO_4^-$ dimer. Despite this, we were still able to identify clear signals for several key species, including the $HIO_2{\cdot}H_2SO_4{\cdot}HSO_4^-$ trimer and a potential signal from the $C_2H_7N{\cdot}HIO_2{\cdot}IO_3^-$ cluster. This provides strong evidence that the multi-component mechanism was indeed active during the period of most significant growth to CCN sizes. We did not find evidence here for OOM involvement, although as acknowledged above, that is not necessarily something expected with the NO_3^- CIMS.

To make this point clearer for the reader, we will revise the main text to better reflect this day-by-day evidence (line 170-176):

"A day-by-day analysis (Fig. S5-6) reveals that while the detectability of specific clusters varies with precursor concentrations and analytical interferences, the synergistic iodine-sulfur mechanism is a persistent feature. On nearly every NPF day, we observe a clear signal for at least one key cluster containing both HIOx and sulfuric acid (e.g., the $HIO_3{\cdot}HSO_4^-$ dimer or the $HIO_2{\cdot}H_2SO_4{\cdot}HSO_4^-$ trimer), confirming the consistent co-involvement of both precursor systems. For example, during the event on 7th June, we identified the key multi-component clusters HIO_2-HIO_3-NO_3^- and HIO_2-H_2SO_4-HSO_4^-."

Comment 2: The second main point is the role of amines, here dimethylamine, for the multi-component nucleation that you propose. You state that you did not measure amines directly even though the nitrate-CIMS can, in principle, measure DMA. Did you do a calibration for the nitrate-CIMS for DMA and, if so, what would be the limit of detection for your instrument? However, if the DMA is in the clusters, why did you not see it directly and why did it not show up in more clusters (e.g., together with H_2SO_4)?

On the other side, the question arises, whether the DMA-HIO_2-IO_3^- cluster is actually real or a subject to noise fitting? From ED Fig. 3, where the last column shows this peak, we are not confident that this peak is real. It is strongly masked by the much higher peaks to the right. On the other hand, this is an average over at least several hours and maybe the peak is sometimes not masked if it only appears shortly? Please indicate for how long you did average in ED Fig. 3. However, if the uppermost row of this figure represents the same data as shown in Fig. 2A (NPF MD plot), where the DMA cluster is shown with a rather large marker size indicating around 1 cps, then we do not understand how you derive this peak confidently from the data of the right upper panel of ED Fig. 3.

You also provide Fig. S4, which specifically addresses this cluster. Panel B of this figure is the only data where one can clearly state that this peak is present. Can you explain here, why the red DMA peak is around 1.5 times smaller than the cyan I-OOM peak in panel B, but it has almost identical count rate in panel A period 1? Something does not add up here.

You further provide ED Fig 5 and state in the section starting at line 518 that "(1) C_2H_7N-HIO_2-IO_3^- and IO_3^- raw signals are well correlated (R^2=0.74, Extended Data Fig. 5A); and (2) the time series of the two also matched well (Extended data Fig. 5B)". This is the same argument presented twice. Of

course, if the time series match well, we have a nice correlation, so this is only one argument and not two. Furthermore, this correlation of data could have a different reason: Let's assume the DMA peak is not real and what you are actually fitting is mainly the much larger neighboring I-OOM peak, then one can expect that an I-OOM peak shows a good correlation with the IO_3^- signal.

So, overall, the evidence for DMA contribution to base stabilization of acid clusters is not convincing. Especially compared to the HIO_2 contribution, which clearly shows up in your peak fits. In your abstract and other statements, you put DMA and HIO_2 on an equal footing. Given the evidence you provide, this is not justified.

Response: We thank the reviewers for this analysis of the evidence for DMA's involvement. We agree that the signal for the DMA-containing cluster is small and that we should not place DMA on an 'equal footing' with HIO_2 in our original text.

As the reviewers note, there are specific periods where the C_2H_7N-HIO_2-IO_3^- cluster is unambiguously detected (e.g., Fig. S9). While the signal is not consistently strong, its clear appearance at the precise expected mass-to-charge during NPF provides compelling evidence for its presence.

Without the detection of this cluster, we would not have the confidence to drive the concentration of this cluster during the study period – as pointed out by the reviewer, in the averaged mass defect plot (Fig. 2A and Fig. S5 and 6), the signal for the C_2H_7N-HIO_2-IO_3^- cluster is small and the interference from a neighbouring I-OOM peak would have made it challenging to confirm whether this cluster is real or due to noise fitting. As pointed out by the reviewers, averaging over long periods where precursor concentrations vary can indeed obscure small signals.

We agree with the reviewers that the correlation plot and the time series in original Extended Data Fig. 5 represent a single line of evidence, and we have revised the manuscript text to reflect this. The reviewers also propose an alternative hypothesis: that the correlation with IO_3^- could arise if we were misfitting the shoulder of a neighbouring I-OOM peak. While plausible, our analysis gives us confidence this is not the case. The sensitivity analysis mentioned above shows that the fitted peak time series is independent of the interfering peak. Most importantly, the strong correlation (R^2=0.74) between the cluster and HIO_3 is remarkable for an ambient dataset and is a powerful indicator of a genuine chemical link between these species, which would be expected for a nucleating cluster. This peak also is not detectable outside of NPF events, while I-OOMs do not. Finally, we cannot find a plausible I-OOM composition for this peak. Regarding the direct detection of DMA, we confirm that gas-phase DMA was not observed above our instrumental detection limit, which we estimate to be a few pptv [23-26], although we did not do a direct calibration for this campaign. This is consistent with other Arctic studies which have also reported amine concentrations below detection limits [27], and is also consistent with our low observed NH_3 concentrations, as we would expect these two species to be somewhat correlated. On the other hand, it is well-established that highly basic compounds like DMA, even at extremely low, sub-ppt concentrations, can be scavenged very efficiently into acidic clusters. Therefore, its presence in a cluster is plausible even when the gas-phase monomer is undetectable, especially as the limit of detection for these clusters is lower than that for the monomer.

Regarding the question why DMA was not observed in clusters with H_2SO_4 alone, a possible reason may be related to the specific thermodynamics favouring the formation of the HIO_x-DMA cluster under the observed conditions, or simply that the concentrations of potential H_2SO_4-DMA clusters were below our detection limits, which are determined by both the "conventional" instrumental detection limit, and the size of the adjacent (interfering) peak. This is different to the identification of iodine-containing peaks, which have a greater distance from any potential organic interference due to the negative mass defect of iodine. However, we acknowledge that we do not have definitive evidence to confirm the above mechanisms. We will add a sentence to the manuscript to acknowledge this.

To provide further confidence in the fit, we also state in the Methods section that our mass calibration uncertainty is <15 ppm, and we performed a sensitivity analysis which confirmed that the time series of the fitted peak is robust and not an artefact of the neighbouring peak. The proximity to the closest peak is 1.1 peak half-widths, which is challenging but resolvable with our instrument.

The relevant sections of the manuscript now read: (line 42-43)

"Our results validate the recent laboratory-based multi-component nucleation mechanism involving iodine oxoacids and sulfuric acid and expands this by showing the **potential** role of amines in **this** nucleation **mechanism**"

Line 227-234

"Considering the average ambient temperature of 1.4 °C during nucleation periods, the observed $J_{1.7}$ values are higher than anticipated from the temperature dependence measured in the CLOUD chamber [17] (at -10 and +10 °C). **This enhancement strongly suggests the participation of other stabilising species. One plausible contributor is DMA, as evidenced by the detection of DMA clustered with HIO_x (Fig. 2 and Figs. S5-6) on multiple NPF days. The DMA-HIO_2-HIO_3 cluster signal correlates with HIO_3 concentrations, and is highest when HIO_3 is at its maximum** (Figs. S9-10)**. The role of DMA in enhancing nucleation rate is corroborated by quantum chemical modelling studies showing that DMA can accept a proton from HIO_3 as a base, forming a stable cluster (DMA-HIO_2-HIO_3) [37]."**

(Line 241-245)

"Our results demonstrate that H_2SO_4, HIO_3, and HIO_2, are the core components of a synergistic nucleation mechanism in our study area. **This process appears to be potentially enhanced by the presence of bases such as DMA, and by OOMs**. This provides … ."

To provide further quantitative constraints on the abundance of amines, we performed an inverse kinetic growth calculation. While gas-phase amines were below the detection limit of the nitrate-CIMS, mass growth of particle-phase DMA and TMA were observed in the AMS data. We include peak fits below (Figure R1)

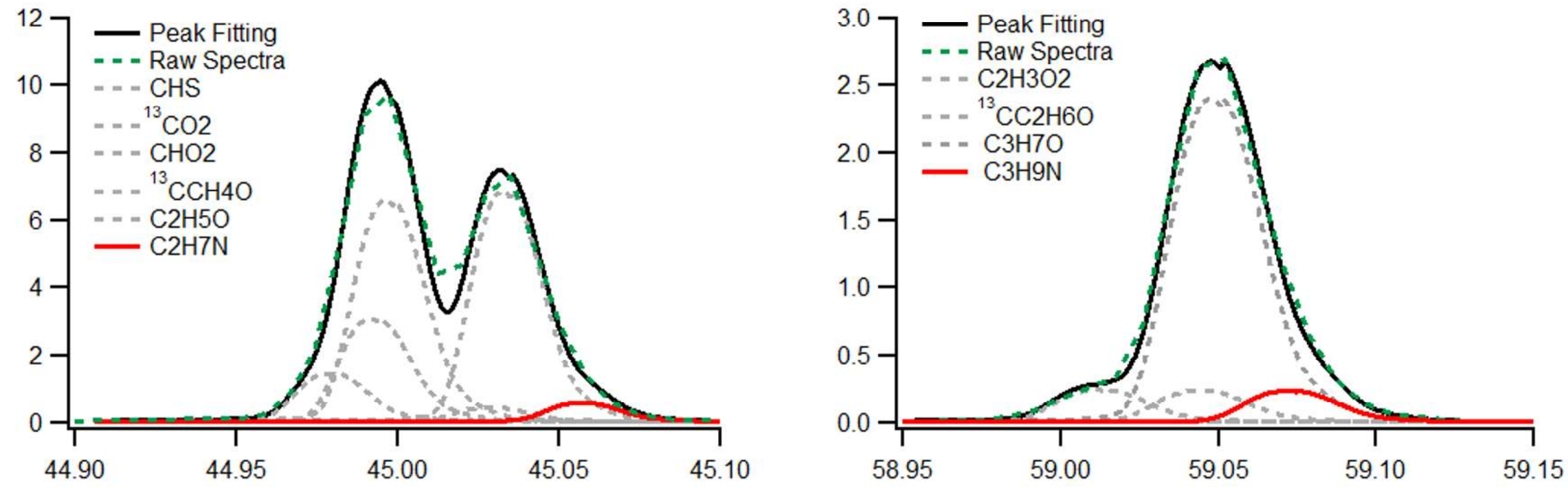

Figure R1: Peka fits for dimethylamine and trimethylamine in the AMS, units of Hz/ns. Data shown are from June 8th 16:30-June 9th 8:30

By employing the condensation growth framework described by Tröstl et al. (*Nature*, 2016), we can work backwards from the observed rate of change in particle-phase mass to estimate the gas-phase concentration required to sustain this growth.

The standard radial growth rate equation is given by:

dDp/dt = [(m_mol * c_mean * alpha * beta) / (2 * rho_p)] * (C_gas - C_eq)

Rearranging to solve for the necessary gas-phase concentration (**C_gas**):

C_gas = [(2 * rho_p) / (m_mol * c_mean * alpha * beta)] * [(Dp / 3) * (dM/dt) / M] + C_surf

C_gas = [(2 * rho_p) / (m_mol * c_mean * alpha * beta)] * (dDp/dt) + C_eq

As the AMS measures bulk mass rather than diameter growth directly, we substitute the radial growth term (Dp/dt) using the geometric relationship **Dp/dt = (Dp / 3) * (dM/dt) / M** This yields the final expression used for our estimation:

C_gas = [(2 * rho_p) / (m_mol * c_mean * alpha * beta)] * [(Dp / 3) * (dM/dt) / M] + C_eq

Where the Equilibrium Surface Concentration (C_eq) is: **C_eq = C_star * a_ip * K_ip**

And the Kelvin Term (K_ip) is: **K_ip = 10^((4 * sigma * Mw) / (2.303 * R * T * rho_p * Dp))**

Variable definitions: dM/dt is the rate of change of particle mass; M is current particle mass; Dp is particle diameter; beta is the Fuchs-Sutugin transition correction factor; c_mean is the mean thermal velocity of gas; C_star is saturation vapour concentration; a_ip is the particle-phase activity; and sigma is the surface tension.

We applied this inverse model to the AMS amine time series during the major growth event. The results are presented in **Figure R2**, which displays the particle phase mass and mass growth rate for the amines, the evolution of particle diameter from the PNSD, and the derived gas-phase mixing ratios.

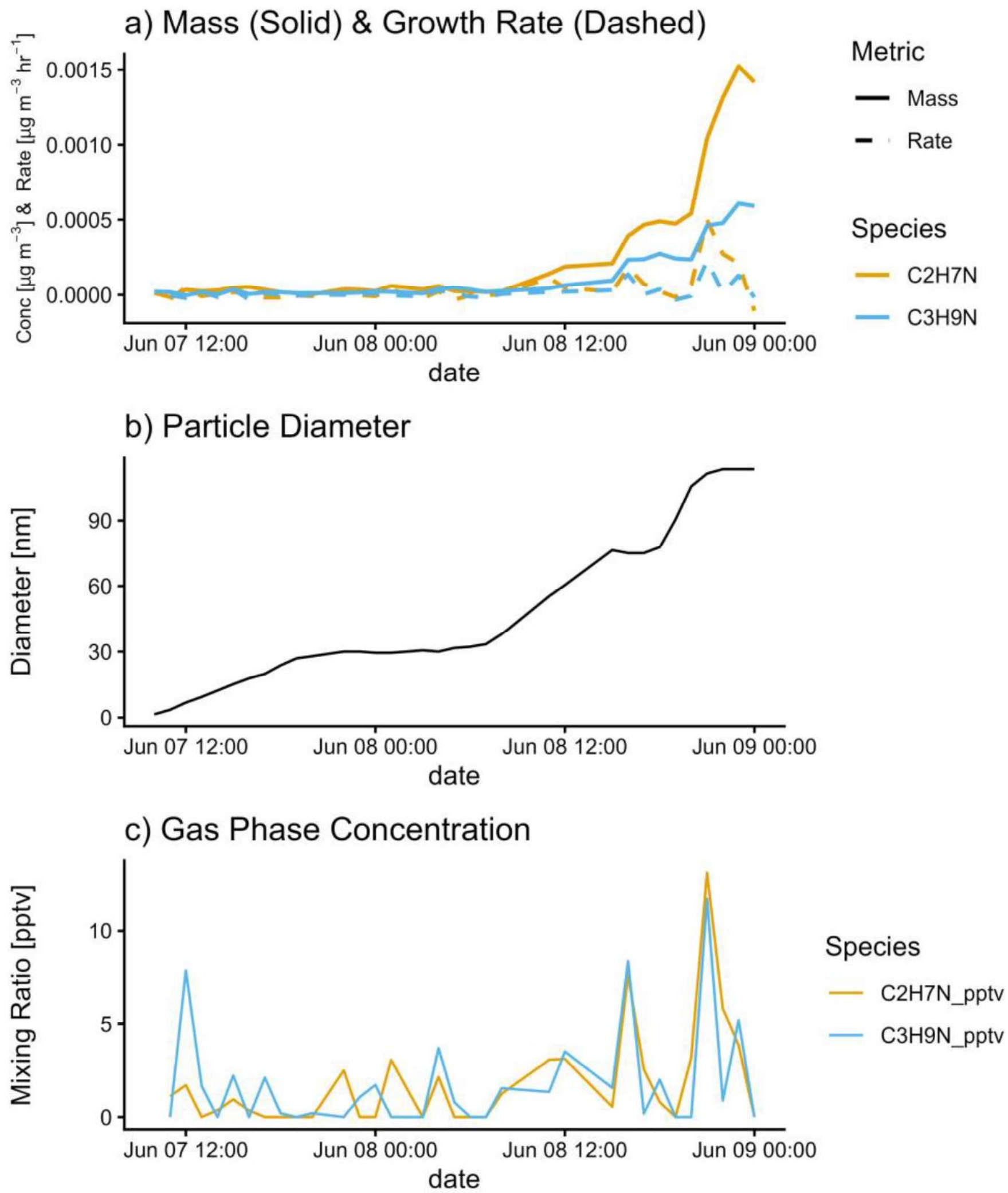

Figure R2: Amines in the AMS. (a) the particle phase concentrations of C_2H_7N and C_3H_9N, and the rate of change in particle phase C_2H_7N and C_3H_9N; (b) the measured particle diameter from the PNSD; (c) the calculated amine mixing ratios.

Assuming irreversible condensation (where C_gas >> C_surface), the model indicates that an average gas-phase concentration of approximately **1.8 pptv** (for both DMA and TMA) is sufficient to drive the observed accumulation of amine mass in the particle phase, and is also sufficient to enhance our observed J values.

To test the robustness of this estimate against the thermodynamic uncertainties of amines (specifically the acid-base chemistry that leads to the formation of low-volatility aminium salts) we performed two additional sensitivity runs allowing for reversible condensation (re-evaporation):

1. Assuming amines behave similarly to Low Volatility Organic Compounds (LVOCs) with a saturation concentration (C*) 10^{-3} ug m^{-3} and activity a_ip = 1).
2. Assuming a high saturation concentration characteristic of pure amines (C*) 10^{6} ug m^{-3} but a very low particle-phase activity a_ip = 10^{-7} to represent strong binding with acids and organics.

Both sensitivity scenarios converged on a required gas-phase concentration of approximately **2 pptv**.

This analysis demonstrates that a mixing ratio of ~2 pptv is sufficient to explain the observed particulate amine mass. This concentration is likely below the detection limit of the nitrate-CIMS, explaining why the monomer was not directly observed in the gas phase. However, even at these trace levels, amines are capable of participating in nucleation and growth, and these numbers are consistent with the low-DMA experiments of Almeida et al (2013) [5]. Thus, the lack of a gas-phase CIMS signal does not contradict the presence of amines; rather, the AMS data and kinetic modelling strongly support their presence and participation in the observed NPF events.

Finally, regarding Figure S4 (now S9), we have reproduced this figure. The peak fits were done appropriately, but there was an error in the time series. Please see the below.

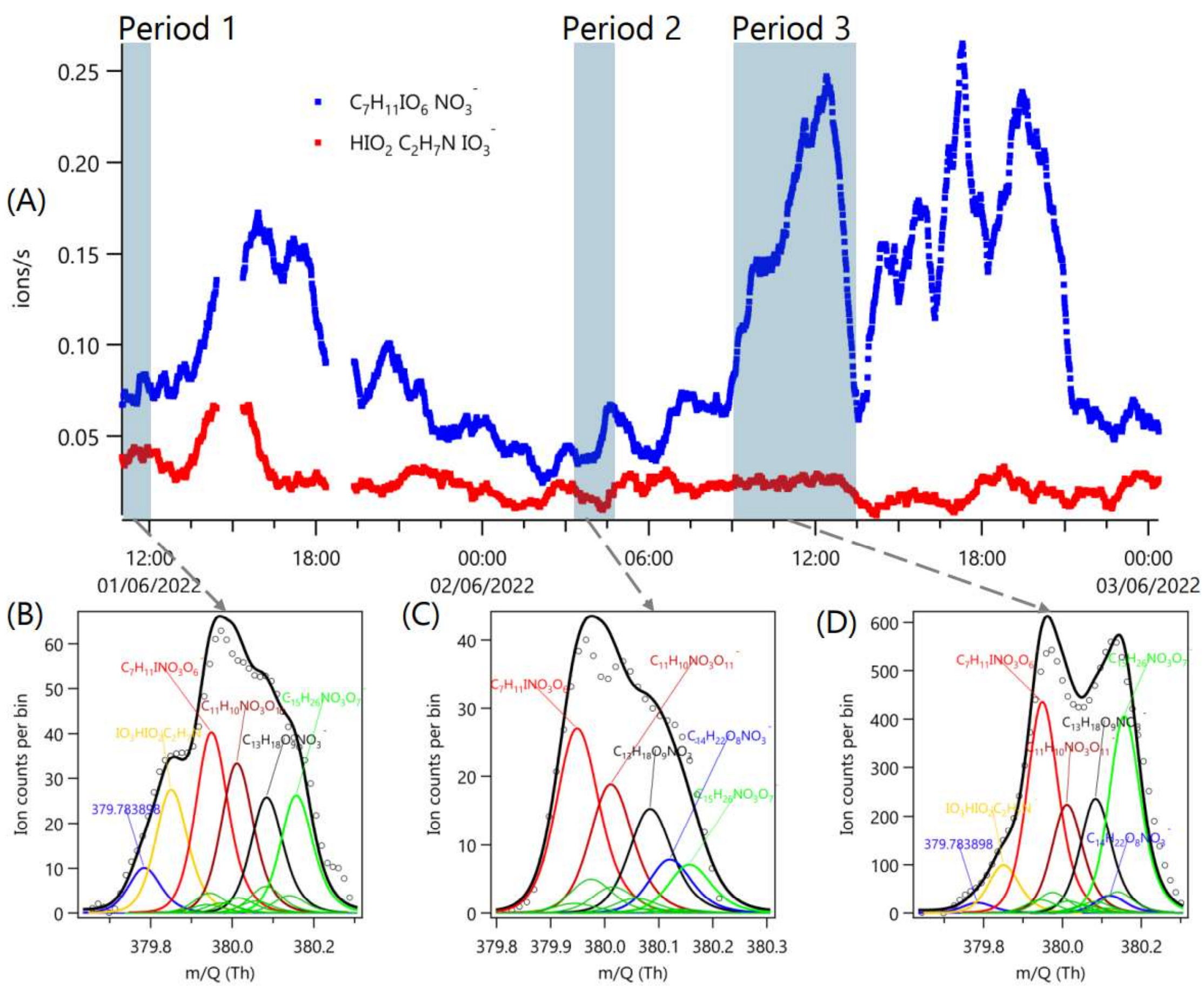

Further points:

Comment 3: Figure 2 (MD plots) and the related conclusions:

The labeling is inconsistent: some peaks are labeled with a negative charge and others not. Please make this consistent. We would suggest to add the negative charge to all peaks in the MD plot. Also, did you measure sulfuric acid only at m/z 97 or also at m/z 160, since it is not shown. The same question arises for MSA. Why is HIO_2 located at m/z of almost 300 and to the right of HIO_3? Either the peak id or position is wrong (We understand that your Table S2 provides the solution to this, but

it is still misleading in the Figure, especially to a non-expert reader). As your goal is to show cluster formation, please also show the peaks where they were measured.

Response: Thank you for pointing this out. As this figure aims to illustrate nucleating clusters, we present our measured inorganic monomers (H_2SO_4, MSA, HIO_3, HIO_2) as aggregated total signals, rather than in the multiple forms in which they are detected. Specifically, 'H_2SO_4' is the sum of HSO_4^- (m/z 96.9601), $H_2SO_4NO_3^-$ (m/z 159.9563), and $H_2SO_4{\cdot}HNO_3{\cdot}NO_3^-$ (m/z 222.9514). 'MSA' is the sum of $CH_3SO_3^-$ (m/z 94.9808), $CH_3SO_3HNO_3^-$ (m/z 157.9770), and $CH_3SO_3{\cdot}HNO_3{\cdot}NO_3^-$ (m/z 220.9727). 'HIO_3' is the sum of IO_3^- (m/z 174.8892), $HIO_3NO_3^-$ (m/z 237.8860), and $HIO_3{\cdot}HNO_3{\cdot}NO_3^-$ (m/z 300.8816). Iodous acid (HIO_2) was observed only as $HIO_2{\cdot}HNO_3{\cdot}NO_3^-$ (m/z 284.8856), the only known detectable HIO_2 ion in nitrate-CIMS (see He et al. 2023). All ion assignments are listed in Table S2. For clarity, we added the following in the caption of Fig. 2 (Line 182-183):

"Here, we report H_2SO_4, MSA, HIO_3, and HIO_3 as the sum of their deprotonated and clustered-clustered forms. The individual ions are shown in Table S2."

Comment 4: Are there noise peaks in the MD plot, i.e. is the lower cutoff for the data sufficiently high to exclude noise? Phrasing this in another way, are the many organic peaks especially in the Pre-NPF plot real? It is actually rather easy to blow up noise in an MD plot, so please be careful here.

Response: We confirm that we carefully excluded noise-peaks (hence the fewer number of data points in panel C). Our lower threshold was 0.01 cps, which is roughly equal to ~10^3 cm^{-3}

Comment 5: In line 140 you state that H_2SO_4-HSO_4^- was only identified during nucleation events, but in panel (c) of Fig. 2 it is clearly visible.

Response: Thank you and apologies for the oversight! This section now reads as follows (Line 164-165):

"Notably, the dimers HIO_3-HSO_4^- and HIO_2-HIO_3-NO_3^-, and trimers HIO_2-H_2SO_4-HSO_4^- and C_2H_7N-HIO_2-IO_3^- were only identified during nucleation events"

Comment 6: There seems to be a surprisingly sharp cutoff in the nitrate-CIMS data at m/z larger than 500. This seems to be true for all compounds regardless of their chemical classification. Could this be due to an instrumental issue, i.e., have you evaluated if there is a strong loss of transmission for the higher masses?

Response: This was intentional—with our limited mass resolution (~4000), it becomes difficult to assign peaks confidently past this point. Peaks with a mass >400 m/z made a very small contribution to total signal, and therefore we set a threshold to not assign peaks much past m/z 500. We mention this explicitly in the following line of the revised manuscript (line 549).

"Peak fitting was performed following the procedure outlined in Tofware, after which molecular formulas were assigned.

Line 561-567

"Our assigned OOM formulas avoided hypervalent molecules, molecules with H:C ratios below 0.5, or O:C ratios exceeding 2, which is the upper limit observed in laboratory studies. Molecules that would not be detected by NO_3^- CIMS (less than four oxygen atoms), as well as deprotonated OOMs (as opposed to clusters with NO_3^-), were deprioritized. **Due to the increasing difficulty in confidently assigning molecular formulas at higher masses with our instrumental resolution, we did not assign peaks beyond an m/z of ~550**."

Comment 7: Can you please discuss more clearly what distinguishes a burst form an NPF event in terms of gas phase concentrations, and which gas phase levels are needed to make it from a burst to a NPF event? You show some data for NPF and bursts in Fig 1 and simply state that there are lower acid and OOMs concentrations in the latter case. But what is the limiting factor for growth vs. no growth? You nicely modelled several NPF events (ED Fig 7), but could you also do this for burst events with lower acid/OOM levels, and does the modelled growth then also stop?

Response: To clarify the distinction between NPF and Burst events, we provided an overview of the conditions for each event type in Fig. S2 (now Fig. S4). This figure shows that while both event types occur under high solar radiation and a relatively low condensation sink, the primary distinguishing factor is the concentration of condensable vapours. For all key precursor species (H_2SO_4, HIO_3, HIO_2, OOMs, and MSA) the median concentrations during NPF events are higher than during Burst events.

We noted that the difference in the concentration of OOMs (the primary driver for particle growth) is about 40% lower during burst events. A reduction of this magnitude would likely lead to slower growth but this should not necessarily halt it completely. This suggests that simple vapour limitation may not be the sole explanation. Therefore, we propose two plausible physical explanations for the lack of observed growth during burst events:

1. **Vapour Limitation:** The local concentration of condensable vapours, while sufficient to initiate nucleation, was too low to drive sustained growth past the initial small cluster sizes.
2. **Meteorological Influence:** The nucleation event may have been localised or occurred at the edge of an airmass. It could be that we sampled the initial burst of particles, but the airmass subsequently moved away from our measurement location before any significant growth could be observed. A recent paper on airmass trajectories around hotspots demonstrates this phenomenon nicely [28].

In the main text we now state the following (Line 153-161):

"The median concentrations of all condensable vapours are lower during burst events relative to NPF events, with OOMs about 40% lower. As the primary growth driver, this would likely only slow, not halt, particle growth. This suggests that while vapour availability is a key factor, other factors may also be important for a lack of growth during burst events, for example, meteorological influence, where the nucleation event may have been localised and the airmass moved away from our measurement location before significant growth could be observed. The condensation sink, the rate at which low-volatility vapours like sulfuric acid are lost to pre-existing particles, was 40% lower on NPF event days as compared to non-event days, and highest on burst events ($7\cdot10^{-4}$ s^{-1}, $5\cdot10^{-4}$ s^{-1}, and 10^{-3} s^{-1} respectively) (Fig. S4 and Table S1).

Comment 8: Related to the growth model is the volatility estimation. Here, you use the volatility basis set, for which you use certain parameters like nO, bC, or bO. These parameters where derived for monoterpene based HOMs. In how far are they valid for aldehyde derived OOMs? For some events your OOM spectrum is aldehyde-OOM dominated (ED Fig. 8), so this might play an important role for your growth model estimations.

Response: The linear equation we use is based on group-contribution estimations, which implicitly assume that the functional groups are known but the molecular structure is not (or it is unimportant) [29]. The first version of this equation from Donahue et al. (2011) presumed that a mixture of carbonyl and alcohol functional groups are present, with the former reducing volatility by 1 order of magnitude, and the latter reducing volatility by 2.4 orders of magnitude. The mean of these numbers is 1.7, therefore the effect of one oxygen molecule was presumed to be 1.7 orders of magnitude ($C^* \propto nObO$ where nO is number of oxygens, and bO is 1.7). As HOMs (a.k.a. OOMs) contain many -OOH and -OO- functionalities, the equation we use in our paper has been modified and contains a non-linear term (in bold)[30]:

$$\log_{10} C_i^*(300K) = (n_0 - n_\mathrm{C})b_\mathrm{C} - (n_\mathrm{O} - 3n_\mathrm{N})b_\mathrm{O} - 2\frac{(n_\mathrm{O}-3n_\mathrm{N})n_\mathrm{C}}{(n_\mathrm{C}+n_\mathrm{O}-3n_\mathrm{N})}b_\mathrm{CO} - n_\mathrm{N}b_\mathrm{N} - (n_\mathrm{I}b_\mathrm{I})$$

where $n_0 = 25$, $b_C = 0.475$, $b_O = 0.2$, $b_{CO} = 0.9$, $b_N = 2.5$, and $b_I = 2.0$. This final term, which does stem from monoterpene research, specifically accounts for how the volatility reduction per oxygen changes as the molecule becomes more oxygenated (e.g., shifting from carbonyl/alcohol groups to a higher proportion of hydroperoxide groups). This behaviour is a general feature of autoxidation, not a phenomenon exclusive to monoterpenes.

We have good reason to believe aldehydes undergo autoxidation, as their autoxidation has been observed in the laboratory [31, 32], and the presence of the C=O group makes the adjacent hydrogen more labile, facilitating the abstraction [33, 34]. It is therefore reasonable to assume their oxidation products will have a distribution of functional groups (hydroperoxides, carbonyls, etc.) that can be approximated by this same general parameterisation.

While we acknowledge that specific parameters for aldehyde-derived OOMs are not yet available, this group-contribution method is the most robust and mechanistically appropriate approach currently available. Furthermore, as the OOM spectrum was not exclusively dominated by aldehyde-OOMs across all events, any potential uncertainty introduced by this assumption is unlikely to alter the overall conclusions of our growth model.

Minor comments:

Comment 9: As a general remark: You first describe your measurements in detail and then go on to describe where the individual gases can originate from. Can you add at least a very brief (half) sentence when you first describe where the gases are coming from, e.g. for your VOC measurements? You could also move the whole section starting at line 311 upwards, but this is up to you.

Response: We understand it could be useful to describe the sources when first mentioned a species. We have now added some contexts when we first mentioned the NPF precursors (see below). We intend to keep the description of the results more concise, and leave discussions for later when we bring all the results together and discuss in the context of literature (line 132-138).

“A suite of instruments measured particle number size distributions (PNSD), gas-phase NPF precursors, and aerosol composition (Fig. 1, Fig. S2-3, see SI-Methods 2-9). **The key precursors for NPF—H_2SO_4, MSA, HIO_x, and a wide array of VOCs and OOMs—are known to originate from a complex mix of marine biogenic and abiotic processes, particularly prevalent in the biologically active marginal sea ice zone.** Background concentrations of NOx (<50 ppt), SO_2 (median 23 ppt) and BC (1.9 ng m^{-3}) were very low…”

Comment 10: L 76: This is worded a bit awkward, as you first state that CLOUD discovered synergistic nucleation of iodine oxoacids and H_2SO_4, which can enhance H_2SO_4-NH_3 nucleation and then you say that this challenges the prevailing view for which you list the exact same components. Please make it clearer what is the prevailing view and what challenges it.

Response: Thank you, we amend this as follows (line 76-77)

“This challenges the prevailing view on the dominant role of H_2SO_4-NH_3 **or** iodine oxoacid nucleation **as independent pathways** at high latitudes [6,8,13,14].”

Comment 11: Paragraph following L 93: Here you point out where other studies are lacking, so here would be the natural place to describe briefly in the main text in how far your work addresses these gaps. Please add also a sentence somewhere in the paragraph stating that your data was collected with the RRS Discovery, it was collected from May 19th to June 26th 2022, stating also the DY151 campaign, see Methods),…”. This would also make a good transition to the text following in l 102.

Response: Great suggestion, thanks. We have included the following (Line 108-115):

“Here, using comprehensive measurements collected aboard the RRS Discovery during the DY151 campaign (19th May – 26th June 2022), we provide the first real-world evidence that Arctic nucleation is driven by a synergistic interaction between sulfuric acid and iodine oxoacids. Furthermore, we show at a molecular level that subsequent growth to CCN sizes is dominated by the condensation of OOMs, including a previously unobserved class of iodine-containing OOMs. By directly linking emissions from the marginal sea ice zone to a substantial increase in CCN, our findings provide a complete observational closure from precursors to cloud- and climate-relevant particles.”

Comment 12: Fig 1: Please add a legend to panel A for the lines and markers. (This is a somewhat recurring theme, clarity would be enhanced also for some of the other figures by adding such legends)

Response: We amended this as suggest.

Comment 13: L 145-146: What is meant here? We hope you did perform peak fits for all your data. What does “to account for the variability” mean?

Response: We did indeed perform a lot of peak fitting. As in Response to Comment 1, we now say the following (line 170-176)

“A day-by-day analysis (Figs. S5-6) reveals that while the detectability of specific clusters varies with precursor concentrations and analytical interferences, the synergistic iodine-sulfur mechanism is a persistent feature. On nearly every NPF day, we observe a clear signal for at

least one key cluster containing both HIOx and sulfuric acid (e.g., the $HIO_3 \cdot HSO_4^-$ dimer or the $HIO_2 \cdot H_2SO_4 \cdot HSO_4^-$ trimer), confirming the consistent co-involvement of both precursor systems. For example, during the event on 7th June, we identified the key multi-component clusters HIO_2-HIO_3-NO_3^- and HIO_2-H_2SO_4-HSO_4^-."

Comment 14: L 156: Rather state values of the ratio (or a range of values) as well as stating the concentrations. Then the reader can judge in how far the ratios are similar, etc.

Response: We agree. The new text now states the typical concentration ranges for our ambient measurements, and then guides the reader to Figure 3A to observe the key finding: that the slope of the relationship in our data is remarkably consistent with the CLOUD chamber data, despite the different concentration regimes. We refer the reader to Figure 3A and He et al. where the power-law equations are stated in the captions rather than state the ratios in the text, as they are described by power-law relationships and therefore the ratio is non-constant.Line 184-190

"The concentrations of HIO_3 and HIO_2 were well correlated during our campaign, **with median HIO_3 of $2 \cdot 10^5$ cm^{-3} and HIO_2 of $1.6 \cdot 10^4$ cm^{-3}. The power-law relationship between them (Fig 3a) is consistent with that stated in He et al. (2023) [17], showing a non-constant ratio between HIO_2 and HIO_3. The concentrations of HIO_3 and HIO_2 in our observations are often over an order of magnitude lower (median HIO_3 $8 \cdot 10^6$ cm^{-3}) than those used in the CLOUD chamber.** This correlation is also consistent with observations at Mace Head and Ny-Ålesund (Fig. 3A) [17]. Using this relationship, we applied the more readily observed HIO_3 to estimate HIO_2 concentrations when they were not detectable on 23rd and 24th May (see Fig. S2)."

Comment 15: Fig 3, panel A: can you provide a 1:10 line to guide the eye? In panel B, please add CLOUD to the legend, as you have done in A.

Response: This is now amended.

Comment 16: L 174: If you report daily hourly peak formation rates, could this mean that J rates on a shorter timescale could be much higher?

Response: Indeed. For example, the half-hourly mean formation rates during the nucleation period are 1.5 cm^{-3} s^{-1} compared to a 0.79 cm^{-3} s^{-1} hourly averaged formation rate on 1st June, and 0.76 cm^{-3} s^{-1} compared to 0.27 cm^{-3} s^{-1} on 2nd June.

Comment 17: L 178-180: J rates for H_2SO_4-DMA should better be compared to the updated analysis by Kürten et al., ACP, 2018.

Response: The conditions under which the correction of of Kürten et al. (2018) are applicable do not apply to our study for two primary reasons.

First, from a practical standpoint, Kürten et al. (2018) could only apply their reconstruction method to experiments where sulfuric acid concentrations were sufficiently high (>~5x10^6 cm^{-3}) to determine a reliable particle growth rate. The high DMA, high H_2SO_4 experiments from the CLOUD chamber where the reconstruction was applied are not representative of our environment where, for example, DMA monomers were below the detection limit of the nitrate CIMS, which we estimate to be a few pptv [23-26]. The low-DMA data of interest were therefore not corrected.

Second, this correction accounts for self-coagulation. The likelihood of coagulation is proportional to the square of the particle concentration. Kürten et al. (2018) corrected data where the $J_{1.7}$ values were on the order of 10^2 cm^{-3} s^{-1}. For the low-DMA datapoints of Almeida et al. (2013), the reported $J_{1.7}$ values are in the range of 10^{-2} to 10^{-1} cm^{-3} s^{-1}, therefore the likelihood of self-coagulation is much smaller and the correction would be much smaller.

Comment 18: L 219: Please clarify that/if this PMF is based on another instrument than the AMS PMF you just described above.

Response: To clarify, we performed two separate PMF analyses, one on AMS data and one on CIMS data. We amend this section as follows, where the logic now introduces all the gas-phase findings, then the particle phase findings. Below we include the full, rearranged section (line 249-266):

"We identified 591 gas-phase OOMs using the NO_3- CIMS. We detected an additional 91 iodine-containing OOMs (referred to I-OOMs), with detailed identification methods given in SI Method 3. To our knowledge, this is the first detection of I-OOMs in a laboratory or field study. Peak fits for the most abundant I-OOMs are shown in Fig. S11. These gas-phase I-OOMs correlate well with HIO_3 (Fig. S12). To understand the sources of these gas-phase precursors, we applied PMF analysis to the NO3- CIMS data, identifying three distinct factors: aldehyde-rich, monoterpene-rich and I-OOMs (Figs. S13-14). Our aldehyde-rich factor has a high contribution from C_9 to C_{15} OOMs, and its carbon number distribution matches our 2D-GC-MS results (Fig. S15), while monoterpene-rich OOMs are characterised by C_9-C_{10} compounds, typical oxidation products of monoterpenes [39]. I-OOM factor has the highest contribution from I-OOMs. All three factors peak in the afternoon, though at different hours. The concentration of I-OOMs represents, on average, ~10% of total OOMs.

A separate PMF analysis of our Aerosol Mass Spectrometer (AMS) data further supports the role of I-OOMs in growth of new particles. We identified an iodine organic aerosol factor, which contains the CH_2I^+ fragment (Methods 12) and is likely due to I-OOM condensation. This factor correlates poorly with sea salt (Fig. S16), but its concentration increased as particles grew larger (Figs. 4F and S16), further confirming the contribution of I-OOMs to particle growth"

Comment 19: L 234: "good agreement" between the growth rate model and the measurements is arguable. For all but three data points you model only half or less of the observed growth. This should be stated, as it seems to be a systematic effect that should be discussed. One reason is certainly, as already stated by you, that the nitrate-CIMS measurements can only be regarded as lower limit estimates, especially for lower oxygen compounds. Also, please discuss somewhere what happened on May 30. Is there an explanation why in this case the measured growth rates are lower than the model predictions (can be done in SI text 3). Note also that in SI Text 3 you state "Our modelled growth rates do not match the measured GRs from the PNSD for every event", which carries a different tone than your "good agreement" stated in the main text.

Response: We agree. Please see our amended text below (line 268-284).

"We observed six events with measurable particle growth rates. Particles formed during these NPF events consistently grew to 20 nm within a few hours. GRs for particles of diameters 3 to 15 nm

varied between 1.5 to 3.6 nm h^{-1} (Table S1), which are relatively high among those observed in long-term data in the Arctic (Fig. S7), but akin to measurements made in the region west of Greenland [40]. To investigate drivers of particle growth during NPF events, we utilised a dynamic aerosol growth model incorporating concentrations of gas-phase acids (H_2SO_4, MSA, HIO_3) and OOMs (see Methods 11). Saturation vapor concentrations were calculated following the methodology outlined by Mohr et al. [41]. Our model typically accounts for half or more of the observed growth rates (Fig. S17). This underprediction is expected, as the NO_3^- CIMS provides a lower-limit estimate on the total OOM concentration. Specifically, it has a well-known lower sensitivity to less oxygenated, more volatile species like semi-volatile organic compounds (SVOCs), which were therefore not constrained by our measurements. The condensation of these unmeasured SVOCs likely accounts for a significant fraction of the unexplained growth, particularly for the later stages of growth towards larger particle sizes. Other factors, such as multiphase reactions and coagulation growth, as well as potential change in meteorological conditions may also explain the discrepancies between observed and modelled GRs (see Supplementary Text 5)."

Please also see response to comment 7 re: 30 May event (Line 291-292):

"On one occasion (30th May), the model overpredicted growth, likely due to meteorological factors not captured by the model (see Supplementary Text 5)."

A recent paper on airmass trajectories around hotspots demonstrates this phenomenon nicely [28].

Comment 20: L 265: Why do you think that the high aldehyde to monoterpene ratios indicate abiotic OVOC emissions? Do the aldehydes not stem from biotic processes originally?

Response: We agree that the ultimate source of the carbon in these aldehydes is biological. Our use of "abiotic" and "biotic" refers to the mechanism of release from the ocean to the atmosphere. Monoterpenes are associated with direct emission from active phytoplankton blooms which then are emitted to the atmosphere. Contrarily, there is strong evidence that aldehydes arise from abiotic processes in the sea surface microlayer [11-13], and inference that this is from the ozonolysis of fatty acids. Therefore, a high aldehyde-to-monoterpene ratio suggests that the dominant source of VOCs in this region is the photochemical processing of background organic matter (abiotic emission), rather than direct emissions from active biological blooms (biotic emission).

Comment 21: Fig. 4E: In the caption you speak of concentration, but your y-axis is a mixing ratio.

Please remove the H^+ from the peak labels in the legend of Fig 4E, as the H^+ is not present in the atmosphere.

L 324: Extended Data Fig. 1G is meant here, not S1G

L 338: Fig 4 A does not show monoterpene measurements

Response: All amended.

Comment 22: L 345: Here you state that NH_3 is abundant in the high Arctic but in the SI Text 6 you state referring to the same reference "vast areas of the Arctic has low NH_3 emissions". Please clarify

whether and where NH_3 plays a role in the Arctic. Please do not simply state a compound is abundant, but always give at least a range or limit for mixing ratio or concentration.

Response: We agree - this was our mistake. Croft et al. in fact do ***not*** predict high concentrations of ammonia in the high Arctic. We amend as follows (line 400-408)

"HIO_x and H_2SO_4 are likely ubiquitous across the marine Arctic [8,13,14], and we argue that the synergistic nucleation process we observed is therefore likely ubiquitous across high latitude marine environments. The importance of our observed NPF processes is likely to manifest throughout polar regions where the ice is melting in larger areas, including in the Antarctic [56]. Nucleation rates can be enhanced by NH_3, which is abundant (>100 pptv) in coastal regions of the Arctic with bird nests, but predicted to be almost absent in the ice-covered high-latitude areas (<1 pptv) and central ocean regions [33,53]. Amines (as detected in the DMA-containing cluster in Figs. S9-10) and OOMs can further enhance nucleation rates."

Comment 23: L 444: Why are you changing the convention and naming a SMPS MPSS? I get that you want to group them together with the NAIS, but it still sounds confusing to read long-MPSS and nano-MPSS.

Response: We understand there are different terms use. We choose MPSS as it falls into the broader category of particle size spectrometer (PSS), also including e.g. aerodynamic particle size spectrometers. We used the suggestions made by ACTRIS which uses mobility particle size spectrometer (MPSS), as is Nano-MPSS. See: https://vocabulary.actris.nilu.no/actris_vocab/particlesizespectrometer.

Comment 24: L 529: Kürten, not Kurten

Response: Amended.

Comment 25: L. 708-714: This sentence is only needed once, not three times.

Response: Amended.

Comment 26: L 753: Please explain why you use condensation sink. You only mention it in the main text, but in a way that a non-expert reader cannot really understand it. Here you also just give the calculation, but not the meaning. I suggest you add a sentence about CS in the main text where you mention it briefly and discuss here why you did calculate it and what information it contributes to your analysis.

Response: We agree. We now include the following sentence when the CS is first mentioned (Line 158-161)-

"The condensation sink, the rate at which low-volatility vapours like sulfuric acid are lost to pre-existing particles, was 40% lower on NPF event days as compared to non-event days, and highest on burst events ($7 \cdot 10^{-4}$ s^{-1}, $5 \cdot 10^{-4}$ s^{-1}, and 10^{-3} s^{-1} respectively) (Fig. S4 and Table S1)."

In our methods, we now amend the sentence introducing the condensation sink as follows Line 832-835)

"**The** condensation sink (CS) **describes the rate** of vapor condensation **to** the existing particle population. It is estimated based on the following equation:

$$\mathrm{CS} = 4\pi D \int_0^{d_\mathrm{p}^{\max}} \beta_\mathrm{m}(d'_\mathrm{p}) d'_\mathrm{p} N_{d'_\mathrm{p}} \, dd'_\mathrm{p} = 4\pi D \sum_{d'_\mathrm{p}} \beta_{m,d'_\mathrm{p}} d'_\mathrm{p} N_{d'_\mathrm{p}} \qquad (9)$$

Where D represents the diffusion coefficient of the condensing vapor, which is here assumed to be sulfuric acid."

Comment 27: L 1016: You state "Factor 2 is dominated by I-OOMs, referred to as "I-OOM" factor." However, looking at ED Fig. 9 only a minor fraction of molecules of this factor does actually contain an iodine atom. Furthermore, it is misleading to term this factor "I-OOM", because you use I-OOMs also as a chemical class (implying iodine content >0). Please avoid using the same name for the chemical class and for the PMF factor. Please make sure that this does not lead to confusion.

Response: We agree. We believe our PMF factor represents a source, rather than a category of molecules. Due to the lability of the carbon-iodine bond, it could be that some I-OOMs form but then subsequently lose the iodine molecule, or the reaction pathway that forms them involves iodine but does not necessarily impart iodine into the resultant OOM. To avoid confusion, we renamed our I-OOM factor to Iodine-related OOMs. We clarify this as follows (line 1188-1192):

"… the largest iodine-containing peaks are $C_8H_{11}IO_5\ NO_3^-$, $C_{10}H_{15}IO_9\ NO_3^-$, and $C_8H_{12}INO_8\ NO_3^-$ (Table S4**). Some of the peaks in this factor do not contain iodine, and therefore it is likely that some I-OOMs form and then subsequently lose the iodine molecule, or the reaction pathway that forms them involves iodine but does not necessarily impart iodine into the resultant OOM."**

Comment 27: L 1022: You state: "Given that reactions between iodine-containing species and VOCs are significantly slower than those involving other halogen radicals such as Br and Cl [101], direct VOC reactions are unlikely pathways for I-OOM production." Smaller reaction rate constants can be overcompensated by higher concentration (e.g., O_3 vs OH reactions). Please clarify what exactly you mean with "direct VOC reactions"? The proposed termination reaction seems plausible. However, you report IO levels of 3 ppt and state that "this is sufficient to intercept a significant fraction of autoxidation reactions". Can you compare this 3 ppt value to values for OH, HO_2 and NO, which you mention, but do not provide numbers for.

Response: Thanks for the suggestions, please see our following additions to the section (line 1193-1210):

"We propose a hypothesis for I-OOM formation. The diurnal profiles of these compounds indicate daytime formation, implicating photochemical processes. Given that reactions between iodine-containing species and VOCs are significantly slower than those involving other halogen radicals such as Br and Cl [115], direct reactions between VOCs and iodine radicals are unlikely pathways for I-OOM production, especially as we do not anticipate substantially higher iodine radical concentrations relative to chlorine and bromine, as indicated by their relative oxidation product

concentrations during recent Arctic campaigns [116,117]. Molecular composition offers further insights: for instance, $C_{10}H_{15}IO_{3-9}$ matches well-characterized OOMs ($C_{10}H_{16}O_{3-9}$) formed via monoterpene oxidation in iodine-free environments [39], differing only by the substitution of a hydrogen atom with iodine. Typical OOM formation proceeds through autoxidation, wherein the radical chain is terminated either by radical–radical collisions or propagated via self-reactions. These terminating radicals are often OH, HO_2, or NO. In the underexplored marine atmosphere, we hypothesize that termination may involve iodine-containing radicals such as IO or organic iodine species (CxHyI). Arctic observations report IO levels of ~3 ppt [50], sufficient to intercept a significant fraction of autoxidation reactions and yield the observed I-OOMs, as these concentrations are comparable with OH and HO_2 concentrations in the Arctic [118]. Hence, I-OOM formation likely adheres to established autoxidation mechanisms, diverging in the identity of the terminating radical. Further laboratory validation is warranted to substantiate this mechanism."

Comment 28: L 1069: Please provide some additional information on the ammonia measurements (fits best in the method section that covers already many gases, but not NH_3).

Response: This has been added to Methods (line 752-755)

"The NH_3 measurement was carried out by an Automated Ammonia Analyzer (AiRRmonia, Mechatronics B.V., NL). The AiRRmonia employs a wet-chemistry approach for NH_3 analysis, utilizing a selective diffusion membrane in combination with conductivity measurements. The detection limit is around 0.05 µg m^{-3} (equivalent to ~65 ppt at STP, 0 °C) [88]." **Comment 29**: L 1123: You state the comparison with Antarctica is beyond the scope of this paper, but then you go on and make a comparison.

Response: Our intention was that it was outside of the scope of the main text, but this is somewhat confusing and adds nothing. We remove this statement.

Comment 30: L 1111: Section 7 focusses on pointing out shortcomings of other studies. Reading the title of this section we expected a text about the shortcomings in understanding of NPF and growth in the Arctic after your study. A text criticizing previous studies is not really useful (especially not in the current form). While It is justified to show your advances, but this section is overemphasizing the shortcomings of other studies, while ignoring the remaining gaps after your study, and acknowledging shortcomings of your own study. This paragraph should rather be used as a chance to constructively discuss the remaining challenges in the Arctic with respect to your topic.

Response: We appreciate this comment and have reflected on the point. We recognise that the original wording could be read as over-emphasising gaps in prior work without sufficiently articulating its foundational contributions. We have revised the section (see below) to (i) explicitly acknowledge the existing work on which our study builds and how it motivated our hypotheses, (ii) clearly state what our study adds and how it tests existing hypotheses, (iii) identify the limitations of our approach, and (iv) frame remaining research needs without implying deficiencies in earlier studies (Line 956-1056):

"1. Advancing the Understanding of Arctic NPF: Context and Future Directions

1.1 Nucleation

Figure S1A illustrates our current understanding of nucleation mechanisms in the Arctic, based on respective statements made in published papers and our new results. Note that this figure is slightly different to Figure 1 in Schmale and Baccarini [6].

Two recent studies provided molecular level information on nucleation processes in the Arctic. Beck et al. [13] observed NPF across two seasons and two Arctic sites. At Ny-Ålesund, they concluded that nucleation is driven by H_2SO_4 and NH_3; at Villum, they reported that springtime nucleation is driven by iodic acid, while in summer, nucleating clusters consist of H_2SO_4 and NH_3. Baccarini et al. [14] reported that all the new particle formation events observed over the central Arctic pack ice during their late-summer/early-autumn campaign were driven primarily by HIO_3, with little contribution from sulfuric acid. Their measurements therefore identify HIO_3 as the dominant nucleating vapour during their campaign

The Antarctic differs significantly from the Arctic in terms of atmospheric chemistry. Sources of molecular iodine and organics are considerably weaker, and NPF appears to occur exclusively via sulfuric acid nucleation with ammonia and amines [35,98,106,107]. This contrast is likely due to the distinct nature of the Arctic, including its extensive coastlines with high freshwater discharge, distinct oceanic biology, and larger annual ice melt.

These studies showed multiple components contributing to nucleation in polar regions, with a dependence on the season and location – they significantly advanced our understanding of NPF processes in a region where large-scale in-situ observations are highly challenging.

Here, we found in-situ evidence of synergistic, multi-component nucleation (i.e., multiple components nucleate simultaneously) in the Arctic atmosphere, specifically HIO_3, HIO_2 and H_2SO_4. This validates the newly proposed mechanism by He et al. [17] through the CLOUD chamber experiment.

We also detected a DMA-containing cluster. Although this is unambiguously detected only during a short period of time in this campaign, DMA is capable of enhancing nucleation rates at sub-pptv concentrations [108]. Finally, we observe high concentrations of OOMs, which have been shown to be capable of enhancing the nucleation rate of sulfuric acid clusters [32,108]. These results led us to hypothesise that DMA and OOMs may also contribute to nucleation in the study region.

1.2 Growth of new particles

Figure S1B illustrates our current understanding of new particle growth mechanisms in the Arctic, based on respective statements made in published papers and our new results. Note that this figure is slightly different to Figure 1 in Schmale and Baccarini [6].

Figure S1B suggests that there is emerging evidence that organics contribute to particle growth in the Arctic, including two sets of CIMS measurements. For example, Beck et al. [13], suggested that OOMs likely contribute to summertime particle growth at their Svalbard site, although the molecular composition of the condensing vapours were not determined. In contrast, Baccarini et al. [14] reported that the NPF events they observed over the central Arctic pack ice were driven by HIO_3,

with negligible contribution from sulfuric acid. This indicates that particle growth mechanisms vary regionally across the Arctic.

There is also a large body of literature regarding the growth of new particles or pre-existing particles in the Arctic. For example, Willis et al. [15] observed nucleation-mode particles between 5-20 nm growing into sizes above ~50 nm during a summertime particle growth event at the Canadian archipelago; the growth was correlated with organic compounds measured in particles of ~80nm and larger by an HR-AMS (>70 nm). And based on AMS observations (~80nm and larger), Willis et al. [23] reported that organic aerosol and MSA:sulfate ratios increased toward the surface and were positively correlated with air-mass residence time over open water in the Canadian Arctic; based on these correlations, they interpreted the aerosol as being strongly influenced by marine-biogenic sources and suggested that such organic material contributes to the summertime aerosol population that reaches CCN-active sizes. Burkart et al. [22] suggested that the growth of two modes of particles is largely via organic condensation, based on a comparison of modelling with the evolution of PNSDs. Croft et al. [21] used an aerosol microphysical model to show that condensable organic vapours are important for reproducing observed summertime aerosol size distributions above ~20 nm in the Canadian Arctic. They suggested that these vapours may have a marine, likely biogenic origin, while noting that the specific precursor emissions and formation mechanisms remain uncertain. Other work has pointed towards sea-surface microlayer derived aldehydes as a potential precursor for these organic vapours [46,109]. These led to the prevailing hypothesis that organic compounds facilitate particle growth to CCN sizes in the Arctic.

Our data supported the above hypothesis, confirming that particle growth is mainly driven by OOMs rather than inorganic acids, with low volatility (LVOC) and extremely low volatility (ELVOC) playing a key role. Furthermore, we estimated that the new class of OOMs that contain iodine contributes substantially to modelled GRs.

1.3 Molecular understanding from nucleation and growth to CCN

A key challenge in Arctic studies has been to observationally connect an NPF event all the way through to a measurable increase in CCN concentration. Previous studies typically estimated a size that would be CCN active [13,14,24]. For example, Beck et al. [13] speculate that atmospheric NPF observed at Ny-Ålesund, despite a rather slow consequent growth to Aitken mode size aerosol, may also contribute to the regional CCN budget. Baccarini et al. [14] provide a size distribution of cloud residuals, many of which have crossover with the diameter of growing particles, and therefore inferred that iodine NPF has the potential to influence CCN and cloud properties. PNSDs down to 5 nm and CCN measurements were made during NETCARE campaigns [24], and they suggested that marine secondary organic aerosol was a major CCN source, and that these particles could become CCN active at <50 nm, attributed to the low concentrations of larger particles. Song et al. [48] also found significant contribution of NPF to particles that could be CCN active in Svalbard. Therefore, previous evidence strongly suggests that NPF often contributes substantially to CCN in the Arctic. However, most available datasets do not track the same particle population continuously from nucleation to CCN activation in situ.

Our work provides a complete observational closure by tracking the full progression within a single particle population, from nucleation, to growth and CCN, at molecular level. This closes the

remaining evidential gap by directly demonstrating that nucleated particles grow to CCN-active sizes in the ambient Arctic atmosphere.

1.4 Remaining gaps and future work on molecular understanding from nucleation and growth to CCN in polar regions

While our study provides a more complete picture of Arctic NPF, it also highlights several key uncertainties that require further investigation.

Future work combining targeted field campaigns, capturing the whole NPF process (from emissions of precursors gases to nucleation, to growth, and CCN), with laboratory studies and regional modelling is needed to address these gaps and accurately represent these complex processes in climate models, for example

1) Spatially and temporally resolved emission fluxes of precursors like iodine, DMA, and aldehydes in the Arctic

2) The role of amines and ULVOC in nucleation processes, involving both iodine and sulfur species, through both laboratory simulations and field observations

3) The formation mechanism and atmospheric fate of the I-OOMs and aldehyde-OOMs, and their contribution to new particle growth spatially and temporally

4) Relative importance of NPF to the CCN budget spatially and temporally, and ultimately to clouds and radiative forcing in polar regions"

Comment 31: L 1140: Define OA

Response: We include the following amendment (line 941).

"The optimal solution was identified as a seven-factor model **describing different kinds of organic aerosol (OA),** consisting of hydrocarbon-like OA…"

Comments on the Supplementary Information file:

Comment 32: This file does not include Materials and Methods and Supplementary Texts as stated on page 1

Response: This has been corrected.

Comment 33: ED Fig. 1: Please provide a higher resolution version of this figure. Please make panel B larger. This is arguably a very important panel, but you squeeze many orders of magnitude into a very narrow space, while, e.g., temperature and chlorophyll-a both get their own panel.

Response: The figure has been updated.

Comment 34: Sometimes there are rings around the data (panel B). What does this mean? What are the black data points with error bars? Are they supposed to be shown here? Please explain everything that is shown in the plot also in the legend.

Response: For clarification, we added the explanation for those in the figure caption:

'The data points corresponding to the nucleation periods in Figure 3 are indicated along the line with circles. The averaged values, along with one standard deviation, are represented by black circles with error bars.'

Comment 35: This is not a mixing ratio of ions, it is a mixing ratio of neutral molecules that are charged in the instrument. Please remove the H^+ from the legend, as in the atmosphere this is not present and this figure is about the atmosphere.

Response: The designation "H^+" for each molecule has been removed from the legend.

Comment 36: Panel D: The legend for $C_2H_6SH^+$ is not blue as stated in the caption, but grey.

Response: It has been updated.

Comment 37: You should display all data with the same range (0-600 ppt), this would enhance comparability (and you could omit the right axis).

Response: The 800 ppt upper limit was retained because the total mixing ratio of all compounds on 13 June exceeded 600 ppt, and truncating it at 600 ppt would obscure this information. A separate axis for C_2H_6S was included to highlight its contribution.

Comment 38: Please add a short label directly to the figure indicating pink, grey and green background shading and make the shaded areas a bit less transparent, as their visibility is very low.

Response: The information regarding the pink, green, and gray shaded areas is included in the figure caption. We feel retaining these details in the caption would be helpful to keep each figure focused solely on the measurement data. Specifically, the caption states: "Pink shaded areas across all panels indicate NPF event days, and green shaded areas indicate burst event days. Gray shaded areas indicate days with significant contamination from own ship (i.e., when we mainly sampled the ship stack emissions)."

Comment 39: ED Fig. 2: Please explain what the box and bar upper and lower limits mean. What time periods were used for this Figure?

- You state: "Note: the ship emission days were filtered out from the non-event days."
- Shouldn't the ship emission days be filtered out from all the data, including also event days?

Response: Some days were discarded entirely due to ship emission. These are "ship emission days" as indicated in the grey day in Fig. S3. We make this clearer as follows, alongside a description of the box limits

"**In each plot, the red line indicates the median value, while the box represents the interquartile range (25th to 75th percentiles). The whiskers extend to data points within 1.5 times the interquartile range, and black dots show outliers beyond this range**. … Note: the days **dominated by** ship emissions, **denoted by grey boxes in Fig. S3,** were filtered out."

Comment 40: ED Fig. 3: Please add tick marks (not labels) to all horizontal axes.

Response: The figure has been updated.

Comment 41: ED Fig. 4: There are no grey diamonds and no grey circles in this plot, as stated in the caption.

Response: The corrected caption is as follows (following addition of another study after prompting from another reviewer):

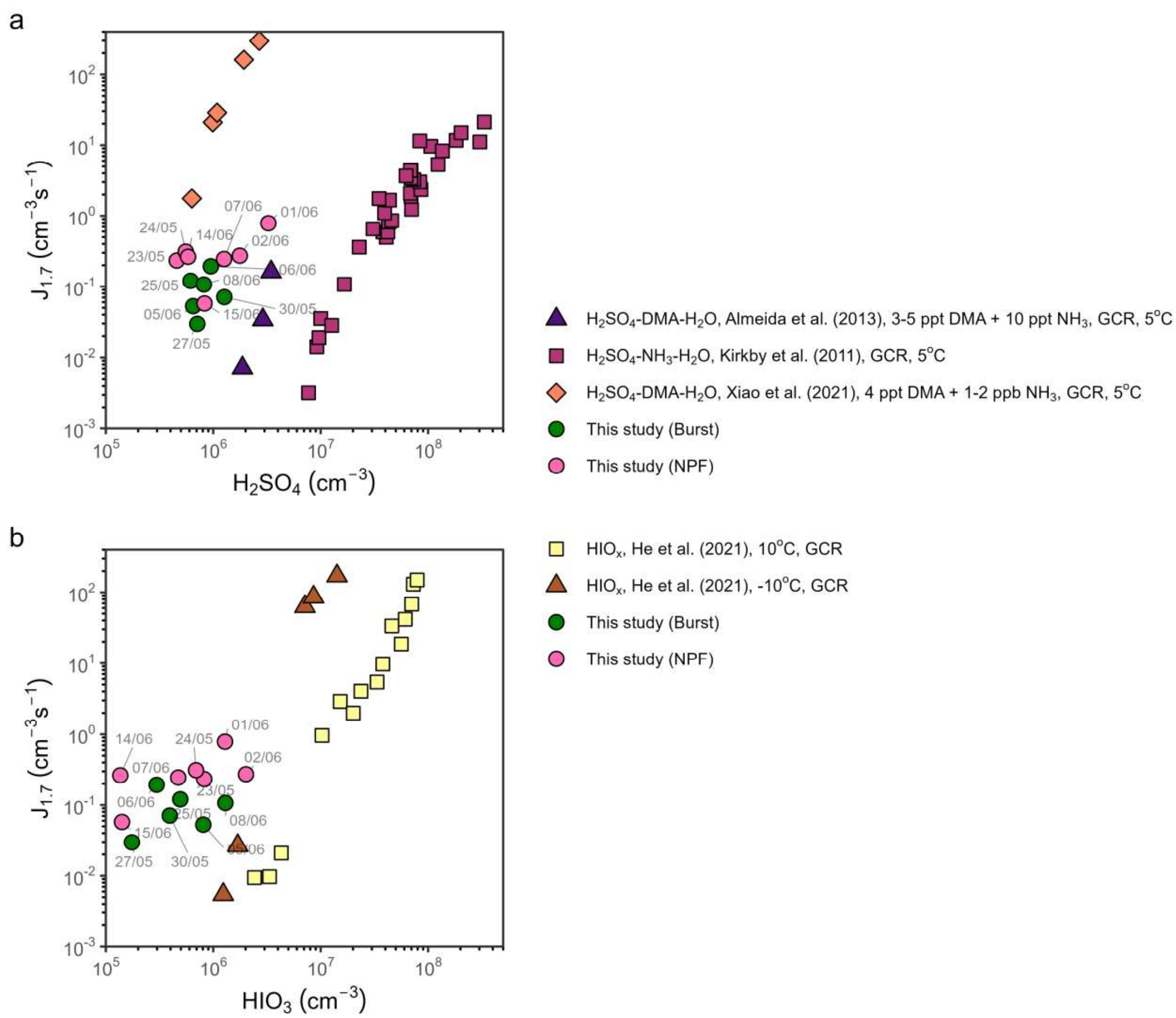

"Figure S8: Comparison of nucleation rates for the H_2SO_4, and HIO_3 systems with previous studies [10,11]. (A) Particle formation rates $J_{1.7}$ plotted against H_2SO_4 concentrations. Data points from CLOUD chamber experiments represent H_2SO_4-DMA-H_2O with 3-5 ppt DMA and 10 ppt NH_3 **(purple triangles)** [10], **H_2SO_4-DMA-H_2O with 4 ppt DMA and 1-2 ppb NH_3 (orange diamonds)** [12], and H_2SO_4-NH_3-H_2O (**magenta squares**) [10]. **The H_2SO_4-DMA-H_2O data from Almeida et al.** [13] **are presented without the self-coagulation correction later described by Kürten et al.** [14]**; this correction is not available for these specific low-concentration experiments and is negligible given the low particle concentrations, and the experiments of Almeida et al. are likely the most representative of our measured conditions.** (B). Particle formation rates $J_{1.7}$ plotted against HIO_3 concentrations. Data points are from CLOUD chamber experiments at 10 °C (yellow squares) and –

10 °C (brown triangles) [11]. **For both plots,** pink circles represent NPF events, while green circles denote burst events, and each data point is labelled on the plot using the day-month format.”

Comment 42: ED Fig. 5: Please add units for J and T in the legend. Why are the grey dots sized differently? This is not explained.

Response: We have added units to the legend. The sizes of the data points are clarified in the caption as follows

“Grey data points represent hourly averages. Larger colored data points are mean ion counts during nucleation period for each NPF or burst event.”

Comment 43: ED Fig. 6: Please explain the meaning of the various lines. Add the names of the targeted peaks as titles to the panels instead of keeping them in the caption text. Add panel labels (a, b, c,…). In panel f there are two red lines, please explain.

Response: We clarify this as follows:

“**Fig. S11. High-resolution peak fits for representative I-OOMs.** The sequence includes m/Q 376: $C_8H_{11}IO_5\ NO_3^-$; 424: $C_8H_{11}IO_8\ NO_3^-$; 439: $C_8H_{12}INO_8\ NO_3^-$; 466: $C_9H_{11}IO_6\ NO_3^-$; 468: $C_{10}H_{15}IO_9\ NO_3^-$; 473: $C_7H_{10}INO_{11}\ NO_3^-$ **and $C_{14}H_{22}INO_5\ NO_3^-$. Dashed blue lines represent fitted non-I-OOM peaks, red lines represent fitted I-OOMs, dashed green line represents the sum of fits, and the black line represents the raw data**. I-OOMs were assigned to mass spectral peaks when configurations of C, H, O, and N alone did not suffice. For instance, at m/Q 467.964, the only plausible formulas within 20 ppm, excluding iodine, were $C_{11}H_6N_2O_{15}NO_3^-$, $C_{23}H_2O_8NO_3^-$, and $C_{16}H_6O_{13}NO_3^-$, and there is no likely VOC oxidation pathway which would produce OOMs with these low H:C ratios. Elements such as chlorine and bromine were ruled out due to their isotope patterns. See SI Method 3 for a discussion of peak fitting and mass calibration.”

Comment 44: ED Fig. 9: Please label the color background in panel b.

Explain why only a small fraction of the I-OOM factor does actually contain an iodine atom.

Response: We have amended the figure caption (to save the repetition of ULVOC, LVOC…). It now reads as follows:

Figure S14: Characteristics of OOM sources. Output from PMF analysis of NO3- CIMS data showing: (a) The mass defect for each of the four factors, colored by the family (CHO corresponding to molecules that solely contain carbon, hydrogen, and oxygen; CHONI corresponding to those with carbon, hydrogen, oxygen, nitrogen, and iodine, and so on). Only the top 25% of signals per factor are shown. (b) The volatility distribution of each factor, colored by carbon number. **The coloured regions correspond to (pink) ULVOC, (grey) ELVOC, (yellow) LVOC, (green) SVOC, and (blue) IVOC.** (c) The average signal per carbon number in each factor, colored by the number of iodine molecules. The noise factor was not included here.

See our response to your Comment 27 on line 1016 r.e. the molecules within the I-OOM factor.

Comment 45: Fig. S3: Explain what the upper and lower limits of the boxes and bars mean.

Response: Clarified as follows:

"DY151 NPF formation and growth rates are illustrated in blue box plots. **In each plot, the red line indicates the median value, while the box represents the interquartile range (25th to 75th percentiles). The whiskers extend to data points within 1.5 times the interquartile range, and black dots show outliers beyond this range.** All data points for J_{10} are selected between 10 a.m. and 3 p.m."

Comment 46: Fig. S4: please keep the x-axis limits constant for all three mass spectra. **Fig. S7:** can you indicate when and why you did start the specific trajectories? Were these NPF and/or burst events? If yes, please add this information to every panel. Following up on our point above: what differentiates NPF from burst events, do you see a systematic difference **in** trajectories for the two types?

Response: For figure S4, this has been amended. For Figure S7, each plot contains hourly back trajectories for one day arriving at the location of the ship. There was no systematic difference between the trajectories, although one may emerge with a sufficiently large dataset. The extent of the data has been clarified as follows:

"Fig S20: 48-hour air mass back trajectories. The NOAA HYSPLIT model was utilized to calculate 48-h back trajectories for air masses arriving at the ship site. **Trajectories were calculated for each individual hour**. The date of nucleation events is shown in each subplot. The grey line represents the cruise track during the campaign. The purple circles represent the coastal cities."

We have signified whether the days are NPF or burst events in the plot now, as well as making it slightly nicer to read:

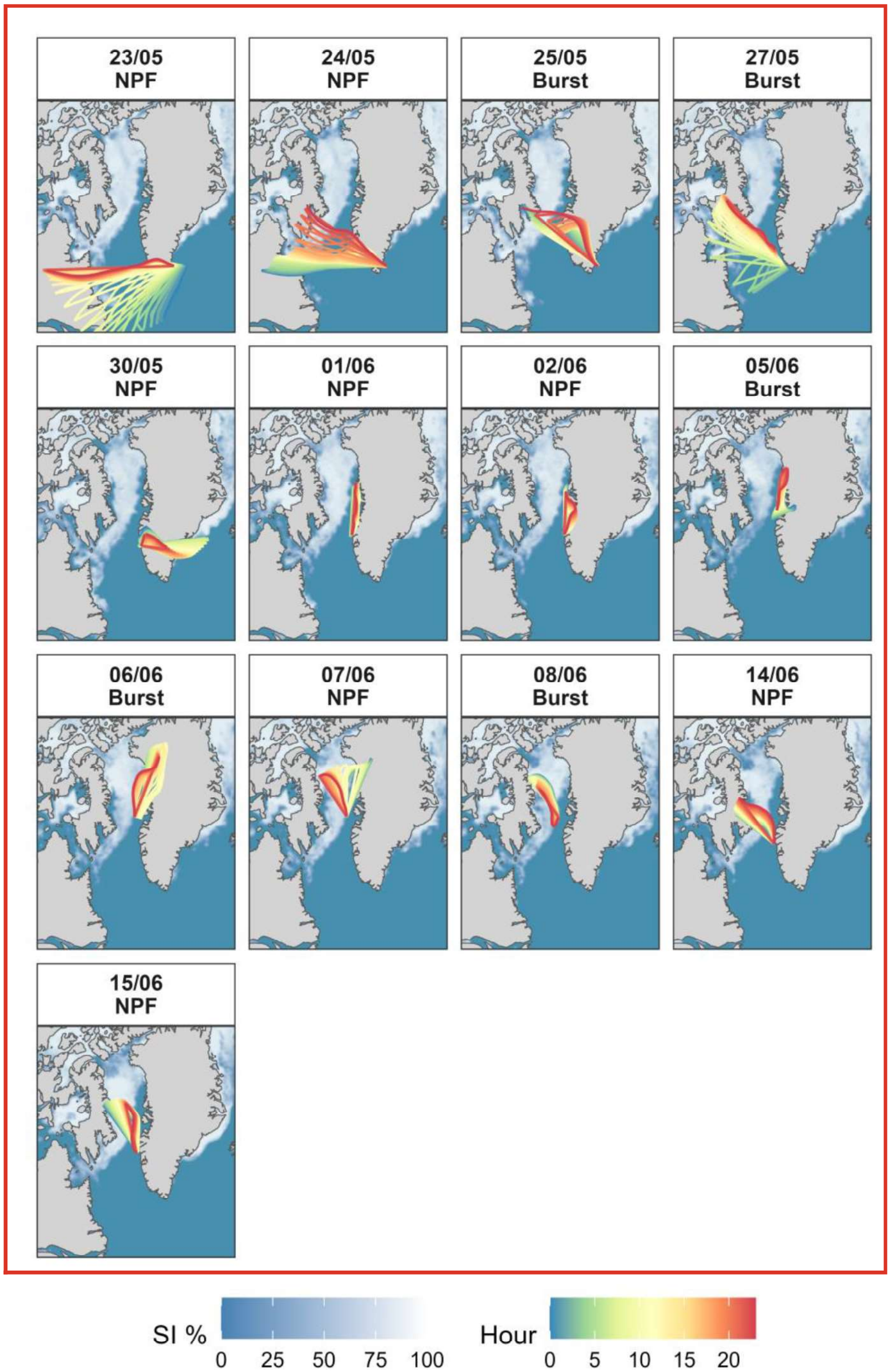

Figure redacted

Comment 47: Fig. S9: This plot was cropped a bit too heavy from the right, so that the last exponent is missing. Why is there an "A" if there is only one panel?

Response: Updated

Reviewer 3&4

Review of Synergistic iodine, sulfur, and organic-driven new particle formation enhances cloud-relevant aerosols in the Arctic

General comment: This manuscript presents a set of new, ship-based measurements off of the southern and southwestern coast of Greenland, primarily in the sub-Arctic regions of the Labrador Sea and Davis Strait, and in the Arctic region of southern Baffin Bay. Observations of gas-phase aerosol precursors and molecular cluster composition provide two key results, including evidence for (1) synergistic multicomponent nucleation (H_2SO_4+HIOx), and (2) low-volatility organics that grow resulting clusters toward CCN-relevant sizes. The authors propose that the growth of newly-formed particles is driven by a mixture of LVOCs, ELVOCs, and IOOMs, supported by an aerosol growth model fed by measured molecular formulas from a nitrate-CIMS and estimated volatilities. During a case study in which nucleation and subsequent growth to CCN-sizes was directly observed, PTR-MS measurements (with structural verification by GC-MS) suggest that this growth can be related to products of aldehyde oxidation. The aerosol growth model suggests a mixture of L/ELVOCs dominate the growth and AMS measurements suggest a high organic fraction after aerosols grew into AMS-measurable sizes. This growth occurred during a period when the measured airmass was travelling over the marginal ice zone in southern Baffin Bay. Overall, this work represents a comprehensive set of aerosol and aerosol precursor observations that furthers our knowledge of particle formation and growth processes in Arctic regions.

The methods are largely consistent with precedent in the literature, the data is high quality and uncertainties are usually communicated along with the various measurements. However, some key aspects require either thorough re-examination or clarification, including approaches to quantify small-particle formation rates, quantification of gas-phase aldehydes with the PTR-MS and justification for attributing certain classes of OOMs to specific precursors, especially aldehydes. The basic conclusions (i.e., H_2SO_4+HIOx nucleation; organic-driven growth) in this work are overall well-justified and clear but they focus too much on the novelty and not enough on the concrete implications of the results. The analysis and discussion, and thus implications, lacks broader context available in the sea ice biogeochemistry literature, and the back trajectory analysis used to link observed atmospheric chemistry to emissions from the sea ice environment is weak. Major concerns and specific comments follow.

General response: We thank the reviewer for recognizing the new results from our study, including: (1) synergistic multicomponent nucleation (H_2SO_4+HIOx), and (2) low-volatility organics that grow resulting clusters toward actual CCN, particularly the role of aldehyde-OOMs.

We appreciate the reviewer's attention to clarity and terminology, particularly related to "Atmospheric Implications" section, which we have re-written. We also addressed all technical questions.

Below, we have slightly re-ordered these comments to put those relating to the atmospheric implications adjacent to one-another, to avoid repetition on our part.

Major Comments:

Comment 1: Overstatement of conclusions, and unreasonable connection to "CLAW-like processes." The authors make a large leap of logic to connect H_2SO_4+HIOx nucleation mechanism to the CLAW hypothesis. This notion appears in the abstract (lines 51-52), and should be removed particularly because the authors do not show impacts on cloud radiative effects in their work. Related, the authors state that "More comprehensive and spatially balanced measurement are essential to generate accurate understanding of Arctic regions (L97-98); however, they seem to also be suggesting that their measurements at a single time and location somehow fulfill these criteria. On the discussion at line 365, the original CLAW hypothesis extended to increased cloud cover/albedo and a negative feedback loop in a warming climate. The authors are discussing one specific segment of CLAW (NPF precursors forming CCN), and have not established enhanced emissions, especially of HIOx precursors, due to warming. The authors have not linked explicitly to cloud radiative forcing (e.g., through a model analysis) and negative feedbacks on atmospheric temperatures.

Response:

We appreciate the reviewer's comment. We agree that we should be clearer when citing "CLAW" hypothesis, focusing on the segment linking ocean DMS emissions with CCN, and our references to potential impacts on clouds (the wider CLAW feedback) should be framed as hypothetical. We have revised the wording accordingly.

Following reviewer advice, we have removed direct reference to CLAW hypothesis in the abstract and rewrote the section related to CLAW hypothesis to clarify that our findings pertain only to a specific component of the hypothesis. We focus on a segment of the CLAW hypothesis: DMS emissions from the ocean cause the formation of cloud condensation nuclei (CCN). A key reason why CLAW hypothesis is contested because of limited evidence for new particle formation (NPF) contributing to CCN in the marine boundary layer. We found that marine emissions — not only DMS but also iodine compounds — can indeed lead to NPF and CCN formation. It is in this sense that our results support a ***specific segment*** of the CLAW hypothesis.

"Our findings confirm that in the Arctic atmosphere west of Greenland, it is the synergistic interaction between sulfur, iodine, and organic compounds that leads to efficient particle and CCN formation (Fig. 5). This demonstrates that ocean emissions of volatile gases can promote the formation of CCN in a pristine marine environment. The fundamental process underpinning this link is consistent with a key segment of the CLAW hypothesis [57] although the link we found here is more complex given the involvement of not just oceanic sulfur species but also iodine and organic compounds. "

The abstract has also been edited accordingly (line 49-52):

"Our findings demonstrate that iodine, sulfur, and organic NPF precursors in the Arctic enhance the abundance of CCN, through NPF, with **potential** impacts on cloud properties, radiation balance, and hydrological cycle~~, with positive feedback effects on ice melting [7]~~."

Comment 2: Implications for predicting changes in Arctic aerosol, cloud properties, and the cloud radiative effect in a warmer future. The authors invoke increasing marginal ice zone (MIZ) area in their introduction (Lines: 99) and closing (Lines 383-384) as a driver of increasing sulfur and iodine emissions. However, analyses of the satellite record does not suggest the MIZ area is increasing

annually. Rolph et al., 2020 (https://tc.copernicus.org/articles/14/1971/2020/) state: "We find no trend in the MIZ extent over the last 40 years from observations. Our results indicate that the constancy of the MIZ extent is the result of an observed increase in width of the MIZ being compensated for by a decrease in the perimeter of the MIZ as it moves further north." But, Rolph 2020 does find an increasing trend in MIZ fraction in August-September, due to a reduction in total sea ice extent. There appears no significant evidence for an increase in the MIZ area or fraction during melt onset and early summer, the period relevant to this study.

In the Atmospheric Implication section the authors make strong claims (e.g., line 374) about aerosol effects on “cloudiness;” however it is unclear whether the authors refer to cloud fraction or cloud reflectivity/emissivity. Do the authors assume that without NPF the Arctic is always in a CCN-limited regime? The subsequent discussion (Lines 376-377; 381-382) is missing a context of total cloud radiative effects and their seasonal cycle in the Arctic (i.e., this may not be so simple as enhanced cooling based on the seasonal cycle in CRF, see: https://link.springer.com/article/10.1007/s40641-016-0051-9), and does not provide useful statistical analysis (e.g., Lines 380, referencing Extended Data Figure 1) on the CCN concentrations on non-NPF days. For example, do those CCN concentrations fall consistently in a CCN-limited concentration range (e.g., based on Leaitch 2016 and Mauritsen 2011)? Is it only on NPF-days that CCN increases above a CCN-limited threshold (typically < 10-20 /cm^3; e.g., Leaitch et al., 2016)?

Response: We thank the reviewer for this comment. To improve clarity, we have revised the text to better reflect the complexity of the processes involved and to more clearly describe the potential impacts of future changes in marginal ice zones (MIZs) on sulfur and iodine emissions.

Thank you for pointing us to Rolph et al. (2020) [35]. They showed that there is no significant long-term trend in the total extent of MIZ, which is defined as the area of grid cells with 15–80 % sea-ice concentration over 1979–2018, based on passive-microwave sea ice concentration data and reflects an area, threshold-based metric. As they [35] and others note [36], the widening of the MIZ is compensated by the move poleward (so less sea ice coverage). We incorporated this important context into our revised manuscript.

From a biogeochemical and air–sea exchange perspective, the permeability and fragmentation of the ice is are primary controlling factors. We have incorporated findings from the IPCC Special Report on the Ocean and Cryosphere in a Changing Climate (Meredith et al., 2019) to highlight that the Arctic is transitioning from thick, multi-year ice to thinner, first-year ice [37]. This thinner ice is significantly more vulnerable to fragmentation by ocean swells and cyclones [38, 39], effectively expanding the "emissions-active" zone even if the satellite-defined MIZ area remains constant.

We now address the nuances about how MIZs have changed and might change in the warming Arctic, incorporating both physical and biological drivers of emissions:

1. Physical Fragmentation: Further reductions in seasonal ice cover and thinning ice allow deeper wave penetration into the pack, expanding the wave-dominated MIZ regime [38]
2. Under-Ice Production: Thinner ice allows sufficient light transmission to support intense phytoplankton blooms under the ice and within leads, creating emission sources within the pack that are not captured by traditional MIZ definitions [40, 41].

3. MIZ Fraction: As noted by Rolph et al. (2020), the fraction of MIZ to total ice is increasing as the ice extent decreases [35].
4. Poleward Shift: MIZs are shifting northward [37].
5. Future Dominance: Recent analyses of CMIP6 HighResMIP models project that the MIZ will become the dominant ice condition in the summer Arctic, potentially comprising over 90% of the total ice cover by 2050 [42], corroborated by other studies [43]

We understand that 1), 2), 3) and 4) will alter when and where the volatile gases are released to the atmosphere at MIZs. But we do not have sufficient knowledge on how all five above-mentioned changes in MIZs in the future will affect such emissions. Considering the above, we revised our manuscript accordingly.

Firstly, we amended Line 99 as follows (line 103-107 in new manuscript):

"More comprehensive and spatially resolved measurements are essential to generate an accurate understanding of Arctic regions [25]. All this is important for predicting changing Arctic climate into the future, where, for example, emissions of gas-phase iodine and dimethylsulfide (DMS) - the major marine biogenic sulfur compound for aerosol formation, are increasing [12,26-29]"

And line 374 states the following, meaning that our framing of the MIZ is as a 'hotspot of precursors' in this work (line 444-453 in new manuscript):

"Furthermore, the faster Arctic warming accelerates sea ice and glacier melting, leading to more frequent under-ice phytoplankton blooms [59] and enhanced river runoff [28]. Warming climate also enhances ocean productivity in the Arctic [28]. All these processes can potentially increase emissions of iodine, sulfur, and VOCs, thereby promoting more frequent NPF events [12]. Warming will drive marginal ice zones northward and increase their width, altering the spatial and temporal distribution of emissions. Although the total area of the marginal ice zone has historically remained stable [60-62], models project it will become the dominant ice condition in the summer Arctic in the future [63,64]. These changes will extend marginal ice zone-related NPF to higher latitudes where permanent sea ice exists today [13], with potential impacts on ice melting and thereby regional and global climate."

Furthermore, we agree that we need to be clearer with the term "cloudiness". In our revised manuscript, we have replaced this with more specific language, referring to the potential for NPF to influence "cloud properties, such as cloud droplet number concentration and reflectivity".

Regarding whether the Arctic is in a limited regime, Mauritsen et al. show that this is, roughly speaking, for CCN concentrations $<10\ cm^{-3}$ [44]. In our measurement set, the CCN number concentrations never dropped below $10\ cm^{-3}$. We do not have data to clarify whether and to what extent such background CCN is from NPF. However, Abbatt et al. (2019) [6] showed that NPF contributes to CCN in the Canadian archipelago and Song et al. (2023) [45] suggested that secondary and nucleation-derived aerosols dominated potential CCN (defined as particles >40nm).

We took all the PNSD data from Brean et al. (2023) [46] for the months of May and June and calculated the fraction of time when particle number concentrations of >50 nm particle was $<10\ cm^{-3}$, and find that, depending on the site, this is anywhere between 1 and 6% of the time (Alert, 4.10%;

Utqiaġvik 1.01%; Gruvebadet, 1.47%; VRS, 6.51%; Tiksi 2.42%; Mt. Zeppelin, 2.79%), see below figure

The full section here now reads as follows (line 433-443):

"The efficient formation of CCN from these natural precursors has the potential to influence Arctic cloud properties, such as cloud droplet number concentration and reflectivity. This is particularly important in the pristine Arctic atmosphere, where clouds are often sensitive to perturbations in CCN concentrations [3,5,24]. While conditions considered to be strongly CCN-limited (e.g., CCN <10 cm^{-3}) occur periodically across the wider Arctic in May and June (Fig. S21), background concentrations in our study region remained above this threshold (Fig. S2) though some of such background CCN likely originated from NPF processes. Furthermore, the NPF events we observed caused order-of-magnitude increases in CCN, representing a substantial perturbation to the regional aerosol population. The net radiative effect of such changes is complex, potentially leading to surface warming over high-albedo snow and sea ice or a cooling effect over the open ocean [57,58]."

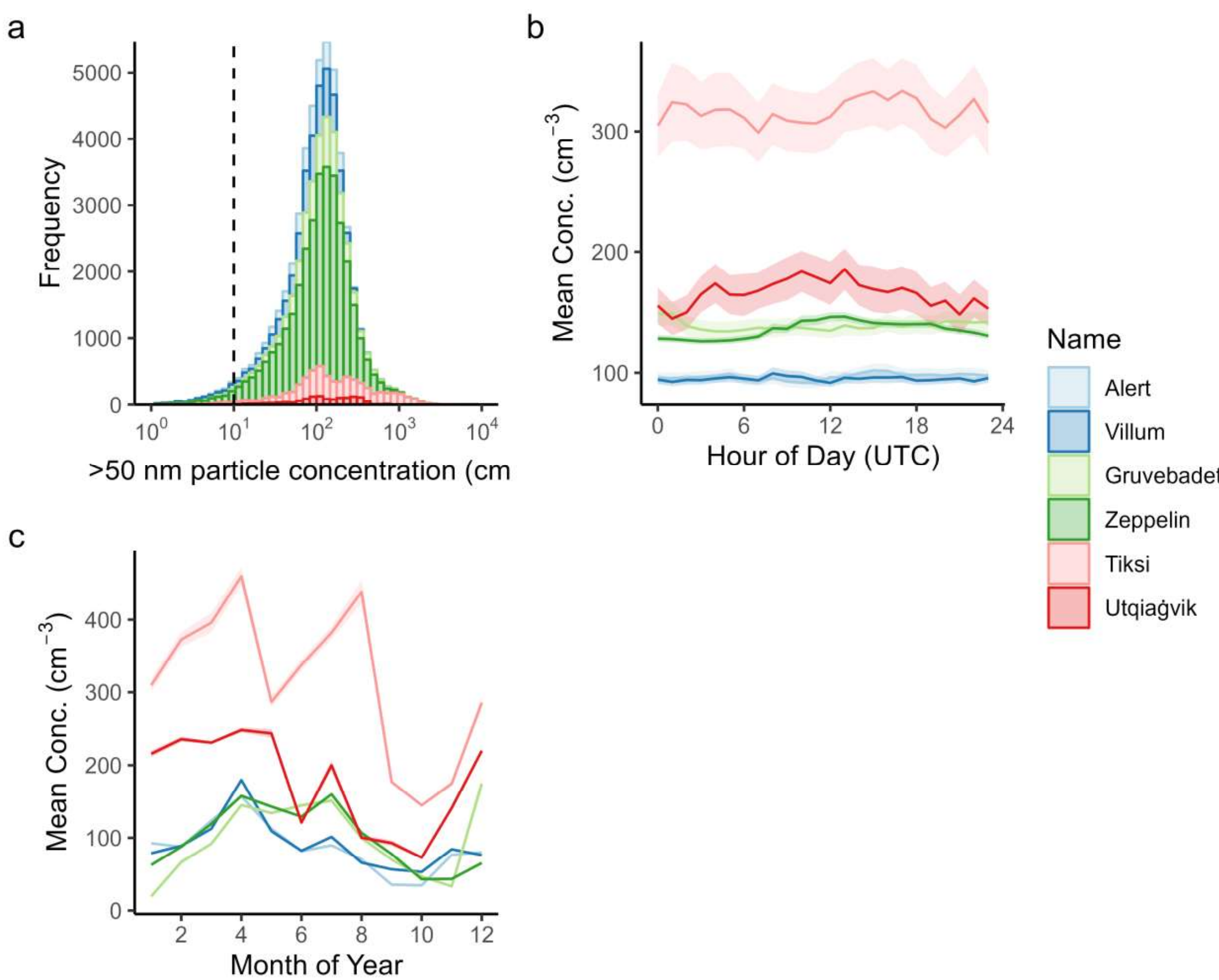

Fig. S21: (a) The frequency of different >50 nm particle concentrations in the Arctic for the months of May and June. Dashed line represents 10 cm^{-3}. The frequency with which particle concentrations are below this threshold are: Alert, 4.10%; Villum Research Station, 6.51%; Gruvebadet, 1.47%;

Mt. Zeppelin, 2.79%; Tiksi 2.42%; Utqiaġvik 1.01%. (b) the diurnal cycle of >50 nm particles. (c) the seasonal cycle of >50 nm particles. Dataset is as in Brean et al., (2023) [9].

Comment 3: Establishing a causal link between atmospheric observations and melting sea ice (e.g., Lines 251-253). The authors seek to broadly establish a link between their observed nucleation and growth mechanisms and melting sea ice, such that future decreases in sea ice extent would seem to suggest potential for large enhancements in NPF and resulting CCN. However, this inference is tenuous at best. The authors assume that all precursors they measure arise from sea ice melt (e.g., Line 252, 344), but do the authors simply assume active melt because of the time of year? What information about sea ice characteristics are they considering? Back trajectory analysis is cursory (e.g., L268-269; Figure 4); for example, no information is provided on the height of the trajectories over 48 hrs. Though described at Lines 1059-1060, the authors do not show a systematic association between high aldehyde concentrations and airmass back trajectories over sea ice, let alone "melting sea ice," for all days with influence from the ice edge. What metrics are the authors using to assess active melt processes and link these to the aldehyde measurements? What is the lifetime of these aldehydes, and how do the authors know that they arise from the sea ice, rather than for example, the surrounding coastal regions? The authors cannot say whether the iodine source is simply "the ice" (Lines 315-317), meltwater around sea ice, snow on sea ice, or high salinity surfaces of sea ice itself. The authors seem to implicate the (presumably saline) surface of first-year ice in the MIZ; however, much of the ice in this region is likely either snow-covered, or covered by melt ponds. Brown et al., 2025 (https://doi.org/10.1039/D4FD00178H) use speciated iodine distributions in first year sea ice and overlying snow during MOSAiC to show evidence against an upward source of iodine from the ice into the overlying snowpack, and shows that iodine in snow likely comes from above (i.e., deposition of iodine-compounds or iodine-containing aerosol on the snow, which is then available for photochemical activation). The source of iodine to snow is relevant, because photochemistry in saline snow is a critical pathway to I_2 production in polar regions (e.g., Raso et al., 2017: https://doi.org/10.1073/pnas.1702803114, which is not cited by the authors). Baccarini et al (the author's Ref 16) showed that HIO_3-driven NPF was associated with decreasing temperature, ice freeze-up and higher O_3 concentrations -- they did not definitively demonstrate that iodine activation takes place on the ice. The authors may then be suggesting the organo-iodine compounds are the source of HIOx; however, they have no evidence to establish this. Overall, the authors seem to struggle to situate their work in the wider literature on polar marine boundary layer chemistry. The sea ice systems the authors are considering are dynamic biogeochemical systems, but the authors seem to treat them as a "black-box" type source of the aerosol precursors they measure, without regard to the wider literature about the chemical, physical and biological dynamics of the sea ice present in Baffin Bay during their study. For example, the authors discussion (Line 323-326) of their DMS measurements is cursory, and they miss the growing body of literature suggesting that methanethiol (MeSH) is an important marine biogenic sulfur emission. Do the authors observe MeSH with their PTR-MS? Chl-a is known to be a poor tracer of DMS emission potential. The authors are not considering the literature on dissolved DMS concentrations around and under sea ice in their study region. Further, in the Atmospheric Implication section (L371-372), the authors then extend their observations to become applicable to "sea ice, coastal regions, and open ocean." This is not addressed in the paper -- their analysis describes the MIZ as an important source based on 48hr back trajectories in a single NPF event.

Response: We thank the reviewer for clarity. We have substantially revised our framing, aiming to provide a more accurate, nuanced, and scientifically robust discussion.

We have revised the relevant sentences (e.g. lines 252 and 344) to clarify that marginal ice zones (MIZs) represent one of several potential sources of NPF precursors, alongside contributions from snow on ice and biological activity. We have revised the statement "The importance of our observed NPF processes is likely to manifest throughout polar regions where the ice is melting in larger areas, including in the Antarctic[47] " to (line 402-404):

"The importance of the observed NPF processes is likely to manifest throughout polar regions, including in the Antarctic [56]. This is true in a warming world even though multiple processes — not only sea-ice melt — can contribute the precursor vapours."

Furthermore, we now consistently refer to the "marginal sea ice zone (MIZ)" as a biogeochemically active *environment* and a "hotspot for precursors". We have also incorporated the suggested literature to better acknowledge the complexity of iodine and sulfur cycles.

On the other hand, on the key source of NPF precursors, we do believe there is a strong link to the MIZ. In observational atmospheric science, it is often impossible to establish a causal link in the way one could in a controlled laboratory experiment. Instead, we build a case on a convergence of evidence. In our key case study, we have three powerful, converging lines of evidence:

Firstly, the air mass trajectory shows a direct and prolonged pathway over the very edge of the sea ice. Secondly, the measurements were made in June, the peak of the seasonal melt, and third, the event occurred on a relatively warm and sunny day, conditions that maximise any melt-related and photochemical emission processes.

The approach that the reviewers suggest, such as looking at aldehyde concentrations for all trajectories which have travelled over the marginal sea ice zone, is not possible given the nature of a cruise dataset, however, we argue that the preponderance of the evidence suggests that these aldehydes and other VOCs come from the marginal sea ice zone (versus from the ocean covered by sea ice).

While we have revised the manuscript to be more precise about the specific processes at play, we believe that this convergence of evidence makes the MIZ environment as a unique and scientifically parsimonious source for the unique mix of precursors we observed.

This is consistent with literature where long-term studies show that NPF is enhanced due to sea ice melt [48], and in the Antarctic the sea ice is a source of amines and other organonitrates [25, 49, 50]

We believe the revised text now better reflects this evidence-based conclusion (line 364-417)

"Our measurements demonstrate that HIO_x and H_2SO_4, alongside a likely involvement of amines and OOMs synergistically and simultaneously drive nucleation in the Arctic, while subsequent growth is primarily driven by a wide range of oxygenated organic molecules, including I-OOMs. We further demonstrate that new particles can grow rapidly to become CCN in the study region. These findings reveal complex and diverse natural NPF sources and processes in the Arctic, leading to CCN formation (Figure 5). We observe nucleation on >80% of the days where peak solar radiation exceeds 600 W m-2, although this did not always occur with subsequent growth. It

indicates that nucleation may be widespread across the Arctic, although missed by existing measurements which typically have not captured sub-5 nm aerosol concentrations.

Precursors of HIO_x and I-OOMs have diverse sources, including coastal regions and the marginal sea ice zone. The elevated HIO_x concentrations observed when air masses passed over the marginal sea ice zone (Fig. 4A) point to this region as a key source. The precise mechanisms are complex, likely involving a combination of emissions from open leads, biological activity in surface waters, and photochemical processing of iodine deposited on the snowpack, a pathway known to be critical for I_2 production in polar regions [49,50]. The good correlation from the I-OOM factor extracted by PMF analysis of NO_3^- CIMS data and HIO_3 (Fig. S12), suggests that they originate from similar sources.

A key precursor of H_2SO_4 in remote atmosphere, DMS, is produced from both ocean and sub-ice biota [11,27]. We observed elevated oceanic chlorophyll-*a* near the marginal sea ice zone (Fig. S2) and directly observed thriving algae beneath the ice packs (Fig. S18). While a simple proxy, the consistent link between the marginal sea ice zone and elevated DMS points to biological productivity of this region as an important driver of sulfur emissions. Once oxidized, DMS can form both H_2SO_4 and MSA, which are correlated in our data (Fig. S19).

In the marine environment, the natural sources of amines in the Arctic include biodegradation of organic matter containing nitrogen compounds [39]. Our NO_3^- CIMS did not detect gas-phase amines, but they were detected in a cluster with HIO_3 and HIO_2 (Fig. 2A and Figs. S5-6). NH_3 can be from marine sources, bird colonies,[33] and tundra soils [51] in the Arctic, although the ocean is a net NH_3 sink [52] and its concentration is below the detection limit (<65pptv) during our campaign.

VOCs come from both abiotic and biotic sources. For example, aldehydes can be emitted from the ocean via abiotic processes in the sea-surface microlayer, i.e., ozone or photochemical oxidation of fatty acids [42,46]. Our study supports this mechanism because concentrations of aldehydes peaked when air masses flew over the coast and marginal sea ice zone (23rd-24th May, 1st, 5th June and 8th June, see Figs. S2D and 20). Monoterpenes are emitted from the ocean, although at lower rates than from most land regions [53], but we show that marginal sea ice zone is a hotspot where highest monoterpene concentration was observed (Fig. 4A and 4E). Both aldehydes and monoterpenes readily undergo autooxidation to form OOMs [39,54].

HIO_x and H_2SO_4 are likely ubiquitous across the marine Arctic [8,13,14], and we argue that the synergistic nucleation process we observed is therefore likely ubiquitous across high latitude marine environments. The importance of the observed NPF processes is likely to manifest throughout polar regions, including in the Antarctic [55]. This is true in a warming world even though multiple processes — not only sea-ice melt — can contribute the precursor vapours. Nucleation rates can be enhanced by NH_3, which is abundant (>100 pptv) in some regions of the Arctic with bird nests, but predicted to be almost absent in the ice-covered high-latitude areas (<1 pptv) and central ocean regions [33,52]. Amines (as detected in the DMA-containing cluster in Figs. S9-10) and OOMs can further enhance nucleation rates.

The concentration of any individual component will be dependent on the rates of emission and oxidation processes of primary emitted volatile gases (such as VOCs, OVOCs, I_2, DMS, DMA, and NH_3, Fig. 5) [39]. As a result, the relative importance of different molecules to nucleation and growth will differ seasonally and spatially (Fig. S1). Similarly, OOMs concentrations are likely low in the ice-covered higher Arctic, for instance, in the central Arctic pack ice region, leading to limited new particle growth and CCN formation [7,10,13,14], while OOMs seem to be more abundant in the Arctic and sub-Arctic seas and marginal ice zone, resulting in faster growth of new particles (Fig. 4 and Fig. S17) and contributing to more CCN formation (Fig. 5)."

And also with the following statement (Lines 444 – 453):

"Furthermore, the faster Arctic warming accelerates sea ice and glacier melting, leading to more frequent under-ice phytoplankton blooms [59] and enhanced river runoff [28]. Warming climate also enhances ocean productivity in the Arctic [28]. All these processes can potentially increase emissions of iodine, sulfur, and VOCs, thereby promoting more frequent NPF events [12]. Warming will drive marginal ice zones northward and increase their width, altering the spatial and temporal distribution of emissions. Although the total area of the marginal ice zone has historically remained stable [60-62], models project it will become the dominant ice condition in the summer Arctic in the future [63,64]. These changes will extend marginal ice zone-related NPF to higher latitudes where permanent sea ice exists today [13], with potential impacts on ice melting and thereby regional and global climate."

Regarding the trajectory heights during Figure 4, we include the following sentence (line 304-307):

"The event on 7th-8th June occurred as the air masses flowed over the marginal sea ice zone, giving us the opportunity to examine NPF precursors and particle growth in an environment influenced by processes associated with the MIZ (Figs. 4A, 4C, and S9), **with >90% of these trajectories have a height <200 meters**."

Regarding MeSH, unfortunately, we cannot consistently resolve MeSH from two large, adjacent peaks. We therefore do not provide this data.

Comment 4: Atmospheric implications and connection to global models of polar aerosol processes. The atmospheric implications section in particular demonstrates a lack of synthesis of the author's observations and the existing literature on Arctic ocean – atmosphere interactions, sea ice biological and physical dynamics, and their potential implications for cloud properties. Some examples follow:

1) **Line 341-343:** The authors claim that synergistic nucleation is "likely ubiquitous" throughout Arctic regions, citing two publications (Ref 15, 16) that observed H_2SO_4-driven and HIOx-driven nucleation, with techniques similar to that used here, separately in distinct locations. In other places in their text, the authors discount the utility of those studies because they had not thoroughly and directly followed the processes of nucleation and growth to CCN sizes at a molecular level (see major comment #6), but they lean on them here to demonstrate the ubiquity of relevant aerosol precursors in the Arctic, even though these past studies did not detect synergistic H_2SO_4-HIOx nucleation.

2) **Line 396 – 399:** The statement beginning “we contend…” is not a direct implication of this study, nor is it an original or insightful conclusion – this is well known within the literature around this topic. The author’s work has added to it, no doubt, by highlighting yet another nucleation process that is not represented in most global models.

3) **Line 400:** What do the authors identify as interdisciplinary in their observations? This is an interesting statement given that the authors have not leveraged the large body of literature around ice and ocean biogeochemistry to support and interpret their observations.

4) **Lines 403-404:** The use of this closing statement for the main text compels this reviewer to ask how much progress we will make by simply calling for these processes to be included in global models, when climate models do not typically even run a coupled ocean and atmosphere, and simply treat sea ice as a cap for emissions? Have the authors considered what underlying emission processes and chemistry would be needed, and could they make a concrete and useful recommendation to the modelling community? There exists a missed opportunity here to make new, quantitative recommendations based on this work, beyond the restatement that global climate models miss nucleation due to a lack of process-level representation, which is already well known and will not be improved without measurement and modelling communities working together.

Response: We carefully considered these comments and revised relevant sections integrating our results in the context of literature.

1) References 15 and 16 are cited for their detection of HIO_3. On this basis, it is plausible that HIO_x–H_2SO_4 nucleation may contribute to new particle formation, as discussed by He et al [1]. To avoid any ambiguity regarding this point, we have revised the text by adding the phrase “considering that HIO_3 is detected in multiple locations” to clarify the context in which these references are cited. A more detailed discussion of our treatment of Baccarini et al. and Beck et al. is provided in our response to reviewer 1&2 Comment 30.

2) We agree and remove the statement beginning “we contend…” .

3) We thank the reviewer for this helpful comment. Our intention was to highlight the value of combining observational field data with laboratory studies in future work. To avoid any ambiguity, we have removed the term “interdisciplinary” from the text and revised the wording accordingly.

4) We thank the reviewers for prompting us to substantially revise the final paragraph. We now conclude the main text as follows (line 457-470):

“Considering that sulfur, organic and iodine sources are widespread in other parts of the world, the mechanisms we showed here are likely relevant globally, however, a key obstacle for global models is how to represent this observed chemical complexity. Updating the core nucleation mechanisms within models to include the synergistic interactions between H_2SO_4, HIO_3, and key stabilising species including DMA, supported by laboratory kinetic studies, would better represent the real world. This moves beyond treating these as separate pathways and reflects the synergistic mechanism now observed in both the laboratory and the field. To be effective, such updates must be underpinned by improved emission inventories. This is especially critical for the Arctic, where the environment is undergoing rapid transformation with changes in emissions of volatile gases, such as

increasing emissions of marine iodine species [26] and DMS [27] alongside anthropogenic pollutants such as SO_2 which will likely perturb these natural processes.

Furthermore, progress will require coordinated efforts combining intensive and long-term field measurements—capturing the full NPF sequence from precursor emissions through nucleation, growth, and CCN formation—with laboratory studies designed to reproduce real-world chemical complexity, including those investigating aldehyde-derived OOM and iodine-organic chemistry. Such integrated approaches are essential for understanding the temporal and spatial variability of these processes and their implications for clouds and radiative forcing."

Comment 5: Context for observed J_10 and CCN number observations. The authors point out that their observed J_{10} and CCN concentrations are larger than other Arctic observations. This is not particularly surprising given the lower latitude of these measurements compared to the field sites the authors compare to; however, little context is given for how these comparisons are made – e.g., are they only compared for the months of May and June? For the same years, or different year? The comparison given at lines 280-281 suggests that the CCN measurements in the June 7-8 growth event are being compared to mean or median values in other campaigns. NETCARE is mentioned, but the cited references are not in the same regions. The authors neglect to compare to Collins et al., 2017 (https://doi.org/10.5194/acp-17-13119-2017) which sampled in similar regions of Baffin Bay and observed notably high growth rates, similar to and higher than that observed in this work.

Response: Thank you for directing us to Collins et al. We have included this in our text as follows (line 269-272):

"GRs for particles of diameters 3 to 15 nm varied between 1.5 to 3.6 nm h^{-1} (Table S1), which are relatively high among those observed in **long-term data in** the Arctic (Fig. S3B), **but akin to measurements made in the region west of Greenland [40]**."

Regarding Figure S3, we reproduce the figure utilising exclusively May and June, and include our revised figure below:

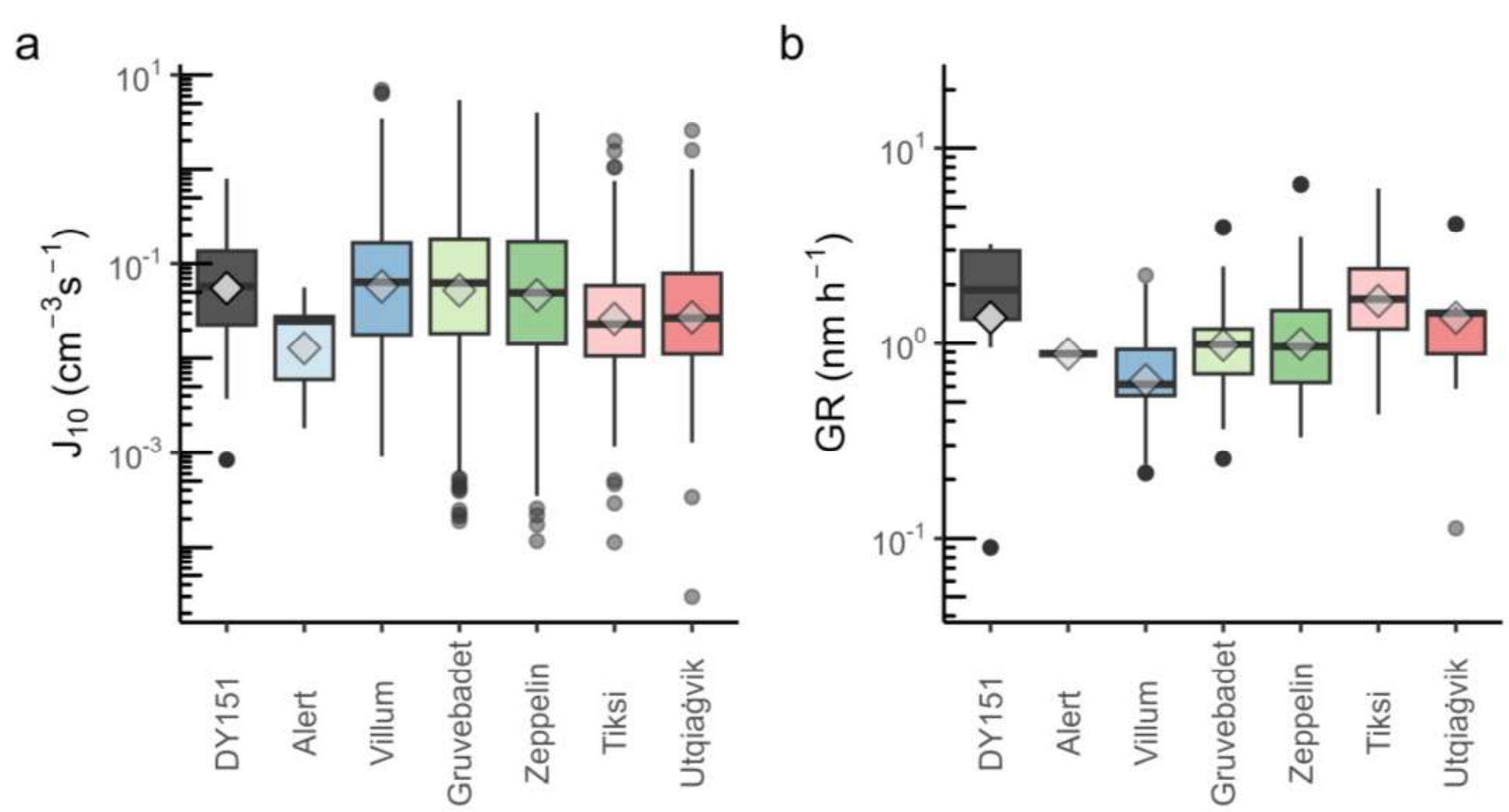

"Fig. S3. Comparison of Formation and Growth Rates: DY151 alongside other Arctic research stations. (A) Formation Rate; (B) Growth Rate. J_{10} denotes the formation rate of particles at 10 nm utilizing the 10-25 nm size range, while GR is computed for particles exceeding 10 nm. Long-term datasets from key Arctic stations—Alert GAW Observatory (Alert), Utqiaġvik (previously Barrow), Gruvebadet, Villum Research Station (Villum), Tiksi, and Zeppelin Mountain (Zeppelin) are depicted in grey box plots (source: Brean et al. [9]). **Data for May and June exclusively plotted for all available years.** DY151 NPF formation and growth rates are illustrated in blue box plots. In each plot, the red line indicates the median value, while the box represents the interquartile range (25th to 75th percentiles). The whiskers extend to data points within 1.5 times the interquartile range, and black dots show outliers beyond this range. All data points for J_{10} are selected between 10 a.m. and 3 p.m. (UTC time) each day to indicate the nucleation periods. Most NPF events for TIK occurred under polluted continental airmasses and thus are not representative of the Arctic marine environment."

Comment 6: Identification of monoterpene-rich, aldehyde-rich and iodine-containing OOMs from PMF analysis of nitrate-CIMS spectra. While this reviewer does not doubt that measured aldehydes contribute to formation of OOMs and thus particle growth, the logic provided for the assignment of monoterpene-rich and aldehyde-rich OOMs leaves significant ambiguity. The aldehyde factor is also dominated by C_{10} and C_9 species (Line 222, Extended Data Fig 9). What percentage of the total concentration is contributed by the top 50 peaks (Lines 1038-1039)? Can the authors show more quantitatively the overlap between monoterpene-rich and aldehyde-rich factors? A more quantitative description of overlap in specific peaks are and how important they are to each factor is needed here. Further, how does the aldehyde-rich factor related with aldehydes measured in the PTR-MS; the reader is left to try to infer this across multiple figures in the Extended Data. The factor identified as I-OOMs is described as Dominated by I-OOMs (Line 1016), but by what metric? Larger number of peaks in this factor have zero I-atoms, though this factor does appear to contain the largest fraction of I-OOMs. Best evidence seems to be correlation with HIO_3. The fact that the I-OOM factor contains more peaks without iodine than with iodine suggests this classification is potentially misleading.

Response: We believe our PMF factors represent distinct sources. Due to the lability of the carbon-iodine bond, it could be that some I-OOMs form but then subsequently lose the iodine molecule, or the reaction pathway that forms them involves iodine but does not necessarily impart iodine into the resultant OOM. To avoid confusion, we renamed our I-OOM factor to Iodine-related OOMs. We clarify this as follows (Line 1187-1192):

"… the largest iodine-containing peaks are $C_8H_{11}IO_5\ NO_3^-$, $C_{10}H_{15}IO_9\ NO_3^-$, and $C_8H_{12}INO_8\ NO_3^-$ (Table S4). **Some of the peaks in this factor do not contain iodine. This could be related to the reaction pathway that forms them involves iodine but does not necessarily impart iodine into the resultant OOM, and/or some I-OOMs lost the iodine molecule later**."

Regarding our use of the top fifty peaks, the majority of signal resides in these peaks, which we clarify as follows (line 1213-1217)

"Of the 50 largest peaks in each spectrum (**accounting for 70% of the concentration for these factors**), only 12 are found in common between both factors. **These shared peaks account for 22%**

of the concentration of the monoterpene-rich factor and 11% of the concentration of the Aldehyde-rich factor. That is to say, these factors are dominated by different sets of OOMs."

The reviewer requests a clearer relationship between the aldehydes measured by the PTR-MS and the 'aldehyde-rich' OOM factor. A direct, quantitative correlation is challenging for two main reasons. Firstly, as we note in the manuscript, aldehydes are known to fragment significantly in the PTR-MS, making it difficult to deconvolve the concentrations of individual precursor aldehydes. Secondly, directly linking specific precursors to their oxidation products in a complex, mixed atmospheric system is an established challenge in the field. Therefore, our assignment of the 'aldehyde-rich' factor is not based on a single correlation, but rather on two strong, converging lines of evidence which we believe collectively make a robust case (line 1218-1224):

"The chemical link between aldehydes and our aldehyde-rich factor is established by the clear overlap in carbon number distribution between these measured precursors and their oxidation products; the aldehyde-rich PMF factor is dominated by C_9 to C_{15} OOMs, a range that is fully consistent with the oxidation of the aldehydes we measured (see Fig. S15). Furthermore, the time series of this PMF factor is highest on days when high concentrations of gas-phase aldehydes were observed in airmasses originating from the marginal sea ice zone, strengthening the link between the precursors, their sources, and their ultimate contribution to particle growth."

Comment 7: Novelty and originality of molecular cluster composition measurements, and linking to progression to CCN. The authors seem to discount the work of Baccarini et al., 2021 that shows HIO_3-driven growth to CCN in the high Arctic (>70N) during autumn freeze-up. While true that Baccarini et al., do not show H_2SO_4 as important and thus do not show evidence for synergistic H_2SO_4+HIOx nucleation, they do provide observations showing HIO_3 drives nucleation and growth. Therefore, this reviewer disagrees that the present work is the first to connect nucleation and growth to CCN in the Arctic.

Response: We agree that Baccarini et al. (2021) "do not show evidence for synergistic H_2SO_4+HIOx nucleation, and they do provide observations showing HIO_3 drives nucleation and growth". We now clearly stated that (line 98-99)

"Baccarini et al. [14] provide a size distribution of cloud residuals, many of which have crossover with the diameter of growing particles, and therefore inferred that iodine NPF has the potential to influence CCN and cloud properties."

We also clarify that "CCN-relevant particles" refers to "particles reached diameters that *could* serve as CCN under certain supersaturations" – this typically refers to a particular size cut, such as 30 or 40 nm. They are fundamentally different to CCN that was measured using a CCN counter – e.g., we found here the CCN critical size is often larger than 80nm.

Please see response to comment 30 by Reviewer 1&2 and to comment 8 below.

Comment 8: Novelty and originality of oxygenated organic molecules driving growth of particles to CCN sizes in the Arctic (L88-92; Supplementary text Section 7). This reviewer acknowledges that direct evidence for aldehydes contributing to particle growth is a strong point of this work, and a very exciting new observation; however, little is gained by systematically downplaying the utility of past work. The existing body of literature has led to the prevailing

hypothesis that organic compounds facilitate particle growth to CCN sizes in the Arctic (e.g., Abbatt et al., 2019; Burkart et al., 2017; Willis et al., 2016, Willis et al., 2017, Croft et al., 2019:) and that these organic precursors are likely sea-surface microlayer derived aldehydes (e.g., Schneider et al., 2019, 2024; Kilgour et al., 2024: 2024; Mungall et al., 2017). The current work provides more definitive confirmation of these processes by supplying gas-phase OOM observations together with molecular cluster composition, which is an important step beyond existing work. At Lines 1139-1142 and 1160-1161 the authors state that NETCARE observations "did not capture nucleation," however, Willis et al., 2016 observe nucleation-mode particles between 5-20 nm (the author's own criteria for directly linking observed particles to nucleation; statement on Line 1146) during a particle growth event where organic compounds were linked to growth once particles entered the size range measurable by an HR-AMS (>70 nm, as is also done in this work). Notably, the authors do not cite Croft et al., 2019 (https://doi.org/10.5194/acp-19-2787-2019) or Willis et al., 2017 (doi: 10.1002/2017GL073359). Croft et al., 2019 shows systematic evidence based on and aerosol microphysical model for the importance of organic compounds for explaining observed Arctic aerosol size distributions, and Willis et al., 2017 demonstrates that secondary organic aerosol contributes to growth of particles to CCN sizes (again, >70 nm, based on HR-AMS measurements) in the Arctic marine boundary layer. The novelty of this manuscript is undoubtedly that it identifies molecular information about low-volatility OOMs that contribute to particle growth, and that conclusion is only supported – not diminished – by the existing body of literature in this area. The manuscript should be significantly revised to reflect this fact.

Response: We very much agree with the framing. The prevailing hypothesis, as put by the reviewers, is that organic compounds facilitate particle growth to CCN sizes in the Arctic (e.g., Abbatt et al., 2019; Burkart et al., 2017; Willis et al., 2016, Willis et al., 2017, Croft et al., 2019) [6-8, 10, 51] and that these organic precursors are likely sea-surface microlayer derived aldehydes (e.g., Schneider et al., 2019, 2024; Kilgour et al., 2024: 2024; Mungall et al., 2017) [11-13]. Accordingly, we have thoroughly rewritten Section S7 (now section S1).

We have revised the text to clarify that, in this context, "nucleation" refers to the formation of new particles from gas-phase precursors, and that the absence of sub-15 nm measurements in the previous study precluded direct attribution of the observed particles to local nucleation.

Please see response to comment 30 by Reviewer 1&2, as we have fully rewritten the relevant SI text section to improve our discussion of this.

We also revised line 283-286 in original manuscript**:**

"Our findings provide the first direct ambient observational evidence that organic compounds—including previously unrecognized classes such as OOMs derived from aldehydes and iodine-containing OOMs—contribute to NPF in the Arctic and drive their growth to CCN (see Supplementary Text 1 for further discussion)**"**

to **(line 113-115)**

"By directly linking emissions from the marginal sea ice zone to a substantial increase in CCN, our findings provide a complete observational closure from precursors to cloud- and climate-relevant particles."

Comment 9: Larger J_1.7 than expected, based on CLOUD observations, at temperatures > 0°C for the HIOx-H_2SO_4 system. The authors infer that dimethylamine drives more rapid H_2SO_4-HIO_3/HIO_2 nucleation than observed at warm temperatures in CLOUD chamber experiments. But a major piece of evidence, beyond direct detection of HIOx-H_2SO_4 molecular clusters, appears to be that the HIOx-H_2SO_4 system most closely reproduces J_1.7 values. Lines 455-457 describe that the NAIS data is used in the conjoined PNSD for 3-25 nm and J_1.7 is calculated from J_4 in the NAIS data (Lines 759-760); however, the methods for accomplishing conjoined PNSD are not described. In particular, NAIS instruments typically over estimate nucleation mode particle concentrations compared to other instruments (e.g., Kangasluoma et al. 2020: 10.1016/j.jaerosci.2020.105584, Manninen et al. 2009: https://www.borenv.net/BER/archive/pdfs/ber14/ber14-591.pdf) – was the NAIS data scaled to match the nano-MPSS in their quite wide overlapping size range (4.5 – 42 nm)? Could overcounting in the NAIS explain higher than expected J_1.7 for the HIOx-H_2SO_4 system. A related conclusion is that synergistic effects of DMA are responsible for the higher than expected nucleation, but this conclusion is completely missing from Figure 3. Overall, the conclusion that DMA explains the agreement with low temperature multi-component nucleation via (H_2SO_4+HIO_3)*HIO_2 when ambient temperatures averaged 1.4°C needs further evidence.

Response: Thank you for the comment! This is indeed missing from our methods. We are aware about the over-counting of the NAIS. Our steps were as follows

1) Plot the size distributions of the Nano-SMPS, NAIS, and Long-SMPS
2) For the regions where there are overlap, calculate the ratio between NanoSMPS and NAIS, and LongSMPS and NAIS
3) Correct the NAIS and NanoSMPS to the LongSMPS data in the regions there are overlap (in this case, we multiplied the NAIS and NanoSMPS down).
4) Plot the individual size distributions to ensure the instruments match one-another for the entire measurement period

We did consider adding a "DMA" term to Figure 3b, but eventually decided that a more direct comparison with the CLOUD chamber results was more valuable. We now, after prompting from reviewer 1, include the hypothesis that OOMs, including I-OOMs, could also drive this enhancement. The section now reads as follows (line 227-247):

"Considering the average ambient temperature of 1.4 ℃ during nucleation periods, the observed $J_{1.7}$ values are higher than anticipated from the temperature dependence measured in the CLOUD chamber [17] (at -10 and +10 ℃). This enhancement strongly suggests the participation of other stabilising species. One plausible contributor is DMA, as evidenced by the detection of DMA clustered with HIO_x (Fig. 2 and Figs. S5-6). The DMA-HIO_2-HIO_3 cluster signal correlates with HIO_3 concentrations, and is highest when HIO_3 is at its maximum (Figs. S9-10). The role of DMA in enhancing nucleation rate is corroborated by quantum chemical modelling studies showing that DMA can accept a proton from HIO_3 as a base, forming a stable cluster (DMA-HIO_2-HIO_3) [37].

Furthermore, given the high abundance of OOMs, particularly ELVOCs (Fig. 1C), it is also plausible that they contribute to the enhancement of nucleation rate, either by participating directly in nucleation or by stabilising the initial inorganic clusters. This would be consistent with studies

from other environments showing OOMs can enhance nucleation rates [38]. Furthermore, as the volatility-reducing effect of iodine in our observed I-OOMs is not fully constrained, it is possible that some of the observed I-OOMs are ULVOCs, which may participate in the initial nucleation.

Our results demonstrate that H_2SO_4, HIO_3, and HIO_2, are the core components of a synergistic nucleation mechanism in our study area and this process appears to be potentially enhanced by the presence of bases such as DMA, and by OOMs. This provides real atmospheric validation of the multicomponent nucleation mechanism identified by He et al. [17], at lower vapour concentrations than measured in CLOUD (Fig. 3), and further suggests that in complex ambient environments, this process is likely modulated by both amines and organic vapours. The precise contribution of each warrants further investigation."

Comment 10: Quantification of aldehyde and monoterpene oxidation products with the PTR-MS. Information on how the calibrations for the PTR-MS were applied to non-directly calibrated species is missing (Lines 607-615). At lines 260-261 the authors state "The total concentration of C_{7-12} aldehydes and their fragments reached a peak of 420 pptv during this NPF event while the monoterpene signal is ~3 pptv" However, by summing all peaks associated with aldehydes and their fragments (Lines 981-984) that authors are likely overestimating the total aldehyde concentration. Unless potentially there is some quantification using the GC-MS data, but this should be clearly described beyond the Extended Data figure.

Response: VOC missing ratios were determined using a transmission curve based on that described by Taipale et al. [52], as implemented in the Ionicon PTRViewer software. For monoterpenes and other stable compounds, it was quantified using the MH^+ ion. For aldehydes, which undergo fragmentation [13], we sum all of their fragments, determined as the known fragments with high correlation coefficients with the parent ion. This is necessary, as the fragments often exceed the signal of the parent ion itself [13]. We acknowledge that co-fragmentation of interfering species to the same mass risks overestimating the total mixing ratio. However, given the strong correlation of these fragments with $C_9H_{18}OH^+$ (Extended Data Fig. 10, now Fig. S15) and the low concentration of monoterpenes (the most likely alternative parent ion), we argue that the signal is dominated by aldehyde fragmentation. While we could not validate this exactly via 2D-GC-ToF-MS due to a lack of calibration for these species, we agree that these values should be treated as an upper limit. We have therefore updated the text to explicitly state that this represents an upper bound of the total concentration (line 316-323):

"The total concentration of C_{7-12} aldehydes and their fragments reached a peak of 420 pptv during this NPF event while the monoterpene signal is ~3 pptv, **although these fragments may have non-aldehyde sources, and this is therefore an upper-limit estimate of the total concentration**."

Comment 11: Data treatment and statistics for observations on NPF and non-NPF days. Figure 2 is notably unhelpful to the reader since the authors are able to describe which clusters are present during NPF, Burst and Pre-NPF in only a couple sentences. A more quantitative approach of moving the boxplots in Extended Data Fig. 2 into the main text, and moving Figure 2 to Extended Data, would make the point more apparent and provide more statistically useful information (if statistically significant differences in observations were noted). The authors occasionally use means (averages) in their main text figures, but the means are not displayed in the relevant boxplots in the supplement.

i.e., Fig. 1C and Extended Data Fig. 2. Related to this, the median condensation sink was on ~40% lower during NPF compared to Burst days (Lines 136-137). Is that not substantial? Are the differences statistically significant?

Response: We agree that a more quantitative statistical comparison is valuable for the reader. We argue that the mass defect plot (Figure 2) offers a unique and complementary perspective that is crucial for the main text. While the boxplots show the *total* concentrations, the mass defect plot provides an valuable visual overview of the entire measured chemical system. It allows the reader to see not only the presence of the specific nucleating clusters, but also the nature, diversity, and relative abundance of the different OOM families (CHO, CHON, and the novel I-OOMs) that are present. This qualitative "fingerprint" of the chemical environment during each event type is a key piece of evidence that may be lost in a simplified statistical summary.

To address these points, we propose a solution that we hope incorporates the best of both approaches. We will add the mean values to our boxplots to enhance consistency with the main text figures. Regarding the condensation sink, there is indeed a difference, it is, however, in all cases, small, so relative differences mean slightly less. We however now acknowledge this in the manuscript (line 158-161):

“The condensation sink, **the rate at which low-volatility vapours like sulfuric acid are lost to pre-existing particles, was 40% lower on NPF event days as compared to non-event days, and highest on burst events (**$7{\cdot}10^{-4}$ s^{-1}**,** $5{\cdot}10^{-4}$ s^{-1}**, and** 10^{-3} s^{-1} **respectively)** (Fig. S4 and Table S1).”

For the reviewer's convenience, we have reproduced the boxplot figure below with the mean values added, and we will incorporate this improved figure into the revised manuscript.

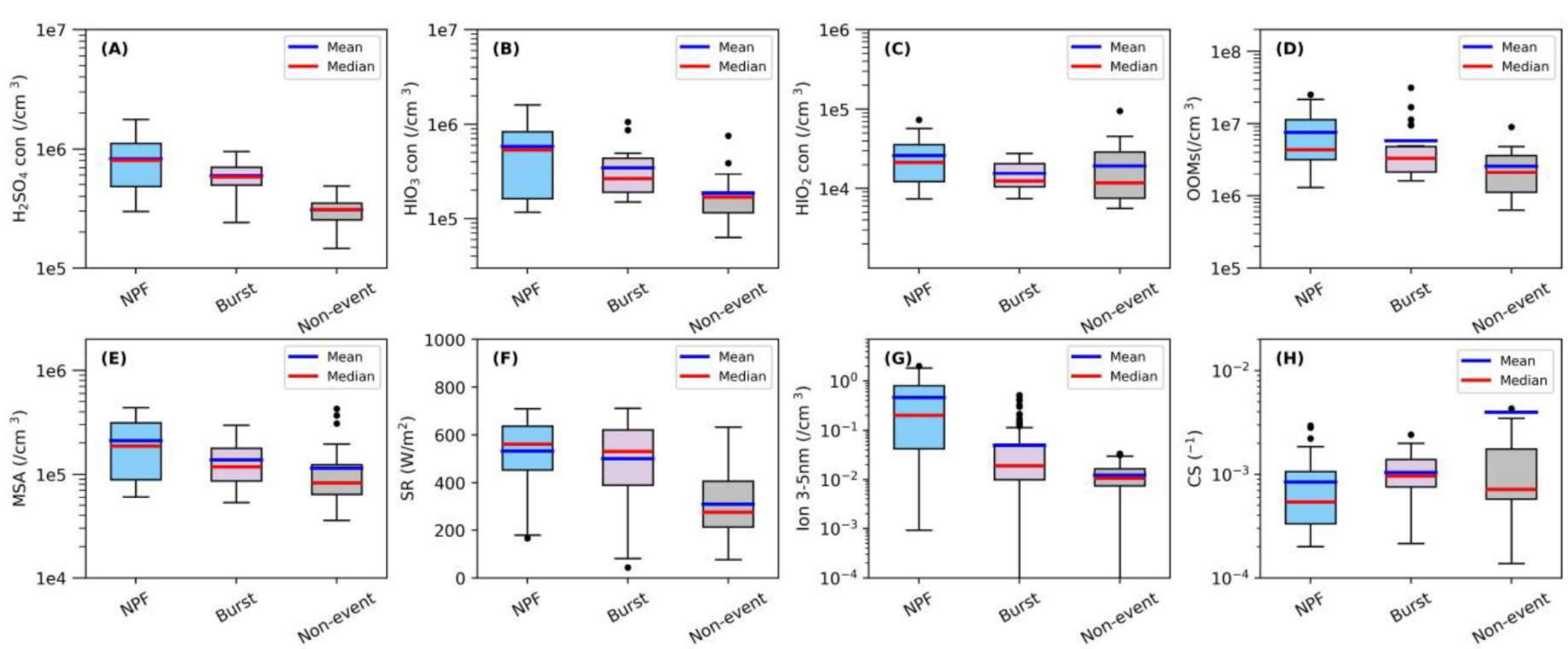

“Figure S4: Box plot for NPF, burst, and non-event events: (A) H2SO4 concentration, (B) HIO3 concentration, (C) HIO2 concentration, (D) OOMs concentration, (E) MSA concentration, (F) Solar Radiation, (G) 3-5 nm ion number concentration, and (H) Condensation sink. In each plot, the red line indicates the median value **and the blue line indicates the mean value**, while the box represents the interquartile range (25th to 75th percentiles). The whiskers extend to data points within 1.5 times the interquartile range, and black dots show outliers beyond this range. Data span from 10 am to 3 pm for each event type. Note: the days dominated by ship emissions, denoted by grey boxes in Fig. S2, were filtered out.”

Specific Comments:

Comment 12: Line 51-53: The authors do not use their observations to establish a negative climate feedback, to quantify the cloud radiative effect due to specific nucleation mechanisms, or show effects on the hydrological cycle. Therefore, these speculative statements do not belong in the abstract.

Response: Please see response to comment 1 and we have removed the statement.

Comment 13: Line 55: "has increased by nearly four times faster", 'by' should be omitted.

Response: This has been amended, and we opt with the following (more elegant) phrasing (line 54-55):

"Over the last four decades, Arctic surface air temperature has increased at more than thrice the global average"

Comment 14: Line 62: Remove cloud from this sentence, or clarify that NPF mechanisms and aerosol processes in general are not the sole reason that model struggle to reproduce cloud.

Response: We make this clearer as follows (line 60-62):

"However, climate models consistently struggle to accurately reproduce observations of Arctic aerosols and CCN, largely ascribed to a limited understanding of precursor sources and NPF mechanisms [8,5,9], limiting their ability to model clouds."

Comment 15: Lines 98-100: This is the first mention of dimethylsulfide but it is mentioned as if the reader should be familiar with it.

Response: We have amended this as follows (line 104-107):

"All this is important for predicting changing Arctic climate into the future, where, for example, emissions of gas-phase iodine and dimethylsulfide (DMS), **the major marine biogenic sulfur compound for aerosol formation**, are increasing [12,26-29]."

Comment 16: Lines 102-103: Is this an average sea ice extent for June? This period of time corresponds to post- ice-melt onset for Baffin Bay. How much did the MIZ and ice concentrations change over the 24 days of measurements?

Response: Yes, this is the average sea ice extent for the month of June. The difference was relatively small (see Fig S7).

Comment 17: L 105: For Fig 1B, 1C are diel mean values of the PNSD the best metric to use? Should this PNSD be normalized before grouping and taking the mean to avoid over-weighting days with high number concentrations? (i.e., is the NPF PNSD mainly representing the June 7-8 case?)

Response: We did consider normalising the data. However, we concluded that this would introduce an alternative bias by giving undue weight to weaker, low-concentration events. This would artificially lower the average concentrations and misrepresent the typical conditions under which significant new particle formation and growth occur. We opt instead to just show the mean of the natural environment.

Comment 18: Lines 142-143: If this cluster is related to an instrument artifact why does is only show up in the NPF set?

Response: We should be clearer. We mean that the NO_3^- comes from the mass spectrometer, so this is a cluster of iodine oxoacids plus an NO_3^- from the CIMS inlet, we amended this (line 166-167):

"The NO_3^- in HIO_2-HIO_3-NO_3^- is a charger ion introduced by the mass spectrometer [17], and so this ion represents a cluster of iodine oxoacids"

Comment 19: Lines 127-129: How are days are attributed to NPF when they have unquantifiable growth?

Response: For the days in question, particle growth was clearly observable in the particle number size distribution data, as shown in Fig. S2. However, a reliable (GR) calculation requires the air masses remain stable and NPF occurs over large areas. These specific measurements were taken while the ship was inside a fjord, where observed large variability in the PNSD, which prevented a robust GR calculation. As the key criteria for an NPF event (the initial formation of new particles followed by observable growth) were met, we classified them as such, despite the inability to quantify the rate, although for a while we did call these "Fjord days".

Comment 20: Lines: 167-168: These are very difficult to distinguish from the rest of the data due to the red line filling in part of the open circle

Response: We modified the red line to cyan to improve the visual distinction of data points (line 192).

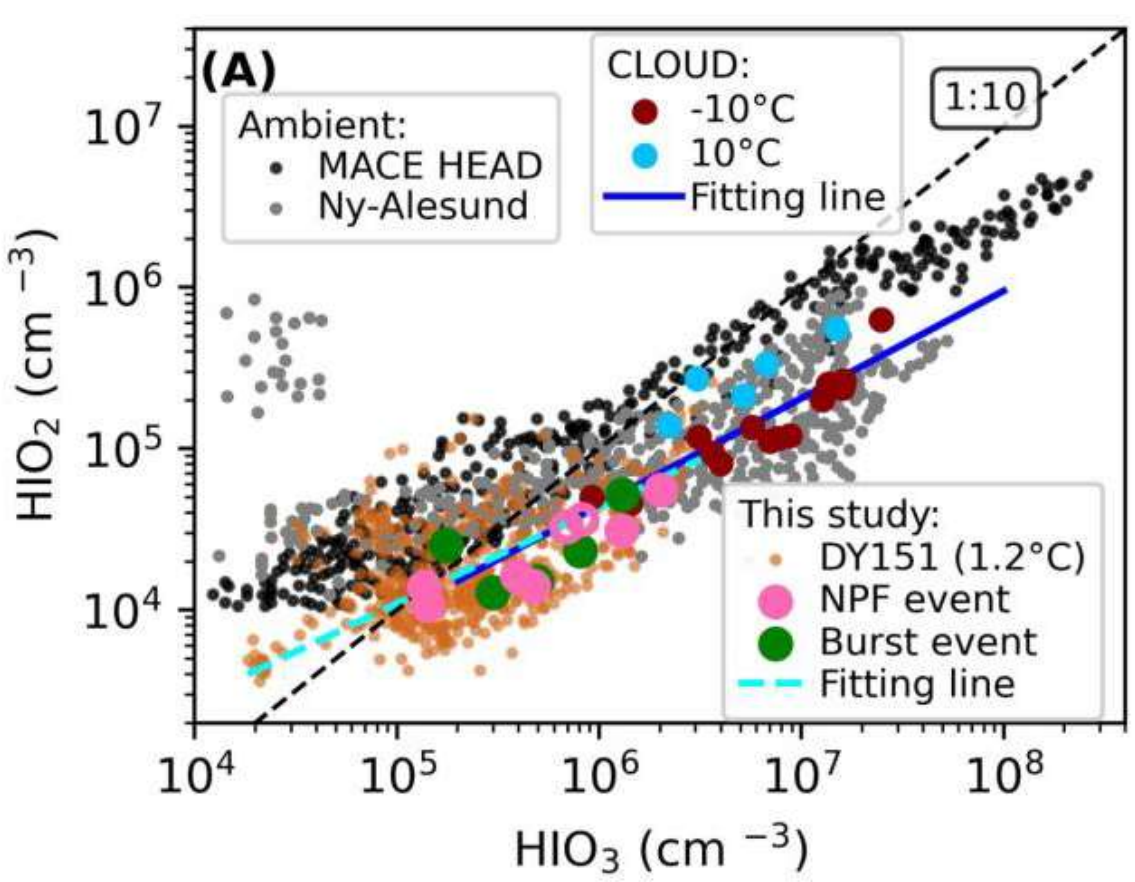

Comment 21: Lines 170-171: "Middle between those regressions" does not have any statistical meaning. Is that mean/median? And between "those" regression lines? Which regression lines are the authors referring to? The difference between the filled and unfilled shapes in B also needs to be explained or clarified in the legend

Response: In an ideal world, we have data at zero degrees. Given that the two regression lines for the 10 and -10 °C are parallel, if we assume that there are linear relationships, then the line in the middle of the two regression lines would be the mean values and represents what would be anticipated of 0°C. We recognize that this is based on a linear relationship and thus we acknowledge this assumption in the revisions.

We have now explained the filled and unfilled shapes in B. And the new section now reads (line 199-201):

"Dashed line in (B) is parallel to and has the same distance to the regression lines of the $J_{1.7}$ and $(HIO_3 + H_2SO_4) \times HIO_2$ at the temperature of 10 and -10°C. Assuming a linear relationship, this line would be the mean values and represents what would be anticipated of 0°C."

Comment 22: Lines 171-172: All data points or only the pink and green?

Response: Amended!

Comment 23: Lines 172-173: Some of these points have estimated HIO_2 values based on extrapolating the HIO_3 vs. HIO_2 relation down when HIO_2 was below detection limit. These points should be marked somehow.

Response: We agree and amended this, using hollow points. Note that the data points in Fig. 3a are more difficult to distinguish due to the high density of data and their smaller size. In the new figure, we changed the line to a dashed style to indicate the data points on the 23rd and 24th of May.

Comment 24: Lines 142-143: Is HIO_3-NO_3^- indicating something atmospherically relevant or not?

Response: It is HIO_2-HIO_3-NO_3^-, not HIO_3-NO_3^-. This is therefore a cluster of iodine oxoacids. We state the following in the manuscript (line 166-167)

'The NO_3^- in HIO_2-HIO_3-NO_3^- is a charged charger ion introduced by the mass spectrometer, and so this ion represents a cluster of iodine oxoacids."

Comment 25: Line 146: What is 'variability' here referring to?

Response: The section that stated "variability" now says the following (line 170-176):

"A day-by-day analysis (Figs. S5-6). reveals that while the detectability of specific clusters varies with precursor concentrations and analytical interferences, the synergistic iodine-sulfur mechanism is a persistent feature. On nearly every NPF day, we observe a clear signal for at least one key cluster containing both HIOx and sulfuric acid (e.g., the $HIO_3 \cdot HSO_4^-$ dimer or the $HIO_2 \cdot H_2SO_4 \cdot HSO_4^-$ trimer), confirming the consistent co-involvement of both precursor systems. For example, during the event on 7th June, we identified the key multi-component clusters HIO_2-HIO_3-NO_3^- and HIO_2-H_2SO_4-HSO_4^-."

Comment 26: Lines 216-218: The appears as confusing logic. We should not expect IOOMs to correlate with sea salt even if IOOMs were primary in origin unless it was by the same primary process producing I-OOMs and sea spray. While IOOMs are likely secondary in origin, but the best evidence for that is the correlation with HIO_3 not a lack of correlation with an unrelated process.

Response: We agree with this argument and amend as follows (line 262-266):

"A separate PMF analysis of our Aerosol Mass Spectrometer (AMS) data further supports the role of I-OOMs in growth of new particles. We identified an iodine organic aerosol factor, which contains the CH_2I^+ fragment (Methods 12) and is likely due to I-OOM condensation. This factor

correlates poorly with sea salt (Fig. S16) but its concentration increased as particles grew larger (Figs. 4F and S16), further confirming the contribution of I-OOMs to particle growth."

(line 334-336):

"The iodine organic aerosol factor **in the AMS** increased as particles grew larger (Figs. 4F and S16), further confirming the contribution of I-OOMs to particle growth"

Comment 27: Lines 272-276: Are the authors claiming here that lower volatility organics contribute more to growth as particles increase with size? This would be counter to microphysical expectations (Semi- volatile organics can overcome the Kelvin barrier as particle size increases, see Burkart (Ref 41)).

Response: Our mistake! We should have been clearer. This now reads as follows (line 332-334)

"…which we confirm with chemical observations that the growth is driven by the greater role of LVOC as opposed to ELVOC as the particle size increases (Fig. 4D)."

Comment 28: Line 273: "at the Canadian Arctic" Be more specific. Your measurements are also in the Canadian Arctic and sub-Arctic (Labrador Sea, Davis Strait, Baffin Bay).

Response: We have revised the manuscript to state the specific location of the Burkart et al. (2017) study (the Canadian Arctic Archipelago)

Comment 29: Line 286-288: Of course this can be true, but what evidence do you have that your non-NPF day CCN concentrations were impacted by NPF processes that you did not directly measure?

Response: This is a fair point. We have no evidence for or against this fact, but as NPF seems to be a major CCN source, it's certainly a possibility. We make the following amendment to acknowledge this fact (line 345-348):

"Note that even when no CCN enhancement was directly observed following nucleation events, newly formed particles may still contribute to the CCN budget downwind as they grow to larger diameters [24]. Similarly, some of our observed background CCN concentrations may themselves come from NPF processes based on data from previous studies [11,48]."

Comment 30: Line 301: More descriptive section headings would be informative.

Response: We amended this to "Sources and processes influencing high latitude new particle and CCN formation"

Comment 31: Line 333: Direct photochemistry of fatty acids in laboratory studies (i.e., the authors Ref 37) is likely a result of contaminants, see DOI: 10.1126/sciadv.adj6438 , while indirect photochemistry and multiphase reaction of organics in the sea surface microlayer with atmospheric ozone remain viable possibilities.

Response: Very interesting point, thank you. We amend our reference list appropriately by removing this reference.

Comment 32: Line 326: "they" here appears to refer to either ice algae or DMS. Should be reworded.

Response: We intended DMS, this has been amended

Comment 33: Line 330: Ocean waters have consistently been shown to be a sink of NH_3 in Arctic regions (e.g., Wentworth et al., https://doi.org/10.5194/acp-16-1937-2016). Tundra soils may also be an important source of NH_3, see Murphy et al., https://doi.org/10.1039/D4FD00173G

Response: Thank you, we now referred to these papers.

Comment 34: Lines 338-340: Is this true? Monoterpenes have yields ranging from <0.1% (b-pinene) to 17% (limonene) (Table 3. Bianchi). Some aldehydes have yields up to 3% (Fig. 3, Wang) which isn't 'much lower' especially within uncertainties associated with many of these measurements.

Response: Yes, we appreciate the complexity. We amend to the following to be appropriately cautious (line 398-399).

"Both aldehydes and monoterpenes readily undergo autooxidation to form OOMs [39,55]."

We provide additional discussion of this in response to your Comment 42.

Comment 35: Lines 344-345: The authors now describe NH_3 as abundant in the Arctic, whereas earlier in the manuscript the authors describe that large regions of the Arctic have very low NH_3 concentrations, citing Croft et al. The authors appear to be invoking different interpretations of existing observations and model predictions as is convenient for their arguments. The logic here needs to be refined, and should be less speculative.

Response: Yes, we need to be clearer. We meant to say that NH_3 is abundant in parts of the Arctic, as suggested in Croft et al. We rewrote this to reflect this point (line 400-408):

"HIO_x and H_2SO_4 are likely ubiquitous across the marine Arctic [8,13,14], and we argue that the synergistic nucleation process we observed is therefore likely ubiquitous across high latitude marine environments. The importance of our observed NPF processes is likely to manifest throughout polar regions where the ice is melting in larger areas, including in the Antarctic [55]. Nucleation rates can be enhanced by NH_3, which is abundant (>100 pptv) in coastal regions of the Arctic with bird nests, but predicted to be almost absent in the ice-covered high-latitude areas (<1 pptv) and central ocean regions [33,53]. Amines (as detected in the DMA-containing cluster in Figs. S9-10) and OOMs can further enhance nucleation rates."

Comment 36: Lines 351-353: How are the authors defining "lower" and "higher" Arctic regions here? What evidence is available on OOM concentrations in "high" Arctic regions?

Response: By "higher Arctic," we are referring to the central Arctic Ocean and pack ice region. Our statement that OOM concentrations are likely low in this region is an inference based on the findings of Baccarini et al. (2020). In their study over the pack ice, they observed that HIO_3 alone was sufficient to explain particle growth, which strongly implies that the contribution from other condensing vapours, such as OOMs, was minimal. By "lower Arctic," we are referring to the sub-Arctic seas and the marginal ice zones, such as our study area. In this environment, our measurements clearly show that OOMs are abundant and are, in fact, the primary drivers of particle

growth. We have revised the manuscript to use clearer terms with these more precise geographical descriptions and to explicitly state the evidence supporting this contrast. Line 413-417:

"Similarly, OOM concentrations are likely low in the ice-covered higher Arctic, for instance, in the central Arctic pack ice region, leading to limited new particle growth and CCN formation [7,10,13,14], while OOMs seem to be more abundant in the Arctic and sub-Arctic seas and marginal ice zone, resulting in faster growth of new particles (Fig. 4 and Fig. S17) and contributing to more CCN formation (Fig. 5)."

Comment 37: Lines 487: remove underscore

Response: Amended

Comment 38: Lines 557-559: repeated sentences.

Response: Amended

Comment 39: Line 823: "The number of factors is determined based on the results." What does this mean?

Response: This now says "the *interpretability* of the results"

Comment 40: Lines 981-984: The nuances of the aldehyde measurements and quantification are not adequately reflected in the main text, which wholly glosses over this.

Response: We now include the following (line 312-317)

"Thus, it is likely that most $C_5H_8H^+$ signal is from aldehyde fragmentation [42-44], as shown in previous laboratory observations [45]. **However, the fragmentation patterns of individual aldehydes are complex, and deconvolving their individual concentration from a mixed atmospheric sample is not possible with our data. For this reason, we report the summed concentration of aldehyde-related signals.** The total concentration of C_{7-12} aldehydes and their fragments reached a peak of 420 pptv during this NPF event while the monoterpene signal is ~3 pptv…"

Comment 41: Line 999: The authors do well to not interpret the PTR-MS $C_5H_9^+$ signal, but how much isoprene was measured in the TD-GD-MS analysis?

Response: Unfortunately for us the tubes and traps are only designed to collect organic compounds from about C_8 upwards, so we don't have any quantification of isoprene in our 2D-GC-ToF-MS

Comment 42: Lines 1044-1045: This sentence needs multiple citations. The OH statement may be true for small saturated aldehydes but reactions of OH with unsaturated aldehydes can be significantly faster than with MT (i.e., butenal compared to a-pinene is ~3x as fast via OH (MCM)). Citation needed for the autoxidation statement.

Response: Very true. We have included citations to both the OOM (HOM) research as well as chemical kinetics literature, while also tempering our statements as follows (line 1226-1232):

"Furthermore, monoterpenes and aldehydes have different reactivities towards OH and O_3, as well as different OOMs yields. **Many of the most common** monoterpenes are highly reactive [119] and

readily undergo autoxidation [39]. Aldehydes are **generally** less reactive with OH and much less reactive with O_3 [127,128], and **likely** undergo less efficient autoxidation [54]. OOM yields of aldehydes are therefore much lower, depending on temperature and oxidants [54]. This likely explains the similar concentrations of the factors, despite different concentrations of precursors."

We have edited this statement, as rightly pointed out we do not know what mixture of monoterpenes are present, and therefore the yields could be anything from <1% to 19%.

Comment 43: Lines 1140: "hypothresized"

Response: This was fixed

Comment 44: Extended Data Fig. 2: Describe the anatomy of the boxplots (i.e.; 1:25:50:75:99 percentiles). Additionally, in the main text you are using the mean values from each period. This mean value should be displayed on the boxplot as well.

Response: This is now in the figure caption, and this figure now contains both the median and mean.

Comment 45: Extended Data Fig. 7: What took place on 2022-5-30 that caused the over prediction in growth rate?

Response: Great question. We do not have definitive answers to this question as we generally expect underprediction, however, other factors can also affect this modelling. Overprediction does happen, as shown in previous studies [53-55]. We also discuss this in detail in the section "*Uncertainties of particle growth modelling*"

To address this, we added this (line 291-293):

"On one occasion (30th May), the model overpredicted growth, likely due to meteorological factors not captured by the model (see Supplementary Text 5)."

Comment 46: Extended Data Fig. 10: Describe the coloring in the timeseries again (Extended Data Fig. 10a) and resize the labels to make them readable (Extended Data Fig. 10b)

Response: amended

Comment 47 Fig. S3: The reported $J_{1.7}$ should also be shown in this figure here. The data should probably be selected to at least be the same time of year.

Response: The data in this figure are now for the months of May and June.

Comment 48: Table S1: Absolute number concentration is needed here.

Response: We are unsure whether this refers total particle number concentration. The time-series data of particle number size distribution and the number concentration of ions in the 3–5 nm range, is presented in Fig. S2A.

Reviewer 5

General comment: Thank you for the opportunity to review this manuscript. Du et al. present in-situ ship-based observations of new particle formation (NPF) events in the Arctic marine boundary layer, where synergistic nucleation of iodic and sulfuric acids is followed by organic-driven growth, in some cases leading to cloud condensation nuclei (CCN). The authors further analyze the composition of oxygenated organic molecules (OOMs) and incorporate them into a growth model, showing that condensation of monoterpene, aldehyde, and iodine-containing oxidation products dominates particle growth. The study highlights the marginal sea ice zone as a hotspot of gaseous precursors, with potential implications for cloud cover, radiation balance, and the Arctic hydrological cycle during summer. The manuscript is clearly written and represents one of the most comprehensive characterizations of Arctic NPF to date. I commend the authors for the quality and quantity of their work. Nonetheless, I have several concerns that currently preclude recommending publication in Nature, although I leave the decision entirely to the editor.

General Response: We thank the reviewers for their thorough feedback on our manuscript. We understand that the main reservations about the novelty and originality of the findings from our study and we have thoroughly explained what we know, what we do not know, what is new in our study, and why it is novel and important (see General Response and Response to Reviewer 5 comment 1). Below, we address the comments point-by-point.

Comment 1: Novelty of the findings. My primary concern relates to the novelty of the results. The manuscript highlights synergistic nucleation of iodic and sulfuric acids, with stabilization by dimethylamine, and subsequent growth dominated by OOMs. While these are important findings, they appear largely incremental when compared with the current state of knowledge. Previous field studies have already demonstrated multiple nucleation pathways in the Arctic. For example, Beck et al. (2019) reported NPF driven by H_2SO_4 and NH_3 at Ny-Ålesund, while HIO_3 dominated at Villum during spring, and H_2SO_4–NH_3 during summer. Baccarini et al. (2020) observed NPF over pack ice exclusively driven by HIO_3. Thus, multiple nucleation precursors have already been documented in different seasons and regions. Even if prior studies did not explicitly report multi-component nucleation in the Arctic, CLOUD laboratory experiments have demonstrated the HIO_3-H_2SO_4 nucleation including the report of HIO_3 and H_2SO_4 concentrations at many relevant locations from field measurements around the globe. The stabilization of nucleating HIO_3-H_2SO_4 clusters by amines is novel, but the role of amines is conceptually consistent with urban field measurements and laboratory evidence from CLOUD and does not constitute the discovery of an overlooked mechanism.

Regarding growth, several studies have already suggested a role of organics. Beck et al. (2019) inferred organic contributions to growth at Svalbard, Willis et al. (2016) hypothesized that biogenic OAs likely contributed to CCN formation in the Canadian Arctic, and Burkart et al. (2017) provided modeling-based evidence for organic-driven growth, albeit without direct molecular confirmation. Baccarini et al. (2020) also excluded sulfuric acid as a significant contributor to growth, indirectly pointing to organics. What Du et al. add here is valuable molecular-level identification of condensing vapors (monoterpenes, aldehydes, and iodine-containing products), but this conclusion is limited by the exclusive reliance on nitrate-CIMS (biased toward highly oxygenated species) and the absence of

particle-phase molecular composition measurements, apart from AMS, which cannot resolve the critical sub-CCN size range and is prone to extensive fragmentation.

Finally, the observation of two events where nucleated particles grew to CCN sizes is noteworthy, yet previous studies already inferred such links (e.g., Beck et al., 2019; Willis et al., 2016). What remains missing here is a quantification of the contribution of this process to the overall CCN budget and, ultimately, to cloud properties and radiative forcing. The connection between natural marine emissions, their enhancement with ice melt, and their potential feedback on climate through CCN formation is not new. This idea dates to the CLAW hypothesis in the 1980s and has since been revisited and modeled repeatedly in the context of marine biogenic sulfur emissions and, more recently, iodine- and organic-driven NPF. While the current study provides valuable in-situ evidence for nucleation and growth fueled by Arctic natural emissions, the authors do not translate their measurements into implications for CCN budgets, cloud cover, or radiative forcing, nor do they assess whether H_2SO_4–HIO_3 nucleation could offset even a fraction of the warming from greenhouse gases. This gap likely reflects the limited number of events observed, but it does mean that the climatic relevance of the findings remains speculative.

In summary, while the study delivers important empirical evidence, it does not uncover a fundamentally new nucleation or growth mechanism, nor does it provide a quantitative assessment of their impact on CCN, clouds, or climate. The findings are therefore best described as incremental, rather than transformative, in the context of Nature.

Response: We appreciate the perspective that our findings build upon an extensive and important body of existing work. We agree that previous studies have laid important groundwork by identifying different, individual (but not synergistic) nucleation pathways (e.g., H_2SO_4-NH_3 or HIO_3) and by providing inferential evidence for the role of organics in particle growth. Here we provide detailed responses to questions regarding the novelty of our findings.

(1) Novelty - nucleation

We thank the reviewer for the detailed comparison with previous Arctic new particle formation (NPF) studies. We agree that earlier Arctic field studies have reported seasonal or regional dominance of individual, predominantly two-component nucleation pathways (e.g., H_2SO_4-NH_3 or HIO_3).

The advance of the present study lies in demonstrating **multicomponent nucleation (involving more than two components) under ambient Arctic conditions**, in which iodic acid, sulfuric acid, and stabilising species (including HIO_2 and likely amines-– see also Respond to Comment 2 by Reviewer 1&2) act **simultaneously within the same aerosol population**, rather than as alternately dominant pathways (Beck et al., 2021; Baccarini et al., 2021). While previous Arctic studies documented individual mechanisms under specific conditions, they did not establish the multiple component nucleation.

In the broader context of NPF research, it has been common for mechanisms first identified in chamber experiments to be subsequently evaluated through **campaign-scale, process-oriented atmospheric observations** in order to establish their relevance under ambient conditions. This approach has been adopted in influential studies of amine-enhanced nucleation (Almeida et al., Nature, 2013; doi: 10.1038/nature12663; Yao et al., Science, 2018; DOI: 10.1126/science.aao4839;

Brean et al., Nature Geoscience, 2021, doi: 10.1038/s41561-021-00751-y) and isoprene-derived organonitrate nucleation (Curtius et al., Nature, 2024, doi: 10.1038/s41586-024-08192-4 ; Shen et al., Nature, 2024, doi: 10.1038/s41586-024-08196-0), which did not aim to quantify global prevalence or climatic forcing, but nevertheless represented important and non-incremental advances by demonstrating that proposed mechanisms operate in the real atmosphere.

As demonstrated by prior work, mechanistic feasibility in chamber studies does not necessarily translate to atmospheric relevance: for example, there is no conclusive evidence that multicomponent H_2SO_4–HNO_3–NH_3 nucleation observed in laboratory experiments (Wang et al., Nature, 2020, doi: 10.1038/s41586-020-2270-4; Wang et al., Nature, 2022, doi: 10.1038/s41586-022-04605-4) was confirmed in the real atmosphere.

Within this context, although iodine oxyacids have been shown to be highly efficient nucleating agents in laboratory studies (He et al., 2023), it has remained unclear whether iodine–sulfur multicomponent nucleation operates under chemically complex Arctic conditions, where amines, ammonium, and organic vapours coexist and often at substantially lower (and variable) concentrations. The present study provides **process-level observational evidence** addressing this question under ambient Arctic conditions.

Accordingly, the contribution of this work is framed in terms of establishing atmospheric operability of a multicomponent nucleation pathway, rather than assessing its regional dominance or climatic impact, which would require broader observational coverage and modelling beyond the scope of a process-focused field investigation.

(2) Novelty – growth

We agree with the reviewer that previous Arctic studies have **hypothesised or inferred** an organic contribution to particle growth.

The contribution of the present work lies instead in **resolving this hypothesis at the molecular level under ambient Arctic conditions**, by directly identifying the low-volatility vapours associated with particle growth (as emphasized by Reviewer 3&4, Comment 8). Earlier studies relied on particle growth rates, modelling constraints, or bulk chemistry observations, whereas molecular identification of the condensing vapours had not previously been available.

We acknowledge the reviewer's point regarding instrumental selectivity and we have clarified these limitations in the revised manuscript. These considerations do not alter the originality of providing the first in situ molecular constraints on growth-driving vapours in the Arctic atmosphere, or the conclusion of our study.

3) From cluster formation to new particles and to CCN in a same aerosol population

As noted by the reviewer, new particle formation **has long been inferred or hypothesised** to contribute to CCN in the Arctic. However, previous studies have relied on indirect evidence for example, based on size-based thresholds (e.g., 30 nm) or cloud residues measurement, rather than following the same particle population from molecular cluster formation through growth to directly measured CCN.

Here, we provide direct in-situ evidence linking molecular precursors, cluster formation, particle growth, and CCN activation within the same aerosol population. This **end-to-end** observational continuity distinguishes our results from earlier inference-based approaches (see General Comments by Reviewer 1&2) and supports the hypothesised role of NPF as a source of secondary CCN under Arctic conditions.

We agree with the reviewer that quantifying the contribution of such events to regional CCN budgets, cloud properties, and radiative forcing is essential for assessing climatic impacts. Addressing these questions rigorously, however, requires multi-year statistics, spatial representativeness, and coupled regional-to-global modelling, which are beyond the scope of process-level studies, such as this work.

Accordingly, the aim of the present work is not to test the full climate feedback loop conceptualised in the CLAW hypothesis, but to provide **mechanistic observational constraints on specific links within that framework**, namely the molecular processes governing nucleation and subsequent growth to CCN sizes under ambient Arctic conditions. As with recent observational studies, we therefore discuss implications for CCN and climate qualitatively. The observation of multiple events in which newly formed particles grow to CCN sizes provides an important process-level constraint that can inform future modelling and synthesis efforts, rather than a standalone assessment of climatic impact. We have clarified this scope and its limitations in the revised manuscript.

Furthermore, we also note that the complexity of the real atmosphere, together with often substantially lower concentrations of key chemical species, makes the identification of relevant clusters and OOMs considerably more challenging (e.g., due to peak interferences) than in chamber studies, as illustrated in Fig. S10. Our analysis nevertheless enables hypothesis testing under these ambient conditions.

Finally, we have re-written supplementary text S1 (Text S7 in the original manuscript) to provide better context for the reader. Please see response to reviewer 1 comment 30, as well as the General Response.

Comment 2: Novelty of Methods and Analytical Approach. A second concern relates to the novelty of the measurement techniques and analytical framework. The authors employ a comprehensive suite of mass spectrometers and particle sizing instruments, which provides one of the most detailed characterizations of nucleating and condensing species in the Arctic to date. However, none of the individual techniques, nor their combination, is novel in itself; similar instrumental setups and conceptual frameworks have been used extensively in both laboratory (e.g., CLOUD) and field studies. Moreover, the chemical analysis of condensing vapors is restricted to nitrate-CIMS, which selectively detects highly oxygenated species (ELVOCs) but leaves large gaps in less oxygenated or semi-volatile compounds that can be equally important for particle growth. Complementary ionization methods (e.g., Br^- CIMS) would have broadened the volatility range and helped identify additional contributors, potentially from other sources. On the modeling side, the authors rely on well-established treatments of aerosol nucleation and growth but do not introduce new theoretical or mathematical concepts that advance our mechanistic understanding of NPF. In conclusion, while the

dataset is valuable and regionally unique, the methodological and analytical advances are incremental rather than novel, which limits the conceptual impact of the study.

Response: We agree that the individual instruments and analytical frameworks employed in this study are well established within the atmospheric science community. The novelty of the work lies in their combined application as a comprehensive observational suite to address long-standing knowledge gaps in an under-studied region of the Arctic (see revised supplementary Text 1 (previously S7 in the previous version), and Response to Reviewer 1 comment 30). Our conclusions are based on one of the most comprehensive set of complementary instruments in the Arctic.

Table R1. Summary of NPF-relevant Instruments Used in Arctic Campaigns.

**Instrument**	**Beck et al (2021)**	**MOCCHA campaign** Baccarini et al (2021)	**NETCARE campaign(s)** (Abbatt et al, 2019)	**MOSAIC campaign** (Heutte et al, 2023; Angot et al., 2022)	**This study**
**NO_3 CIMS**	√	√	×	√	√
**Api-ToF**	√	√	×	√	×
**PTR-MS**	×	×	×	×	√
**HR-ToF-AMS**	×	×	√	√	√
**(Neutral cluster and) air ion spectrometer**	√	×	×	×	√
**Mobility particle size spectrometer (e.g., SMPS, DMPS)**	√	√	√	√	√
**CCN counter**	×	×	√	√	√
**SP2XR**	×	×	×	×	√
**Traditional SO2 analyser***	×	√	×	√	√
**SO2 analyser (LIF or Acetate-CIMS)** $	×	×	√	×	√
**O3 analyser**	×	×	√	√	√
**NO/NO2 analyser**	×	×	√	×	√
**CO analyser**	×	×	√	√	√

*** Detection limit is too high for Arctic environment due to its cleanliness.**
$ Detection limit of 25 and 33 ppt, respectively

We acknowledge the inherent selectivity of the nitrate-CIMS. Nevertheless, our growth model demonstrates that the species it detects are sufficient to explain a large fraction of the observed particle growth. Given the limited contribution of inorganic acids to growth, our data provide a strong support for the role of OOMs in Arctic particle growth.

We do concur, however, that a wider range of instruments, e.g., a Br^- or I^- CIMS, could measure less oxygenated organics alongside a wide range of halogen-containing molecules, which would indeed make for a valuable dataset in future field campaigns.

Comment 3: Broader Impacts and Generalization. My third concern relates to the broader impact and generalizability of the findings. The measurements, while technically challenging and unique for

this region, are limited to a one-month period in the marine boundary layer around south of Greenland. As the authors themselves acknowledge, Arctic NPF mechanisms are highly diverse and vary with both season and location — for example, processes linked to melting sea ice can dominate in some regions, whereas algal emissions or other biological sources may be more important elsewhere. In addition, recent work highlights that summertime Arctic aerosol is increasingly influenced by large-scale Boreal wildfires, a source not addressed here. These limitations raise concerns about how representative the observations are for the wider Arctic.

Response: We agree that the Arctic is heterogeneous (e.g. Brean et al. [46]) with new particle formation mechanisms varying by season, location, and dominant source regimes. The present study is not intended to capture this full diversity, but to provide **process-level constraints** on specific nucleation pathways operating in the marine boundary layer.

The iodine–sulfur multicomponent mechanism identified here is not proposed to be site-specific, but rather to reflect a **chemical pathway enabled by the co-presence of sulfuric acid and iodine oxoacids,** which appear to be present at multiple sites and seasons in the Arctic (e.g., Beck et al.; Beccarani et al.). These species originate from precursors that are widespread in the marine boundary layer, although their relative strengths vary between the marginal ice zone, open ocean, and biologically active regions. Such variability affects the frequency and intensity of events, but does not preclude the operation of the underlying mechanism.

The reviewer notes that iodine sources can differ between melting sea ice and biogenic emissions. Importantly, both abiotic (e.g. ozone deposition to ice or seawater) and biotic (e.g. algal activity) processes can lead to the co-emission of iodine- and sulfur-containing species. On this basis, the mechanism described here may be relevant across a range of Arctic marine source regimes, while recognising that the relative importance of individual sources and resulting mechanisms may vary locally.

We further note that the increasing influence of boreal wildfire emissions on summertime Arctic aerosol represents an additional, largely continental source regime that lies outside the scope of this marine boundary layer study. The presence of such influences does not negate the relevance of marine-driven nucleation pathways, but rather underscores the coexistence of multiple aerosol formation mechanisms in the Arctic.

Accordingly, the contribution of this work is framed in terms of **establishing the operation of a multicomponent nucleation pathway under ambient marine Arctic conditions**, rather than assessing its prevalence across all Arctic environments. Evaluating spatial representativeness and relative importance across seasons and regions will require coordinated multi-site observations and modelling, which we identify as an important direction for future research.

Please see also "General Response".

Comment 4: Beyond representativeness, I also question the broader usability of the results. The study does not propose a new mechanism, parameterization, or transferable framework that could be readily used by the atmospheric measurement and modeling communities. In fact, the most straightforward way to retrieve a parameterization for multi-component H_2SO_4–HIO_3 nucleation would be by relying on previous CLOUD results obtained under controlled conditions and at different temperatures. As such, the impact of this paper on advancing predictive capability remains limited. This also reduces

its relevance to the broad readership of Nature, where conceptual advances and generalizable implications are typically expected.

Response:

We agree that CLOUD chamber studies provide valuable data for essential parameterisation under controlled conditions, including at different temperatures. This is indeed why we suggested that future chamber studies should investigate the hypothesis on the role of amines, under concentrations representative of ambient air (e.g., representative concentrations of DMA (a few ppt) and VOCs) in H_2SO_4-HIO_x nucleation (see Supplementary Text 1.4).

We believe that laboratory-derived mechanisms and supporting datasets can only translate into improved predictive capability when: (1) their atmospheric occurrence is demonstrated in the real atmosphere; (2) their relevance is evaluated under the complexity of ambient conditions rather than (justifiably) simplified laboratory systems; and (3) there are suitable in-situ observations to evaluate model performance.

This study addresses all three (See also response to comment 1 for points (1) and (2)).

With respect to (3), we provide a comprehensive in-situ quantitative dataset spanning the full causal chain — from gases, to molecular clusters, to sub-3 nm particles, to CCN, including nucleation and growth rates. Given the potential importance of the mechanism proposed by He et al. (2023) [56], it is likely already being parameterised in models. Our dataset provides, for the first time to our knowledge, the observational coverage needed to confront emerging parameterisations with atmospheric measurements across the full NPF-to-CCN process chain. This offers a basis for re-evaluating the magnitude of iodine-mediated CCN formation in models.

Field observational and CLOUD chamber studies are complementary to each other.

Technical limitations.

Comment 5: Nucleation. The authors present compelling evidence that NPF is driven by synergetic H_2SO_4–HIO_3 nucleation in the presence of dimethylamine (DMA). I find the detection of nucleating clusters containing H_2SO_4–HIO_3–DMA particularly convincing. Nevertheless, I have two concerns: i. First, I believe Extended Data Fig. 4 is critical for understanding the nucleation results and should replace Fig. 3A, which appears less central to the paper's main argument. ii. Second, I am concerned about the comparison of the measured nucleation rates (J) with the laboratory measurements of H_2SO_4–DMA nucleation rates from Almeida et al. (2013). These older CLOUD results have since been re-evaluated by Kürten et al. (2018 - https://doi.org/10.5194/acp-18-845-2018) and updated with more accurate measurements by Xiao et al. (2021 - https://doi.org/10.5194/acp-21-14275-2021) at different temperatures. Both studies demonstrate that H_2SO_4–DMA nucleation proceeds at the kinetic limit at 5 °C when DMA concentrations are around ~4 ppt, orders of magnitude higher than reported in Almeida et al. This has direct implications for the interpretation of the Arctic nucleation rates. For instance, at 5 °C and $[H_2SO_4] = 2 \times 10^6$ molecules cm^{-3}, Almeida et al. reported $J \approx 10^{-2}$ cm^{-3} s^{-1}, whereas Xiao et al. measured $J \approx 10^2$ cm^{-3} s^{-1} under similar conditions. The authors report $J \approx 3 \times 10^{-1}$ cm^{-3} s^{-1} at ~0 °C for the same $[H_2SO_4]$. When benchmarked against the outdated Almeida et al. data, the authors conclude that H_2SO_4 alone cannot explain the observed nucleation rates. However, when compared with the updated Xiao et al. results, H_2SO_4–DMA nucleation could in fact account for the observed J values, without necessarily invoking an additional HIO_3 contribution. While I do

not exclude an important role of HIO_3, the interpretation of the nucleation mechanism should be reconsidered on the basis of the newer CLOUD data.

Response: The conditions under which the correction of of Kürten et al. (2018) are applicable do not apply to our study for two primary reasons.

First, from a practical standpoint, Kürten et al. (2018) could only apply their reconstruction method to experiments where sulfuric acid concentrations were sufficiently high (>~5x10^{6} cm^{-3}) to determine a reliable particle growth rate. The high DMA, high H_2SO_4 experiments from the CLOUD chamber where the reconstruction was applied are not representative of our environment where, for example, DMA monomers were below the detection limit of the nitrate CIMS, which we estimate to be a few pptv [23-26]. The low-DMA data of interest were therefore not corrected.

Second, this correction accounts for self-coagulation. The likelihood of coagulation is proportional to the square of the particle concentration. Kürten et al. (2018) corrected data where the $J_{1.7}$ values were on the order of 10^{2} cm^{-3} s^{-1}. For the low-DMA datapoints of Almeida et al. (2013), the reported $J_{1.7}$ values are in the range of 10^{-2} to 10^{-1} cm^{-3} s^{-1}, therefore the likelihood of self-coagulation is much smaller and the correction would be much smaller.

Regarding Xiao et al., (2021), we agree these are relevant data, we did not include these as they were performed at high NH_3 concentrations (1-2 ppb), while our measured NH_3 concentrations were consistently below our LOD of 65 pptv. As NH_3 can form more intermolecular bonds than DMA, NH_3 can help stabilise DMA-sulfuric acid clusters. This difference of several orders of magnitude in concentration between our data and that of Xiao et al (2021) fundamentally alters the nucleation mechanism, enhancing formation rates by orders of magnitude compared to DMA alone [57, 58].

Hasan et al. [58] used quantum chemistry to model the stability of sulfuric acid-ammonia-DMA clusters under two distinct scenarios: a 'low concentration' regime ([NH_3] = 10 ppt, [DMA] = 1 ppt), which closely reflects our experimental conditions, and a 'high concentration' regime ([NH_3] = 10 ppb, [DMA] = 10 ppt), which reflects the conditions of the proposed comparison.

They demonstrate that in the low concentration regime, DMA is the dominant stabilising agent for the initial clusters. Clusters with a high proportion of ammonia are less stable. In the high concentration regime, the increased abundance of NH_3 dramatically enhances the stability of clusters containing ammonia. The study shows that under these conditions, NH_3 begins to appear in the most stable clusters at much smaller sizes.

Therefore, we include the data of Xiao et al (2021) in Fig. S8, but argue that a comparison with the low-DMA data of Almeida et al. (2013) is the most appropriate comparison, however, as no comparison is completely representative, we opted keep this figure in the SI. We include the following in the main text (line 209-211)

"The measured H_2SO_4 concentration is too low to explain $J_{1.7}$ when compared to CLOUD chamber experiments involving H_2SO_4-NH_3-H_2O or H_2SO_4-DMA-H_2O (<5 pptv DMA, 10 pptv NH_3) [31]. **Our experiments similarly do not match experiments with 4 pptv DMA and 10 ppb NH_3 [32]** (Fig. S8A)."

And our figure caption now reads as follows:

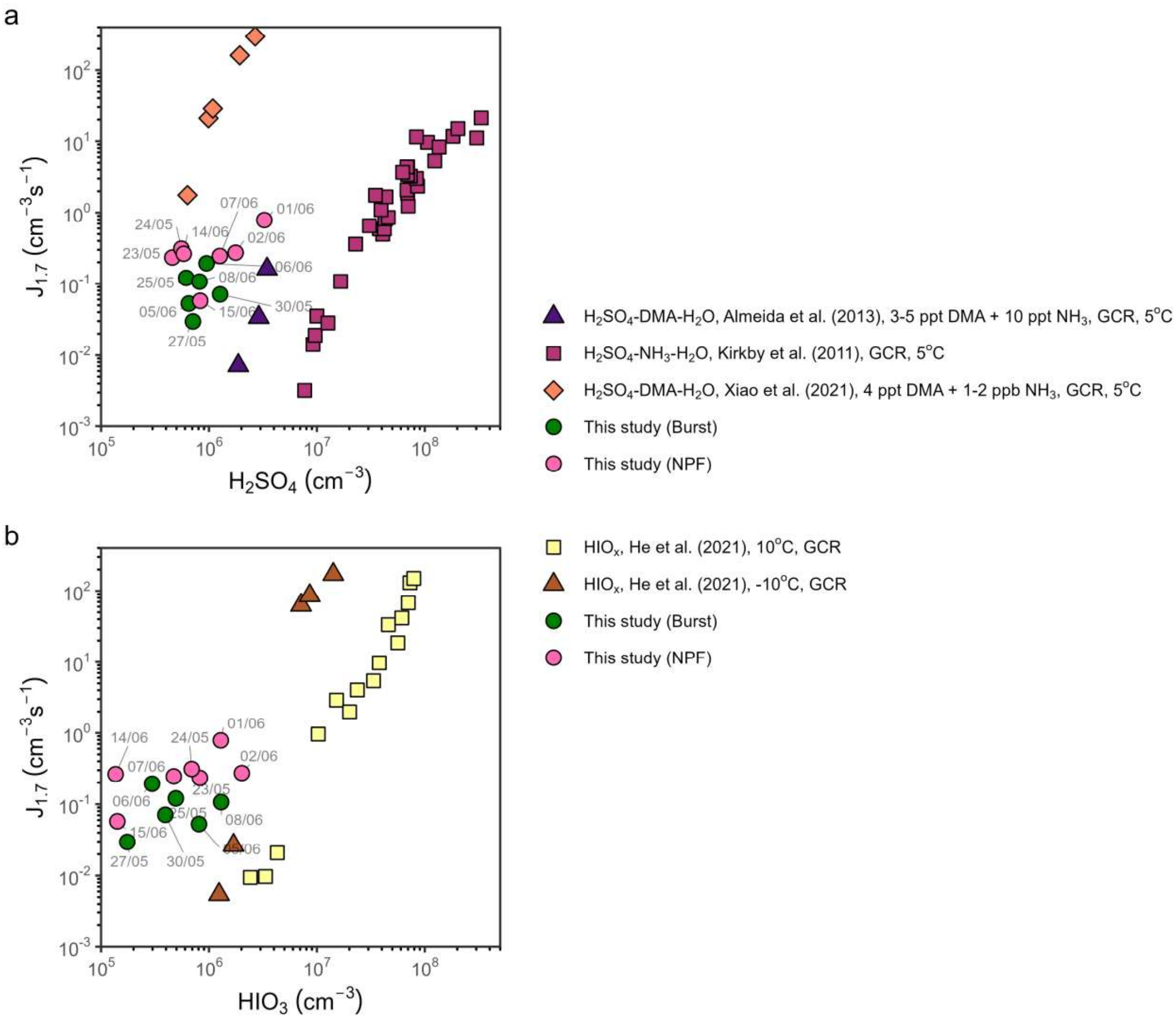

"Figure S8: Comparison of nucleation rates for the H_2SO_4, and HIO_3 systems with previous studies [10,11]. (A) Particle formation rates $J_{1.7}$ plotted against H_2SO_4 concentrations. Data points from CLOUD chamber experiments represent H_2SO_4-DMA-H_2O with 3-5 ppt DMA and 10 ppt NH_3 (**purple triangles**) [10], **H_2SO_4-DMA-H_2O with 4 ppt DMA and 1-2 ppb NH_3 (orange diamonds)** [12], and H_2SO_4-NH_3-H_2O (**magenta squares**) [10]. **The H_2SO_4-DMA-H_2O data from Almeida et al.** [13] **are presented without the self-coagulation correction later described by Kürten et al.** [14]**; this correction is not available for these specific low-concentration experiments and is negligible given the low particle concentrations, and the experiments of Almeida et al. are likely the most representative of our measured conditions.** (B). Particle formation rates $J_{1.7}$ plotted against HIO_3 concentrations. Data points are from CLOUD chamber experiments at 10 °C (yellow squares) and –10 °C (brown triangles) [11]. **For both plots,** pink circles represent NPF events, while green circles denote burst events, and each data point is labelled on the plot using the day-month format."

Finally, to provide further quantitative constraints on the abundance of amines, we performed an inverse kinetic growth calculation. While gas-phase amines were below the detection limit of the

nitrate-CIMS, mass growth of particle-phase DMA and TMA were observed in the AMS data. We include peak fits below (Figure R1)

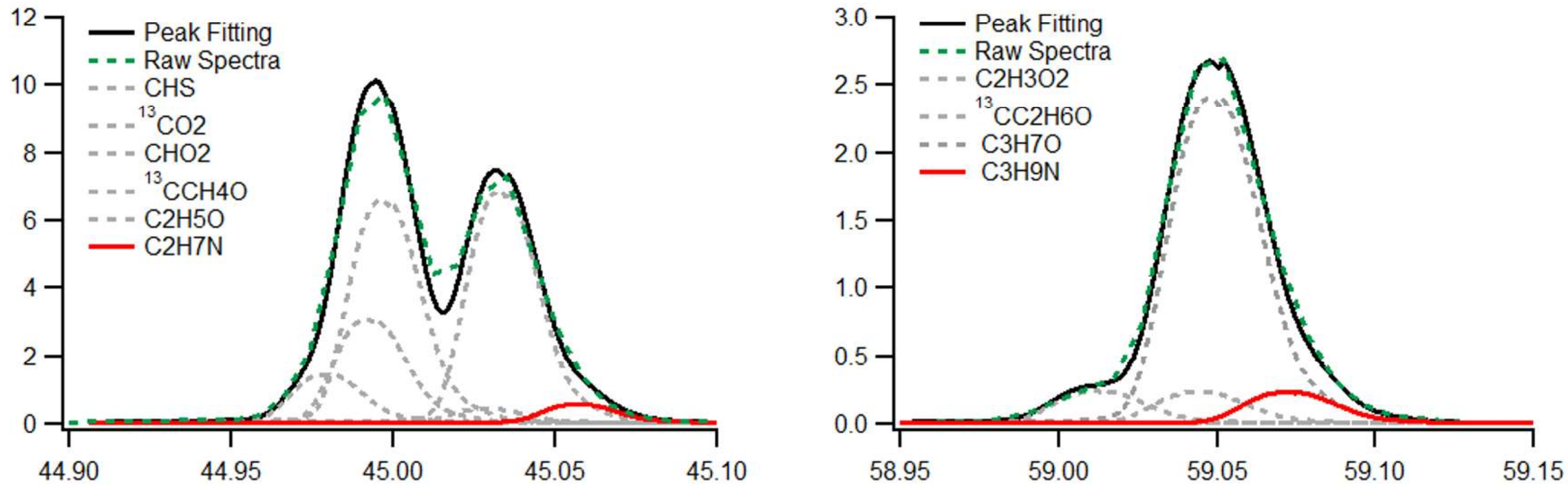

Figure R1: Peka fits for dimethylamine and trimethylamine in the AMS, units of Hz/ns. Data shown are from June 8th 16:30-June 9th 8:30

By employing the condensation growth framework described by Tröstl et al. (*Nature*, 2016), we can work backwards from the observed rate of change in particle-phase mass to estimate the gas-phase concentration required to sustain this growth.

The standard radial growth rate equation is given by:

dDp/dt = [(m_mol * c_mean * alpha * beta) / (2 * rho_p)] * (C_gas - C_eq)

Rearranging to solve for the necessary gas-phase concentration (**C_gas**):

C_gas = [(2 * rho_p) / (m_mol * c_mean * alpha * beta)] * [(Dp / 3) * (dM/dt) / M] + C_surf

C_gas = [(2 * rho_p) / (m_mol * c_mean * alpha * beta)] * (dDp/dt) + C_eq

As the AMS measures bulk mass rather than diameter growth directly, we substitute the radial growth term (Dp/dt) using the geometric relationship **Dp/dt = (Dp / 3) * (dM/dt) / M** This yields the final expression used for our estimation:

C_gas = [(2 * rho_p) / (m_mol * c_mean * alpha * beta)] * [(Dp / 3) * (dM/dt) / M] + C_eq

Where the Equilibrium Surface Concentration (C_eq) is: **C_eq = C_star * a_ip * K_ip**

And the Kelvin Term (K_ip) is: **K_ip = 10^((4 * sigma * Mw) / (2.303 * R * T * rho_p * Dp))**

Variable definitions: dM/dt is the rate of change of particle mass; M is current particle mass; Dp is particle diameter; beta is the Fuchs-Sutugin transition correction factor; c_mean is the mean thermal velocity of gas; C_star is saturation vapour concentration; a_ip is the particle-phase activity; and sigma is the surface tension.

We applied this inverse model to the AMS amine time series during the major growth event. The results are presented in **Figure R2**, which displays the particle phase mass and mass growth rate for the amines, the evolution of particle diameter from the PNSD, and the derived gas-phase mixing ratios.

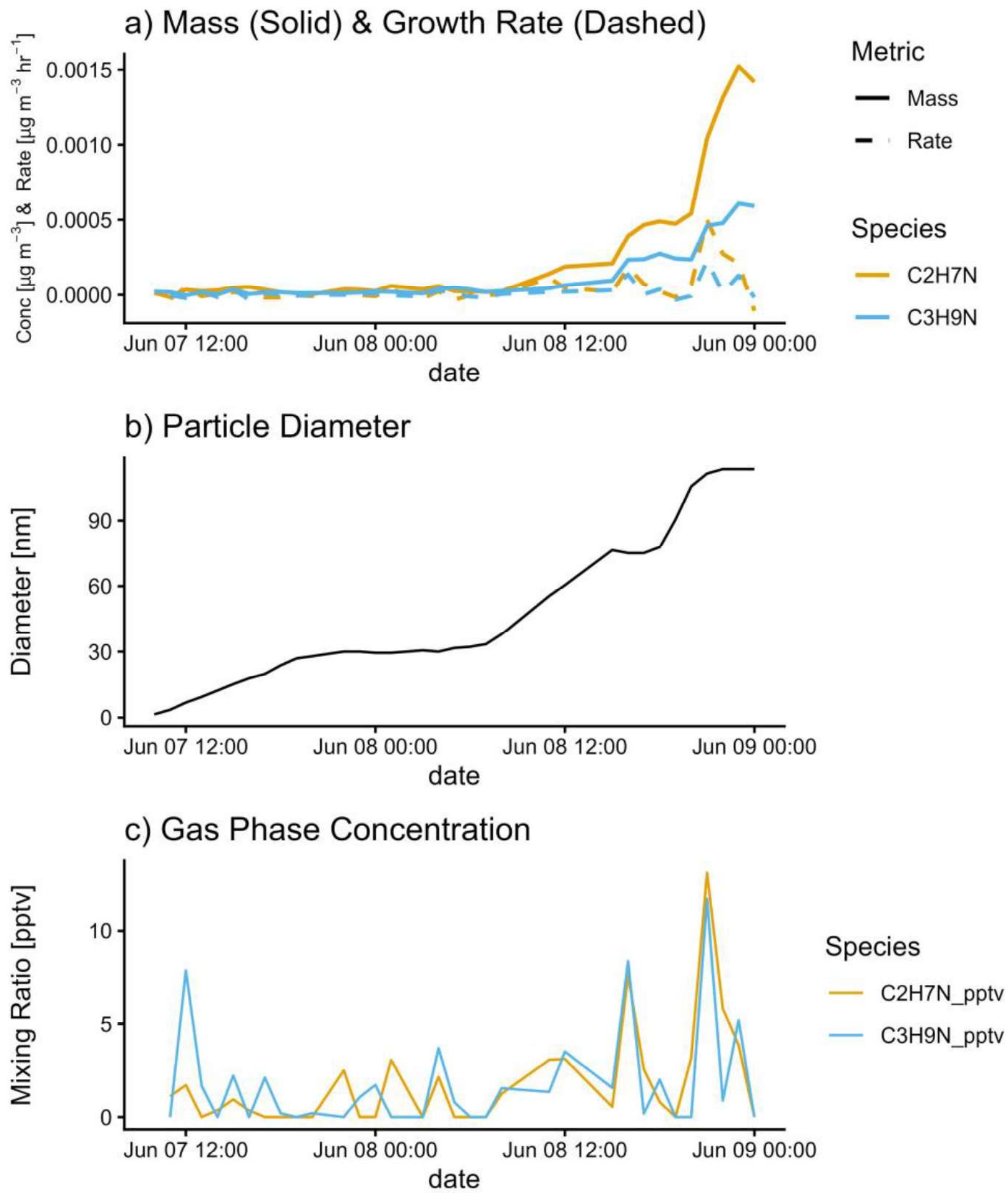

Figure R2: Amines in the AMS. (a) the particle phase concentrations of C_2H_7N and C_3H_9N, and the rate of change in particle phase C_2H_7N and C_3H_9N; (b) the measured particle diameter from the PNSD; (c) the calculated amine mixing ratios.

Assuming irreversible condensation (where C_gas >> C_surface), the model indicates that an average gas-phase concentration of approximately **1.8 pptv** (for both DMA and TMA) is sufficient to drive the observed accumulation of amine mass in the particle phase, and is also sufficient to enhance our observed J values.

To test the robustness of this estimate against the thermodynamic uncertainties of amines (specifically the acid-base chemistry that leads to the formation of low-volatility aminium salts) we performed two additional sensitivity runs allowing for reversible condensation (re-evaporation):

1. Assuming amines behave similarly to Low Volatility Organic Compounds (LVOCs) with a saturation concentration (C*) 10^{-3} ug m^{-3} and activity a_ip = 1).
2. Assuming a high saturation concentration characteristic of pure amines (C*) 10^{6} ug m^{-3} but a very low particle-phase activity a_ip = 10^{-7} to represent strong binding with acids and organics.

Both sensitivity scenarios converged on a required gas-phase concentration of approximately **2 pptv**.

This analysis demonstrates that a mixing ratio of ~2 pptv is sufficient to explain the observed particulate amine mass. This concentration is likely below the detection limit of the nitrate-CIMS, explaining why the monomer was not directly observed in the gas phase. However, even at these trace levels, amines are capable of participating in nucleation and growth, and these numbers are consistent with the low-DMA experiments of Almeida et al (2013) [5]. Thus, the lack of a gas-phase CIMS signal does not contradict the presence of amines; rather, the AMS data and kinetic modelling strongly support their presence and participation in the observed NPF events.

Comment 6: Growth. The authors interpret their particle growth results primarily based on nitrate-CIMS measurements, concluding that ELVOCs dominate growth, whereas low-volatility and semi-volatile organic compounds (L+SVOCs) play a minor role. However, nitrate-CIMS is inherently selective and does not detect less oxygenated, more volatile compounds. These species were therefore excluded from both the measurements and the growth model, despite their potential importance, particularly in the later stages of growth toward larger particle sizes. This limitation raises questions about the completeness of the growth analysis and the robustness of the conclusion that ELVOCs alone dominate the observed growth.

Response: Our original manuscript stated that (Line 286-287)

"The modelled particle GRs consistently show that OOMs, particularly the low volatility (LVOC) and extremely low volatility (ELVOC) fractions, drive modelled particle growth".

We do acknowledge that the NO_3^- CIMS is selective, mostly measuring organics with 2 or more hydrogen bond donor groups [30] (typically 4 or more oxygen atoms). This often means the NO_3^- CIMS has limited sensitivity to many SVOCs, but the sensitivity to LVOCs is still high (our volatility distributions show that the concentrations of SVOCs is, in fact, quite high, see Fig. S14, and other work in fact shows the difference between nitrate and bromide CIMS is not overwhelmingly large [55]). These SVOCs can indeed contribute to particle growth, especially at larger sizes. We therefore provide the following discussion in the supplementary text, which we have expanded to make this clearer (bold text) (line 1276-1283):

"The NO_3^- CIMS lacks sensitivity to certain OOMs, such as those with only one hydrogen bond donor group [39], and our OOM concentrations are therefore a lower limit on the real OOMs concentration in the environment. **Specifically, it has a well-known lower sensitivity to less oxygenated, more volatile species like semi-volatile organic compounds (SVOCs), which were therefore not constrained by our measurements**. Despite those, our modelled condensation rates match the evolution of the PNSD within ~50%, and the resultant mass concentrations match the sulfate to organic ratio we measure in the AMS. We therefore capture the key processes relevant to particle growth."

Finally, our main text now reads as follows, following your suggestion and that of other reviewers (line 268-293):

"We observed six events with measurable particle growth rates. Particles formed during these NPF events consistently grew to 20 nm within a few hours. GRs for particles of diameters 3 to 15 nm varied between 1.5 to 3.6 nm h^{-1} (Table S1), which are relatively high among those observed in long-term data in the Arctic (Fig. S7), but akin to measurements made in the region west of Greenland [40]. To investigate drivers of particle growth during NPF events, we utilised a dynamic aerosol growth model incorporating concentrations of gas-phase acids (H_2SO_4, MSA, HIO_3) and OOMs (see Methods 11). Saturation vapor concentrations were calculated following the methodology outlined by Mohr et al. [41]. Our model typically accounts for half or more of the observed growth rates (Fig. S17). This underprediction is expected, as the NO_3^- CIMS provides a lower-limit estimate on the total OOM concentration. Specifically, it has a well-known lower sensitivity to less oxygenated, more volatile species like semi-volatile organic compounds (SVOCs), which were therefore not constrained by our measurements. The condensation of these unmeasured SVOCs likely accounts for a significant fraction of the unexplained growth, particularly for the later stages of growth towards larger particle sizes. Other factors, such as multiphase reactions and coagulation growth, as well as potential change in meteorological conditions may also explain the discrepancies between observed and modelled GRs (see Supplementary Text 5).

The modelled particle GRs consistently show that OOMs, particularly the low volatility (LVOC) and extremely low volatility (ELVOC) fractions, drive particle growth, with I-OOMs estimated to contribute 7-23% to modelled GRs (see Supplementary Text 5). The fact that inorganic acids such as H_2SO_4 and HIO_x contribute little to particle growth and that this lower-limit OOMs' concentration can explain such a large fraction of the observed growth provides strong evidence that OOMs are indeed the primary drivers. On one occasion (30th May), the model overpredicted growth, likely due to meteorological factors not captured by the model (see Supplementary Text 5). Our findings provide the first conclusive evidence linking OOMs at a molecular level, including those containing iodine, to the growth of new particles in the Arctic."

An additional point is that the contribution of inorganic acids to the growth of the new particles is small. Any growth that is unaccounted for is not due to these inorganic acids.

Comment 7: While I am convinced that the largest source of uncertainty in the growth modeling arises from the determination of condensing vapor concentrations, an additional, and still significant, source stems from the estimation and parameterization of compound volatilities. From my understanding, the authors apply the same parameterization to both monoterpene- and aldehyde-derived oxidation products. How robust is this assumption? And how do uncertainties in volatility estimates propagate into the modeled growth rates? The authors should explicitly assess this by presenting bounds on the modeled growth rates that account for uncertainties in both vapor concentrations and volatility.

Response: The linear equation we use is based on group-contribution estimations, which implicitly assume that the functional groups are known but the molecular structure is not (or it is unimportant) [29]. The first version of this equation from Donahue et al. (2011) presumed that a mixture of carbonyl and alcohol functional groups are present, with the former reducing volatility by 1 order of magnitude, and the latter reducing volatility by 2.4 orders of magnitude. The mean of these numbers

is 1.7, therefore the effect of one oxygen molecule was presumed to be 1.7 orders of magnitude ($C^* \propto nObO$ where nO is number of oxygens, and bO is 1.7). As HOMs (a.k.a. OOMs) contain many -OOH and -OO- functionalities, the equation we use in our paper has been modified and contains a non-linear term (in bold)[30]:

$$\log_{10} C_i^*(300K) = (n_0 - n_\mathrm{C})b_\mathrm{C} - (n_\mathrm{O} - 3n_\mathrm{N})b_\mathrm{O} - \mathbf{2}\,\frac{\boldsymbol{(n_\mathrm{O} - 3n_\mathrm{N})n_\mathrm{C}}}{\boldsymbol{(n_\mathrm{C} + n_\mathrm{O} - 3n_\mathrm{N})}}\boldsymbol{b_\mathrm{CO}} - n_\mathrm{N}b_\mathrm{N} - (n_\mathrm{I}b_\mathrm{I})$$

where $n_0 = 25$, $b_C = 0.475$, $b_O= 0.2$, $b_{CO}= 0.9$, $b_N= 2.5$, and $b_I=2.0$. This final term, which does stem from monoterpene research, specifically accounts for how the volatility reduction per oxygen changes as the molecule becomes more oxygenated (e.g., shifting from carbonyl/alcohol groups to a higher proportion of hydroperoxide groups). This behaviour is a general feature of autoxidation, not a phenomenon exclusive to monoterpenes.

We have good reason to believe aldehydes undergo autoxidation, as their autoxidation has been observed in the laboratory [33, 34], and the presence of the C=O group makes the adjacent hydrogen more labile, facilitating the abstraction [33, 34]. It is therefore reasonable to assume their oxidation products will have a distribution of functional groups (hydroperoxides, carbonyls, etc.) that can be approximated by this same general parameterisation.

While we acknowledge that specific parameters for aldehyde-derived OOMs are not yet available, this group-contribution method is the most robust and mechanistically appropriate approach currently available. Furthermore, as the OOM spectrum was not exclusively dominated by aldehyde-OOMs across all events, any potential uncertainty introduced by this assumption is unlikely to alter the overall conclusions of our growth model.

Comment 8: Peak fitting and assignments. I commend the authors for their effort in peak fitting and molecular assignments. The identification of H_2SO_4, HIO_3, DMA clusters, and I-containing OOMs appears convincing and is a strength of the study. However, I have serious doubts regarding the reliability of the assignments for other OOM peaks, particularly at higher m/z, where spectral complexity increases and interferences are more likely. To strengthen confidence in their conclusions, the authors should present their fitting workflow in more detail, including the treatment of overlapping peaks, and provide a quantitative assessment of fitting errors. Moreover, they should evaluate the sensitivity of their results (e.g. PMF) to these uncertainties, as misassignments at high m/z could significantly affect the inferred chemical composition of condensing vapors and, consequently, the interpretation of their sources. Another concern is whether the I-OOMs are covalently bonded compounds or clusters between iodine oxo-acids and OOMs. Could the authors exclude the presence of these clusters?

Response: Considerable efforts were made, together with Tofware package developers, who are co-authors (Harald Stark and Doug Wosnop) in peak fitting. We are confident on the assignment of OOMs peak.

Regarding our workflow, we can provide more detail (line 548-567):

"Data analysis was conducted using the Tofware package (Tofwerk AG) and Igor Pro 9.0.4 (Wavemetrics Inc.). The mass spectrometer's resolution was 3700 at m/Q 201. Peak fitting was performed following the procedure outlined in Tofware. **The fitting process is initiated by defining a mass calibration, before the instrumental peak shape and width is established from**

well-isolated peaks across the mass spectrum. Following this, mass calibration is re-applied and peaks are fit. For spectrally complex regions with overlapping signals, a multi-peak fitting procedure was employed [72], which always accounted for almost all of the signal at this mass (see peak fits in Figs. S5, S10, S11), after which molecular formulas were assigned. As peaks were assigned, the mass-calibration was refined, ultimately utilising NO_3^-, $HNO_3NO_3^-$, HSO_4^-, $C_6H_5NO_3NO_3^-$, and $C_{10}H_4F_{12}ONO_3^-$. $C_{10}H_4F_{12}ONO_3^-$ is a contaminant likely from the tube walls. Fluctuations in mass calibration resulted in no more than a 5% variation in the total fitted intensity for a representative OOM. The mass calibration and precision of peak separation follow Stark et al. (2015) [72] and Cubison et al. (2015) [73], respectively.

Our assigned OOM formulae avoided hypervalent molecules, molecules with H:C ratios below 0.5, or O:C ratios exceeding 2, which is the upper limit observed in laboratory studies. Molecules that would not be detected by NO_3^- CIMS (less than four oxygen atoms), as well as deprotonated OOMs (as opposed to clusters with NO_3^-), were deprioritized. Due to the increasing difficulty in confidently assigning molecular formulas at higher masses with our instrumental resolution, we did not assign peaks beyond an m/z of ~550. Iodine-OOMs were assigned when no reasonable non-iodine containing molecular formulas matched"

Regarding uncertainties, with our limited mass resolution (~4000), it becomes difficult to assign peaks confidently past m/Q ~400. What peaks are present past m/Q 500 made a very small contribution to total signal, and therefore we set a threshold to not assign peaks much past m/Q 500. Regarding misassignments at lower m/Qs, we provide the following argument regarding the condensing vapours: at m/Q 264 there are a number of possible OOMs, which we present below in Table R1. These OOMs all have very different compositions, covering a large range of masses (our mass accuracy was ~15 ppm). We find that all of these, except those with multiple nitrogen atoms or low numbers of oxygens have similar volatilities and resultant GRs. Therefore, while there are uncertainties in assignment (which we have attempted to minimise), the effect of this on the calculated GRs would be relatively small (especially as these errors can go both ways—one assignment may induce a higher C*, while the next may induce a lower C*).

Table R1: Six potential OOMs at m/Q 264, their difference in mass from the closest integer mass in ppm, their estimated volatilities, and the growth rate for a 25 nm particle, presuming a mass fraction of 10% and an OOM concentration of 10^7 cm^{-3}

Formula	ppm difference from 264 m/Q	C* ($\mu g\ m^{-3}$)	GR_{25} ($nm\ h^{-1}$)
$C_9H_{12}O_9$	182	-2.3	1.5
$C_{10}H_{16}O_8$	320	-2.5	1.7
$C_{10}H_{20}O_6N_2$	500	2.1	0
$C_{11}H_{20}O_7$	458	-2.5	1.6
$C_{12}H_{24}O_6$	596	-2.2	1.5
$C_{13}H_{28}O_5$	733	-1.8	0.7

Regarding misassignments causing misunderstandings of our PMF factors, consider two plausible candidates from Table R1: $C_{10}H_{16}O_8$ (a typical monoterpene oxidation product) and $C_{12}H_{24}O_6$ (an aldehyde oxidation product). Their exact masses differ by ~0.073 Da. Given our instrument's

resolution, the calculated peak width (FWHM) at this mass is ~0.071 Da, meaning the peaks are indeed resolvable. More importantly, their mass difference of ~276 ppm is an order of magnitude larger than our instrument's mass calibration accuracy of 15 ppm. This high mass accuracy provides strong confidence that even if the peaks were to overlap, we would not misassign these OOMs, and our attribution of PMF factors is robust. The same applies for any iodine-containing OOM, where the negative mass-defect of iodine confers a ~350 ppm shift.

Finally, regarding OOM-IO_3 clusters, we regard this as unlikely, as the concentration of measured I-OOMs is equal to the concentration of neutral HIO_3 measured in the NO_3^- CIMS. In order for this to be the case, 1) neutral HIO_3 would need to form clusters with neutral OOMs which not only exist in the atmosphere, but also are resistant to evaporation upon charging. This is not seen for any other strong acids; 2) all of our measured HIO_3 would need to in fact be IO_3^-, which then forms stable clusters with OOMs and enters our mass spectrometer, which is also unlikely.

Comment 9: Uncertainties. The authors mention uncertainties in individual parameters at several points, but they do not propagate these errors through to the reported nucleation rates, growth rates, or concentrations of nucleating and condensing species. A comprehensive error propagation would allow readers to evaluate the robustness of the quantitative results and should be reflected in the figures as error bars or uncertainty ranges. I view the absence of this as a weakness of the study, even if the overarching conclusions are unlikely to change.

Response: We appreciate the reviewer’s suggestion regarding the rigorous treatment of uncertainties. Here, we have adopted the methodology established in recent high-impact field studies of new particle formation (e.g., Curtius et al., 2024; Baccarini et al., 2020) [59, 60] while adapting the baseline constraints from chamber literature (Almeida et al., 2013; Tröstl et al., 2016) [5, 61]. We opted for estimated uncertainty factors rather than continuous formal error propagation for three primary reasons.

First, the error budget in these measurements is dominated by systematic uncertainties in calibration and transmission rather than statistical counting errors. Chamber studies define the lower bound of uncertainty for CIMS-measured sulfuric acid at +100%/−50% (factor of ~2) due to calibration limits (Almeida et al., 2013). To account for the additional challenges of ship-borne sampling—such as inlet turbulence and rapid airmass advection—we apply a conservative factor of 3 uncertainty to our inorganic acid measurements.

Second, for organic compounds, chamber studies indicate larger uncertainties. Tröstl et al. (2016) estimate ELVOC uncertainties at +80%/−45% but note that semi-volatile species (LVOC/SVOC) can be underestimated by a factor of 4 due to charging inefficiencies. Consequently, we adopt a factor of 5 uncertainty for total OOM concentrations to robustly bracket these uncertainties.

Third, regarding particle formation rates J, the calculation relies on the balance equation (Eq. 7) where the GR is a dominant term. Following standard methodologies, we estimate the uncertainty in GR retrieved from the PNSD to be approximately ~50% for field data [62]. When propagated alongside uncertainties in the coagulation sink and particle counting, this results in a combined uncertainty for J of approximately a factor of 3. This falls within the "factor of 2–5" range estimated in recent polar nucleation studies (Boyer et al., 2025) [63].

Crucially, our conclusions rely on relative changes in signal that span orders of magnitude. Because the signal strength during nucleation events exceeds these estimated combined uncertainty factors, the qualitative conclusions regarding the drivers of nucleation remain robust. We have updated the Methods section to explicitly state these aggregated uncertainty ranges.

In methods section 3 we include the following text (line 540-547):

"Uncertainties in CIMS measurements are derived from chamber experiments and subsequently adapted for field conditions. While CLOUD chamber studies estimate sulfuric acid uncertainties at +100%/−50% (approx. factor of 2) [74], we adopt a conservative factor of 3 for all inorganic acids to account for additional inlet turbulence and environmental variability inherent to ship-borne measurements. For OOMs, chamber studies suggest ELVOC uncertainties of ~+80%/−45% but acknowledge that less oxidized species (LVOCs/SVOCs) may be underestimated by up to a factor of 4 due to lower charging efficiencies [71]. Therefore, we apply a factor of 5 uncertainty to total OOM concentrations to encompass these biases"

In methods section 11 we include the following two sections (line 854-860)

"Uncertainties in GR determination are estimated to be approximately ~50% [96].The uncertainty in J is estimated based on the propagation of systematic errors in the input parameters of the balance equation (Eq. 7), including the ~50% uncertainty in the growth rate term GR and uncertainty in the quantification of particle count (~30%) [97], accounting for additional inhomogeneity inherent to mobile marine sampling, we estimate the total combined uncertainty for our ambient formation rates to be a factor of 3. This falls within the uncertainty range of factor 2–5 reported in recent polar aerosol studies [98]."

References:

1. He, X.-C.; Simon, M.; Iyer, S.; Xie, H.-B.; Rörup, B.; Shen, J.; Finkenzeller, H.; Stolzenburg, D.; Zhang, R.; Baccarini, A.; Tham, Y. J.; Wang, M.; Amanatidis, S.; Piedehierro, A. A.; Amorim, A.; Baalbaki, R.; Brasseur, Z.; Caudillo, L.; Chu, B.; Dada, L.; Duplissy, J.; El Haddad, I.; Flagan, R. C.; Granzin, M.; Hansel, A.; Heinritzi, M.; Hofbauer, V.; Jokinen, T.; Kemppainen, D.; Kong, W.; Krechmer, J.; Kürten, A.; Lamkaddam, H.; Lopez, B.; Ma, F.; Mahfouz, N. G. A.; Makhmutov, V.; Manninen, H. E.; Marie, G.; Marten, R.; Massabò, D.; Mauldin, R. L.; Mentler, B.; Onnela, A.; Petäjä, T.; Pfeifer, J.; Philippov, M.; Ranjithkumar, A.; Rissanen, M. P.; Schobesberger, S.; Scholz, W.; Schulze, B.; Surdu, M.; Thakur, R. C.; Tomé, A.; Wagner, A. C.; Wang, D.; Wang, Y.; Weber, S. K.; Welti, A.; Winkler, P. M.; Zauner-Wieczorek, M.; Baltensperger, U.; Curtius, J.; Kurtén, T.; Worsnop, D. R.; Volkamer, R.; Lehtipalo, K.; Kirkby, J.; Donahue, N. M.; Sipilä, M.; Kulmala, M., Iodine oxoacids enhance nucleation of sulfuric acid particles in the atmosphere. *Science* **2023,** *382*, (6676), 1308-1314.
2. Baccarini, A.; Karlsson, L.; Dommen, J.; Duplessis, P.; Vüllers, J.; Brooks, I. M.; Saiz-Lopez, A.; Salter, M.; Tjernström, M.; Baltensperger, U.; Zieger, P.; Schmale, J., Frequent new particle formation over the high Arctic pack ice by enhanced iodine emissions. *Nature Communications* **2020,** *11*, (1), 4924.
3. Beck, L. J.; Sarnela, N.; Junninen, H.; Hoppe, C. J. M.; Garmash, O.; Bianchi, F.; Riva, M.; Rose, C.; Peräkylä, O.; Wimmer, D.; Kausiala, O.; Jokinen, T.; Ahonen, L.; Mikkilä, J.; Hakala, J.; He, X. C.; Kontkanen, J.; Wolf, K. K. E.; Cappelletti, D.; Mazzola, M.; Traversi, R.; Petroselli, C.; Viola, A. P.; Vitale, V.; Lange, R.; Massling, A.; Nøjgaard, J. K.; Krejci, R.; Karlsson, L.; Zieger, P.; Jang, S.; Lee, K.; Vakkari, V.; Lampilahti, J.; Thakur, R. C.; Leino, K.; Kangasluoma, J.; Duplissy, E. M.; Siivola, E.; Marbouti, M.; Tham, Y. J.; Saiz‐Lopez, A.; Petäjä, T.; Ehn, M.; Worsnop, D. R.; Skov, H.; Kulmala, M.; Kerminen, V. M.; Sipilä, M., Differing Mechanisms of New Particle Formation at Two Arctic Sites. *Geophysical Research Letters* **2021,** *48*, (4).
4. Yao, L.; Garmash, O.; Bianchi, F.; Zheng, J.; Yan, C.; Kontkanen, J.; Junninen, H.; Mazon, S. B.; Ehn, M.; Paasonen, P.; Sipilä, M.; Wang, M.; Wang, X.; Xiao, S.; Chen, H.; Lu, Y.; Zhang, B.; Wang, D.; Fu, Q.; Geng, F.; Li, L.; Wang, H.; Qiao, L.; Yang, X.; Chen, J.; Kerminen, V.-M.; Petäjä, T.; Worsnop, D. R.; Kulmala, M.; Wang, L., Atmospheric new particle formation from sulfuric acid and amines in a Chinese megacity. *Science* **2018,** *361*, (6399), 278-281.
5. Almeida, J.; Schobesberger, S.; Kürten, A.; Ortega, I. K.; Kupiainen-Määttä, O.; Praplan, A. P.; Adamov, A.; Amorim, A.; Bianchi, F.; Breitenlechner, M.; David, A.; Dommen, J.; Donahue, N. M.; Downard, A.; Dunne, E.; Duplissy, J.; Ehrhart, S.; Flagan, R. C.; Franchin, A.; Guida, R.; Hakala, J.; Hansel, A.; Heinritzi, M.; Henschel, H.; Jokinen, T.; Junninen, H.; Kajos, M.; Kangasluoma, J.; Keskinen, H.; Kupc, A.; Kurtén, T.; Kvashin, A. N.; Laaksonen, A.; Lehtipalo, K.; Leiminger, M.; Leppä, J.; Loukonen, V.; Makhmutov, V.; Mathot, S.; McGrath, M. J.; Nieminen, T.; Olenius, T.; Onnela, A.; Petäjä, T.; Riccobono, F.; Riipinen, I.; Rissanen, M.; Rondo, L.; Ruuskanen, T.; Santos, F. D.; Sarnela, N.; Schallhart, S.; Schnitzhofer, R.; Seinfeld, J. H.; Simon, M.; Sipilä, M.; Stozhkov, Y.; Stratmann, F.; Tomé, A.; Tröstl, J.; Tsagkogeorgas, G.; Vaattovaara, P.; Viisanen, Y.; Virtanen, A.; Vrtala, A.; Wagner, P. E.; Weingartner, E.; Wex, H.; Williamson, C.; Wimmer, D.; Ye, P.; Yli-Juuti, T.; Carslaw, K. S.; Kulmala, M.; Curtius, J.; Baltensperger, U.; Worsnop, D. R.; Vehkamäki, H.; Kirkby, J., Molecular understanding of sulphuric acid–amine particle nucleation in the atmosphere. *Nature* **2013,** *502*, (7471), 359-363.
6. Abbatt, J. P. D.; Leaitch, W. R.; Aliabadi, A. A.; Bertram, A. K.; Blanchet, J. P.; Boivin-Rioux, A.; Bozem, H.; Burkart, J.; Chang, R. Y. W.; Charette, J.; Chaubey, J. P.; Christensen, R. J.; Cirisan, A.; Collins, D. B.; Croft, B.; Dionne, J.; Evans, G. J.; Fletcher, C. G.; Gali, M.; Ghahreman, R.; Girard, E.; Gong, W. M.; Gosselin, M.; Gourdal, M.; Hanna, S. J.; Hayashida, H.; Herber, A. B.; Hesaraki, S.; Hoor, P.; Huang, L.; Hussherr, R.; Irish, V. E.; Keita, S. A.; Kodros, J. K.; Kollner, F.; Kolonjari, F.; Kunkel, D.; Ladino, L. A.; Law, K.; Levasseur, M.; Libois, Q.; Liggio, J.; Lizotte, M.; Macdonald, K. M.; Mahmood, R.; Martin, R. V.; Mason, R. H.; Miller, L. A.; Moravek, A.; Mortenson, E.; Mungall, E. L.; Murphy, J. G.; Namazi, M.; Norman, A. L.; O'Neill, N. T.; Pierce, J.

R.; Russell, L. M.; Schneider, J.; Schulz, H.; Sharma, S.; Si, M.; Staebler, R. M.; Steiner, N. S.; Thomas, J. L.; von Salzen, K.; Wentzell, J. J. B.; Willis, M. D.; Wentworth, G. R.; Xu, J. W.; Yakobi-Hancock, J. D., Overview paper: New insights into aerosol and climate in the Arctic. *Atmospheric Chemistry and Physics* **2019,** *19*, (4), 2527-2560.

7. Croft, B.; Martin, R. V.; Leaitch, W. R.; Burkart, J.; Chang, R. Y. W.; Collins, D. B.; Hayes, P. L.; Hodshire, A. L.; Huang, L.; Kodros, J. K.; Moravek, A.; Mungall, E. L.; Murphy, J. G.; Sharma, S.; Tremblay, S.; Wentworth, G. R.; Willis, M. D.; Abbatt, J. P. D.; Pierce, J. R., Arctic marine secondary organic aerosol contributes significantly to summertime particle size distributions in the Canadian Arctic Archipelago. *Atmos. Chem. Phys.* **2019,** *19*, (5), 2787-2812.

8. Willis, M. D.; Burkart, J.; Thomas, J. L.; Köllner, F.; Schneider, J.; Bozem, H.; Hoor, P. M.; Aliabadi, A. A.; Schulz, H.; Herber, A. B.; Leaitch, W. R.; Abbatt, J. P. D., Growth of nucleation mode particles in the summertime Arctic: a case study. *Atmospheric Chemistry and Physics* **2016,** *16*, (12), 7663-7679.

9. Burkart, J.; Hodshire, A. L.; Mungall, E. L.; Pierce, J. R.; Collins, D. B.; Ladino, L. A.; Lee, A. K. Y.; Irish, V.; Wentzell, J. J. B.; Liggio, J.; Papakyriakou, T.; Murphy, J.; Abbatt, J., Organic Condensation and Particle Growth to CCN Sizes in the Summertime Marine Arctic Is Driven by Materials More Semivolatile Than at Continental Sites. *Geophysical Research Letters* **2017,** *44*, (20), 10,725-10,734.

10. Willis, M. D.; Köllner, F.; Burkart, J.; Bozem, H.; Thomas, J. L.; Schneider, J.; Aliabadi, A. A.; Hoor, P. M.; Schulz, H.; Herber, A. B.; Leaitch, W. R.; Abbatt, J. P. D., Evidence for marine biogenic influence on summertime Arctic aerosol. *Geophysical Research Letters* **2017,** *44*, (12), 6460-6470.

11. Schneider, S. R.; Collins, D. B.; Boyer, M.; Chang, R. Y. W.; Gosselin, M.; Irish, V. E.; Miller, L. A.; Abbatt, J. P. D., Abiotic Emission of Volatile Organic Compounds from the Ocean Surface: Relationship to Seawater Composition. *ACS Earth and Space Chemistry* **2024**.

12. Mungall, E. L.; Abbatt, J. P. D.; Wentzell, J. J. B.; Lee, A. K. Y.; Thomas, J. L.; Blais, M.; Gosselin, M.; Miller, L. A.; Papakyriakou, T.; Willis, M. D.; Liggio, J., Microlayer source of oxygenated volatile organic compounds in the summertime marine Arctic boundary layer. *Proceedings of the National Academy of Sciences* **2017,** *114*, (24), 6203-6208.

13. Kilgour, D. B.; Novak, G. A.; Claflin, M. S.; Lerner, B. M.; Bertram, T. H., Production of oxygenated volatile organic compounds from the ozonolysis of coastal seawater. *Atmos. Chem. Phys.* **2024,** *24*, (6), 3729-3742.

14. Abbatt, J. P. D.; Leaitch, W. R.; Aliabadi, A. A.; Bertram, A. K.; Blanchet, J. P.; Boivin-Rioux, A.; Bozem, H.; Burkart, J.; Chang, R. Y. W.; Charette, J.; Chaubey, J. P.; Christensen, R. J.; Cirisan, A.; Collins, D. B.; Croft, B.; Dionne, J.; Evans, G. J.; Fletcher, C. G.; Galí, M.; Ghahreman, R.; Girard, E.; Gong, W.; Gosselin, M.; Gourdal, M.; Hanna, S. J.; Hayashida, H.; Herber, A. B.; Hesaraki, S.; Hoor, P.; Huang, L.; Hussherr, R.; Irish, V. E.; Keita, S. A.; Kodros, J. K.; Köllner, F.; Kolonjari, F.; Kunkel, D.; Ladino, L. A.; Law, K.; Levasseur, M.; Libois, Q.; Liggio, J.; Lizotte, M.; Macdonald, K. M.; Mahmood, R.; Martin, R. V.; Mason, R. H.; Miller, L. A.; Moravek, A.; Mortenson, E.; Mungall, E. L.; Murphy, J. G.; Namazi, M.; Norman, A. L.; O'Neill, N. T.; Pierce, J. R.; Russell, L. M.; Schneider, J.; Schulz, H.; Sharma, S.; Si, M.; Staebler, R. M.; Steiner, N. S.; Thomas, J. L.; von Salzen, K.; Wentzell, J. J. B.; Willis, M. D.; Wentworth, G. R.; Xu, J. W.; Yakobi-Hancock, J. D., Overview paper: New insights into aerosol and climate in the Arctic. *Atmos. Chem. Phys.* **2019,** *19*, (4), 2527-2560.

15. He, X. C.; Abraham, N. L.; Ding, H.; Russo, M. R.; Grosvenor, D. P.; Ge, Y.; Wang, X.; Jones, A. C.; Campuzano-Jost, P.; Nault, B.; Kupc, A.; Blake, D.; Jimenez, J. L.; Williamson, C. J.; Carslaw, K. S.; Weber, J.; Archibald, A. T.; Gordon, H., Evaluation of UKESM aerosol size and composition using ATom measurements indicates missing marine aerosol formation mechanisms. *EGUsphere* **2025,** *2025*, 1-71.

16. Rose, C.; Zha, Q.; Dada, L.; Yan, C.; Lehtipalo, K.; Junninen, H.; Mazon, S. B.; Jokinen, T.; Sarnela, N.; Sipilä, M.; Petäjä, T.; Kerminen, V.-M.; Bianchi, F.; Kulmala, M., Observations of biogenic ion-induced cluster formation in the atmosphere. *Science Advances* **2018,** *4*, (4), eaar5218.

17. Bianchi, F.; Tröstl, J.; Junninen, H.; Frege, C.; Henne, S.; Hoyle, C. R.; Molteni, U.; Herrmann, E.; Adamov, A.; Bukowiecki, N.; Chen, X.; Duplissy, J.; Gysel, M.; Hutterli, M.; Kangasluoma, J.; Kontkanen, J.; Kürten, A.; Manninen, H. E.; Münch, S.; Peräkylä, O.; Petäjä, T.; Rondo, L.; Williamson, C.; Weingartner, E.; Curtius, J.; Worsnop, D. R.; Kulmala, M.; Dommen, J.; Baltensperger, U., New particle formation in the free troposphere: A question of chemistry and timing. *Science* **2016,** *352*, (6289), 1109-1112.

18. Huang, W.; Junninen, H.; Garmash, O.; Lehtipalo, K.; Stolzenburg, D.; Lampilahti, J. L. P.; Ezhova, E.; Schallhart, S.; Rantala, P.; Aliaga, D.; Ahonen, L.; Sulo, J.; Quéléver, L. L. J.; Cai, R.; Alekseychik, P.; Mazon, S. B.; Yao, L.; Blichner, S. M.; Zha, Q.; Mammarella, I.; Kirkby, J.; Kerminen, V.-M.; Worsnop, D. R.; Kulmala, M.; Bianchi, F., Potential pre-industrial–like new particle formation induced by pure biogenic organic vapors in Finnish peatland. *Science Advances* *10*, (14), eadm9191.

19. Kirkby, J.; Duplissy, J.; Sengupta, K.; Frege, C.; Gordon, H.; Williamson, C.; Heinritzi, M.; Simon, M.; Yan, C.; Almeida, J.; Tröstl, J.; Nieminen, T.; Ortega, I. K.; Wagner, R.; Adamov, A.; Amorim, A.; Bernhammer, A.-K.; Bianchi, F.; Breitenlechner, M.; Brilke, S.; Chen, X.; Craven, J.; Dias, A.; Ehrhart, S.; Flagan, R. C.; Franchin, A.; Fuchs, C.; Guida, R.; Hakala, J.; Hoyle, C. R.; Jokinen, T.; Junninen, H.; Kangasluoma, J.; Kim, J.; Krapf, M.; Kürten, A.; Laaksonen, A.; Lehtipalo, K.; Makhmutov, V.; Mathot, S.; Molteni, U.; Onnela, A.; Peräkylä, O.; Piel, F.; Petäjä, T.; Praplan, A. P.; Pringle, K.; Rap, A.; Richards, N. A. D.; Riipinen, I.; Rissanen, M. P.; Rondo, L.; Sarnela, N.; Schobesberger, S.; Scott, C. E.; Seinfeld, J. H.; Sipilä, M.; Steiner, G.; Stozhkov, Y.; Stratmann, F.; Tomé, A.; Virtanen, A.; Vogel, A. L.; Wagner, A. C.; Wagner, P. E.; Weingartner, E.; Wimmer, D.; Winkler, P. M.; Ye, P.; Zhang, X.; Hansel, A.; Dommen, J.; Donahue, N. M.; Worsnop, D. R.; Baltensperger, U.; Kulmala, M.; Carslaw, K. S.; Curtius, J., Ion-induced nucleation of pure biogenic particles. *Nature* **2016,** *533*, (7604), 521-526.

20. Dada, L.; Stolzenburg, D.; Simon, M.; Fischer, L.; Heinritzi, M.; Wang, M.; Xiao, M.; Vogel, A. L.; Ahonen, L.; Amorim, A.; Baalbaki, R.; Baccarini, A.; Baltensperger, U.; Bianchi, F.; Daellenbach, K. R.; DeVivo, J.; Dias, A.; Dommen, J.; Duplissy, J.; Finkenzeller, H.; Hansel, A.; He, X.-C.; Hofbauer, V.; Hoyle, C. R.; Kangasluoma, J.; Kim, C.; Kürten, A.; Kvashnin, A.; Mauldin, R.; Makhmutov, V.; Marten, R.; Mentler, B.; Nie, W.; Petäjä, T.; Quéléver, L. L. J.; Saathoff, H.; Tauber, C.; Tome, A.; Molteni, U.; Volkamer, R.; Wagner, R.; Wagner, A. C.; Wimmer, D.; Winkler, P. M.; Yan, C.; Zha, Q.; Rissanen, M.; Gordon, H.; Curtius, J.; Worsnop, D. R.; Lehtipalo, K.; Donahue, N. M.; Kirkby, J.; El Haddad, I.; Kulmala, M., Role of sesquiterpenes in biogenic new particle formation. *Science Advances 9*, (36), eadi5297.

21. Xiao, M.; Hoyle, C. R.; Dada, L.; Stolzenburg, D.; Kürten, A.; Wang, M.; Lamkaddam, H.; Garmash, O.; Mentler, B.; Molteni, U.; Baccarini, A.; Simon, M.; He, X.-C.; Lehtipalo, K.; Ahonen, L. R.; Baalbaki, R.; Bauer, P. S.; Beck, L.; Bell, D.; Bianchi, F.; Brilke, S.; Chen, D.; Chiu, R.; Dias, A.; Duplissy, J.; Finkenzeller, H.; Gordon, H.; Hofbauer, V.; Kim, C.; Koenig, T. K.; Lampilahti, J.; Lee, C. P.; Li, Z.; Mai, H.; Makhmutov, V.; Manninen, H. E.; Marten, R.; Mathot, S.; Mauldin, R. L.; Nie, W.; Onnela, A.; Partoll, E.; Petäjä, T.; Pfeifer, J.; Pospisilova, V.; Quéléver, L. L. J.; Rissanen, M.; Schobesberger, S.; Schuchmann, S.; Stozhkov, Y.; Tauber, C.; Tham, Y. J.; Tomé, A.; Vazquez-Pufleau, M.; Wagner, A. C.; Wagner, R.; Wang, Y.; Weitz, L.; Wimmer, D.; Wu, Y.; Yan, C.; Ye, P.; Ye, Q.; Zha, Q.; Zhou, X.; Amorim, A.; Carslaw, K.; Curtius, J.; Hansel, A.; Volkamer, R.; Winkler, P. M.; Flagan, R. C.; Kulmala, M.; Worsnop, D. R.; Kirkby, J.; Donahue, N. M.; Baltensperger, U.; El Haddad, I.; Dommen, J., The driving factors of new particle formation and growth in the polluted boundary layer. *Atmospheric Chemistry and Physics* **2021,** *21*, (18), 14275-14291.

22. Riccobono, F.; Schobesberger, S.; Scott, C. E.; Dommen, J.; Ortega, I. K.; Rondo, L.; Almeida, J.; Amorim, A.; Bianchi, F.; Breitenlechner, M.; David, A.; Downard, A.; Dunne, E. M.;

Duplissy, J.; Ehrhart, S.; Flagan, R. C.; Franchin, A.; Hansel, A.; Junninen, H.; Kajos, M.; Keskinen, H.; Kupc, A.; Kürten, A.; Kvashin, A. N.; Laaksonen, A.; Lehtipalo, K.; Makhmutov, V.; Mathot, S.; Nieminen, T.; Onnela, A.; Petäjä, T.; Praplan, A. P.; Santos, F. D.; Schallhart, S.; Seinfeld, J. H.; Sipilä, M.; Spracklen, D. V.; Stozhkov, Y.; Stratmann, F.; Tomé, A.; Tsagkogeorgas, G.; Vaattovaara, P.; Viisanen, Y.; Vrtala, A.; Wagner, P. E.; Weingartner, E.; Wex, H.; Wimmer, D.; Carslaw, K. S.; Curtius, J.; Donahue, N. M.; Kirkby, J.; Kulmala, M.; Worsnop, D. R.; Baltensperger, U., Oxidation Products of Biogenic Emissions Contribute to Nucleation of Atmospheric Particles. *Science* **2014,** *344*, (6185), 717-721.

23. Brean, J.; Bortolussi, F.; Rowell, A.; Beddows, D. C. S.; Weinhold, K.; Mettke, P.; Merkel, M.; Kumar, A.; Barua, S.; Iyer, S.; Karppinen, A.; Sandström, H.; Rinke, P.; Wiedensohler, A.; Pöhlker, M.; Dal Maso, M.; Rissanen, M.; Shi, Z.; Harrison, R. M., Traffic-Emitted Amines Promote New Particle Formation at Roadsides. *ACS ES&T Air* **2025**.

24. Brean, J.; Beddows, D. C. S.; Shi, Z.; Temime-Roussel, B.; Marchand, N.; Querol, X.; Alastuey, A.; Minguillón, M. C.; Harrison, R. M., Molecular insights into new particle formation in Barcelona, Spain. *Atmospheric Chemistry and Physics* **2020,** *20*, (16), 10029-10045.

25. Brean, J.; Dall'Osto, M.; Simó, R.; Shi, Z.; Beddows, D. C. S.; Harrison, R. M., Open ocean and coastal new particle formation from sulfuric acid and amines around the Antarctic Peninsula. *Nature Geoscience* **2021,** *14*, (6), 383-388.

26. Simon, M.; Heinritzi, M.; Herzog, S.; Leiminger, M.; Bianchi, F.; Praplan, A.; Dommen, J.; Curtius, J.; Kürten, A., Detection of dimethylamine in the low pptv range using nitrate chemical ionization atmospheric pressure interface time-of-flight (CI-APi-TOF) mass spectrometry. *Atmos. Meas. Tech.* **2016,** *9*, (5), 2135-2145.

27. Croft, B.; Wentworth, G. R.; Martin, R. V.; Leaitch, W. R.; Murphy, J. G.; Murphy, B. N.; Kodros, J. K.; Abbatt, J. P.; Pierce, J. R., Contribution of Arctic seabird-colony ammonia to atmospheric particles and cloud-albedo radiative effect. *Nat Commun* **2016,** *7*, 13444.

28. O'Donnell, S. E.; Croft, B.; Ford, B.; June, N. A.; Kuang, C.; Singh, A.; Chang, R. Y. W.; Collins, D. R.; Hakala, S.; Jathar, S. H.; Paasonen, P.; Shrivastava, M.; Smith, J. N.; Pierce, J. R., Going Off Grid: A Comparative Study of the Lagrangian and Eulerian Perspectives of New Particle Formation Events. *Journal of Geophysical Research: Atmospheres* **2025,** *130*, (18), e2025JD043713.

29. Donahue, N. M.; Epstein, S. A.; Pandis, S. N.; Robinson, A. L., A two-dimensional volatility basis set: 1. organic-aerosol mixing thermodynamics. *Atmospheric Chemistry and Physics* **2011,** *11*, (7), 3303-3318.

30. Bianchi, F.; Kurten, T.; Riva, M.; Mohr, C.; Rissanen, M. P.; Roldin, P.; Berndt, T.; Crounse, J. D.; Wennberg, P. O.; Mentel, T. F.; Wildt, J.; Junninen, H.; Jokinen, T.; Kulmala, M.; Worsnop, D. R.; Thornton, J. A.; Donahue, N.; Kjaergaard, H. G.; Ehn, M., Highly Oxygenated Organic Molecules (HOM) from Gas-Phase Autoxidation Involving Peroxy Radicals: A Key Contributor to Atmospheric Aerosol. *Chem Rev* **2019,** *119*, (6), 3472-3509.

31. Barua, S.; Kumar, A.; Seal, P.; Iyer, S.; Rissanen, M., Rapid formation of secondary aerosol precursors from the autoxidation of C5–C8 n-aldehydes. *EGUsphere* **2025,** *2025*, 1-25.

32. Wang, Z.; Ehn, M.; Rissanen, M. P.; Garmash, O.; Quelever, L.; Xing, L.; Monge-Palacios, M.; Rantala, P.; Donahue, N. M.; Berndt, T.; Sarathy, S. M., Efficient alkane oxidation under combustion engine and atmospheric conditions. *Commun Chem* **2021,** *4*, (1), 18.

33. Iyer, S.; Rissanen, M. P.; Valiev, R.; Barua, S.; Krechmer, J. E.; Thornton, J.; Ehn, M.; Kurtén, T., Molecular mechanism for rapid autoxidation in α-pinene ozonolysis. *Nature Communications* **2021,** *12*, (1), 878.

34. Wang, Z.; Ehn, M.; Rissanen, M. P.; Garmash, O.; Quélever, L.; Xing, L.; Monge-Palacios, M.; Rantala, P.; Donahue, N. M.; Berndt, T.; Sarathy, S. M., Efficient alkane oxidation under combustion engine and atmospheric conditions. *Communications Chemistry* **2021,** *4*, (1).

35. Rolph, R. J.; Feltham, D. L.; Schröder, D., Changes of the Arctic marginal ice zone during the satellite era. *The Cryosphere* **2020,** *14*, (6), 1971-1984.

36. Strong, C.; Rigor, I. G., Arctic marginal ice zone trending wider in summer and narrower in winter. *Geophysical Research Letters* **2013,** *40*, (18), 4864-4868.

37. Stroeve, J.; Notz, D., Changing state of Arctic sea ice across all seasons. *Environmental Research Letters* **2018,** *13*, (10), 103001.

38. Thomson, J.; Rogers, W. E., Swell and sea in the emerging Arctic Ocean. *Geophysical Research Letters* **2014,** *41*, (9), 3136-3140.

39. Zhang, J.; Lindsay, R.; Schweiger, A.; Steele, M., The impact of an intense summer cyclone on 2012 Arctic sea ice retreat. *Geophysical Research Letters* **2013,** *40*, (4), 720-726.

40. Arrigo, K. R.; Perovich, D. K.; Pickart, R. S.; Brown, Z. W.; van Dijken, G. L.; Lowry, K. E.; Mills, M. M.; Palmer, M. A.; Balch, W. M.; Bahr, F.; Bates, N. R.; Benitez-Nelson, C.; Bowler, B.; Brownlee, E.; Ehn, J. K.; Frey, K. E.; Garley, R.; Laney, S. R.; Lubelczyk, L.; Mathis, J.; Matsuoka, A.; Mitchell, B. G.; Moore, G. W. K.; Ortega-Retuerta, E.; Pal, S.; Polashenski, C. M.; Reynolds, R. A.; Schieber, B.; Sosik, H. M.; Stephens, M.; Swift, J. H., Massive Phytoplankton Blooms Under Arctic Sea Ice. *Science* **2012,** *336*, (6087), 1408-1408.

41. Horvat, C.; Jones, D. R.; Iams, S.; Schroeder, D.; Flocco, D.; Feltham, D., The frequency and extent of sub-ice phytoplankton blooms in the Arctic Ocean. *Science Advances* **2017,** *3*, (3), e1601191.

42. Selivanova, J.; Iovino, D.; Cocetta, F., Past and future of the Arctic sea ice in High-Resolution Model Intercomparison Project (HighResMIP) climate models. *The Cryosphere* **2024,** *18*, (6), 2739-2763.

43. Frew, R. C.; Bateson, A. W.; Feltham, D. L.; Schröder, D., Toward a marginal Arctic sea ice cover: changes to freezing, melting and dynamics. *The Cryosphere* **2025,** *19*, (6), 2115-2132.

44. Mauritsen, T.; Sedlar, J.; Tjernström, M.; Leck, C.; Martin, M.; Shupe, M.; Sjogren, S.; Sierau, B.; Persson, P.; Brooks, I., An Arctic CCN-limited cloud-aerosol regime. *Atmospheric Chemistry and Physics* **2011,** *11*, (1), 165-173.

45. Song, C.; Becagli, S.; Beddows, D. C. S.; Brean, J.; Browse, J.; Dai, Q.; Dall’Osto, M.; Ferracci, V.; Harrison, R. M.; Harris, N.; Li, W.; Jones, A. E.; Kirchgäßner, A.; Kramawijaya, A. G.; Kurganskiy, A.; Lupi, A.; Mazzola, M.; Severi, M.; Traversi, R.; Shi, Z., Understanding Sources and Drivers of Size-Resolved Aerosol in the High Arctic Islands of Svalbard Using a Receptor Model Coupled with Machine Learning. *Environmental Science & Technology* **2022,** *56*, (16), 11189-11198.

46. Brean, J.; Beddows, D. C. S.; Harrison, R. M.; Song, C.; Tunved, P.; Ström, J.; Krejci, R.; Freud, E.; Massling, A.; Skov, H.; Asmi, E.; Lupi, A.; Dall'Osto, M., Collective geographical ecoregions and precursor sources driving Arctic new particle formation. *Atmospheric Chemistry and Physics* **2023,** *23*, (3), 2183-2198.

47. Xavier, C.; de Jonge, R. W.; Jokinen, T.; Beck, L.; Sipila, M.; Olenius, T.; Roldin, P., Role of Iodine-Assisted Aerosol Particle Formation in Antarctica. *Environ Sci Technol* **2024,** *58*, (17), 7314-7324.

48. Dall´Osto, M.; Beddows, D. C. S.; Tunved, P.; Krejci, R.; Ström, J.; Hansson, H. C.; Yoon, Y. J.; Park, K.-T.; Becagli, S.; Udisti, R.; Onasch, T.; O´Dowd, C. D.; Simó, R.; Harrison, R. M., Arctic sea ice melt leads to atmospheric new particle formation. *Scientific Reports* **2017,** *7*, (1), 3318.

49. Dall’Osto, M.; Ovadnevaite, J.; Paglione, M.; Beddows, D. C. S.; Ceburnis, D.; Cree, C.; Cortés, P.; Zamanillo, M.; Nunes, S. O.; Pérez, G. L.; Ortega-Retuerta, E.; Emelianov, M.; Vaqué, D.; Marrasé, C.; Estrada, M.; Sala, M. M.; Vidal, M.; Fitzsimons, M. F.; Beale, R.; Airs, R.; Rinaldi, M.; Decesari, S.; Cristina Facchini, M.; Harrison, R. M.; O’Dowd, C.; Simó, R., Antarctic sea ice region as a source of biogenic organic nitrogen in aerosols. *Scientific Reports* **2017,** *7*, (1), 6047.

50. Dall’Osto, M.; Airs, R. L.; Beale, R.; Cree, C.; Fitzsimons, M. F.; Beddows, D.; Harrison, R. M.; Ceburnis, D.; O’Dowd, C.; Rinaldi, M.; Paglione, M.; Nenes, A.; Decesari, S.; Simó, R., Simultaneous Detection of Alkylamines in the Surface Ocean and Atmosphere of the Antarctic Sympagic Environment. *ACS Earth and Space Chemistry* **2019,** *3*, (5), 854-862.

51. Burkart, J.; Willis, M. D.; Bozem, H.; Thomas, J. L.; Law, K.; Hoor, P.; Aliabadi, A. A.; Köllner, F.; Schneider, J.; Herber, A.; Abbatt, J. P. D.; Leaitch, W. R., Summertime observations of elevated levels of ultrafine particles in the high Arctic marine boundary layer. *Atmospheric Chemistry and Physics* **2017,** *17*, (8), 5515-5535.

52. Taipale, R.; Ruuskanen, T. M.; Rinne, J.; Kajos, M. K.; Hakola, H.; Pohja, T.; Kulmala, M., Technical Note: Quantitative long-term measurements of VOC concentrations by PTR-MS – measurement, calibration, and volume mixing ratio calculation methods. *Atmos. Chem. Phys.* **2008,** *8*, (22), 6681-6698.

53. Qiao, X.; Yan, C.; Li, X.; Guo, Y.; Yin, R.; Deng, C.; Li, C.; Nie, W.; Wang, M.; Cai, R.; Huang, D.; Wang, Z.; Yao, L.; Worsnop, D. R.; Bianchi, F.; Liu, Y.; Donahue, N. M.; Kulmala, M.; Jiang, J., Contribution of Atmospheric Oxygenated Organic Compounds to Particle Growth in an Urban Environment. *Environ Sci Technol* **2021,** *55*, (20), 13646-13656.

54. Brean, J.; Rowell, A.; Beddows, D. C. S.; Weinhold, K.; Mettke, P.; Merkel, M.; Tuch, T.; Rissanen, M.; Maso, M. D.; Kumar, A.; Barua, S.; Iyer, S.; Karppinen, A.; Wiedensohler, A.; Shi, Z.; Harrison, R. M., Road Traffic Emissions Lead to Much Enhanced New Particle Formation through Increased Growth Rates. *Environmental Science & Technology* **2024,** *58*, (24), 10664-10674.

55. Stolzenburg, D.; Sarnela, N.; Bianchi, F.; Cai, J.; Cai, R.; Cheng, Y.; Dada, L.; Donahue, N. M.; Grothe, H.; Holm, S.; Kerminen, V.-M.; Lehtipalo, K.; Petäjä, T.; Sulo, J.; Winkler, P. M.; Yan, C.; Kangasluoma, J.; Kulmala, M., Incomplete mass closure in atmospheric nanoparticle growth. *npj Climate and Atmospheric Science* **2025,** *8*, (1), 75.

56. He, X. C.; Simon, M.; Iyer, S.; Xie, H. B.; Rorup, B.; Shen, J.; Finkenzeller, H.; Stolzenburg, D.; Zhang, R.; Baccarini, A.; Tham, Y. J.; Wang, M.; Amanatidis, S.; Piedehierro, A. A.; Amorim, A.; Baalbaki, R.; Brasseur, Z.; Caudillo, L.; Chu, B.; Dada, L.; Duplissy, J.; El Haddad, I.; Flagan, R. C.; Granzin, M.; Hansel, A.; Heinritzi, M.; Hofbauer, V.; Jokinen, T.; Kemppainen, D.; Kong, W.; Krechmer, J.; Kurten, A.; Lamkaddam, H.; Lopez, B.; Ma, F.; Mahfouz, N. G. A.; Makhmutov, V.; Manninen, H. E.; Marie, G.; Marten, R.; Massabo, D.; Mauldin, R. L.; Mentler, B.; Onnela, A.; Petaja, T.; Pfeifer, J.; Philippov, M.; Ranjithkumar, A.; Rissanen, M. P.; Schobesberger, S.; Scholz, W.; Schulze, B.; Surdu, M.; Thakur, R. C.; Tome, A.; Wagner, A. C.; Wang, D.; Wang, Y.; Weber, S. K.; Welti, A.; Winkler, P. M.; Zauner-Wieczorek, M.; Baltensperger, U.; Curtius, J.; Kurten, T.; Worsnop, D. R.; Volkamer, R.; Lehtipalo, K.; Kirkby, J.; Donahue, N. M.; Sipila, M.; Kulmala, M., Iodine oxoacids enhance nucleation of sulfuric acid particles in the atmosphere. *Science* **2023,** *382*, (6676), 1308-1314.

57. Myllys, N.; Chee, S.; Olenius, T.; Lawler, M.; Smith, J., Molecular-Level Understanding of Synergistic Effects in Sulfuric Acid–Amine–Ammonia Mixed Clusters. *The Journal of Physical Chemistry A* **2019,** *123*, (12), 2420-2425.

58. Hasan, G.; Wu, H.; Knattrup, Y.; Elm, J., Base synergy in freshly nucleated particles. *Aerosol Research* **2025,** *3*, (1), 101-111.

59. Curtius, J.; Heinritzi, M.; Beck, L. J.; Pöhlker, M. L.; Tripathi, N.; Krumm, B. E.; Holzbeck, P.; Nussbaumer, C. M.; Hernández Pardo, L.; Klimach, T.; Barmpounis, K.; Andersen, S. T.; Bardakov, R.; Bohn, B.; Cecchini, M. A.; Chaboureau, J.-P.; Dauhut, T.; Dienhart, D.; Dörich, R.; Edtbauer, A.; Giez, A.; Hartmann, A.; Holanda, B. A.; Joppe, P.; Kaiser, K.; Keber, T.; Klebach, H.; Krüger, O. O.; Kürten, A.; Mallaun, C.; Marno, D.; Martinez, M.; Monteiro, C.; Nelson, C.; Ort, L.; Raj, S. S.; Richter, S.; Ringsdorf, A.; Rocha, F.; Simon, M.; Sreekumar, S.; Tsokankunku, A.; Unfer, G. R.; Valenti, I. D.; Wang, N.; Zahn, A.; Zauner-Wieczorek, M.; Albrecht, R. I.; Andreae, M. O.; Artaxo, P.; Crowley, J. N.; Fischer, H.; Harder, H.; Herdies, D. L.; Machado, L. A. T.; Pöhlker, C.; Pöschl, U.; Possner, A.; Pozzer, A.; Schneider, J.; Williams, J.; Lelieveld, J., Isoprene nitrates drive new particle formation in Amazon's upper troposphere. *Nature* **2024,** *636*, (8041), 124-130.

60. Baccarini, A.; Karlsson, L.; Dommen, J.; Duplessis, P.; Vullers, J.; Brooks, I. M.; Saiz-Lopez, A.; Salter, M.; Tjernstrom, M.; Baltensperger, U.; Zieger, P.; Schmale, J., Frequent new

particle formation over the high Arctic pack ice by enhanced iodine emissions. *Nat Commun* **2020,** *11*, (1), 4924.

61. Trostl, J.; Chuang, W. K.; Gordon, H.; Heinritzi, M.; Yan, C.; Molteni, U.; Ahlm, L.; Frege, C.; Bianchi, F.; Wagner, R.; Simon, M.; Lehtipalo, K.; Williamson, C.; Craven, J. S.; Duplissy, J.; Adamov, A.; Almeida, J.; Bernhammer, A. K.; Breitenlechner, M.; Brilke, S.; Dias, A.; Ehrhart, S.; Flagan, R. C.; Franchin, A.; Fuchs, C.; Guida, R.; Gysel, M.; Hansel, A.; Hoyle, C. R.; Jokinen, T.; Junninen, H.; Kangasluoma, J.; Keskinen, H.; Kim, J.; Krapf, M.; Kurten, A.; Laaksonen, A.; Lawler, M.; Leiminger, M.; Mathot, S.; Mohler, O.; Nieminen, T.; Onnela, A.; Petaja, T.; Piel, F. M.; Miettinen, P.; Rissanen, M. P.; Rondo, L.; Sarnela, N.; Schobesberger, S.; Sengupta, K.; Sipila, M.; Smith, J. N.; Steiner, G.; Tome, A.; Virtanen, A.; Wagner, A. C.; Weingartner, E.; Wimmer, D.; Winkler, P. M.; Ye, P.; Carslaw, K. S.; Curtius, J.; Dommen, J.; Kirkby, J.; Kulmala, M.; Riipinen, I.; Worsnop, D. R.; Donahue, N. M.; Baltensperger, U., The role of low-volatility organic compounds in initial particle growth in the atmosphere. *Nature* **2016,** *533*, (7604), 527-31.

62. Yli-Juuti, T.; Nieminen, T.; Hirsikko, A.; Aalto, P. P.; Asmi, E.; Hõrrak, U.; Manninen, H. E.; Patokoski, J.; Dal Maso, M.; Petäjä, T.; Rinne, J.; Kulmala, M.; Riipinen, I., Growth rates of nucleation mode particles in Hyytiälä during 2003−2009: variation with particle size, season, data analysis method and ambient conditions. *Atmos. Chem. Phys.* **2011,** *11*, (24), 12865-12886.

63. Boyer, M.; Quéléver, L.; Brasseur, Z.; McManus, B.; Herndon, S.; Agnese, M.; Nelson, D.; Roscioli, J.; Weis, F.; Sel, S.; Marincovich, G. L.; Quarin, F. J.; Buchholz, A.; Xavier, C.; Perchivale, P. J.; Kerminen, V.-M.; Kulmala, M.; Petäjä, T.; He, X.-C.; Sofieva-Rios, S.; Timonen, H.; Aurela, M.; Barreira, L.; Virkkula, A.; Asmi, E.; Worsnop, D.; Sipilä, M., Penguin guano is an important source of climate-relevant aerosol particles in Antarctica. *Communications Earth & Environment* **2025,** *6*, (1), 368.

Du et al., Response to reviewer comments

We have thoroughly reviewed all comments, provided a point-by-point response, and revised the manuscript accordingly. Below, we include the reviewers' comments in black, our responses in blue, and any manuscript text in orange. Where edits are made to a larger section, we will include the full section.

Please note that we have re-formatted the manuscript based on *Nature Geoscience* guidance. Ten of the SI figures have been moved to the main text as *Extended Data figures*, and we have reduced our Methods section from 7,500 words to roughly 500, relegating most of this information to the Supplementary Information. Supplementary Information in the original main manuscript has also been moved to form a separate document "*Supplementary Information figures and tables*" that includes detailed methods, supplementary texts, and supplementary figures/tables.

Reviewer #1:

I have already reviewed the first version of this manuscript, which was originally submitted to Nature. Many thanks to the authors for their careful and comprehensive revisions. The manuscript has improved significantly as a result, and I have no further substantial comments. The manuscript presents data on new particle formation during an Arctic expedition, thus representing an understudied but important region for regional climate change. The interpretation is comprehensive and it is now appropriately cautious. I recommend publication in Nature Geoscience.

General Response: We would like to thank the reviewer for the recognition of our efforts in addressing reviewer comments to make our interpretation comprehensive and cautious.

Technical comments:

(line numbers refer to file 50911_1_supp_462901_t81ys0_convrt.pdf, manuscript with track changes included).

l. 50, enhancing --> enhance

Response: We have revised it.

1. 101: consequent --> subsequent

Response: We have revised it.

l. 101: growth to Aitken mode size aerosol --> growth to aerosol sizes that range in the Aitken mode

Response: We have revised it.

1. 102: what do you mean when stating “many of which have crossover with the diameter of growing particles”?

Response: To avoid confusion, the sentence has changed into below in the manuscript:

‘Baccarini et al. [14] determined the size distribution of cloud residuals, many of which are similar in diameter to particles from iodine-driven NPF, and therefore inferred that iodine-driven NPF has the potential to influence CCN and cloud properties.’

l. 132-133: “on all NPF (8 days), burst (5 days), and non-event (8 days) event days” --> “on all NPF event (8 days), burst event (5 days), and non-event (8 days) days”

Response: We have revised it.

l. 196: clustered-clustered forms --> clustered forms

Response: We have revised it.

1. 210 “solid red line” --> “dashed cyan line” ?

Response: We have amended it to ‘dashed red line’ in the manuscript, as the line is now red.

l. 211 and l. 214., not useful to print the same fitting equation twice

Response: We have deleted the second fitting equation.

l. 224: what is meant by “daily hourly…”

Response: We have changed it into:

‘The peak hourly formation rate at 1.7 nm (J1.7) for each day...’

l. 230 Fig S8A says1-2 ppb NH_3, here you state 10 ppb NH_3. Please correct. Also, you sometimes use “pptv” and “ppbv”, and sometimes “ppt” and “ppb”. Please use one unit consistently throughout.

Response: Thank you, we amend to 1-2 pptv NH_3 in the manuscript. We now use ppt**v** and ppb**v** in the entire manuscript.

l. 342: “MIZ”. Please explain the acronym (or omit the acronym as it is not used in the rest of the paper.

Response: we have changed MIZ to “marginal ice zone” across the manuscript. This is defined as the zone with sea ice concentration between 10% and 80%.

l. 343: Do not start a sentence with ">90 %" --> "). More than 90%"

Response: We have revised it.

l. 363: flew --> flowed

Response: We have revised it.

l. 367-371. Long convoluted sentence, please simplify…

Response: The sentence has changed into below:

'This faster growth at larger sizes is consistent with the phenomenon observed by Burkart et al. [22] in the Canadian Arctic Archipelago, which they hypothesized as being driven by semi-volatile organic compounds. Our chemical observations confirm that as particles grow larger, LVOCs contribute more to growth than ELVOCs (Fig. 4D).'

l. 377 previous --> previously

Response: We have revised it.

l. 379 direct ambient observational evidence --> direct observational evidence

Response: We have revised it.

l. 445 flew --> flowed

Response: We have revised it.

l. 447 marginal sea ice zone --> the marginal sea ice zone

Response: We have revised it.

l. 451-452 repetition of "likely ubiquitous"

Response: We have deleted the second one.

l. 457 bird nests --> bird colonies

Response: We have revised it.

l. 458 central ocean --> open ocean

Response: We have revised it.

Reviewer #6:

Du et al. report comprehensive shipborne measurements of new particle and CCN formation processes in the Arctic, building a critical link between earlier laboratory studies and ambient observations. The primary novelty of this work lies in the full coverage of NPF processes up to CCN sizes, including direct CCN detection, which distinguishes it from many previous studies. This study also validates the iodine-sulfur synergy in aerosol formation proposed by He et al. (2023) within realistic ambient conditions, confirming the relevance of this laboratory-based nucleation mechanism. Furthermore, the authors provide solid results indicating that OOMs, likely derived from aldehydes, which dominate particle growth processes. Additionally, a new category of OOMs, namely I-OOMs, is identified for the first time and proposed to contribute to particle growth, albeit to a lesser extent.

In my view, while each of these advances is valuable, it is not immediately clear that any single contribution alone necessitates publication in Nature Geoscience. The authors appear to recognize this, as the manuscript endeavors to articulate various aspects of novelty relative to existing literature. However, the strategy of aggregating these subtle novelties occasionally detracts from the central narrative. Consequently, the current results do not yet appear sufficient to support several key conclusions - points which seemingly reflect the challenges raised during the initial review process by other reviewers.

Specifically, the manuscript attempts to distinguish this work from previous studies, such as investigations by Beck et al. (2021) and Baccarini et al. (2020), by characterizing them as regional studies to reflect the complexity of specific Arctic environments that cannot be easily extrapolated. However, it effectively remains that the current work is also a regional study, with an observational timeframe that is shorter than many preceding campaigns. This therefore puts a large weight on trying to generalize these results to marginal sea ice zones, but the current justification remains insufficient.

As a reviewer invited for the second round, I initially evaluated the updated manuscript without consulting the previous reviews or response letters. Upon subsequently reviewing the history, I noted that my independent observations largely echoed concerns previously raised by other reviewers. This suggests that the revision has not yet sufficiently addressed the fundamental issues required to clearly improve the manuscript.

Despite the critical nature of the comments above and the detailed discussion below, I consider this study to be an important contribution to our understanding of Arctic aerosol formation processes. Many of the raised items are something that can be addressed by the authors, and I believe the work has the potential to meet the standards of Nature Geoscience provided these issues are thoroughly addressed. I encourage the authors to consider these points, after which I would be willing to reevaluate the manuscript.

Response: We would like to thank the reviewer for succinctly outlining the four key results from this study and for the recognition of this study as an important contribution to our understanding of Arctic aerosol formation processes.

We resolve a major observational gap by measuring the whole process of how particles form and grow into CCN. This study integrates the fundamental stages of nucleation and growth with the final formation of CCN, ensuring these are presented as a single, connected causal chain rather than isolated events. While we discuss novel findings in detail (such as the first identification of I-OOMs), we contextualise them within the overall atmospheric lifecycle described in this paper.

In the following, we provided a point-by-point response to all comments, including those mentioned above, and revised the manuscript accordingly.

Major comments:

Comment 1: A significant oversight in the present analysis, which was also not addressed in the previous round of review, is the effect of the condensation sink (CS) - and more specifically the associated coagulation sink - on new particle formation rates. The authors compare their observed nucleation rates with mechanisms derived from the CERN CLOUD experiments, identifying the HIOx-H2SO4 mechanism presented by He et al. (2023) as the best fit. However, the measured values remain roughly an order of magnitude higher than those reported by He et al. (2023). To reconcile this discrepancy, the authors appear to seek explanations involving other mechanisms, such as enhancement by DMA and OOMs. However, as reported in this version of the manuscript, the CS during the cruise expedition was approximately 3-4 times lower than in the CLOUD experiments. Consequently, based on the same HIOx-H2SO4 nucleation mechanism, higher nucleation rates should indeed be expected in the expedition data due to reduced scavenging losses. For example, Zhang et al. (2022; 10.1021/acs.est.2c04328) calculated the impact of CS on the nucleation rate of the iodine oxoacid system. With 1e6 of HIO3 (and fixed 4.32e5 HIO2), at typical CLOUD CS conditions (0.002 s-1), the nucleation rate is approximately 0.2 cm-3 s-1 (Figure 4 of their study). Conversely, if the CS is reduced to 0.0002 s-1 under the same vapor conditions (Figure S2 of their study), the rate increases to around 40 cm-3 s-1. Although Zhang et al. investigated the pure HIOx system, it is mechanistically a simplified version of the HIOx-H2SO4 system, suggesting that these quantitative trends are likely applicable here as well. Therefore, it is not surprising that the present study measures rates an order of magnitude higher than the He et al. (2023) fit. On the contrary, when accounting for the differences in sinks, this likely represents a surprisingly good agreement. I believe a major revision is needed in this study to reflect this (e.g., lines 227-234; 235-240 etc); I believe this has the potential to sharpen the message of this study as well.

Response: We thank the reviewer for highlighting the important role of pre-existing aerosols. We agree that the J value is sensitive to the background aerosol (beyond what is already accounted for in the calculation). As demonstrated by Zhang et al. (2022), at any given precursor concentration the calculated nucleation rates at a lower CS (e.g., Arctic air, 0.0003 s^{-1}) will be

higher than those at a higher CS (e.g., CLOUD chamber, 0.002 s^{-1}) because the CS governs not only monomer concentrations (already accounted for on our x-axis) and coagulational losses of newly formed particles (already accounted for on our y-axis), but also the scavenging of intermediate clusters during their growth to detectable sizes. This intermediate cluster scavenging is not explicitly captured in our analysis of either the ambient or CLOUD data, and differences in CS between the two could therefore introduce meaningful differences in J that are not reflected in a direct comparison.

We therefore agree with the reviewer that accounting for CS differences would likely bring our observations into better agreement with He et al. (2023) than a direct comparison implies. We also acknowledge that we cannot make any corrections with appropriate precision, given remaining uncertainties the processes of cluster loss to preexisting aerosols. To address this comment, we revised our manuscript to account for this factor (line 237 – manuscript with tracked changes):

"Following He et al. [17], we plotted $J_{1.7}$ versus (HIO_3 + H_2SO_4) × HIO_2, representing the formation rate of H_2SO_4-HIO_2 and HIO_3-HIO_2 (Fig. 3B). ~~**While** our $J_{1.7}$ measurements are comparable with those observed for the HIO_x-H_2SO_4 and HIO_x-H_2SO_4-NH_3 in CLOUD chamber experiments at -10 °C within uncertainties for most days, **the central values of our observations sit slightly what we would anticipate based on CLOUD**.~~ Of all systems that have been studied at the CLOUD chamber, the HIO_x-H_2SO_4 system most consistently reproduces our $J_{1.7}$ values, indicating the key role of both HIO_x and H_2SO_4 in our observed nucleation events. Considering the average ambient temperature of 1.4 °C during nucleation periods, the central value of our $J_{1.7}$ values on some days **sit slightly above our interpolated fitting line based on** ~~are higher than anticipated from~~ the temperature dependence measured in the CLOUD chamber [17] (at -10 and +10 °C). **This enhancement is likely driven by two key factors: reduced cluster scavenging and** the potential participation of other stabilising species. **Firstly, our condensation sink was lower than in the CLOUD chamber, which can cause deviations due to differences in cluster scavenging [37].** Second is the potential participation of other stabilising species. One plausible contributor is DMA, as evidenced by the occasional detection of DMA clustered with HIO_x (Fig. S4 and Extended Data Figures 3-4). The DMA-HIO_2-HIO_3 cluster signal correlates with HIO_3 concentrations and is highest when HIO_3 is at its maximum (Figs. S4-5). Quantum chemical modelling studies also showed that DMA can accept a proton from HIO_3 as a base, forming a stable cluster (DMA-HIO_2-HIO_3) [38]. OOMs may also enhance nucleation rates [39], however, we see no positive relationship between OOM concentrations (of any volatility class or source) and enhanced nucleation rates relative to He et al. [17] (Fig. S6). This indicates that the OOMs we observed do not appear to have a substantial enhancing effect."

Where ref. 37 is Li et al. (https://doi.org/10.1039/D2EA00066K)

Comment 2: Potentially related to my previous major comment, I find it difficult to understand the authors' insistence on positioning the DMA contribution to aerosol nucleation as a key element, particularly given the controversies raised by Reviewers 1, 2, 3, and 4 previously, as well as my own concerns detailed below. Compared to other conclusions in the manuscript, the assertion that DMA is a major player in aerosol nucleation appears fundamentally flawed. The primary evidence supporting the role of DMA is the detection of C2H7N-HIO2-IO3- (Fig. S5). This relies on a single peak which is not unambiguously detected in many events. Unlike the other four peaks discussed - which have periods where they clearly dominate the spectra - this specific DMA cluster is, in most cases, fitted from residual signals, except the short period where the peak is identified (Fig. S9). In my view, it is impossible to use this as concrete evidence for identifying NPF mechanisms, particularly not quantitatively. At maximum, this is a sporadic event that is not regionally important. All previous studies cited in this work (e.g., Almeida et al., 2013; Yao et al., 2018; Xiao et al., 2021) show multiple unambiguous DMA-containing clusters when DMA enhancement is significant. It is also noteworthy that the authors did not measure DMA concentrations but instead measured this cluster, which is uncommon, as cluster concentrations (especially for this trimer) are typically lower than monomer concentrations during NPF events.

Additionally, in the response materials (e.g., Comment 2 of Reviewers 1 & 2), the authors combined AMS mass/volume identification with growth inverse modeling to estimate the gas-phase concentration of DMA, concluding that DMA+TMA might be around 1.8 pptv. I find the identification of C2H7N and C3H9N in Figure R1 unclear and the quantitative assignment of their concentrations challenging (even worse than the gas phase identification). Furthermore, one cannot apply growth modeling developed for condensable vapors (like organics and H2SO4) to amines in this manner. Because amines are not directly condensable vapors (often requiring acid-base stabilization), their accommodation coefficient is highly uncertain; simply relying on volatility ranges for this calculation is inaccurate and methodologically unsound.

Finally, if ~2 pptv of DMA were indeed present in the field observations (levels comparable to urban environments), one would expect to see significantly more DMA-containing clusters and a much faster nucleation rate than currently reported. The observed rates should be comparable than the Xiao et al. (2021) data (Figure S8) and the Kürten et al. (2018) data - which should be included in Figure S8 for reference. Once again, considering the much lower CS in the Arctic compared with CLOUD, especially during the DMA experiments, if DMA were important, the rates in the field should be significantly higher than Xiao et al. and Kürten et al. The current data contradicts this expectation.

Overall, the slight modification acknowledging that DMA enhancement is merely "potential" (lines 42–43) is insufficient to address the collective concerns of the reviewers, especially since this is still proposed as a major advance of the study (lines 227–234 and Fig. 5). I do not believe the DMA enhancement arguments are solid, and the heavy focus on them seems counterproductive (there are over 20 mentioning of DMA in the manuscript). It makes the manuscript vulnerable by stretching to propose a "new" mechanism not supported by robust evidence. I strongly suggest the

authors reevaluate their data and avoid making overly strong claims regarding DMA enhancement, unless they have additional, much more solid evidence.

Response: We thank the reviewer for their detailed consideration of the role of dimethylamine. We have revised our manuscript as shown in the following amendments, which are made alongside response to comment 1 above and updates on the potential role of OOMs in response to Comment 6. Thanks to the helpful comments by yourself and Reviewer 7, we have conducted an extended error analysis. This enabled us to discuss the nucleation mechanisms in light of this error analysis, which is presented in response to Comment 8 below,

We recognise that there are different views on how strongly our observations related to DMA can be interpreted. However, we agree that the role of DMA in nucleation, including its contribution to the nucleation rate, cannot be considered conclusive on the basis of the present data alone, particularly in the presence of HIOx, and any inference drawn by working backwards from AMS measurements is inherently uncertain.

What we can confirm is that the DMA cluster was unambiguously identified during the period shown in Fig. S4, which led us to conclude that DMA and this cluster were present at least during that time. We could not identify this cluster unambiguously in other periods because of interference from nearby I-OOM signals. The limit of detection for a cluster is, we believe, slightly lower than for amine-$(HNO_3)_{1-2}NO_3^-$, which may explain why we did not detect amines.

We understand the reasoning for caution in describing the role of DMA enhancement and revised our manuscript accordingly.

In the abstract:

Our results validate the recent laboratory-based multi-component nucleation mechanism involving iodine oxoacids and sulfuric acid ~~and expands this by showing the potential role of amines in this nucleation mechanism~~

In “multicomponent nucleation”:

~~Neutral~~ Clusters containing H2SO4, HIO3, **and** HIO2~~, and dimethylamine (DMA)~~ were detected during nucleation events (Figs. 2A and B, Table S2), **including** ~~Notably,~~ the dimers HIO3-HSO4- and HIO2-HIO3-NO3-, and **occasional detection of** the trimers HIO2-H2SO4-HSO4⁻ and C2H7N-HIO2-IO3- ~~were only identified~~ during nucleation events. ~~**Furthermore, a cluster containing dimethylamine (DMA), specifically the trimer DMA-HIO2-IO3- was sporadically identified during these events.**~~

…

Considering the average ambient temperature of 1.4 ℃ during nucleation periods, the **central value of our** ~~observed~~ $J_{1.7}$ values **on some days** ~~**appear to be**~~ **are** higher than anticipated from the

temperature dependence measured in the CLOUD chamber [17] (at -10 and +10 ℃). **This enhancement is likely driven by two key factors: reduced cluster scavenging and** the potential participation of other stabilising species. **Firstly, our condensation sink was lower than in the CLOUD chamber, which can cause deviations due to differences in cluster scavenging [37].** Second is the potential **participation** of other stabilising species. One plausible contributor is DMA, as evidenced by the occasional detection of DMA clustered with HIO_x (Fig. S4 and Extended Data Figure 3-4). The DMA-HIO_2-HIO_3 cluster signal…

In "Atmospheric implications"

… Updating the core nucleation mechanisms within models to include the synergistic interactions between H_2SO_4 and HIO_3, ~~and key stabilising species including DMA~~ , **and key stabilising organic and inorganic species**, supported by laboratory kinetic studies, would better represent the real world.

In Supplementary Information 1.1

We also detected a DMA-containing cluster unambiguously during a short period of time in this campaign, **indicating that amines are present at low concentrations. Nonetheless, its presence indicates that trace amines ~~can~~ may participate in the clustering process in this region**. Note that DMA is capable of enhancing nucleation rates at sub-pptv concentrations [108]. Finally, we observe high concentrations of OOMs, which have been shown to be capable of enhancing the nucleation rate of sulfuric acid clusters [32,108]. Our analysis did not provide concrete evidence to support or refute their role in nucleation. ~~These results led us to hypothesize that DMA and OOMs may also contribute to nucleation in the study region.~~

Quantifying the relative contributions of DMA and HIOx to nucleation would require a dedicated chamber study beyond the scope of the present work.

Regarding our "2 pptv" calculations, we agree, and meant for this only as a rough, back of the envelope calculation. We agree that such calculations come with large uncertainty and will forego this analysis.

Comment 3: Throughout the response documents and the introduction, the authors imply that earlier studies (e.g., Baccarini et al., Beck et al.) are limited by their specific regional and temporal scope, potentially lacking representativeness for the wider Arctic. Conversely, they imply the current study as more representative (lines 101–107) and suitable for predicting the future Arctic. However, this distinction appears overstated given that the present study is similarly constrained to a specific region during a specific season (May–June) for approximately one month. It is not immediately clear that the geographical and temporal coverage here represents a significant advancement over earlier studies in this specific regard.

The authors attempt to associate certain NPF events (e.g., lines 304 onwards) with the marginal sea ice zone and subsequently extrapolate these findings to claim relevance for the entire Arctic (lines 400–408). As discussed in detail by Reviewers 3 and 4, such extrapolation remains tenuous. Furthermore, it is unclear why the limitations cited for Beck et al. (2021) and Baccarini et al. (2020) would not apply here, or conversely, why those studies could not make similar extrapolations using the same logic.

It is likely that the mechanisms proposed in this study and those identified in earlier works coexist, with dominance depending on location and season. However, a genuine distinction and strength of this work lies in the validation of the iodine-sulfur synergy proposed by He et al. (2023). Unlike earlier studies that lacked evidence for this specific interaction, the current measurements provide crucial validation for this mechanism in ambient conditions. Therefore, the discussion should be repositioned to more accurately reflect the study's scope, rather than framing shared methodological limitations as distinct advantages over prior work.

Response: We thank the reviewer for raising important points regarding how our findings fit within the broader context of Arctic research. We agree that the distinction of our work lies in the validation of the mechanism of He et al. (2023). We also agree that the mechanisms identified in earlier works likely coexist with the pathway we describe, with their relative importance dictated by local precursor availability.

To correct the unintended implication that our study is more regionally representative than works like Beck et al. (2021) and Baccarini et al. (2020), we have revised the Introduction (former lines 101–107). We now explicitly frame the value of our study and those before it as advancing *process understanding*. The revised text reads:

"However, no studies have yet followed the same particle population from molecular cluster formation through growth to directly measured CCN at the molecular level ~~(Supplementary Text 1.3). More comprehensive and spatially resolved measurements are essential to generate an accurate understanding of Arctic regions. All this~~. **A better understanding of NPF processes, from nucleation to growth of new particles and CCN formation** is important for predicting changing Arctic climate into the future, where, for example, the environmental conditions are changing and emissions of gas-phase iodine and dimethylsulfide (DMS) - the major marine biogenic sulfur compound for aerosol formation, are increasing [12,26-29]. "

To respond to the concern regarding "extrapolation" to the wider Arctic, we have revised our "Sources and Processes" section to anchor our new results in the context of literature, acknowledging specifically that the relative importance of different mechanisms identified so far in literature may depend on season and location (and thus precursor availability). There is strong evidence that melting ice zone is a precursor hotspot, and if this is the case across other parts of the Arctic, we hypothesize that changes in the melting ice zone (timing and location) due to

warming Arctic can have implications for NPF and CCN formation. Please see also "Response to Comment 4 below".

Finally, to explicitly delineate the boundaries of our claims and appropriately contextualise our findings against prior literature, we point the reviewer to Supplementary Text 1 (*Advancing the Understanding of Arctic NPF: Context and Future Directions*). This section acts to frame our study as a vital complement to existing literature, rather than a replacement:

- Section 1.1 maps out currently understood nucleation mechanisms, clarifying how our observation of a synergistic iodine-sulfur pathway complements the separate pathways identified by Beck et al. and Baccarini et al.
- Sections 1.2 and 1.3 contextualise our findings on particle growth, highlighting that our primary novelty is the continuous molecular-level tracking of a single particle population.
- Section 1.4 explicitly acknowledges the spatial and temporal limitations of current expeditionary data (including our own) and outlines the remaining gaps that require targeted future campaigns and regional modelling (e.g., resolving spatially resolved precursor emission fluxes, the role of ULVOCs/amines, and the ultimate CCN budget).

We believe that Supplementary Text 1 provides the contextual framing.

Comment 4: The section titled "Sources and processes influencing high latitude new particle and CCN formation" reads more like a literature review than a synthesis of results from the present study. It relies heavily on findings from earlier studies; for example, it puts MSA and NH3 as key species in the particle formation events, even though this study provides limited data on these compounds. While it is scientifically plausible - based on the broader literature - that Arctic NPF involves these species, the mechanisms currently depicted in the figure are sufficiently generic that they could describe NPF events in many environments globally, rather than highlighting the unique characteristics of the events observed here. Consequently, the utility of this schematic is limited. I suggest that the authors significantly streamline this section and revise Figure 5 to focus primarily on the direct findings of this study, rather than presenting a generalized overview of potential pathways.

Response: We understand the concern that incorporating findings from earlier studies might make the schematic appear broad.

Furthermore, we have streamlined the text in the "Sources and processes" section. We have restructured the paragraphs to lead with our direct measurements, ensuring the distinction between our novel contributions and the contextual literature.

Our measurements demonstrate that HIO_x and H_2SO_4 synergistically drive nucleation during our campaign. Subsequent particle growth to cloud condensation nuclei (CCN) is propelled by a

range of OOMs, including I-OOMs. We observed nucleation on over 80% of days when peak solar radiation exceeded 600 W m^{-2}, suggesting that this process is widespread across the region, which could have been missed if not with sub-5 nm particle observations. The precursor vapours emerge from diverse natural sources (Figure 5), including open ocean, ice, melting ice zone and coast.

HIO_x and H_2SO_4 primarily originate from the oxidation of DMS and iodine-containing species (e.g., I_2 and C_xH_yIz). Elevated concentrations of both species were observed near the marginal ice zone, particularly when air masses were exposed to the ice edge for extended periods, identifying this region as a key hotspot. This enhancement is likely driven by combined emissions from open leads, intensified biological activity, and active photochemistry [48,49]. Consistent with this, thriving under-ice algae and elevated oceanic chlorophyll-a were observed (Fig. S10), which are established sources of DMS, and therefore H_2SO_4 and MSA (Fig. S11) [11,50,51].

The highest HIO_x concentrations occurred in coastal Greenland, including fjord regions, indicating strong local iodine sources. In addition to the marginal ice zone, the open ocean represents a broader source of both DMS and iodine-containing species [50,52], although emission fluxes in this study region remain unconstrained. Iodine emissions may also arise from ice and snow surfaces through photochemical recycling of deposited iodine [48,49].

Previous Arctic studies have identified H_2SO_4-NH_3 and HIO_x nucleation as key processes, before the discovery of the synergistic behaviour of HIO_x-H_2SO_4. However, the detection of HIO_x and H_2SO_4 in these studies [14,53] and mixed iodine-sulfur clusters suggests that the HIO_x-H_2SO_4 nucleation process may have been present in their observations. The relative importance of different nucleation mechanisms in the wider Arctic likely depends on season and location (Fig. S1) [54], which controls emission and oxidation processes, and thus the concentration of precursor gases (such as DMS and I_2, Fig. 5) [39]. To better quantify nucleation mechanisms across the Arctic, comprehensive observations at multiple platforms and locations are needed.

The OOMs and I-OOMs that drive growth of new particles are formed from oxidation of VOCs and OVOCs which were observed at highest concentrations when air masses flowed over the ice edge. Aldehyde-OOMs originate from aldehydes, which are formed by oxidation of fatty acids in the sea-surface microlayer [42,55]. Monoterpenes are emitted from the ocean through diffusion, although at lower rates than from most land regions [56,57]. Both aldehydes and monoterpenes readily undergo autoxidation [39,58] to form the OOMs required for particle growth. We propose that I-OOMs form through regular autoxidation, with iodine-containing radicals serving as autooxidation terminator (see details in Supplementary Text 5). The strong correlations between HIO3 and I-OOMs (Fig. S5) indicate they share similar sources.

Like nucleation precursors, the growth of new particles is dependent on season and location, which dictates the availability of VOCs and oVOCs and thus OOMs. While the ice-covered higher Arctic, for instance, over the central Arctic pack ice region has high abundance of iodine

oxides [14], VOCs and thus OOMs concentrations appear to be low (Supplementary Text 1), leading to limited new particle growth and CCN formation [7,10,13,14]. On the other hand, OOMs seem to be more abundant over the Arctic and sub-Arctic seas and marginal ice zone, resulting in faster growth of new particles (Fig. 4 and Extended Data Fig. 9) and contributing to CCN formation (Fig. 5, Supplementary Text 1).

We agree with the reviewer that our direct findings need to take centre stage so that the figure does not read as a generic global schematic. To resolve this, we have revised Figure 5, focusing on the dominant pathways and species linked to NPF in this study (such as the synergistic HIOx-H2SO4 pathway, I-OOMs, and OOMs).

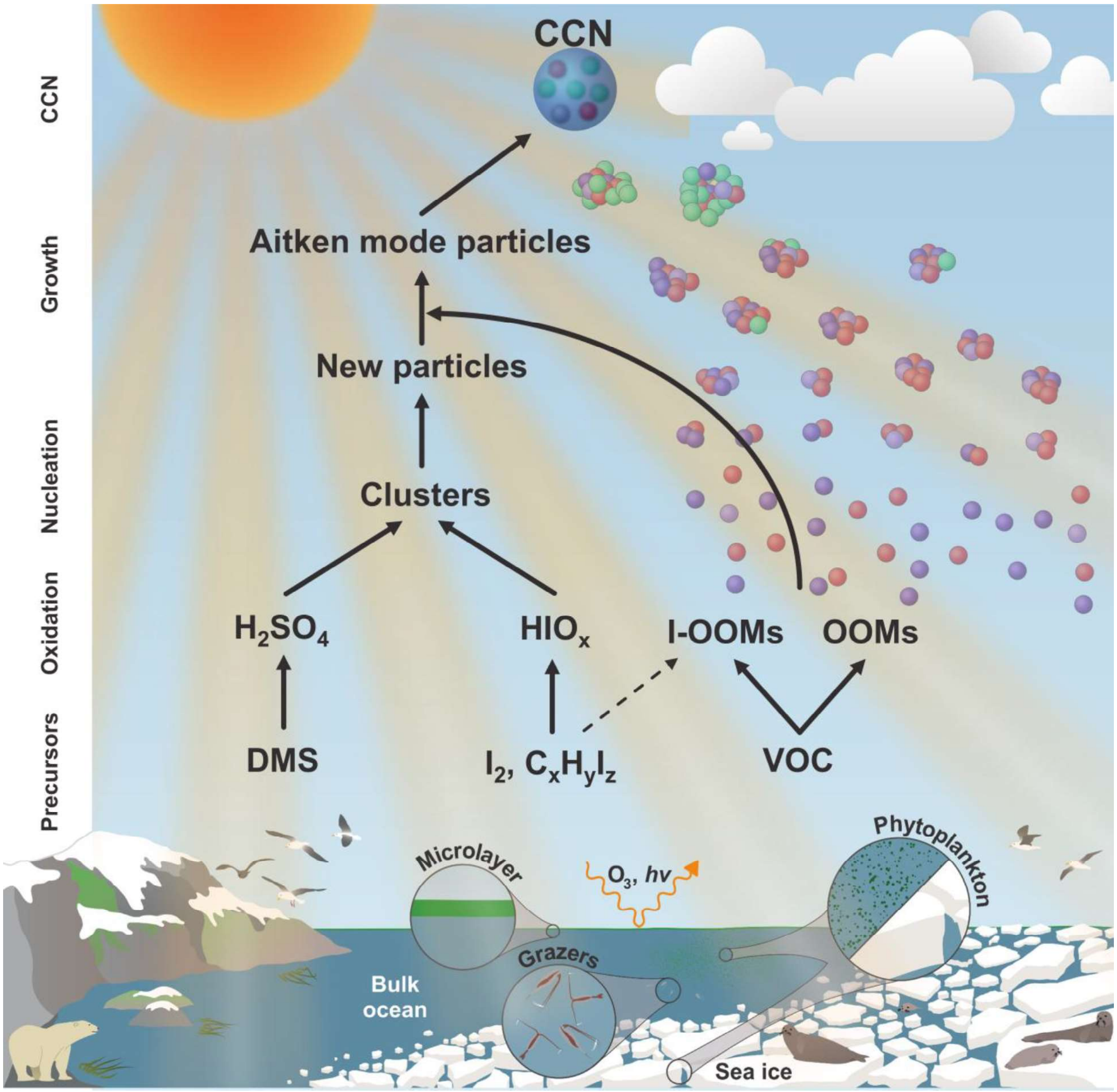

Fig. 5 | Schematic representation of key processes in this study, from natural nucleation and growth of new particles to CCN in the marine boundary layer. Volatile gases are emitted from diverse sources including sea ice, open ocean, coastal systems and bird colonies [33] via both biotic and abiotic processes. They then undergo oxidation to produce NPF precursors such as HIO_3 [56], HIO_2 [39], **and** H_2SO_4, driving particle formation, and ~~and MSA~~ , OOMs [39] and I-OOMs **which are the primary drivers of particle growth**. I-OOM formation (dashed line) is hypothesized **to occur through autoxidation termination via iodine-containing radicals** ~~based on previous studies [39]. These compounds contribute to particle nucleation and growth to form CCN., although their relative importance will vary depending on the season and location.~~

We have now moved the previous Fig. 5 to SI – This figure synthesized both our understanding and those in the literature. Our intention was to put our new finding in the current state of the understanding in NPF mechanisms in the Arctic. For example, while our data show that NH3 was below detection limits and MSA played a negligible role in initial clustering during this specific campaign, these species are present and were detected in other studies such as Beck et al. This reflects that our newly validated mechanisms coexist with the previously recognized pathways.

Comment 5: Another critical limitation of the current study is the absence of natural ion measurements using APi-TOF. While the authors consistently highlighted the comprehensiveness of their dataset compared to earlier studies (Table R1), the APi-TOF remains a critical instrument for atmospheric nucleation studies. This is particularly relevant in pristine environments like the Arctic, where measuring abundant small ionic clusters is often essential for identifying specific nucleation schemes. Without these data, it is difficult to definitively assess the importance of mechanisms such as SA-NH3 and SA-DMA nucleation.

Furthermore, neutral measurements obtained using Nitrate-CIMS can be influenced by natural ion detection if an inlet ion filter is not installed. As the manuscript does not mention the use of such an ion filter, I assume one was not present. Without parallel information from an APi-TOF, it becomes difficult to determine if species such as H2SO4-HSO4- are genuine neutral sulfuric acid dimers (which would indicate strong sulfuric acid nucleation) or if they originate from ambient natural ions. Consequently, there is a risk that the clusters presented in Figure S5 - with the exception of HIO3HIO2NO3- - may be sourced from natural ions rather than neutral clusters. In fact, it appears highly probable that the presented trimers are natural ions rather than neutral clusters, given that the reported nucleation rates are primarily below 1 cm-3 s-1. Generally, nucleation events at such low rates do not yield neutral cluster concentrations distinct enough to exceed the typical detection limit of nitrate-CIMS (approximately 1e4 cm-3). I suggest the authors employ a simple kinetic model to simulate the cluster formation process to evaluate this feasibility. When doing so, they should avoid simply assuming kinetic collision rates for HIO3 or H2SO4, given their own argument that the nucleation mechanism involves additional components such as HIO2.

This possibility reinforces the concerns raised in Comment 2 regarding the DMA cluster (C2H7NHIO2IO3-); if this signal originates from natural ions, a single peak in the spectra is essentially inconclusive unless a corresponding cluster sequence is observed. Unless the authors observe forms involving the NO3- ion (which would confirm chemical ionization), this uncertainty remains.

I strongly suggest that the authors review their arguments with this limitation in mind and acknowledge this caveat in SI and main text. Additionally, I recommend structuring the narrative to prioritize solid, valid measurements - such as the concentration of neutral acids and observed aerosol formation and growth rates - before expanding into highly uncertain discussions regarding DMA and OOMs, if such discussion is necessary at all.

Response: We agree that without an APi-TOF and an inlet ion filter, our Nitrate-CIMS signals for certain clusters represent a combination of neutral clusters, naturally charged ambient ions, and fragmentation products of larger clusters.

We welcome the reviewer's specific recommendations, and have taken the following actions:

First, we have added explicit caveats to both the main text (Methods)

"Because the instrument was operated without an inlet ion filter, these cluster signals represent a combination of chemically ionised neutral clusters, naturally charged ambient ions, and potential fragmentation products of larger clusters. As detailed in Supplementary Text 3, kinetic modelling suggests that at the measured monomer concentrations, signals for specific strongly bound trimers (e.g., $HIO_2 \cdot H_2SO_4 \cdot H_2SO_4$) is potentially dominated by ambient natural ions rather than neutral clusters. However, because chamber studies demonstrate that the presence of a charge has minimal impact on the nucleation rates of these strongly bound systems, the detection of these clusters remains an indicator of the underlying chemistry"

… and the Supplementary Information

"To evaluate the feasibility of neutral cluster detection, we built a kinetic model based on the Atmospheric Cluster Dynamics Code (ACDC) to simulate the birth-death equations, utilizing binding energies from refs 8 and 22. The simulations indicate that under the observed atmospheric conditions and formation rates, the neutral concentration of the inorganic trimer ($HIO_2 \cdot H_2SO_4 \cdot H_2SO_4$) would likely fall well below our instrumental LOD. Consequently, the observed signal for this specific cluster is likely sourced primarily from ambient natural ions or the fragmentation of larger clusters upon charging with NO_3^-. In contrast, the model suggests the neutral $C_2H_7N \cdot HIO_2 \cdot HIO_3$ cluster could theoretically exceed the LOD at approximately 1 pptv of DMA. Regardless of the charge state, the presence of these strongly bound clusters confirms the active chemical pathways, as ionising radiation has been shown to have minimal impact on the nucleation rates of these systems"

Second, as detailed in our response to Comments 2 and 4, we have restructured the manuscript's narrative. We now prioritise the robust measurements of neutral acids, OOMs, and macroscopic formation rates, whilst being more cautious on the role of DMA. As advised, we intend to focus on the strongest empirical findings while maintaining complete transparency about instrumental limitations.

Thirdly, following the reviewer's suggestion, we built a kinetic model using the equations of ACDC [1] to evaluate the feasibility of neutral cluster detection. We simulated the birth-death equations using binding energies of [2] for the HIO2-H2SO4 system and from [3] for the HIOx-DMA system. Our simulations (Figure R1, R2) indicate that the neutral concentration of the HIO2-H2SO4-H2SO4 cluster would indeed likely fall well below our instrumental LOD under the observed conditions. Consequently, the observed signal for this specific cluster is indeed likely dominated by ambient natural ions or the fragmentation products of larger clusters (for which the concentration does indeed exceed the instrumental LOD). Conversely, the model suggests that the neutral C2H7N-HIO2-HIO3 cluster could theoretically exceed the LOD at a range of DMA concentrations, being the most abundant mixed HIOx-DMA cluster.

These results suggest that the presence of ambient ions does not alter our conclusions regarding the chemical mechanisms at play. Chamber studies demonstrate that for strongly bound clusters, such as the HIOx-H2SO4 system [2] and the SA-DMA system [4], the presence of a charge has minimal impact on the fundamental nucleation chemistry and rates. Therefore, whether the detected clusters were neutral or naturally charged, their presence unambiguously confirms that this synergistic clustering process is occurring in the ambient Arctic atmosphere. As the reviewer rightly notes, identifying the absolute dominant pathway (ion-induced versus neutral) would require a suite of instruments capable of resolving all components.

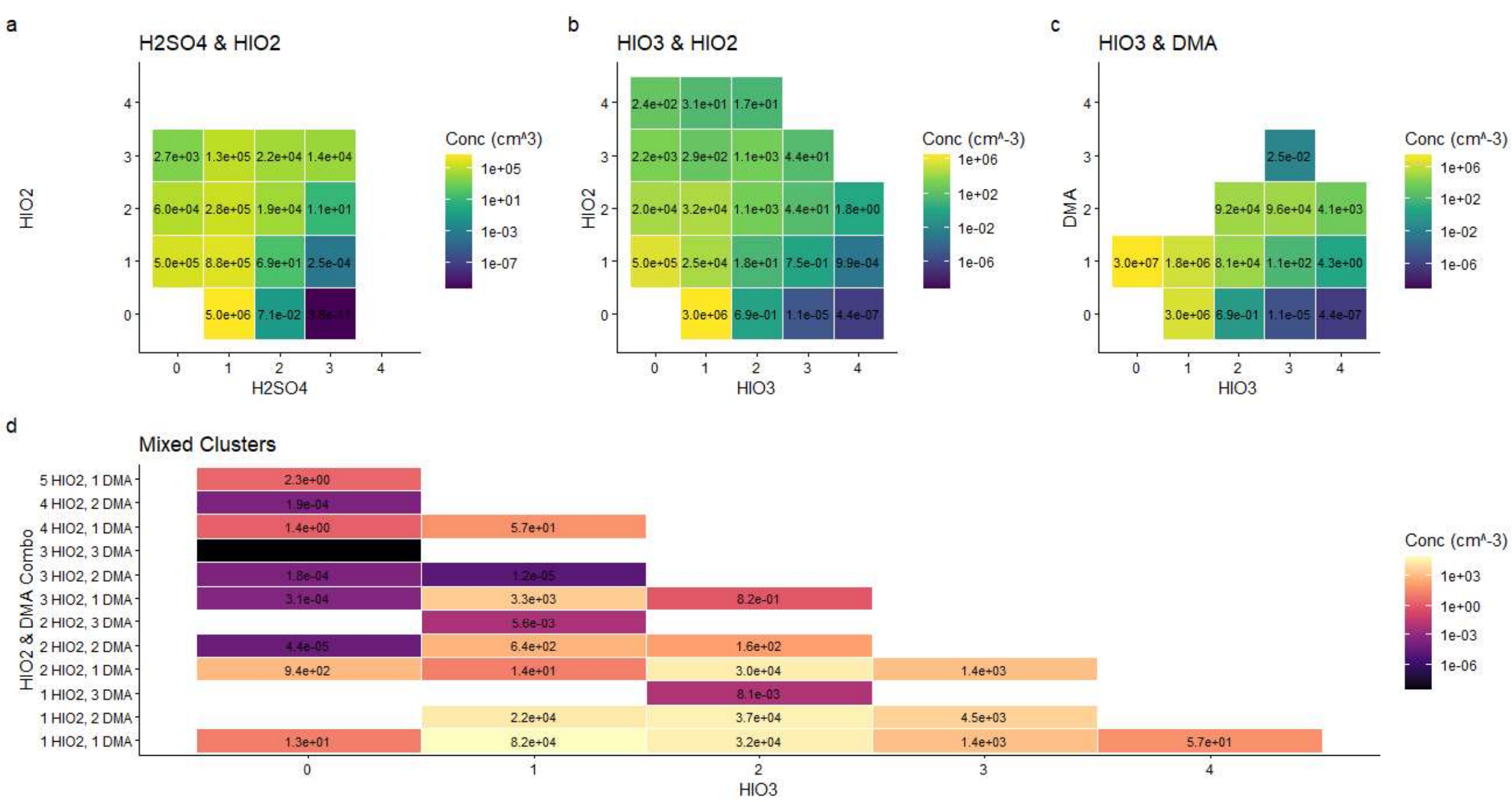

Figure R1: Simulated cluster concentrations on 1st June. Input concentrations: 3x10⁶ HIO3, 5x10⁶ H₂SO₄, 5x10⁵ HIO₂, 1 pptv DMA. Temperature set to 273.15 K.

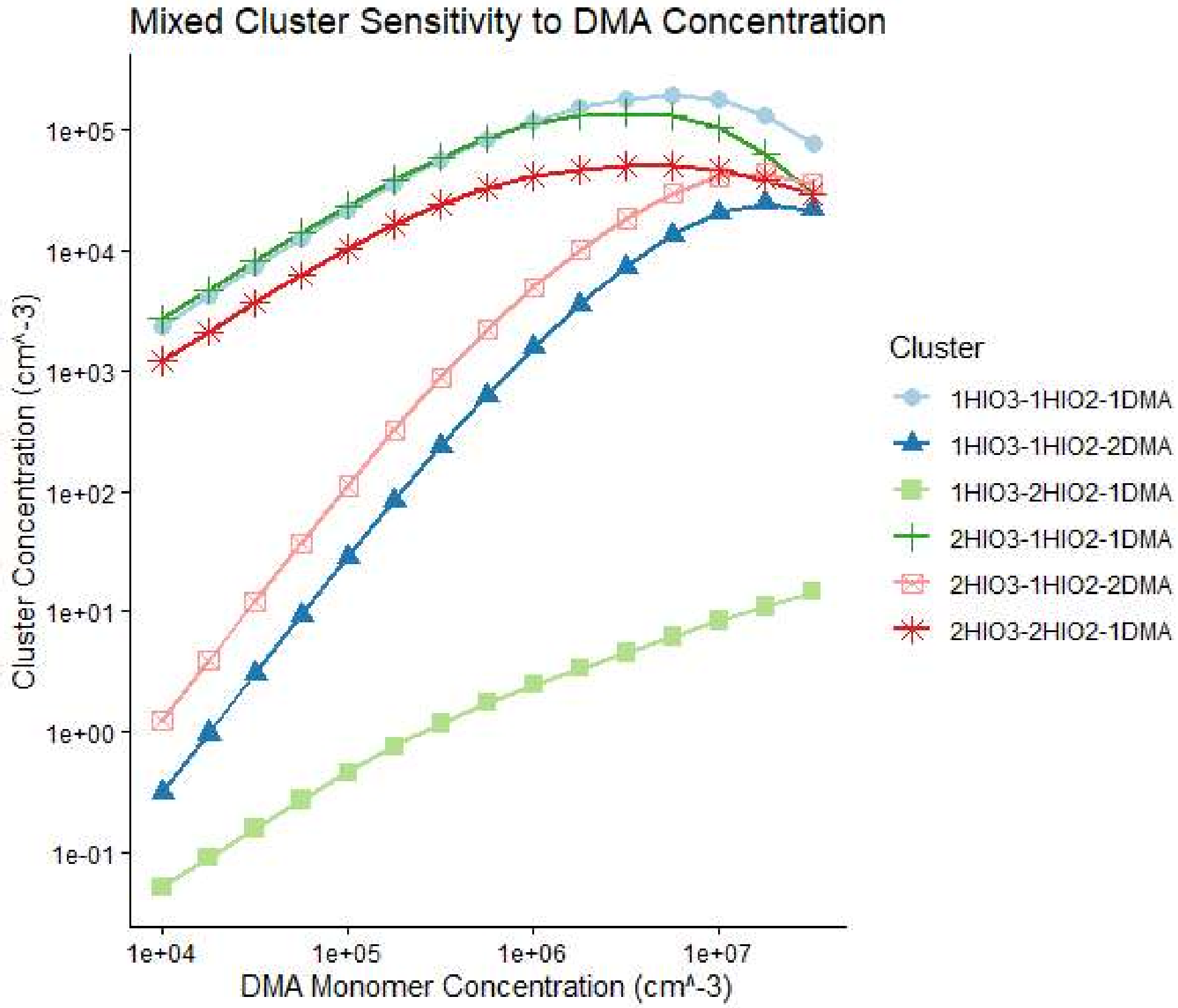

Figure R2: sensitivity of mixed concentrations to DMA concentrations. $3x10^6$ HIO3, $5x10^5$ HIO_2

Comment 6: Lines 235-240 discuss the potential contribution of OOMs to aerosol nucleation. Based on the review history, this section appears to have been added in response to Reviewers 1 and 2 and was not present in the original manuscript. Issues raised by other reviewers remain regarding whether the VBS parameterization used here can adequately describe OOMs derived from aldehydes. While I commend the authors for choosing a general form over specific targeted forms for monoterpenes, this does not eliminate the substantial uncertainty inherent in the parameterization (imagine that's exactly why targeted forms are needed...). Specifically, the categorization of ELVOCs and ULVOCs - nor arguments based purely on concentration - can be used as direct evidence of whether OOMs are contributing to nucleation.
To address this, I suggest the authors color-code their field observations in Figure 3B by OOM concentrations. This visualization could help verify the role of OOMs in nucleation: if OOMs are indeed critical to the process, one would expect to see a vertical trend associated with OOM levels in Figure 3B. Conversely, if no pattern emerges, OOMs might simply be co-emitted with other vapors without driving the nucleation event itself. Including this analysis as a new SI figure would allow the discussion in this section (and elsewhere in the paper) to be grounded in observational evidence rather than relying heavily on uncertain parameterization calculations.

Response: We thank the reviewer for this insightful comment. Below we include a figure (in the manuscript as Figure S6) where we colour Figure 3b by our four PMF factors, as well as different volatility classes of OOMs. We note that there is a vertical relationship between J1.7 and ULVOC, as well as J1.7 and I-OOMs. However, to explain our observed phenomena, we would instead be interested in the horizontal difference between the datapoints of He et al., 2023, and our datapoints. An agreement between the two systems indicates that the nucleation is dominated by HIOx-H2SO4, while datapoints sat to the left (e.g., similar J1.7 at lower (HIO3+H2O4)*HIO2) should have additional contributors to NPF. These results do not indicate that higher OOM concentrations, split by volatility nor PMF factors, seem to explain our elevated J values relative to CLOUD. We amend our manuscript as follows:

"…The role of DMA in enhancing nucleation rate is corroborated by quantum chemical modelling studies showing that DMA can accept a proton from HIO_3 as a base, forming a stable cluster (DMA-HIO_2-HIO_3) [13]. **OOMs may also enhance nucleation rates [38], however, we see no positive relationship between OOM concentrations (of any volatility class or source) and enhanced nucleation rates relative to He et al. [17] (Fig S6). This indicates that the OOMs we observed do not appear to have a substantial enhancing effect.**

~~Furthermore, given the high abundance of OOMs, particularly ELVOCs (Fig. 1C), it is also plausible that they contribute to the enhancement of nucleation rate, either by participating directly in nucleation or by stabilising the initial inorganic clusters. This would be consistent with studies from other environments showing OOMs can enhance nucleation rates [38]. Furthermore, as the volatility-reducing effect of iodine in our observed I-OOMs is not fully constrained, it is possible that some of the observed I-OOMs are ULVOCs, which may participate in the initial nucleation.~~

Our results demonstrate that H_2SO_4, HIO_3, and HIO_2, are the core components of a synergistic nucleation mechanism in our study area. This provides real atmospheric validation of the multicomponent nucleation mechanism identified by He et al. [17], at lower vapour concentrations than measured in CLOUD ~~(Fig. 3)~~. ~~and further suggests that in complex ambient environments, this process is likely modulated by amines and organic vapours. The precise contribution of each warrants further investigation.~~"

a

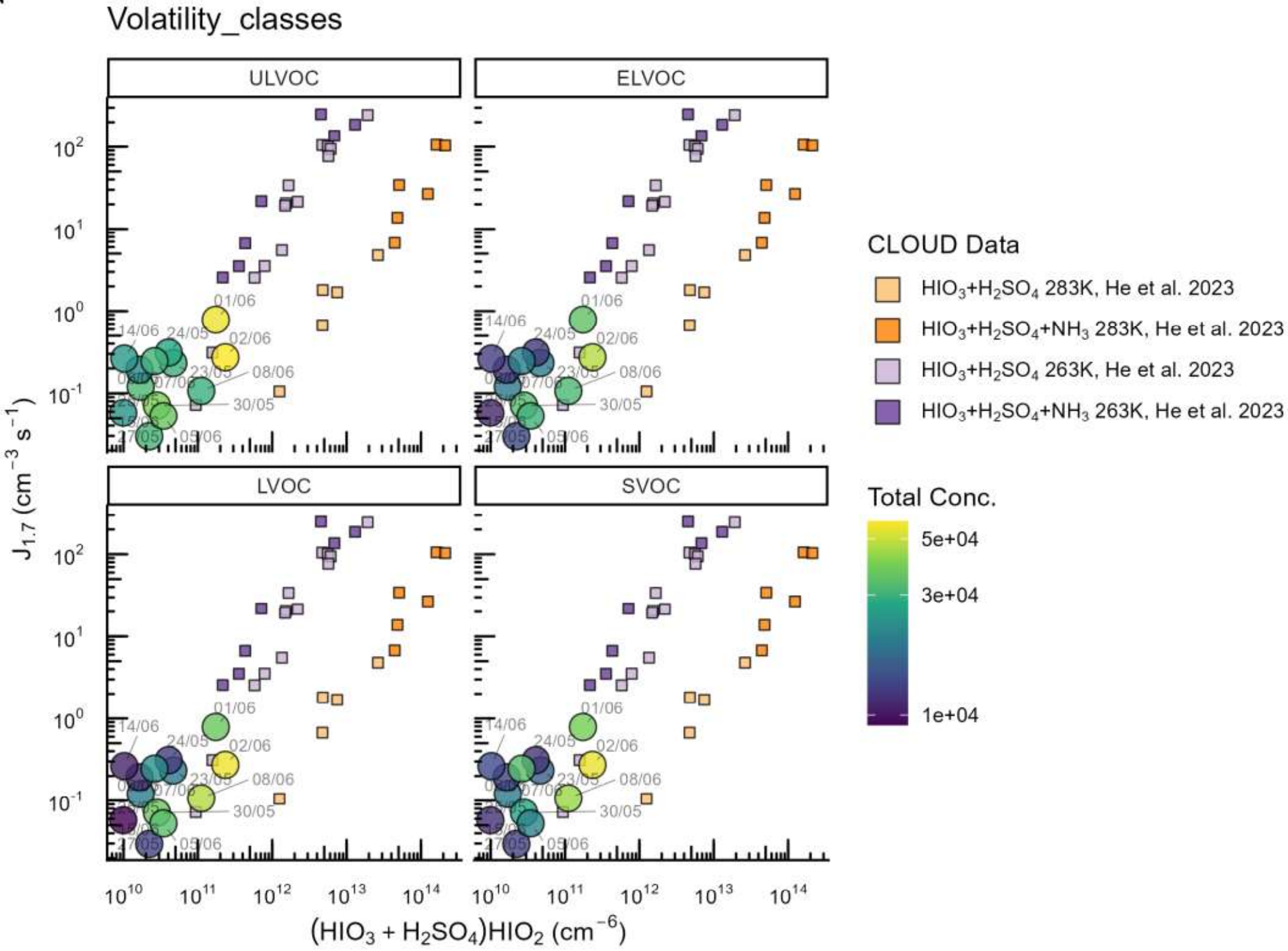
Volatility_classes
ULVOC
ELVOC
LVOC
SVOC
J1.7 (cm−3 s−1)
(HIO3 + H2SO4)HIO2 (cm−6)
CLOUD Data
HIO3+H2SO4 283K, He et al. 2023
HIO3+H2SO4+NH3 283K, He et al. 2023
HIO3+H2SO4 263K, He et al. 2023
HIO3+H2SO4+NH3 263K, He et al. 2023
Total Conc.
5e+04
3e+04
1e+04

b

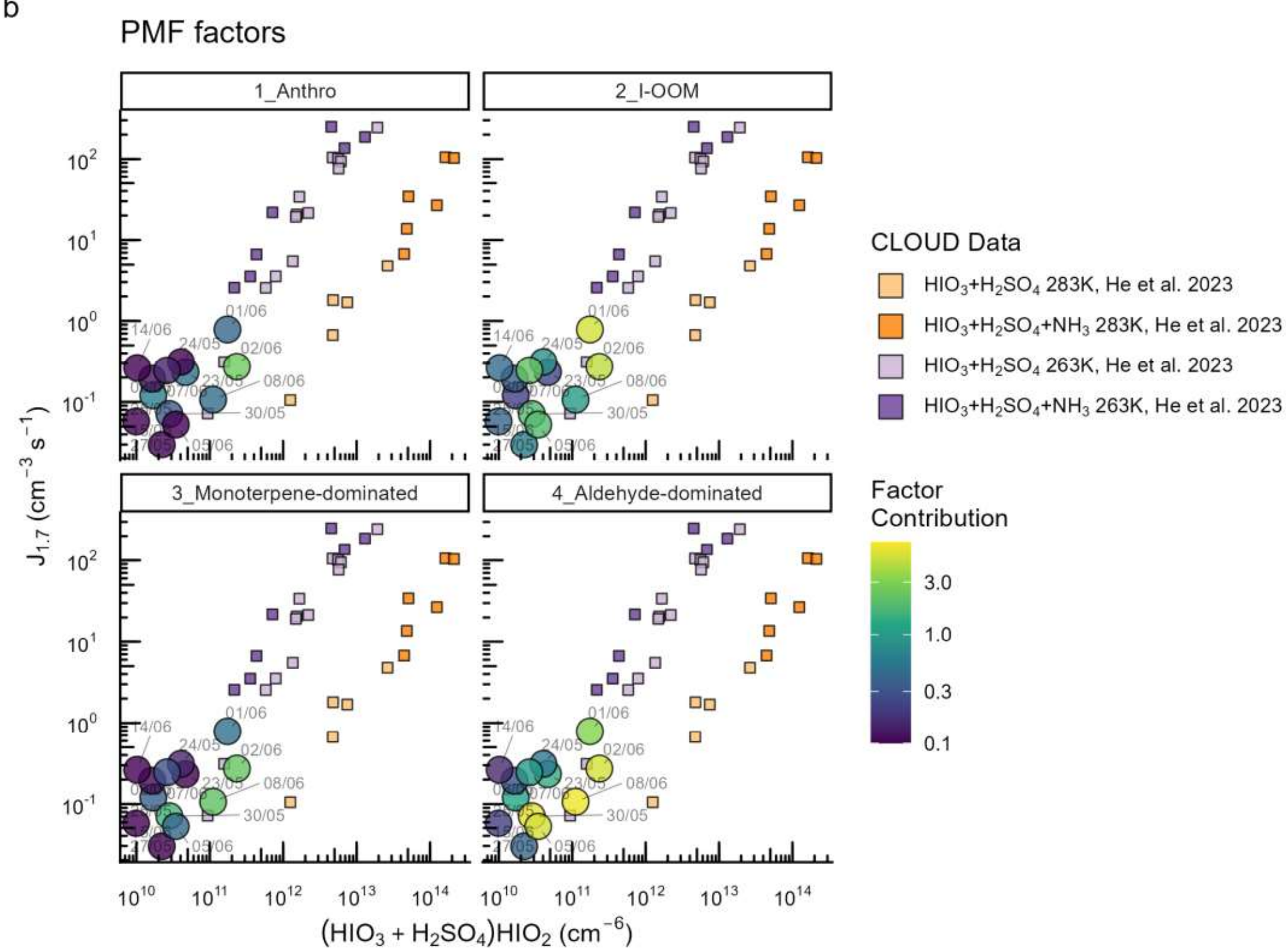
PMF factors
1_Anthro
2_I-OOM
3_Monoterpene-dominated
4_Aldehyde-dominated
J1.7 (cm−3 s−1)
(HIO3 + H2SO4)HIO2 (cm−6)
CLOUD Data
HIO3+H2SO4 283K, He et al. 2023
HIO3+H2SO4+NH3 283K, He et al. 2023
HIO3+H2SO4 263K, He et al. 2023
HIO3+H2SO4+NH3 263K, He et al. 2023
Factor Contribution
3.0
1.0
0.3
0.1

Figure S6: As Figure 3b, coloured by (a) different volatility classes of OOMs & I-OOMs, and (b) different PMF factors. We see no relationship between the enhancement to NPF relative to He et al. (2023) and our observed OOMs & I-OOMs.

Comment 7: I also feel that Reviewer 5's suggestion to link current observations to the CCN budget, as well as to cloud and radiative forcing (presumably via global modelling), falls outside the scope of this study.

Response: Thank you. We agree and end our manuscript with some closing thoughts on how the matter would be addressed.

Comment 8: The authors should acknowledge that their acid measurements were conducted under considerably more variable conditions compared to controlled laboratory experiments, which inherently introduces greater uncertainty. Additionally, the calibration procedure for sulfuric acid represents a significant source of error. A factor-of-two uncertainty in acid concentrations can propagate non-linearly, readily resulting in an order-of-magnitude difference in derived aerosol nucleation rates. With this consideration, the data presented in this study are not necessarily in conflict with CLOUD data; on the contrary, given the aforementioned uncertainties, the agreement is actually quite reasonable.

Response: Thank you for the supportive comment. We agree entirely. Please see below our extended error analysis. We make an amendment to our main manuscript

"Following He et al. [17], we plotted $J_{1.7}$ versus (HIO_3 + H_2SO_4) × HIO_2, representing the formation rate of H_2SO_4-HIO_2 and HIO_3-HIO_2 (Fig. 3B). ~~**While** our $J_{1.7}$ measurements are comparable with those observed for the HIO_x-H_2SO_4 and HIO_x-H_2SO_4-NH_3 in CLOUD chamber experiments at -10 °C within uncertainties for most days, **the central values of our observations sit slightly what we would anticipate based on CLOUD**.~~ Of all systems that have been studied at the CLOUD chamber, the HIO_x-H_2SO_4 system most consistently reproduces our $J_{1.7}$ values, indicating the key role of both HIO_x and H_2SO_4 in our observed nucleation events. Considering the average ambient temperature of 1.4 °C during nucleation periods, the central value of our $J_{1.7}$ values on some days **sit slightly above our interpolated fitting line based on** ~~are higher than anticipated from~~ the temperature dependence measured in the CLOUD chamber [17] (at -10 and +10 °C). **This enhancement is likely driven by two key factors: reduced cluster scavenging and** the potential participation of other stabilising species. **Firstly, our condensation sink was lower than in the CLOUD chamber, which can cause deviations due to differences in cluster scavenging [37].** Second is the potential participation of other stabilising species. One plausible contributor is DMA, as evidenced by the occasional detection of DMA clustered with HIO_x (Fig. S4 and Extended Data Figures 3-4). The DMA-HIO_2-HIO_3 cluster signal correlates with HIO_3 concentrations and is highest when HIO_3 is at its maximum (Figs. S4-5). Quantum chemical modelling studies also showed that DMA can accept a proton from HIO_3 as a base, forming a stable cluster (DMA-HIO_2-HIO_3) [38]. OOMs may also enhance

nucleation rates [39], however, we see no positive relationship between OOM concentrations (of any volatility class or source) and enhanced nucleation rates relative to He et al. [17] (Fig. S6). This indicates that the OOMs we observed do not appear to have a substantial enhancing effect."

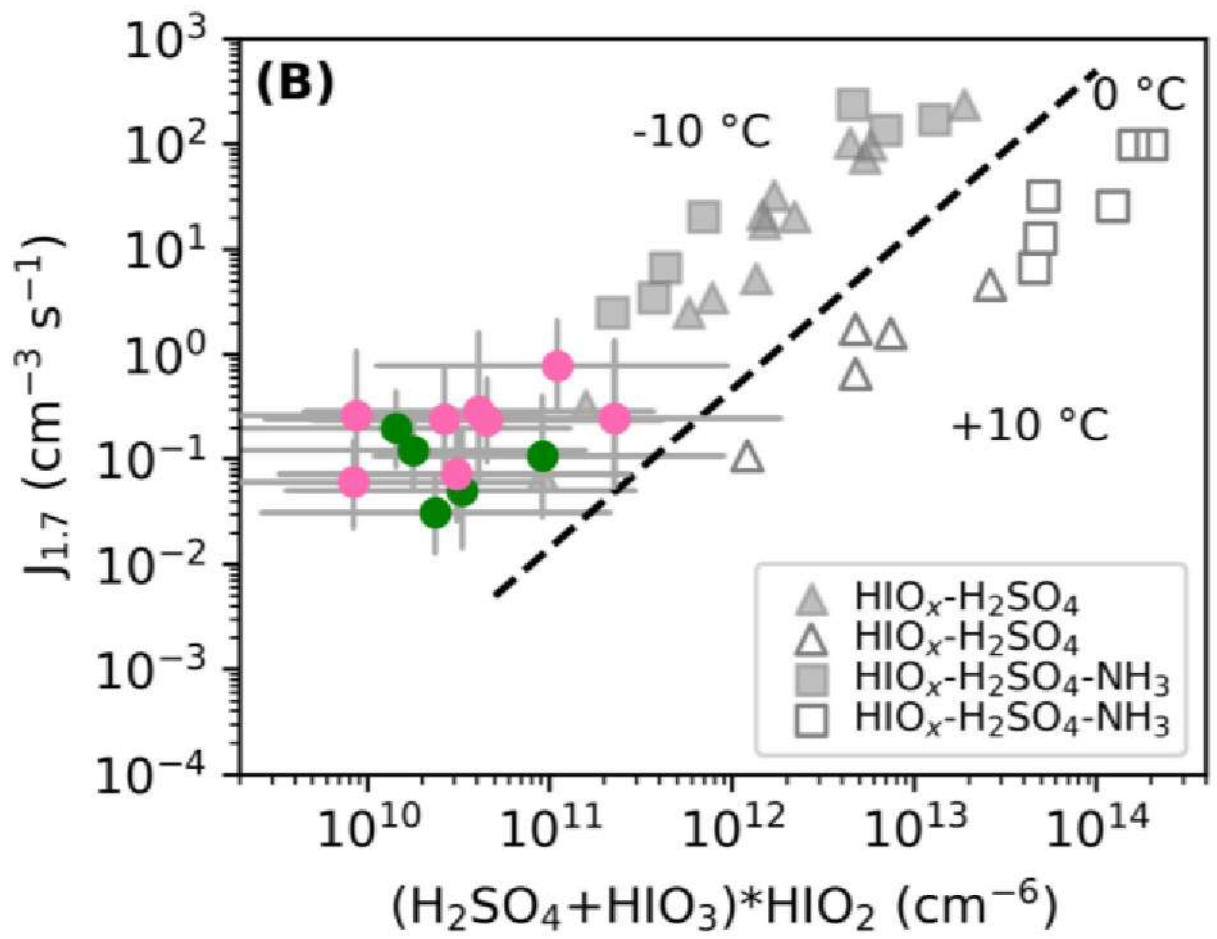

Fig. 3| Multi-component nucleation involving HIO_x. … (B) $J_{1.7}$ versus (HIO_3 + H_2SO_4) × HIO_2 for all measured nucleation events, together with CLOUD. Dashed line in (B) is parallel to and has the same distance to the regression lines of the $J_{1.7}$ and (HIO_3 + H_2SO_4) × HIO_2 at the temperature of 10 and -10°C. Assuming a linear relationship, this line represents what would be anticipated of 0°C. Pink and green data points in (B) represent average values during each nucleation event. Error bars represent one standard deviation of the mean value. The hollow circle in the figure represents the HIO_2 values estimated based on the fitting equation shown above. See Methods 3 and Supplementary Text 3 for estimates of uncertainties in concentration measurements and nucleation rate, and limits of detection.

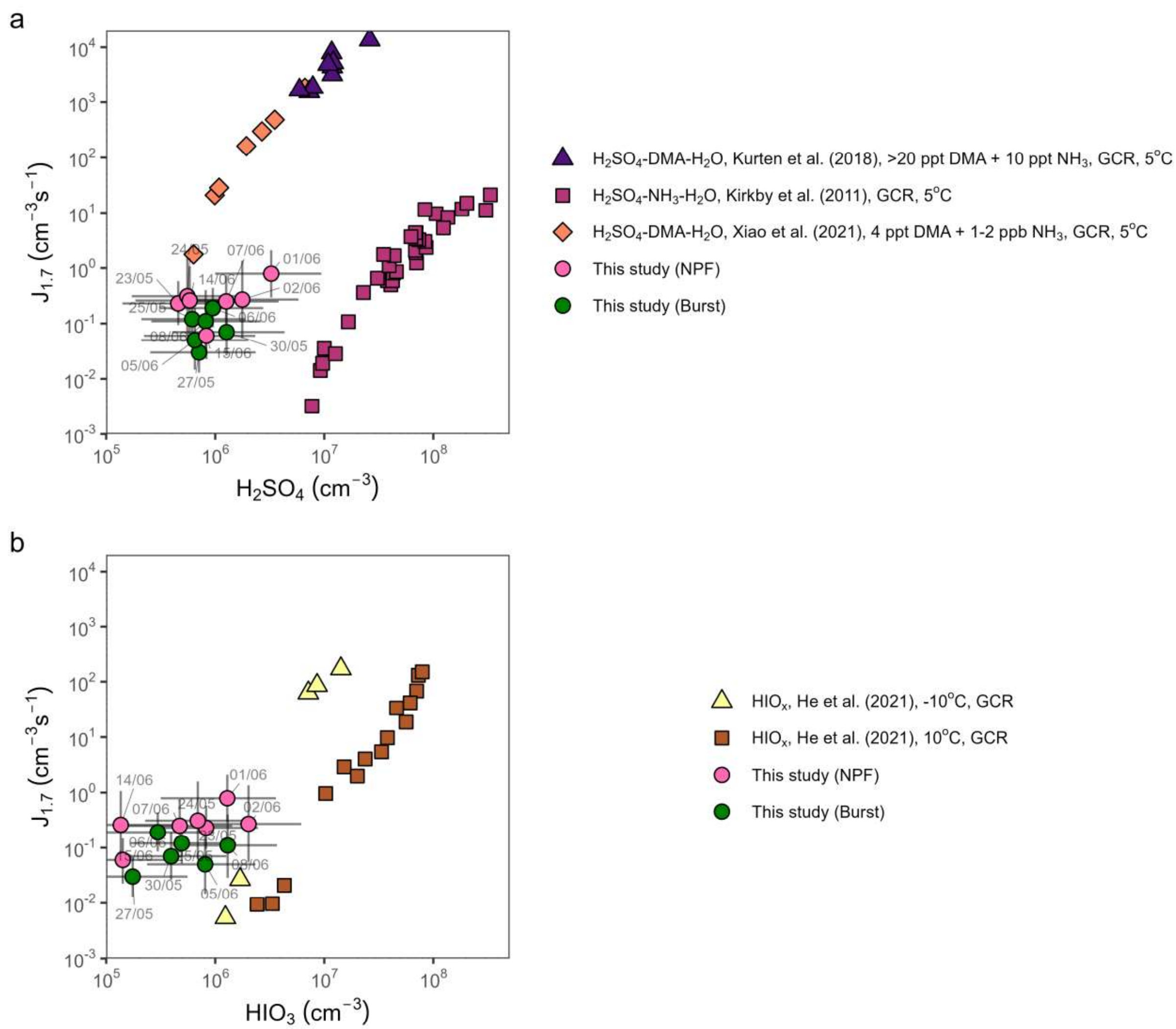

Figure S8: Comparison of nucleation rates for the H_2SO_4, and HIO_3 systems with previous studies [9,10]. (A) Particle formation rates $J_{1.7}$ plotted against H_2SO_4 concentrations. Data points from CLOUD chamber experiments represent H_2SO_4-DMA-H_2O with **>20 pptv** DMA and 10 pptv NH_3 (purple triangles) [9], H_2SO_4-DMA-H_2O with 4 pptv DMA and 1-2 ppbv NH_3 (orange diamonds) [11], and H_2SO_4-NH_3-H_2O (magenta squares) [9]. ~~The H_2SO_4-DMA-H_2O data from Almeida et al. [12] are presented without the self-coagulation correction later described by Kürten et al. [13]; this correction is not available for these specific low-concentration experiments and is negligible given the low particle concentrations, and the experiments of Almeida et al. are likely the most representative of our measured conditions.~~ (B) Particle formation rates $J_{1.7}$ plotted against HIO_3 concentrations. Data points are from CLOUD chamber

experiments at 10 °C (yellow triangles) and –10 °C (brown squares) [10]. For both plots, pink circles represent NPF events for this study, while green circles denote burst events. Each data point is labelled on the plot using the day/month format.

And we make the following additions to our methods when we discuss our particle counting instruments:

"… and estimate a 30% relative uncertainty in our quantification of particle number related to both uncertainty in measurement, and uncertainty associated with harmonizing measurements from these different instruments."

When we discuss our CIMS data:

Uncertainties in CIMS measurements are derived from chamber experiments and subsequently adapted for field conditions. For inorganic acids, while CLOUD chamber studies estimate sulfuric acid uncertainties at approximately a factor of 2 (+100%/−50%) [31], we adopt a conservative factor of 3 (+200% / -67%) to account for the additional inlet turbulence and environmental variability inherent to ship-borne measurements.

The uncertainty in OOM measurements arises from several compounding physical sources, including the application of the sulfuric acid calibration coefficient, mass-dependent transmission efficiency, sampling line losses, and the charging efficiency of OOMs by nitrate ions. Because the calibration factor C is estimated based on the collision-limited sensitivity of H_2SO_4, it provides a lower-limit concentration for species charged less efficiently, such as OOMs [78]. Chamber studies reflect this limitation, suggesting ELVOC uncertainties of a factor of 1.8 (+80%/−45%), whilst acknowledging that less oxidised species (LVOCs/SVOCs) may be underestimated by up to a factor of 4 (+300% / -75%) due to lower charging efficiencies [71]. To comprehensively encompass these errors, we apply an overall uncertainty of a factor of 5 (+400% / -80%) to the total measured OOM concentrations.

For the comparisons with CLOUD chamber data the systematic instrument uncertainties (factor of 3) for the quantification of HIO_x and H_2SO_4 were combined in quadrature with the relative atmospheric standard deviation of the measurement for each nucleation event ($\frac{\sigma_{std,X}}{X}$).

For each individual species (Figure S8) the total relative fractional uncertainties (δ_{tot}) were calculated as:

$$\delta_{tot} = \sqrt{(\ln 3)^2 + \left(\frac{\sigma_{std,X}}{X}\right)^2}$$

For the multi-component plot (Figure 3b, $X = HIO_2 \times (H_2SO_4 + HIO_3)$), because the systematic uncertainties of the individual components are derived from the same instrument, they were treated as systematically correlated. The log-space errors compound linearly, resulting in a combined systematic variance of $2ln(3)$. This was combined in quadrature with the relative atmospheric standard deviation using the Gaussian formula:

$$\delta_{tot} = \sqrt{(2\ln 3)^2 + \left(\frac{\sigma_{std,X}}{X}\right)^2}$$

The absolute upper and lower bounds for all vapour concentrations were then determined by multiplying and dividing the total value X by the respective geometric error factor $\exp(\delta_{tot})$. This gave an average of a factor of 8.4 uncertainty on the x-axis of Figure 3b.

… And in the discussion of J values:

Because $J_{1.7}$ were inferred rather than measured directly, uncertainties were propagated through the survival probability framework following Cai et al. (2022) [94]. The overall uncertainty for $J_{1.7}$ comprises the baseline uncertainty in J_4 (factor of 2, see above). The errors regarding CoagS were approximated to be equivalent to the errors equivalent to quantification of particle number (30%) and those relating to the growth rate to be 50%.

Due to the exponential nature of the survival probability scaling, the resulting uncertainties are log-normally distributed. The relative systematic uncertainty ($\delta_{sys,J}$) was calculated using the Gaussian error propagation formula, combining the log-normal variance of J_4 (ln 2) with the fractional variances of the sink and growth rate:

$$\delta_{sys,J} = \sqrt{(\ln 2)^2 + E^2 [0.30^2 + 0.50^2]}$$

Where E is the integrated ratio of the coagulation sink to the growth rate across the 1.7 to 4 nm interval ($E = \Delta d_p \frac{CoagS}{GR}$). To determine the total fractional uncertainty ($\delta_{tot,J}$) visualised as error bars in Figure 3b and Figure S8, this systematic error was combined in quadrature with the relative standard deviation ($\frac{\sigma_{std,J}}{J_{1.7}}$) of the measurement period:

$$\delta_{tot,J} = \sqrt{\delta_{sys,J}^2 + \left(\frac{\sigma_{std,J}}{J_{1.7}}\right)^2}$$

Using this methodology, we estimate our uncertainty in our J values to be approximately a factor of 3.2 This falls within the uncertainty range of factor 2–5 reported in recent polar aerosol studies [98].

Minor comments:

CCN is written as 'Cloud Condensation Nuclei (CCN)' in the abstract, but as 'cloud condensation nuclei (CCN)' in the introduction. This is a minor issue, but it would be good to be consistent with capitalization (also for other terms).

Response: We revised this.

Line 87: I think Baccarini et al. (2020) reports both iodine NPF and growth? Could the authors double check?

Response: Baccarini indeed do report that the particle growth is driven by iodine oxoacids, with HIO_3 describing a lot of the observed particle growth. We mention in the line 87 that 'In the high Arctic, HIO3 contributes to particle growth (Fig. S1B) 14', where ref. 14 refers to Baccarini et al. (2020)

Line 93: "...not necessarily to new particle formation"

Response: Amended

Figure S3: fix the caption of panel D

Response: We have fixed it!

Lines 158-161: is the correspondence of numbers and classification correct? Should the NPF events be with 0.0005 s-1 CS? Please clarify.

Response: We have checked and revised it into below:

'The condensation sink, the -rate at which low-volatility vapours like sulfuric acid are lost to pre-existing particles, was ~~40%~~ lower on NPF event days as compared to ~~non-event days, and highest on~~ burst events and non-event days (7×10^{-4} s^{-1}, 5×10^{-4} s^{-1}, and 1×10^{-3} s^{-1} respectively) (~~Fig. S4~~ Extended Data Fig. 2 and Table S1).'

Lines 185-187: Please consider rephrasing this sentence, as it feels somewhat congested. In addition, 'power-law relationship' and 'non-constant ratio conveys somewhat similar concepts.

Response: We have revised into below:

"The concentrations of HIO_3 and HIO_2 were well correlated during our campaign, with median concentration 2×10^5 cm^{-3} for HIO_3 and 1.6×10^4 cm^{-3} for HIO_2."

We revised the text to avoid redundancy by retaining the power-law description (supported by the fitted log–log regression) and explicitly stating its implication (slope < 1 means concentration-dependent HIO_2:HIO_3 ratio), rather than using both "power-law relationship" and "non-constant ratio."

Line 193: CLOUD chamber, Mace Head and Ny-Ålesund. Also, where is the solid red line? I don't see that in the panel A? Is it the dashed cyan line?

Response: We have amended the words 'solid red line' to 'dashed red line'.

Lines 205-207: is it really true that J1.7 has not been reported in the Arctic? I'm sure that things like J1.5-J1.8 have been reported for example in Beck et al. (2021).

Response: Beck et al. (2021) report formation rates at 1.5 nm ($J_{1.5}$). These parameters are indeed similar enough as a comparison is possible. We amend as follows

"The peak hourly formation rate at 1.7 nm ($J_{1.7}$) for each day varied from 0.04 to 1.4 cm^{-3} s^{-1}, **in line with values at Svalbard of 0.33 ($J_{1.5}$)[13] and 0.04 (J_{3})[31]"**

Where ref. 13 is Beck et al., and ref. 31 is Lee et al (https://doi.org/10.5194/acp-20-13425-2020)

Figure S8: I suggest remove the Almeida data from the comparison. Instead, use the data from Xiao alone or else from Kürten et al. (2018; 10.5194/acp-18-845-2018), which primarily provides a correction to the Almeida et al. (2013) dataset (which is not precise by current standard). For the reasons and details, see Kürten et al. (2018). Few other reviewers also mention this and the responses by the authors are not really convincing... To be frank, the Almeida data is simply not correct and should not be used anymore.

Response: Please see revised figure and caption in response to your Comment 8.

Lines 262-266; 334-336: these seem interesting. Is there a way we can speculate the source of I-OOM? Is it possible that they source from the particles through heterogeneous reaction of organics and iodide/iodate, instead of the other way around? If this is the case, there growth contribution needs to be questioned.

Response: Thank you. We do find this result interesting and require more laboratory work to understand their formation. We proposed potential pathways in SI section 5:

'We propose a hypothesis for I-OOM formation. The diurnal profiles of these compounds indicate daytime formation, implicating photochemical processes. Given that reactions between iodine-containing species and VOCs are significantly slower than those involving other halogen radicals such as Br and Cl [115], direct reactions between VOCs and iodine radicals are unlikely pathways for I-OOM production, especially as we do not anticipate substantially higher iodine radical concentrations relative to chlorine and bromine, as indicated by their relative oxidation product concentrations during recent Arctic campaigns [116,117]. Molecular composition offers further insights: for instance, $C_{10}H_{15}IO_{3-9}$ matches well-characterized OOMs ($C_{10}H_{16}O_{3-9}$) formed via monoterpene oxidation in iodine-free environments [39], differing only by the substitution of a hydrogen atom with iodine. Typical OOM formation proceeds through autoxidation, wherein the

radical chain is terminated either by radical–radical collisions or propagated via self-reactions. These terminating radicals are often OH, HO_2, or NO. In the underexplored marine atmosphere, we hypothesize that termination may involve iodine-containing radicals such as IO or organic iodine species (CxHyI). Arctic observations report IO levels of ~3 ppt [50], sufficient to intercept a significant fraction of autoxidation reactions and yield the observed I-OOMs, as these concentrations are comparable with OH and HO_2 concentrations in the Arctic [118]. Hence, I-OOM formation likely adheres to established autoxidation mechanisms, diverging in the identity of the terminating radical. Further laboratory validation is warranted to substantiate this mechanism.'

Line 291: ... primary particle growth drivers.

Response: We have revised it to the following (avoiding the use of the word "primary" for the sake that "primary particle growth" could be understood to mean the growth of primary particles: "OOMs are indeed the dominant drivers of particle growth"

Lines 295-296: these are overstatements - it may be true to the I-OOM. However, other studies have carried out i

n simple terms by comparing OOM concentrations with GR. These also provided molecular information in the MS. If the statement refers to using the VBS method, please be specific since what "molecular-level" means is rather vague and can easily trigger debates.

Response: Thank you. We amend as follows

"Our findings provide the first conclusive evidence linking OOMs ~~at a molecular level~~, including those containing iodine, to the growth of new particles in the Arctic."

We also amend the introduction

"Furthermore, we show ~~at a molecular level~~ that subsequent growth to CCN sizes is dominated by the condensation of OOMs, including a previously unobserved class of iodine-containing OOMs"

Lines 304-306: define MIZ when first appears.

Response: We included a definition as follows:

"…the marginal ice zone (the region with sea-ice concentration between 10% and 80%)"

Lines 396-398: I feel the discussion about monoterpene is a bit biased. In the plot, even after scaling of a factor of 10, monoterpene concentration is still minor. The authors should report the real concentration here as the scaling is a bit confusing as well. The authors should also quite frankly say that it is not so important from their measurements.

Response: The monoterpene time series was scaled by a factor of 10 solely to facilitate visual comparison. This adjustment is explicitly indicated in the legend ("C10H16 ×10") and was not intended to obscure the original concentrations. This helps to keep the trace observable.

In SI section 5, we explained this:

"Furthermore, monoterpenes and aldehydes have different reactivities towards OH and O_3, as well as different OOMs yields. Many of the most common monoterpenes are highly reactive [119] and readily undergo autoxidation [39]. Aldehydes are generally less reactive with OH and much less reactive with O_3 [119,120], and autoxidation [54]. OOM yields of aldehydes are therefore likely lower, depending on temperature and oxidants [54]. This likely explains the similar concentrations of the factors, despite different concentrations of precursors."

Lines 441-443: I think whether this is a warming or cooling in the Arctic is far from clear. Or would the authors argue differently?

Response: We agree. We suggest that:

"The net radiative effect of such changes is complex, potentially leading to surface warming over high-albedo snow and sea ice or a cooling effect over the open ocean [57,58]."

Figure S9, the colors of the same peak in panel A and B,C,D should be consistent.

Response: Great point. This has been amended.

Table S1 07/06/2022 data miss placed

Response: This has been fixed

Comment: The authors claimed that their results of OOM new particle formation is measured and supported by global modelling. I do not see the global modelling results from this study? Or is it supported by other studies?

Response: We did not do anything related to global modelling. We are not sure to which line this comment is directed. Perhaps this line? If so, yes, it refers to "*other* studies"

"Together with other observational and modelling studies, this leads to the prevailing hypothesis that OOMs facilitate particle growth to become CCN in the Arctic [11,15,21-23]."

Reviewer #7:

The manuscript by Du et al. presents ship-based measurements conducted from southeastern to western Greenland to investigate atmospheric new particle formation (NPF) in this understudied region. The measurement campaign employs an impressive and comprehensive suite of instruments, spanning precursor gas measurements, molecular cluster observations, particle-phase measurements, and cloud condensation nuclei (CCN) observations. This unique combination enabled the authors to follow the NPF process from its molecular origins through particle growth and directly to CCN formation. According to the authors, these measurements serve as in-situ evidence for HIOx/H2SO4 enhanced nucleation, corroborating previous findings from the CLOUD chamber study by He et al. (Science, 2023). In addition, the study identifies a new class of iodine-containing oxygenated organic molecules (OOMs), which plays a role in particle growth, while speculating that they may also influence early-stage nucleation. The study further demonstrates that the marginal sea-ice zone is associated with elevated concentrations of precursor gases and acts as a significant source region for CCN production. Overall, the manuscript reports potentially important observations of particle formation and growth that are not readily explained by the measured concentrations of known acidic precursors, together with the identification of a class of iodine-containing OOMs that has not been previously reported**. While these findings make the manuscript suitable for publication in Nature Geoscience, several issues must be addressed before the manuscript can be accepted for publication**. These are outlined below.

In addition, the authors are encouraged to substantially improve the quality of the Supplementary Material and to correct multiple inconsistencies between the figures and their captions. The Supplement should aid the reader in understanding the results, rather than creating uncertainty about what is being shown.

General Response: We thank the review for these constructive comments and addressed all below.

Major comments:

Comment 1: The authors did not directly measure particle number concentrations at 1.7 nm and therefore could not derive the particle formation rate at 1.7 nm from observations. Their measurements using the NAIS extend down to approximately 3 nm. To overcome this limitation, the authors first derived the formation rate at 4 nm (J4) from NAIS measurements after scaling the NAIS size distribution to match that of the nanoMPSS. They then inferred J1.7 from J4 using the survival probability formulation of Cai et al. (2022). It is great to see that the authors employed the most recent survival probability framework. However, because J1.7 is inferred rather than directly measured, the uncertainty associated with this calculation should be quantified and explicitly reported. Cai et al. (2022) demonstrate that a systematic difference in growth rate by a factor of three can lead to changes in the median survival probability exceeding two orders of magnitude. Given this strong sensitivity and considering that the inferred J1.7 values are used to benchmark the campaign observations against CLOUD experiments, it is essential that the J1.7

data presented in Figure 3 include uncertainty estimates. The authors are therefore encouraged to propagate uncertainties in the parameters of the survival probability equation (e.g., scaling factor between NAIS and nano-MPSS, growth rate and coagulation sink of small particle sizes) and assess how these uncertainties affect the derived J1.7 values. These uncertainties should be reflected as error bars or shaded ranges in Figure 3.

Response: Thank you for the comment. We agree and please see below our extended error analysis, with the results in Figure S8 below. We make an amendment to our main manuscript

"Following He et al. [17], we plotted $J_{1.7}$ versus (HIO_3 + H_2SO_4) × HIO_2, representing the formation rate of H_2SO_4-HIO_2 and HIO_3-HIO_2 (Fig. 3B). ~~**While** our $J_{1.7}$ measurements are comparable with those observed for the HIO_x-H_2SO_4 and HIO_x-H_2SO_4-NH_3 in CLOUD chamber experiments at -10 °C within uncertainties for most days,~~ ~~**the central values of our observations sit slightly what we would anticipate based on CLOUD.**~~ Of all systems that have been studied at the CLOUD chamber, the HIO_x-H_2SO_4 system most consistently reproduces our $J_{1.7}$ values, indicating the key role of both HIO_x and H_2SO_4 in our observed nucleation events. Considering the average ambient temperature of 1.4 °C during nucleation periods, the central value of our $J_{1.7}$ values on some days **sit slightly above our interpolated fitting line based on** ~~are higher than anticipated from~~ the temperature dependence measured in the CLOUD chamber [17] (at -10 and +10 °C). **This enhancement is likely driven by two key factors: reduced cluster scavenging and** the potential participation of other stabilising species. **Firstly, our condensation sink was lower than in the CLOUD chamber, which can cause deviations due to differences in cluster scavenging [37].** Second is the potential participation of other stabilising species. One plausible contributor is DMA, as evidenced by the occasional detection of DMA clustered with HIO_x (Fig. S4 and Extended Data Figures 3-4). The DMA-HIO_2-HIO_3 cluster signal correlates with HIO_3 concentrations and is highest when HIO_3 is at its maximum (Figs. S4-5). Quantum chemical modelling studies also showed that DMA can accept a proton from HIO_3 as a base, forming a stable cluster (DMA-HIO_2-HIO_3) [38]. OOMs may also enhance nucleation rates [39], however, we see no positive relationship between OOM concentrations (of any volatility class or source) and enhanced nucleation rates relative to He et al. [17] (Fig. S6). This indicates that the OOMs we observed do not appear to have a substantial enhancing effect.""

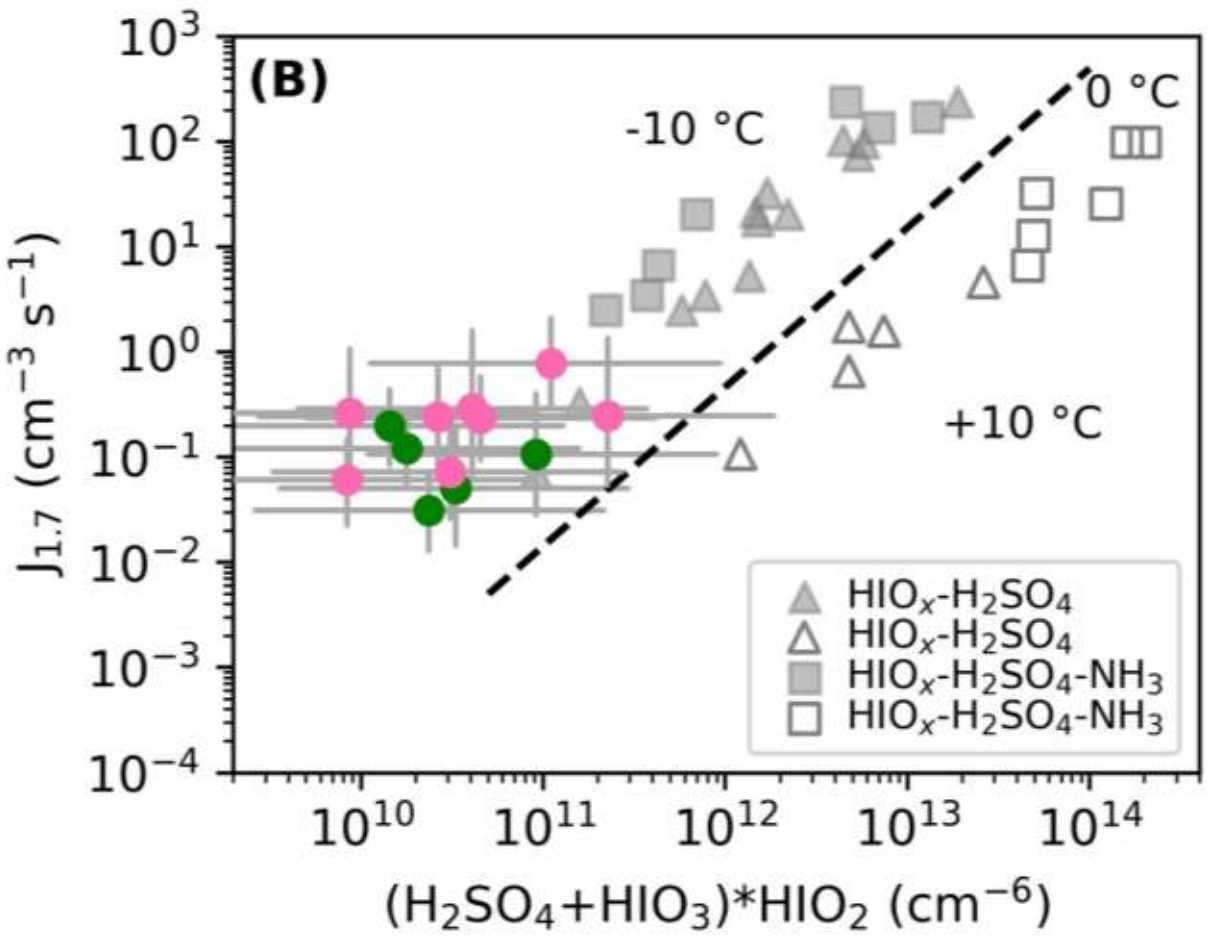

Fig. 3| Multi-component nucleation involving HIO_x. … (B) $J_{1.7}$ versus (HIO_3 + H_2SO_4) × HIO_2 for all measured nucleation events, together with CLOUD. Dashed line in (B) is parallel to and has the same distance to the regression lines of the $J_{1.7}$ and (HIO_3 + H_2SO_4) × HIO_2 at the temperature of 10 and -10°C. Assuming a linear relationship, this line represents what would be anticipated of 0°C. Pink and green data points in (B) represent average values during each nucleation event. Error bars represent one standard deviation of the mean value. The hollow circle in the figure represents the HIO_2 values estimated based on the fitting equation shown above. See Methods 3 and Supplementary Text 3 for estimates of uncertainties in concentration measurements and nucleation rate, and limits of detection.

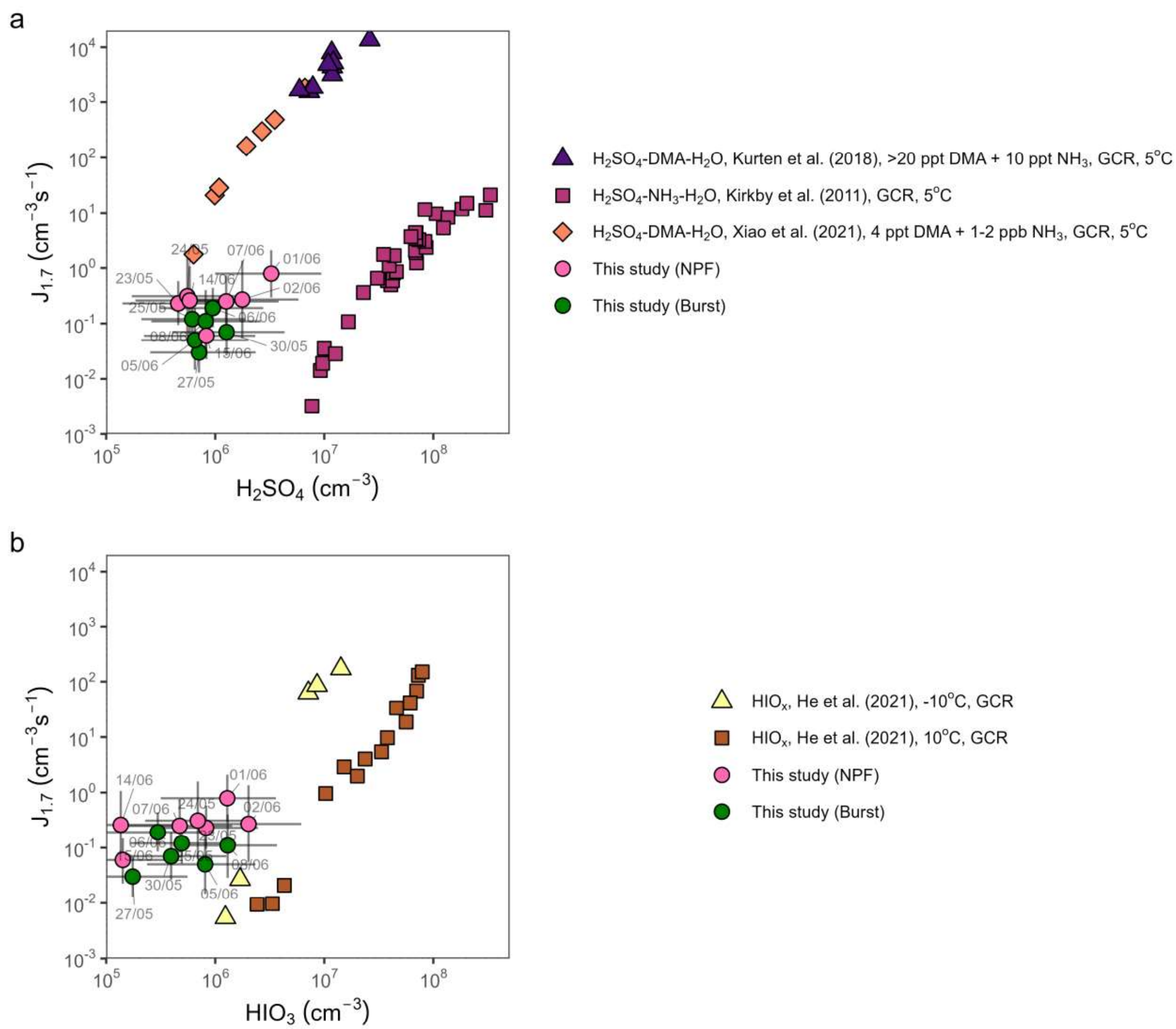

Figure S8: Comparison of nucleation rates for the H_2SO_4, and HIO_3 systems with previous studies [9,10]. (A) Particle formation rates $J_{1.7}$ plotted against H_2SO_4 concentrations. Data points from CLOUD chamber experiments represent H_2SO_4-DMA-H_2O with **>20 pptv** DMA and 10 pptv NH_3 (purple triangles) [9], H_2SO_4-DMA-H_2O with 4 pptv DMA and 1-2 ppbv NH_3 (orange diamonds) [11], and H_2SO_4-NH_3-H_2O (magenta squares) [9]. ~~The H_2SO_4-DMA-H_2O data from Almeida et al. [12] are presented without the self-coagulation correction later described by Kürten et al. [13]; this correction is not available for these specific low-concentration experiments and is negligible given the low particle concentrations, and the experiments of Almeida et al. are likely the most representative of our measured conditions.~~ (B) Particle formation rates $J_{1.7}$ plotted against HIO_3 concentrations. Data points are from CLOUD chamber

experiments at 10 °C (yellow triangles) and –10 °C (brown squares) [10]. For both plots, pink circles represent NPF events for this study, while green circles denote burst events. Each data point is labelled on the plot using the day/month

And we make the following additions to our methods when we discuss our particle counting instruments:

"… and estimate a 30% relative uncertainty in our quantification of particle number related to both uncertainty in measurement, and uncertainty associated with harmonizing measurements from these different instruments."

When we discuss our CIMS data:

Uncertainties in CIMS measurements are derived from chamber experiments and subsequently adapted for field conditions. For inorganic acids, while CLOUD chamber studies estimate sulfuric acid uncertainties at approximately a factor of 2 (+100%/−50%) [31], we adopt a conservative factor of 3 (+200% / -67%) to account for the additional inlet turbulence and environmental variability inherent to ship-borne measurements.

The uncertainty in OOM measurements arises from several compounding physical sources, including the application of the sulfuric acid calibration coefficient, mass-dependent transmission efficiency, sampling line losses, and the charging efficiency of OOMs by nitrate ions. Because the calibration factor *C* is estimated based on the collision-limited sensitivity of H_2SO_4, it provides a lower-limit concentration for species charged less efficiently, such as OOMs [78]. Chamber studies reflect this limitation, suggesting ELVOC uncertainties of a factor of 1.8 (+80%/−45%), whilst acknowledging that less oxidised species (LVOCs/SVOCs) may be underestimated by up to a factor of 4 (+300% / -75%) due to lower charging efficiencies [71]. To comprehensively encompass these errors, we apply an overall uncertainty of a factor of 5 (+400% / -80%) to the total measured OOM concentrations.

For the comparisons with CLOUD chamber data uncertainties were treated as log-normally distributed to prevent unphysical negative uncertainty. The systematic instrument uncertainties (factor of 3) for the quantification of HIO_x and H_2SO_4 were converted to relative fractions and combined in quadrature with the relative atmospheric standard deviation of the measurement for each nucleation event ($\frac{\sigma_{std,X}}{X}$).

For each individual species (Figure S8) the total relative fractional uncertainties (δ_{tot}) were calculated as:

$$\delta_{tot} = \sqrt{(\ln 3)^2 + \left(\frac{\sigma_{std,X}}{X}\right)^2}$$

For the multi-component plot (Figure 3b, $X = HIO_2 \times (H_2SO_4 + HIO_3)$), because the systematic uncertainties of the individual components are derived from the same instrument, they were treated as systematically correlated. The log-space errors compound linearly, resulting in a combined systematic variance of $2ln(3)$. This was combined in quadrature with the relative atmospheric standard deviation using the Gaussian formula:

$$\delta_{tot} = \sqrt{(2\ln 3)^2 + \left(\frac{\sigma_{std,X}}{X}\right)^2}$$

The absolute upper and lower bounds for all vapour concentrations were then determined by multiplying and dividing the total value X by the respective geometric error factor exp (δ_{tot}). This gave an average of a factor of 8.4 uncertainty on the x-axis of Figure 3b.

… And in the discussion of J values:

Because $J_{1.7}$ were inferred rather than measured directly, uncertainties were propagated through the survival probability framework following Cai et al. (2022) [94]. The overall uncertainty for $J_{1.7}$ comprises the baseline uncertainty in J_4 (factor of 2, see above). The errors regarding CoagS were approximated to be equivalent to the errors equivalent to quantification of particle number (30%) and those relating to the growth rate to be 50%.

Due to the exponential nature of the survival probability scaling, the resulting uncertainties are log-normally distributed. The relative systematic uncertainty ($\delta_{sys,J}$) was calculated using the Gaussian error propagation formula, combining the log-normal variance of J_4 (ln 2) with the fractional variances of the sink and growth rate:

$$\delta_{\mathrm{sys,J}} = \sqrt{(\ln\ 2)^2 + \mathrm{E}^2\,[\,0.30^2 + 0.50^2\,]}$$

Where E is the integrated ratio of the coagulation sink to the growth rate across the 1.7 to 4 nm interval ($E = \Delta d_p \frac{CoagS}{GR}$). To determine the total fractional uncertainty ($\delta_{tot,J}$) visualised as error bars in Figure 3b and Figure S8, this systematic error was combined in quadrature with the relative standard deviation ($\frac{\sigma_{std,J}}{J_{1.7}}$) of the measurement period:

$$\delta_{tot,J} = \sqrt{\delta_{sys,J}^2 + \left(\frac{\sigma_{std,J}}{J_{1.7}}\right)^2}$$

Using this methodology, we estimate our uncertainty in our J values to be approximately a factor of 3.2 This falls within the uncertainty range of factor 2–5 reported in recent polar aerosol studies [98].

Comment 2: The authors emphasize that their measurements provide direct in situ evidence that HIOx/H2SO4 clustering enhances nucleation rates in the real atmosphere, thereby validating the CLOUD results of He et al. (Science, 2023). Upon first reading, this claim led me to expect either (i) formation rates (J1.7) comparable to those reported by He et al. , or (ii) the observation of clustering behaviour analogous to that reported in the CLOUD experiment. However, the results section shows that the J1.7 values reported in this study (at a mean temperature of ~1.2 °C) are in fact higher than would be expected from iodine oxoacid/H2SO4 nucleation alone at this temperature and are instead comparable to the J1.7 values reported by He et al. at −10 °C (Fig. 3b). The authors interpret these elevated formation rates as likely resulting from additional enhancement by DMA and/or oxygenated organic molecules (OOMs). While this interpretation is plausible, it implies that the observed nucleation cannot be attributed to HIOx/H2SO4 clustering alone, and therefore does not constitute a direct confirmation of the CLOUD results.

The second line of evidence used to support the authors' claim is the mass defect (MD) analysis shown in Figures 2 and S6. However, these plots reveal only two mixed iodine–sulfuric acid cluster (HIO3HSO4- and HIO2·H2SO4·HSO4-) which appears insufficient to support a claim of direct observational proof of HIOx/H2SO4 -driven nucleation. This limitation is associated with the fact that the MD plots are derived solely from nitrate CIMS measurements of neutral clusters. An observation of a broader suite of mixed sulfuric acid–iodine oxoacid clusters (which are typically detected as naturally charged clusters using an APi-TOF) is what in fact would give the direct molecular evidence HIOx/H2SO4 -driven nucleation. For example, the CLOUD study by He et al. shows clear sequential clustering of sulfuric acid–iodine oxoacid species extending to masses exceeding 600 Th. Similar sequential growth of iodine-containing clusters to high molecular weights has also been reported in Arctic field measurements at Villum by Beck et al. (2021; their Fig. 3) and by Baccarini et al. (2020; their Fig. 1). These studies reported multiple mixed iodine–sulfuric acid clusters, including HIO3HSO4SO3-, HIO3HIO2IO3H2SO4-, and (I2O5)1-2HSO4- . Baccarini et al. further reported clusters such as I2O5HIO3HSO4- , (I2O5)2HIO3HSO4- and (I2O5)3-4HSO4-.

Therefore, while the present study is indeed original in reporting J1.7 measurements and comparing them to the CLOUD measurements, it is not the first to provide in situ evidence of HIO_x/H_2SO_4 clustering in the atmosphere. Such clustering has already been observed in previous field studies. The authors' claims on validating CLOUD measurements, should therefore be rephrased to align with the existing literature and to more accurately reflect the specific novelty of the present work.

Response: We thank the reviewer for their detailed assessment of our data. We would like to respond to the points about the interpretation of what constitutes validation of a chamber study, and the findings from previous Arctic field campaigns. We address their two primary lines of reasoning below.

Firstly, regarding the observed formation rates ($J_{1.7}$), we note that ambient field studies cannot reproduce the exact controlled conditions of chamber experiments. Rather than being a limitation, this reflects the added chemical complexity of the ambient Arctic boundary layer, where a wider range of trace species (e.g. amines and OOMs) are present compared to the simplified HIOx/H2SO4 system investigated in CLOUD experiments.

We appreciate the reviewer's suggestion to carry out a more detailed error analysis. This has helped clarify that our measurements are quantitatively consistent, within uncertainty, with the fundamental HIOx/H2SO4 mechanism reported by He et al. (2023).

Taken together, this suggests that the baseline chamber-derived mechanism remains relevant under ambient conditions, and that our observations provide an in situ validation on its operation within a more complex atmospheric environment.

Secondly, regarding the prior observation of mixed sulfur-iodine clusters. Baccarini et al. did report clusters of iodine-containing and sulfur-containing acids in the SI and they did not discuss this finding in the main paper. Similarly, Beck et al. do not publish the chemical composition of their identified peaks, but we have checked the m/Q values from their dataset on Zenodo and note that, in their "HIO3/I2O5" category of clusters, there are three peaks which match $HIO_3H_2SO_4$ (272.857), and $HIO_2(HIO_3)_{2\text{-}3}H_2SO_4$ (608.668 and 686.597). We acknowledge this fact as follows in our main text:

"These clusters are similar to those identified in previous Arctic studies of mixtures of sulfuric acid and iodine oxoacids [13,14]."

These studies were published before He et al. (2023) so it is not possible for them to compare with CLOUD data for an estimate of the role of HIOx/H2SO4 nucleation.

Finally, regarding the APi-ToF, we argue that the NO_3^- CIMS can reliably quantify monomers that contribute to new particle formation such as H_2SO_4, HIO_3, HIO_2, MSA, OOMs. The NO_3^- CIMS can also quantify dimers (e.g., $HIO_3HSO_4^-$ and to detect clusters up to pentamers (He et al., 2023), or even clusters with over thirty monomers in them, if the concentrations are high enough (e.g., in controlled chamber studies; Kürten et al., 2016). They are used in real atmospheric observation to detect species up to hexamers, as demonstrated in studies such as Yao et al. (2018, *Science*) and Brean et al. (2022, *Nat Geosci*).

In our case, we used the concentration of monomers to predict nucleation rates, which agree well with that of He et al. (2023), indicating that HIO_2 stabilises H_2SO_4 and HIO_3. Then, searching through our mass spectra, we report $H_2SO_4{\cdot}HSO_4^-$, $HSO_4{\cdot}IO_3^-$, $HIO_2H_2SO_4HSO_4^-$,

$HIO_2 \cdot C_2H_7N \cdot IO_3^-$, and $HIO_2 \cdot IO_3^-$. These clusters may be charged forms of neutral dimers and trimers, fragmentation products of larger dimers and trimers, or naturally charged ions (we expand upon this in our response to Reviewer 6, Comment 5). This provides qualitative evidence as to which clusters are present in the atmosphere. For instance, the detection of the dimer $HSO_4 \cdot IO_3^-$ and trimers such as $HIO_2H_2SO_4HSO_4$ and $HIO_2 \cdot C_2H_7N \cdot IO_3^-$ confirms the involvement of four key components—sulfuric acid, iodic acid, and iodous acid—in forming mixed molecular clusters. As we are using qualitative evidence to confirm the presence of different clusters, it does not matter whether what we detected is a real dimer or trimer, or the evaporation product of a larger cluster.

We acknowledge that our analysis is unable to determine the precise stoichiometric ratios of these components in larger clusters (as would be possible with an APi-ToF), but this does not change any of our conclusions. It is important to note that the nucleation mechanisms involving HIO_2, HIO_3, and H_2SO_4 we revealed are consistent with and built upon the results of He et al. (2023) and quantum chemistry calculations (Zu et al, 2024).

While Baccarini et al. (2020) and Beck et al. (2021) used an APi-ToF to detect larger naturally charged atmospheric clusters, their analyses are qualitative. We firmly believe that our approach is robust; although incorporating an APi-ToF would strengthen our findings, it would not alter our conclusions, with regards to the formation mechanisms.

Therefore, our study is the first to measure the neutral precursors, identify the relevant clusters, and quantitatively link these precursor concentrations to the observed particle formation rates using the framework provided by He et al. (2023). Therefore, we agree with Reviewer 6 that this work provides the direct in situ validation of the synergistic iodine and sulfur-driven nucleation mechanism. To make this clear, we now state the following in "Sources and processes…":

"Previous Arctic studies have identified H_2SO_4-NH_3 and HIO_x nucleation as key processes, before the discovery of the synergistic behaviour of HIO_x-H_2SO_4. However, the detection of HIO_x and H_2SO_4 in these studies [14,53] including the detection of mixed iodine-sulfur clusters suggests that the HIO_x-H_2SO_4 nucleation process may have been present in their observations. The relative importance of different nucleation mechanisms in the wider Arctic likely depends on season and location (Fig. S1) [54], which controls emission and oxidation processes, and thus the concentration of precursor gases (such as DMS and I_2, Fig. 5) [39]. To better quantify nucleation mechanisms across the Arctic, comprehensive observations at multiple platforms and locations are needed."

Comment 3: In relation to Comments 1 and 2, the reported J1.7 values appear high, given that the combined concentrations of sulfuric acid, iodic acid, and methanesulfonic acid remained below ~5e6 cm-3 during the measurement period. Specifically, the reported formation rates (0.03–0.8 cm-3s-1) exceed those previously reported for Ny-Ålesund, including J1.5 reported by Beck et al. and J3 measured at the Zeppelin station by Lee et al. (2020; https://doi.org/10.5194/acp-20-13425-2020). The authors attribute these elevated J values to the presence of DMA and OOMs; however, the evidence supporting their involvement remains weak. It is understandable that, due to the inherent difficulty of quantifying low DMA

concentrations with a nitrate-CIMS, neither the absolute DMA concentration nor its day-to-day variability during NPF events can be robustly constrained. This limitation makes it difficult to assess whether DMA alone can account for the observed magnitude and variability of J1.7. At the same time, a substantial fraction of OOMs was quantified in this study. Given this, it would be useful to explore more explicitly how these compounds relate to the variability in J1.7. The authors performed a PMF analysis of the OOM measurements and derived a volatility basis set; however, it remains unclear how these PMF factors relate to the measured formation rates. At present, a qualitative inspection of the mass-defect plots, together with comparisons of J across different days, does not reveal a clear link between enhanced cluster abundances and higher nucleation rates, despite Fig. 2 trying to suggest that nucleation is associated with high abundance of clusters. In particular, days with elevated cluster signals do not necessarily correspond to higher J values. Have the authors attempted to correlate the PMF factors or volatility classes directly with the measured J values?
Finally, the NPF event on 14 June appears peculiar: although its J value is comparable to those of other events, the mass-defect plot shown in Fig. S6 exhibits substantially fewer clusters than on other nucleation days. The authors are encouraged to comment on possible reasons for this behaviour and its implications for the proposed nucleation mechanism.

Response: First, we agree with the reviewers' assessment with regards to uncertainties (see response to your comments 1 & 2, and Fig. R5). On the merit of these uncertainties, we find that all of our datapoints do indeed agree with He et al., and argue that:

"Of all systems that have been studied at the CLOUD chamber, the HIO_x-H_2SO_4 system most consistently reproduces our $J_{1.7}$ values, indicating the key role of both HIO_x and H_2SO_4 in our observed nucleation events".

Second, while Fig. R5 does indeed show that our uncertainties mean that our data is well explained by the HIOx-H2SO4 synergistic nucleation proposed by He et al. (2023), the central value of our observations sit slightly above CLOUD observations. We do agree that it is not possible for us to evaluate whether DMA can explain such enhancement. However, given the huge complexity in the real atmosphere, the challenge is anticipated. The role of DMA and HIOx in nucleation, in different concentration ranges, would make a great subject of future research, e.g., in CLOUD chamber.

"Following He et al. [17], we plotted $J_{1.7}$ versus (HIO_3 + H_2SO_4) × HIO_2, representing the formation rate of H_2SO_4-HIO_2 and HIO_3-HIO_2 (Fig. 3B). ~~While our $J_{1.7}$ measurements are comparable with those observed for the HIO_x-H_2SO_4 and HIO_x-H_2SO_4-NH_3 in CLOUD chamber experiments at -10 °C within uncertainties for most days,~~ ~~**the central values of our observations sit slightly what we would anticipate based on CLOUD.**~~ Of all systems that have been studied at the CLOUD chamber, the HIO_x-H_2SO_4 system most consistently reproduces our $J_{1.7}$ values, indicating the key role of both HIO_x and H_2SO_4 in our observed nucleation

events. Considering the average ambient temperature of 1.4 °C during nucleation periods, the central value of our $J_{1.7}$ values on some days **sit slightly above our interpolated fitting line based on** ~~are higher than anticipated from~~ the temperature dependence measured in the CLOUD chamber [17] (at -10 and +10 °C). **This enhancement is likely driven by two key factors: reduced cluster scavenging and** the potential participation of other stabilising species. **Firstly, our condensation sink was lower than in the CLOUD chamber, which can cause deviations due to differences in cluster scavenging [37].** Second is the potential participation of other stabilising species. One plausible contributor is DMA, as evidenced by the occasional detection of DMA clustered with HIO_x (Fig. S4 and Extended Data Figures 3-4). The DMA-HIO_2-HIO_3 cluster signal correlates with HIO_3 concentrations and is highest when HIO_3 is at its maximum (Figs. S4-5). Quantum chemical modelling studies also showed that DMA can accept a proton from HIO_3 as a base, forming a stable cluster (DMA-HIO_2-HIO_3) [38]. OOMs may also enhance nucleation rates [39], however, we see no positive relationship between OOM concentrations (of any volatility class or source) and enhanced nucleation rates relative to He et al. [17] (Fig. S6). This indicates that the OOMs we observed do not appear to have a substantial enhancing effect."

Third, below we include a figure (in the manuscript as Figure S6) where we colour Figure 3b by our four PMF factors, as well as different volatility classes of OOMs. These results do not indicate that higher OOM concentrations, split by volatility nor PMF factors, seem to explain our elevated J values relative to CLOUD. We amend our manuscript as follows:

"…The role of DMA in enhancing nucleation rate is corroborated by quantum chemical modelling studies showing that DMA can accept a proton from HIO_3 as a base, forming a stable cluster (DMA-HIO_2-HIO_3) [13]. **Despite the observed OOMs, we see no positive relationship between OOM concentrations of any volatility class or source, and enhanced nucleation rates relative to He et al. (2023), indicating that our observed OOMs do not appear to have a substantial enhancing effect on nucleation rates (Fig S6).**

~~Furthermore, given the high abundance of OOMs, particularly ELVOCs (Fig. 1C), it is also plausible that they contribute to the enhancement of nucleation rate, either by participating directly in nucleation or by stabilising the initial inorganic clusters. This would be consistent with studies from other environments showing OOMs can enhance nucleation rates [38]. Furthermore, as the volatility-reducing effect of iodine in our observed I-OOMs is not fully constrained, it is possible that some of the observed I-OOMs are ULVOCs, which may participate in the initial nucleation.~~

Our results demonstrate that H_2SO_4, HIO_3, and HIO_2, are the core components of a synergistic nucleation mechanism in our study area and this process appears to be potentially enhanced by the presence of bases such as DMA, and by OOMs. This provides real atmospheric validation of the multicomponent nucleation mechanism identified by He et al. [17], at lower vapour concentrations than measured in CLOUD (Fig. 3). ~~and further suggests that in complex ambient environments,~~

this process is likely modulated by amines and organic vapours. The precise contribution of each warrants further investigation. "

Finally, with regards to J values on 14 June, we propose three possible reasons. Firstly, following the response to Reviewer 6 comment 1 we argue that the CS for this event was very low (0.0003 s^{-1}) which will elevate the J value at any given monomer concentration due to less efficient scavenging of intermediate clusters. Secondly, the HIO_2 was also low, but the H_2SO_4 was relatively high. If HIO_2 is low (the stabilising base) then the signals of clusters containing this base will be low. It is therefore possible that other stabilising bases are present on this day, but this is not substantiated by the data, and we would argue this uncertainty is a common feature of any field measurement. Finally, the error analysis you recommended (comment 1) also suggest a high uncertainty in J values and acid concentrations, and the results look more aligned with He et al. (2023) given such uncertainties.

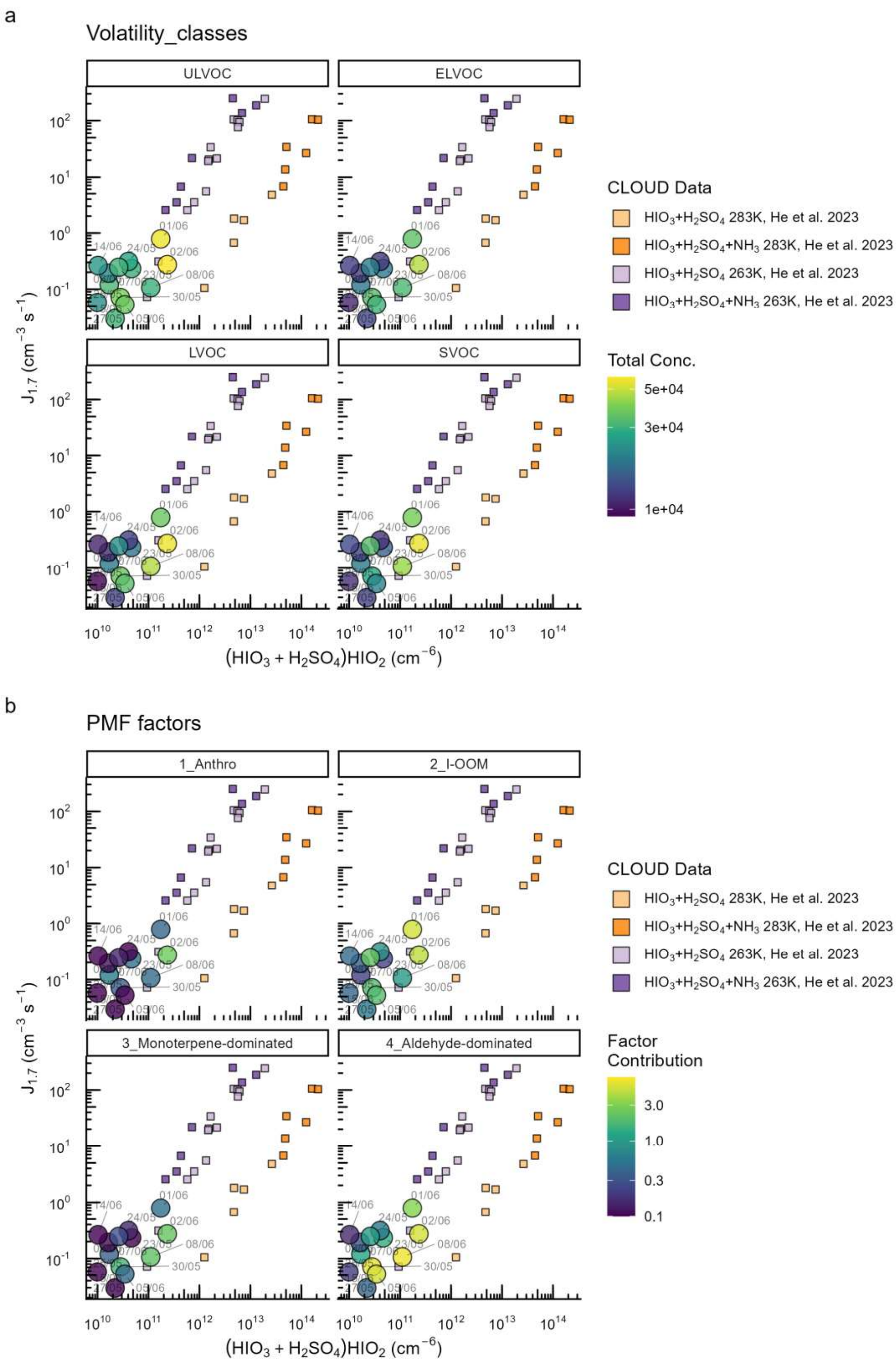

Figure S6: As Figure 3b, coloured by (a) different volatility classes of OOMs & I-OOMs, and (b) different PMF factors. We see no relationship between the enhancement to NPF relative to He et al. (2023) and our observed OOMs & I-OOMs.

Comment 4: In relation to particle growth and the growth modelling presented in Figure S17 and partly in Figure 4, the authors conclude that oxygenated organic molecules (OOMs), including iodine-containing OOMs (I-OOMs), enhance particle growth to sizes relevant for CCN. However, the modelled growth rates based on acid and organic condensation are, in most cases, unable to fully reproduce the measured growth rates. While this discrepancy may partly arise because growth rates derived from particle number size distributions represent apparent growth rates rather than true condensational growth rates, an additional mechanism may also contribute to the enhanced growth observed. In particular, Huang et al. (2022) [https://doi.org/10.1073/pnas.2201729119] demonstrated that iodine-oxide nanoparticles exposed to organic vapours can undergo accelerated growth through heterogeneous reactions. Could the authors discuss whether such heterogeneous chemistry involving iodine-containing particles and organic vapours may contribute to the enhanced particle growth observed here, especially during periods when the modelled condensational growth underestimates the measured growth?

Great point. We thank the reviewer for this suggestion. We do agree that additional mechanisms may also contribute to the enhanced growth observed. In our manuscript, we state the following

"Other factors, such as multiphase reactions and coagulation growth, as well as potential change in meteorological conditions may also explain the discrepancies between observed and modelled GRs (see Supplementary Text 6)."

And in supplementary text 6:

"At diameters larger than 25 nm, a role of solute phase chemistry with multiphase processes including salt formation, oligomerization, **and other heterogeneous reactions** in particle growth becomes possible, which is not dealt with in the model [125,126]. We neglect coagulation growth, although this will be a relatively small contributor to overall growth. We also presume that particle-phase reactions cause no change in partitioning, although these reactions are relatively fast and likely result in a net decrease in evaporation rates of condensed phase OOMs [127]."

Where ref. 126 is Huang et al. (2022).

The mechanism proposed by Huang et al. (2022) provides a compelling explanation for accelerated growth in pure iodine-oxide systems. We are unsure whether we are able to extrapolate such laboratory results to the present observations for several reasons:

Huang et al.[5] utilised pure iodine-oxide seeds in a controlled environment. Our observations (evidenced by Figure 4 and the AMS PMF analysis) show that growing particles in the Arctic are chemically complex and become rapidly dominated by organic mass. As noted by Yli-Juuti et al. (2020)[6], there are open questions regarding how particle-phase reactions and non-ideal mixing influence growth in such multi-component systems. The efficiency of heterogeneous reactions on these organic-rich surfaces, as opposed to pure iodine-oxide is unknown.

Our modelled growth rates already account for the condensation of a wide suite of OOMs. Given that the NO_3^- CIMS is less sensitive to semi-volatile organic compounds (SVOCs), which are known to be able to condense on particles, we argue that they contribute to the discrepancy between measured and modelled growth rates. This is consistent with the "orders-of-magnitude" uncertainties inherent in converting molecular composition to volatility information for complex mixtures [6,7]. Acknowledging all of this, we add the following to the supplementary text where we discuss the growth modelling in detail

"While our model captures the primary drivers of growth, we acknowledge that complex multiphase chemistry such as the heterogeneous reactions between organics and iodine-oxide seeds [122] may contribute to growth during periods of model underestimation. However, the precise role of such reactions in organic-dominated, multi-component Arctic particles remains an open question [123] and warrants further targeted study"

Where ref. 126 is Huang et al. (2022) and ref. 128 is Yli-Juuti et al. (2020; https://doi.org/10.1038/s42004-020-00339-4)

Finally, we agree that the measured growth rate represents an apparent rate as the sum of coagulational and condensational growth (alongside any jumps in D_p due to changing airmasses). However, the contribution of coagulation to this apparent growth is minimal given the low ambient particle number concentrations in this pristine Arctic environment.

Comment 5: The authors mainly attribute the enhanced particle growth observed on 8 June to elevated aldehyde concentrations measured by the PTR-MS (Lines 342-344 and the discussion of Fig. 4: Lines 304-323). However, the PMF analysis of the nitrate-CIMS OOM data suggests a different interpretation. Specifically, inspection of the PMF factor time series in Fig. S2c (and Fig. S13) shows that the increase in the aldehyde-rich factor on 8 June coincides with a relatively stable particle mode diameter, indicating limited subsequent growth. In contrast, during periods dominated by the monoterpene-related factor, particles continue to grow to diameters exceeding 100 nm. This apparent discrepancy between the nitrate-CIMS-based PMF results and the PTR-MS gas-phase VOC measurements warrants careful consideration, as it leads to differing conclusions regarding which compound classes are primarily responsible for growing the particle up to CCN size.

Response: On the 8th June, the Aldehyde-dominated factor reaches a concentration >10^7 cm^{-3} at 08:00, while the monoterpene-dominated factor reaches a concentration >10^7 cm^{-3} at 14:00. The growth rate is consistent across the daytime of the 8th June (R^2 of a linear fit between measured particle diameter and time between 08:00 and 22:00 = 0.956), indicating that both sources contribute roughly equally to particle growth on this day. On average, the aldehyde-dominated factor is more abundant, and has a lower volatility. We do agree that these sources are, all things considered, quite similar in behavior with regards to particle growth. We acknowledge this as follows:

"~~The high aldehyde to monoterpene ratios, up to 140 (v/v) indicate significant sources of abiotic OVOC emissions for OOM formation in this study region~~ **While the aldehyde to monoterpene ratios are high (up to 140 *v/v*, Fig. 4E), PMF analysis of the CIMS data (Extended Data Fig. 8, Fig. S7) indicates that both aldehyde- and monoterpene-derived OOMs were highly abundant during this period**."

Specific Comments:

Comment 6: L49-52: "Our findings demonstrate that iodine, sulfur, and organic precursors in the Arctic enhance the abundance of CCN, through new particle formation, that can potentially influence the earth's cloud cover, radiation balance, and hydrological cycle." The authors should consider tempering this claim by noting that, while the results indicate a potential enhancement of CCN abundance, the magnitude of this effect and its climatic relevance is yet to be quantified.

Response: We agree that the climatic relevance of the observed CCN enhancement cannot be quantified from our dataset alone. We have therefore tempered the statement to clarify that our results indicate a *potential* increase in CCN abundance, while the magnitude of the effect and its implications for clouds and climate remain to be determined. The revised text:

"Our findings demonstrate that iodine, sulfur, and organic precursors in the Arctic can enhance CCN abundance through new particle formation, highlighting a potential **but unquantified** pathway for influencing cloud cover, radiative balance, and the hydrological cycle."

Comment 7: L122: Figure 1. Please clarify the criteria used to select the events displayed in Panel A and explain why the remaining events were omitted, or include all of them.

Second, it is difficult for the reader to relate the events shown in Figure S2 to those presented in Figure 1, as the map does not provide any information on the timing of the measurements. To improve clarity, the authors could consider encoding the measurement date using a colour scale, while using marker shape to distinguish between NPF and burst events, rather than using a single fixed colour for event types.

Third, for readers who may not be familiar with Arctic geography, it would be helpful to indicate the Davis Strait marginal sea-ice (melting) zone on the map.

Response: First, we agree and have discussed the criteria in detail in Methods section 10, where the criteria are discussed:

"This study focuses on a 24-day period, from 23rd May to 15th June. The broader expedition encompasses a total of 38 days, which includes sailing time across the North Atlantic Ocean (from 19th to 22nd May) and the return trip to Southampton (from 16th to 26th June). We have identified three distinct event types that will be the primary focus of our analysis based on the established criteria [92-94]. These event types are:

- New Particle Formation (NPF) Event: These events are characterized by the formation of small particles, notably in the 3–5 nm range. Such events typically exhibit minimal or no pre-existing particles within the smallest size categories at their onset and continuous growth for at least several hours. The particles usually grow to a diameter exceeding 20 nm.

- Burst Event: These events are marked by the formation of small particles. There is a noticeable increase in ion numbers within the 3-5 nm range; however, these particles do not exhibit subsequent growth.

- Non-event Event: These observations are characterized by an absence of both particle nucleation and growth.

N.b., days that are heavily influenced by the ship's own emissions are excluded in this category."

Second, individual maps are presented in Extended Data Fig. 10

As for the third point, because Figure 1A is visually dense, we provide a zoomed-in view of the Davis Strait marginal sea-ice (melting) zone in Figure 4A.

Comment 8: L137-140: "Background concentrations of NOx (<50 ppt), SO2 (median 23 ppt) and BC (1.9 ng m-3) were very low, indicating pristine marine conditions." These values are difficult to verify from Figure S3 as currently presented. Please revise the figure so that the stated concentrations can be clearly identified.

Response: We have revised the SO_2 scale. We note that these reported values represent average/median statistics of the background concentration which cannot be reliably inferred by visual inspection of Figure S3 alone. We therefore provide the raw dataset in Zenodo used for these calculations to ensure transparency and reproducibility.

Comment 9: L140-141: "Based on the criteria detailed in Methods 10, nucleation events were observed on 13 days…" What does 10 (after Methods) stands for?

Response: Methods 10 refers to the Methods section #10 (at P26), where we describe how nucleation events were classified.

Comment 10: L188-190: "Using this relationship, we applied the more readily observed HIO3 to estimate HIO2 concentrations when they were not detectable on 23rd and 24th May (see Fig. S2)." Why is HIO2 not detectable on those days given that it was detectable in 10/06 at even lower HIO3 concentration?

Response: A few factors can affect the detection limit of the NO_3^- CIMS, as discussed in Methods Section 3. In the case of these days, there was a large adjacent peak which made unambiguous resolution of the HIO2 ion impossible. Further discussion of this is present in SI section 3.

Comment 11: L 191: Figure 3. It would greatly improve readability if the colours of the markers referring to the CLOUD data were swapped, such that blue represents −10 °C and red represents +10 °C, rather than the opposite. This colour convention is more intuitive and would reduce the potential for confusion (I initially misinterpreted the data until reading the legend).

Response: We have swapped the colors in Figure 3: now blue represents −10 °C and red represents +10 °C.

Comment 12: L205-207: "While J1.7 has not been reported elsewhere around the Arctic, our observed formation rate at 10 nm (J10) is within the observed range 10, although the median is relatively high (Fig. S7)."

Formation rate measurements at smaller particle diameters have previously been reported for Ny-Ålesund. In particular, Beck et al. reported formation rates at 1.5 nm while Lee et al. (2020) (https://doi.org/10.5194/acp-20-13425-2020) reported formation rates at 3 nm. A brief comparison of the present results with these studies would provide more insight into the underlying NPF processes than a comparison limited to J10 across multiple Arctic sites.

Response: We thank the reviewer for this suggestion. We agree that comparison with formation rates measured at smaller particle diameters can provide useful process-level information for Ny-Ålesund. Beck et al. provide data for four events. For the events on the 5th and 6th August, the J1.7 values don't emerge beyond zero. For the events on the 4th and 8th May, the values during the NPF event are 0.15 and 0.05 $cm^{-3}s^{-1}$ respectively. In our manuscript, we include the following line

"The peak hourly formation rate at 1.7 nm ($J_{1.7}$) for each day varied from 0.04 to 1.4 cm^{-3} s^{-1}, ~~While $J_{1.7}$ has not been reported elsewhere around the Arctic~~ **in line with values at Svalbard of 0.33 ($J_{1.5}$)[13] and 0.04 (J_3)[31]"**

Where ref. 13 is Beck et al., and ref. 31 is Lee et al (https://doi.org/10.5194/acp-20-13425-2020)

Comment 13: L 211: "Our experiments similarly do not match experiments with 4 pptv DMA and 10 ppb NH3" The ammonia unit is incorrect

Response: We have changed it into pptv.

Comment 14: L350: Figure 4 Panel A:

- Although the back trajectories seem to converge at a certain point, it would improve clarity if the ship position is marked clearly on panel A.
- Remember to add the symbol "A" to this panel.
- Panel F please indicate directly on the legend that the IOA trace is a PMF factor

Response:

- We now overlay the ship track on top of the trajectories for visibility

- "A" is added in panel A of Figure 4.
- We have indicated the IOA trace from a PMF factor via the caption for Panel F in the manuscript, as shown below:

"**(F)** Concentrations of organic and inorganic species measured by the aerosol mass spectrometer (AMS). Iodine-organic aerosol (IOA) AMS PMF factor is multiplied by 10000 times, due to low AMS signals of iodine containing fragments (see Methods 12)." ''

Comment 15: L521: "Utilizing the data from the three MPSS systems, we produced a conjoined PNSD for the entire" Please include to the methods section an explanation detailing how the NAIS particle size distributions were scaled to match the nano-MPSS data. It would also be helpful to report the range of scaling factors applied during the campaign and to comment on their variability and potential impact on the derived formation rates as requested earlier.

Response: Thank you! Our procedure for matching the distributions is as follows

1. Plot the size distributions of the Nano-SMPS, NAIS, and Long-SMPS
2. For the regions where there are overlap, calculate the ratio between NanoSMPS and NAIS, and LongSMPS and NAIS
3. Correct the NAIS and NanoSMPS to the LongSMPS data in the regions there are overlap (in this case, we multiplied the NAIS and NanoSMPS down).
4. Plot the individual size distributions to ensure the instruments match one-another for the entire measurement period

We regularly follow this procedure for our instruments, as we find that the NanoSMPS and NAIS tend to "over-read" particle number counts. The correction factors are 1.38 for the NanoSMPS and 1.2 for the NAIS.

Because these scaling factors were static rather than variable, their overall effect on the derived particle formation rates is relatively small. We are confident in the accuracy of these specific corrections, as they were validated against a reference CPC during deployments immediately preceding and following this cruise.

We add the following to the methods:

"To harmonise the PNSDs across the instruments, the NAIS and Nano-SMPS data were scaled to match the Long-SMPS measurements within their respective overlapping size regions. As the NAIS and Nano-SMPS routinely over-report particle counts, we applied constant reduction factors of 1.20 and 1.38, respectively, across the entire campaign to align them with the Long-SMPS. The stability of these static scaling factors was validated using reference condensation particle counter data from deployments immediately preceding and following this cruise. We estimate a 30% relative uncertainty in our quantification of particle number related to both

uncertainty in measurement, and uncertainty associated with harmonizing measurements from these different instruments..”

Comment 16: L 530-532: “In parallel, nitric acid vapor is volatilized from a glass tube containing liquid HNO3 and carried by a mixture of 3 cm3 min-1 N2 and 20 L min-1 pristine Arctic ambient air as a sheath gas”.

Is this correct? Did you use ambient "uncleaned" air as sheath flow? If yes please explain how you made sure that this air is free of contaminants?

Response: We thank the reviewer for raising this important point. We did indeed use unfiltered ambient air, which was a conscious choice. While commercial zero air generators are often used for sheath flow, they are notoriously difficult to render completely amine-free and frequently introduce more amine contaminants into the system than they remove. Given the pristine and low-humidity nature of the Arctic atmosphere, we determined that ambient air would provide a cleaner sheath flow.

To rigorously assess the risk of a local amine source interfering with our measurements, we simulated the formation kinetics of the C2H7N-HIO2-IO3 cluster within our inlet. Assuming a system containing solely HIO2 and HIO3 that is suddenly exposed to DMA, we calculated the cluster production over the 200 ms contact time between the sheath and sample flows. As shown in Figure R3, our calculations indicate that even if the sheath air contained an unrealistically high concentration of 100 ppt DMA, it would produce only ~20 cm^{-3} of the C2H7N-HIO2-IO3 cluster. This brief residence time prevents meaningful artifact formation, giving us high confidence that our sheath air choice did not contaminate the measurements. We will add a brief summary of this rationale to the methodology section of the revised manuscript.

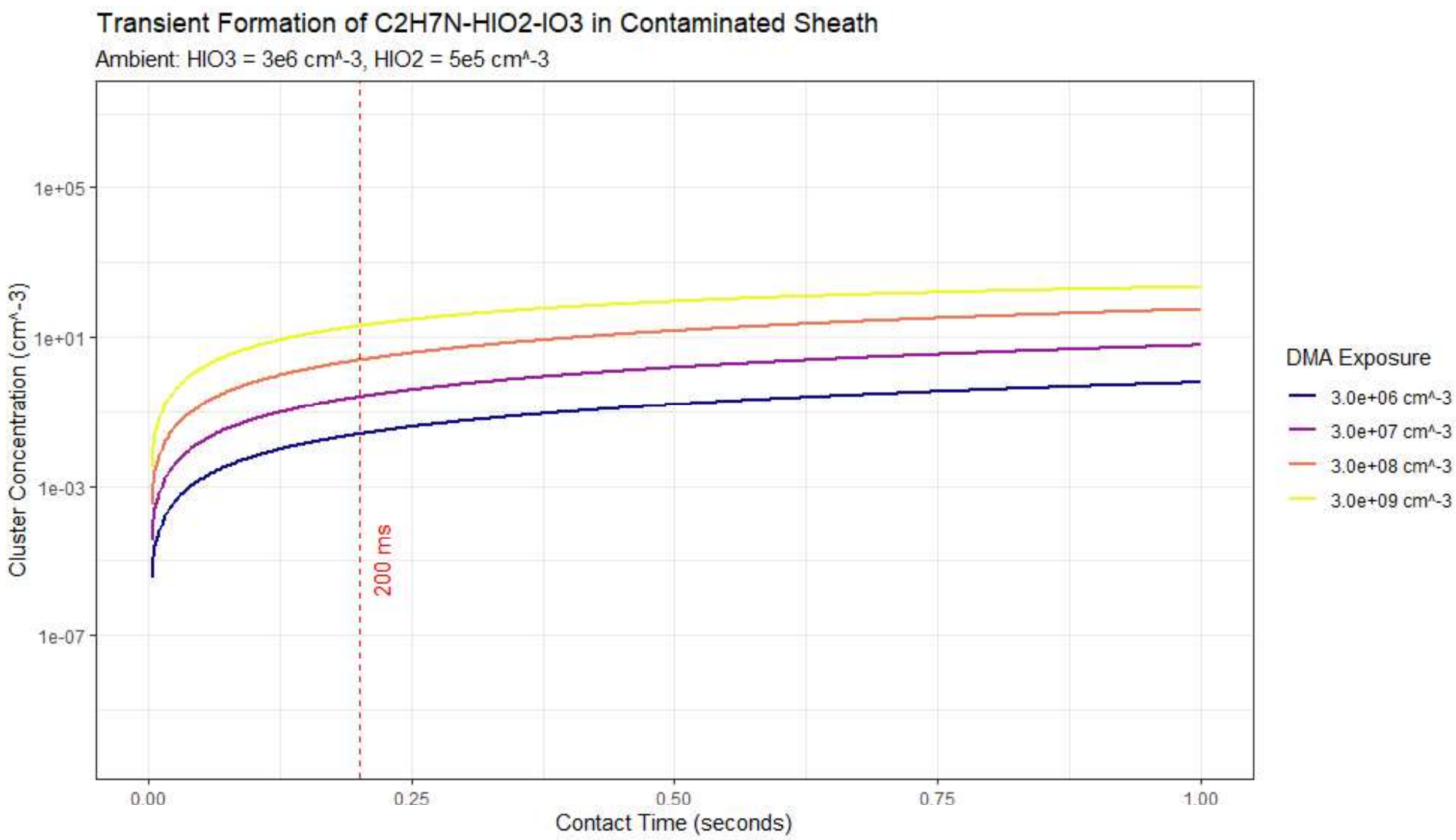

Figure R3: Simulated cluster formation for DMA-HIO2-HIO3 at different DMA concentrations in our inlet.

Comment 17: L 555: Reference should be made to figure S9 instead of Figure S10

Response: We have revised it.

Comment 18: L 834-835: The equation should be $2\pi D$ instead of $4\pi D$ when dp refers to the diameter of the particle rather than the radii. This was also a typo in the Kulmala et al protocol paper.

Response: We have revised it.

Comment 19: L 847: " Growth rate (GR) calculation" It should be emphasized that the growth rates reported here represent apparent growth rates rather than true condensational growth rates. This is especially important given that the measurements were conducted onboard a moving ship. Growth rates inferred from diameter–time evolution in general reflect the net contributions of many processes that affect the modal diameter, including vapour condensation, coagulation, continuing nucleation and changes due to transport and dilution of spatially inhomogeneous aerosols.

Response: We have updated the methodology section to explicitly state that our reported values represent apparent growth rates rather than true condensational growth rates. Furthermore, we have added text detailing how these inferred rates reflect the net contributions of condensation, coagulation, continuous nucleation, and the transport and dilution of spatially inhomogeneous aerosols inherent to mobile shipborne sampling. We include the following

"Because these measurements were conducted on a mobile shipborne platform, the inferred apparent growth rates reflect the net contribution of multiple processes, including vapour condensation, coagulation, continuous nucleation, and the transport and dilution of spatially inhomogeneous aerosols"

Comment 20: L 912: "performed in the PMF2 program" Does this refer to the EPA PMF program? If yes, why was an older version without a robust uncertainty analysis used instead of the later releases?

Response: No, we use PMF2.exe (specifically pmf2wopt.exe), not the EPA PMF program.

Comment 21: L 941-945: "The optimal solution was identified as a seven-factor model describing different kinds of organic aerosol (OA), consisting of hydrocarbon-like OA (HOA), cooking OA (COA), marine secondary OA (MSOA), sea salt OA, two types of semi-volatile OA (SVOA and SVOA1), and low-volatile OA (LVOA) based on time series, diel patterns, factor profiles (especially by representative markers), and correlation with other measurements in this campaign" Why do the PMF factors referred to here differ from those presented in the main manuscript and in Section 5 of the Supplement?

Response: Because of the diverse chemical datasets collected during this campaign, we conducted three entirely separate PMF analyses, which are discussed in distinct subsections of the main text, and methodology:

1. A PMF model on the CIMS data to identify sources of gas-phase oxygenated organic molecules (OOMs). This is what is referred to throughout the manuscript
2. A PMF model on the AMS data for bulk organic aerosol (OA) source apportionment.
3. A targeted PMF model on the AMS iodine-containing species to distinguish sources of particulate iodine from sea salt.

The text referenced by the reviewer (lines 941-945) refers explicitly to the second model: the AMS OA source apportionment.

Comments on SI Figures:

Comment 22: Figure S2:

- Panel A: while it is clear that the black circles are the fits from the maximum concentration method used to calculate GR, this is not explicityly mentioned in the caption. Please include it
- Panel B is supposed to show the solar radiation as a yellow shading but it is actually not shown
- Panel F: the legend refers to a CCN trace ar 0.7% saturation but this trace is not shown in the plot.
- All panels: Figure S2 shows nine pink-shaded areas corresponding to NPF events and four green-shaded areas corresponding to burst events, whereas the manuscript text and Table S1 refer to eight NPF events and five burst events. The authors should ensure consistent

reporting of event numbers throughout the manuscript, figures, and supplementary material (same comment for Figure S3, S12, S13, S15).

Response: Panel A: We have added the text in the caption

Panel B: We have added it.

Panel F: We have added it.

All panels: The event on 8 June was a burst event, but it was colored pink throughout the manuscript because particle growth was continuously ongoing from 7 June. We have clarified it in the Figure S2.

Comment 23: Figure S3:

- Panel B: The y-axis scale is too large for the data presented in this panel. In addition, the caption refers to both CO and SO2, while only the SO2 time series is shown.
- Panel C: It is hard to see the baseline BC from this plot. Can the y-axis be cut to a certain value as done with the other sub plots or plot the data in log scale.
- Panel D: Remove the unneeded characters ($, ^) from the legend. Adjust the inconsistency between the figure caption and the y label (one refers to a signal while the other refers to a mixing ratio)

Response: Panel B: We have revised it.

Panel C: We have changed the plot in log scale.

Panel D: We have revised the legend.

Comment 24: Figure S4: Reference is made to Extended Data Fig. 1 which does not exist.

Response: We have reordered our manuscript to distinguish between supplementary and extended data figures, and this text now points to the appropriate figure.

Comment 25: Figure S6: It seems the panels are sorted by J1.7 rate instead of date. It would actually be helpful to mark the J and GR measured for these events on the subtitles of the subplots.

Response: The figure S6 is sorted by J1.7 value. We add the following to the caption

"Panels are sorted vertically by descending $J_{1.7}$ (see Table S1 for values)."

Comment 26: Figure S7: The figure caption contains several inconsistencies with the plotted data. Growth rates from other locations are described as being shown in grey boxplots, whereas the boxplots in the figure are coloured. In addition, the DY151 data are indicated as blue boxplots, which is not the case in the figure. The caption also refers to a red line that is not present in the

plots. Finally, the caption mentions a location labelled “TIK,” which does not appear in the figure and may refer to Tiksi; this should be clarified.

Response: Thank you. The caption has been revised.

Comment 27: Figure S15: caption of panel (a) refers to Fig. 3 while the correct figure number is 4.

Response: We have revised it.

Comment 28: Figure S20: The figure caption refers to purple circles, grey lines, and green and pink crosses; however, these elements are not shown in the figure.

Response: We have added the ship track in the figure, and opt to remove mention of circles, lines, and crosses, to keep the figure from feeling too busy.

Du et al., Response to reviewer comments

We have thoroughly reviewed all comments, provided a point-by-point response, and revised the manuscript accordingly. Below, we include the reviewers' comments in black, our responses in blue, and any manuscript text in orange. Where edits are made to a larger section, we will include the full section**.**

Referee comments:

Reviewer #6 (Remarks to the Author):

The authors have convincingly addressed all of my major comments. I recommend publication subject to minor revisions.

Minor comments:

Comment: Lines 235–236: It would be preferable to explicitly state the CS values for both this study and the CLOUD experiments, so that readers can assess the difference quantitatively. It is also worth emphasizing that CS has a non-linear effect on J.

Response: Thanks for the suggestion. This now reads as follows:

"Firstly, our condensation sink was lower than in the CLOUD chamber (9×10^{-4} s^{-1} vs 2×10^{-3} s^{-1}), and given the non-linear dependence of J on CS, this difference likely contributed to the observed deviations."

Comment: Lines 237–241: This issue carries over from the previous revision. The DMA discussion requires additional constraints, consistent with the texts in other parts of the manuscript. The QC data are of interest, but their value is contingent on the reliability of the underlying measurements.

The expanded discussion of the correlation between OOMs and J is very helpful and effectively clarifies OOM's contribution.

We have amended as follows. We agree that the discussion of OOMs and J is useful. Thank you for that suggestion.

"Second is the potential participation of other stabilising species. One plausible contributor is DMA, as evidenced by the occasional detection of DMA clustered with HIO_x (Fig. S4 and Extended Data Figs. 3-4). The DMA-HIO_2-HIO_3 cluster signal correlates with HIO_3 concentrations and is highest when HIO_3 is at its maximum (Figs. S4-5). **While the detection is occasional**, quantum chemical modelling studies also showed that DMA can accept a proton from HIO_3 as a base, forming a stable cluster (DMA-HIO_2-HIO_3) [38], **supporting DMA as a potential stabilising species**."

Comment: Line 375: HOI is the primary form and should be stated explicitly for clarity. Please also add it to Figure 5 to avoid ambiguity when it is referenced later.

Response: We include HOI here, and have also added it to Figure 5.

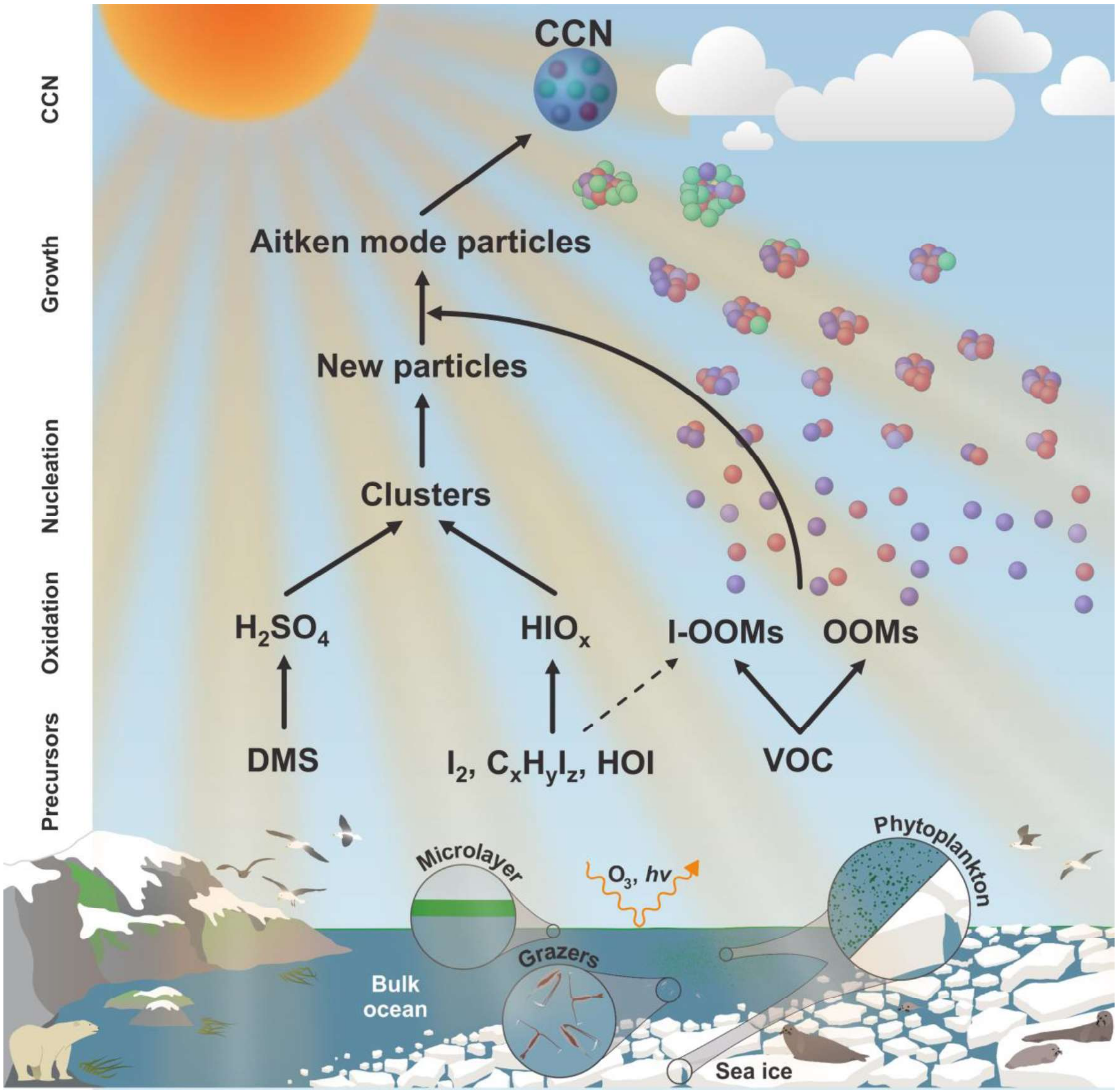

Comment: Lines 386–393: This is a valid and well-made point - earlier studies may indeed have missed an important opportunity. For instance, the NH3 concentrations in Beck et al. (2021) were back-calculated, which suggests the present study is not necessarily in conflict with some prior work. A sentence or two acknowledging that certain earlier studies may have lacked the opportunity to detect this directly would strengthen the discussion.

Response: We agree. We now say the following

"Previous Arctic studies have identified H_2SO_4-NH_3 and HIO_x nucleation as key processes[13,14]. HIO_x, H_2SO_4 and sometimes mixed iodine-sulfur clusters were also detected[13,14]. This suggests that the HIO_x-H_2SO_4 nucleation process may have been present in their observations, though they lacked the opportunity to identify its potential importance."

Reviewer #7 (Remarks to the Author):

Comment: I have reviewed the revised (Round 2) version of the manuscript by Du et al. The authors have addressed my comments and have made substantial improvements to the manuscript.

In particular, the authors have moderated claims that were previously not fully evidence-supported, improving the overall balance and credibility of the study. They have also better placed their findings in clearer context with the existing literature. They incorporated the requested uncertainty analysis for the J1.7 measurements, which strengthens the comparison of their results to the He et al study. In addition, the manuscript has been effectively restructured, with key material now presented in the main text and supporting details appropriately moved to the Supplementary Information.

Overall, the revisions have enhanced the clarity and rigor of the work, and I recommend it for publication.

Response: We thank the reviewer for the positive assessment, and all of the assistance in improving our manuscript.
